# Ameliorative effect and mechanism of ursodeoxycholic acid on hydrogen peroxide-induced hepatocyte injury

**DOI:** 10.1038/s41598-024-55043-3

**Published:** 2024-02-23

**Authors:** Xueqin Wang, Guangxi Liang, Yang Zhou, Banggao Ni, Xiangyu Zhou

**Affiliations:** 1 Department of Thyroid Surgery, Affiliated Hospital of Southwest Medical University, Luzhou, 646000 Sichuan China; 2 Center for Endocrine and Thyroid Diseases, Deyang People’s Hospital, Deyang, 618000 Sichuan China; 3 Department of Vascular Surgery, Deyang People’s Hospital, Deyang, 618000 Sichuan China

**Keywords:** Ursodeoxycholic acid, Oxidative stress, LO2, H_2_O_2_, Diseases, Health care, Pathogenesis

## Abstract

To assess the ameliorative effect of ursodeoxycholic acid (UDCA) on hydrogen peroxide (H_2_O_2_)-induced hepatocyte injury. In our in vivo experiments, we modelled hyperlipidemia in ApoE^−/−^ mice subjected to a 3-month high-fat diet and found that HE staining of the liver showed severe liver injury and excessive H_2_O_2_ was detected in the serum. We modelled oxidative stress injury in L02 cells by H_2_O_2_ in vitro and analyzed the levels of reactive oxygen species (ROS), malondialdehyde (MDA), superoxide dismutase (SOD) and related genes. UDCA significantly improved the level of oxidative stress in H_2_O_2_-injured L02 cells (P < 0.05). In addition, UDCA improved the transcription levels of inflammation and oxidative stress-related genes (P < 0.05), showing anti-inflammatory and anti-oxidative stress effects. UDCA has a protective effect on H_2_O_2_-damaged L02 cells, which lays a theoretical foundation for its application development.

## Introduction

At present, the number of people suffering from metabolic diseases is increasing, and the most common ones are atherosclerosis, myocardial infarction, cerebrovascular accident, peripheral vascular disease, high cholesterol lipids, hyperlipidemia, obesity, diabetes mellitus and so on, which seriously jeopardize people's physical and mental health^1–4^. Regarding the development of metabolic diseases, common pathological mechanisms include chronic inflammation, lipid metabolism, necrosis, apoptosis and oxidative stress^5,6^. Current research on mechanisms in the context of metabolic diseases is focused more on the first three areas, while relatively few studies have been conducted in the direction of oxidative stress. As we know, oxidative stress is associated with the development of many diseases, and the excess oxygen radicals or reactive oxygen species (ROS) it causes can lead to large-scale damage to DNA and cellular macromolecules, such as altering membrane fluidity and permeability, destroying the cytoskeleton and promoting protein denaturation^7–9^. This can ultimately lead to cellular dysfunction, including apoptosis and cancer^10–13^. Therefore, anti-oxidative stress therapy has become one of the most effective strategies to improve the disease process. The liver is an important organ for lipid metabolism, and liver injury is a common pathological damage to the liver, as well as one of the important causes of liver fibrosis, cirrhosis and hepatocellular carcinoma^14,15^. In many in vivo experiments on hyperlipidemia, it has been found that the liver undergoes corresponding damage changes during this disease^16^. The liver injury process is closely related to oxidative stress (OS). OS leads to excessive oxidation of liver cell membrane lipids, interferes with cellular energy metabolism and ultimately leads to cell death^17,18^.

Hydrogen peroxide (H_2_O_2_) is a commonly used inducer of cellular oxidative stress^19,20^, which can trigger cellular stress injury through the imbalance of oxidative and antioxidant systems, which is manifested by the excessive accumulation of intracellular free radicals, leading to cellular damage and secondary apoptosis^21–23^. Hepatocellular injury in various liver diseases can lead to apoptosis, and inhibition of apoptosis can protect against hepatocellular injury, so the use of apoptosis inhibitors will provide a new way to prevent and control various liver injuries in the clinic. Ursodeoxycholic acid (UDCA) is a classical drug used to treat cholestasis in clinical practice, and it has shown good improvement in various liver diseases such as alcoholic liver injury, fatty liver and hepatocellular carcinoma^24^. Clinical studies have found that UDCA alone prevents the development of hepatic steatosis^25^, and in conjunction with Atorvastatin^26^ and Bezafibrate^27^, there is also an enhancement of the treatment of some cirrhotic diseases. And recent studies have confirmed that UDCA can be used as a treatment for atypical coronavirus pneumonia^28^. The use of UDCA has been very well documented both clinically and in vivo in animals, but hyperlipidemia is a complex process in which peroxidation and antioxidant dysregulation are very important. And there are no studies addressing this pathogenesis, so we are interested in whether UDCA can also treat hyperlipidemic liver injury and plan to validate it both in vivo and in vitro. Given the important role of oxidative stress in liver diseases, in this paper, we used H_2_O_2_-induced hepatocyte L02 cell to establish a model of oxidative stress-induced cell injury and analyzed the protective effect of UDCA on it to lay a theoretical foundation for the hepatoprotective effect of UDCA.

## Materials and methods

### Materials and instruments

UDCA (MCE, cat: HY-13771); fetal bovine serum (Biological Industries); trypsin (Beyotime, cat: C0201), penicillin–streptomycin (Beyotime, cat: C0222), reactive oxygen fluorescent probe (DCFH-DA, Yeasen, cat: 50101ES01); reverse transcription kit (Vazyme; code: R333-01); DMEM medium (Gibco), hydrogen peroxide (H_2_O_2_, Sigma, CAS-No: 7722-84-1); SYBR Green Mix (Vazyme); CCK-8 kit (Beyotime, cat: C0037); H_2_O_2_ kit (Solarbio, cat: BC353); and H_2_O_2_ kit (Solarbio, cat: BC3595); MDA kit (Beyotime; cat: S0131S); SOD kit (Beyotime; cat: S0101S); ROS kit (Yeasen; cat: 50101ES01).

Enzyme Labeling Instrument (BioTek, cat: Cytation3); Fluorescence Quantitative PCR Instrument (Applied Biosystems, USA, ABI PRISM®7900HT system).

### Methods

#### Cell and animal cultures

L02 cells were grown in HG medium containing 10% fetal bovine serum, 1% penicillin–streptomycin double antibody, and cultured at 37 °C in 5% CO_2_.

Male ApoE^−/−^ (18 ± 20 g) was provided by Chengdu Pharmachem. Under standard specific pathogen-free (SPF) conditions at the Animal Experimentation Center of Southwest Medical University, the control group was given a normal diet and the model group was given a high-fat diet (Research Diets; cat: D12108C) in which the treatment group was given a high-fat diet along with UDCA-containing water, and the UCDA group was given a normal diet with UDCA-containing water (n = 6 per group). The body weights were measured and recorded weekly, and all animals were taken at 20 weeks for testing. All animal experiments were performed with the approval of the Southwest Medical University Animal Care and Use Committee (License No. 20210223-231), and the ARRIVE guideline. All methods were carried out in accordance with the relevant guidelines of the Southwest Medical University and the ARRIVE guideline, including any relevant details.

#### Biochemical detection

For sampling, blood was taken from the eyes of mice after anesthesia, loaded into 1.5 mL EP tubes (with 1% sodium heparin anticoagulant added beforehand), mixed thoroughly, and then left to stand for 30 min, centrifuged at 4000 rpm for 10 min, and the upper layer of serum was retained in a refrigerator and stored at − 80 °C and sent to the Laboratory Department of the Affiliated Hospital of Southwestern Medical University for testing within 1 month.

#### Histological observations

Mice were anaesthetized and executed, and the livers were removed and fixed in 4% paraformaldehyde for 48 h^29^. Paraffin-embedded tissue sections, 5 μm thick^30,31^, were stained with H&E and photographed.

#### H_2_O_2_ detection

The reagent solution was added to each 100 μL of serum and allowed to stand at room temperature for 5 min. 200 μL was transferred to a 96-well plate and the absorbance value at 415 nm was measured using a multifunctional enzyme marker (BioTek; Synergy H1).

#### Modeling of oxidative stress in H_2_O_2_-stimulated L02 cells

The same volume of L02 cell suspension (1.2 × 10^4^ cells) was added to each well of a 96-well plate, and after the cells were attached to the wall, different concentrations of H_2_O_2_ (100 μmol/L, 200 μmol/L, 400 μmol/L, 600 μmol/L) were added to the plate and incubated for 1 or 2 h. The cell viability was determined by measuring the absorbance values of the cells at 450 nm by using the serum-free medium containing 10% CCK-8 reagent. The absorbance values of different groups of cells were measured at 450 nm, and the cell viability was calculated to determine the modelling concentration of H_2_O_2_.

#### Effects of UDCA on damaged L02 cells

The same volume of L02 cell suspension was added into 96-well plates (1.2 × 10^4^). The cells were divided into control group, model group (H_2_O_2_ group), and treatment group (100 μmol/L, 200 μmol/L, 300 μmol/L, 400 μmol/L, 500 μmol/L). After the cells were completely attached to the wall, they were incubated without or with different concentrations of UDCA in the culture medium for 24 h, and 200 μmol/L H_2_O_2_ was added in the last 2 h. After that, cell viability was detected.

#### Detection of oxidative stress levels

Malondialdehyde (MDA): treated cells were lysed with lysis buffer, centrifuged at 12,000*g* for 10 min, supernatant was removed, MDA assay working solution was added, mixed and heated at 100 °C for 15 min. Cool to room temperature, then centrifuge at 1000*g* for 10 min at room temperature. 200 μL of supernatant from each of the three sub wells in each group was added to a 96-well plate according to the manufacturer’s protocol using the MDA kit (Beyotime, China, cat: S0131S), followed by measurement of absorbance at 532 nm using a multifunctional enzyme marker (BioTek; Synergy H1).

Superoxide dismutase (SOD): 100 μL of SOD sample preparation solution was added to each petri dish and the cells were lysed thoroughly, then centrifuged at approximately 12,000*g* for 5 min at 4 °C. The supernatant was taken and 160 μL of WST-8/enzyme working solution and 20 μL of reaction starting solution were added to every 20 μL, and the cells were incubated at 37 °C for 30 min according to the manufacturer’s protocol using the MDA kit (Beyotime, China, cat: S0101S). Absorbance was measured at 450 nm using a multifunctional enzyme labeller (BioTek; Synergy H1).

ROS: Cells were incubated with DCFH-DA (Yeasen Biotechnology, China, cat: 50101ES01) at a concentration of 10 μmol/L for 30 min at 37 °C in a cell culture incubator, washed to remove the DCFH-DA that did not enter the cells, and then visualized using fluorescence microscopy at 488 nm.

#### Real-time quantitative fluorescence PCR reaction (qRT-PCR) analysis

Cells were harvested and total RNA was extracted using TRIzol reagent (Solarbio Biotechnology Company, China). The yield and purity of extracted RNA were determined using a Nanodrop spectrophotometer (Thermo Scientific, USA). And cDNA was synthesized from 1 μg of total RNA using the Vazyme cDNA RT kit (Takara, Japan) at 50 °C for 15 min, 85 °C for 5 s, and 4 °C for storage according to the manufacturer's instructions. QRT-PCR was performed in an ABI PRISM®7900HT system (Applied Biosystems, USA) using SYBR Green Real-Time PCR Master Mix (Toyobo, Japan). Three steps were included: 50 °C for 30 s, 95 °C for 5 s per cycle (denaturation), and 58 °C for 20 s (elongation). Transcript levels were quantified relative to GAPDH gene levels using the threshold cycling (2-ΔΔCt) method. Look up the ID number of the gene on NCBI (http://www.ncbi.nlm.nih.gov/). The design was then performed at PrimerBanK by ID (https://pga.mgh.harvard.edu/primerbank/) and returned to NCBI'sPrimer-Blast for validation. Primer sequences are shown in Table 1.

**Table 1:** Primer sequences.

Nrf2 (ID: 4780)	F	TTCCCGGTCACATCGAGAG
	R	TCCTGTTGCATACCGTCTAAATC
HO-1 (ID: 3162)	F	AAGACTGCGTTCCTGCTCAAC
	R	AAAGCCCTACAGCAACTGTCG
IL-1b (ID: 3553)	F	ATGATGGCTTATTACAGTGGCAA
	R	GTCGGAGATTCGTAGCTGGA
IL-6 (ID: 3569)	F	CCTGAACCTTCCAAAGATGGC
	R	TTCACCAGGCAAGTCTCCTCA
NLRP3 (ID: 114548)	F	GATCTTCGCTGCGATCAACAG
	R	CGTGCATTATCTGAACCCCAC
GAPDH (ID: 2597)	F	ACAACTTTGGTATCGTGGAAGG
	R	GCCATCACGCCACAGTTTC

#### Protein immunoblotting

The extracted total protein samples were subjected to SDS-PAGE gel electrophoresis and the membranes were transferred to NC membranes by wet transfer method (GE/General Electric, Cat: 1060002) and rapid closure solution (EpiZyme, Cat: PS108P) for 30 min, and the membranes were washed with membrane washing buffer (TBST) three times for 10 min each time. Primary antibodies IL-6, IL-1$\upbeta$, NLRP-3, NRF2, HO-1 (1:1000) and GAPDH (1:2000) were added, and the membranes were incubated at 4 °C overnight. The membrane was washed three times with TBST for 10 min each time and HRP-labeled secondary antibody (1:5000) (Proteintech) was added. The membranes were incubated at 37 °C for 2 h and the relative expression of proteins was analyzed by Image J image analysis system.

### Statistical analysis

Data results were used for mean ± standard deviation, one-way ANOVA was used to analyze one-way data, and P < 0.05 was considered as significant difference (*P < 0.05, **P < 0.01, ***P < 0.001, ****P < 0.0001, ns: no statistical difference). All data results were graphically analyzed using Graph Pad Prism 9 software.

## Results

### Modeling of hyperlipidemia

**Figure 1:**
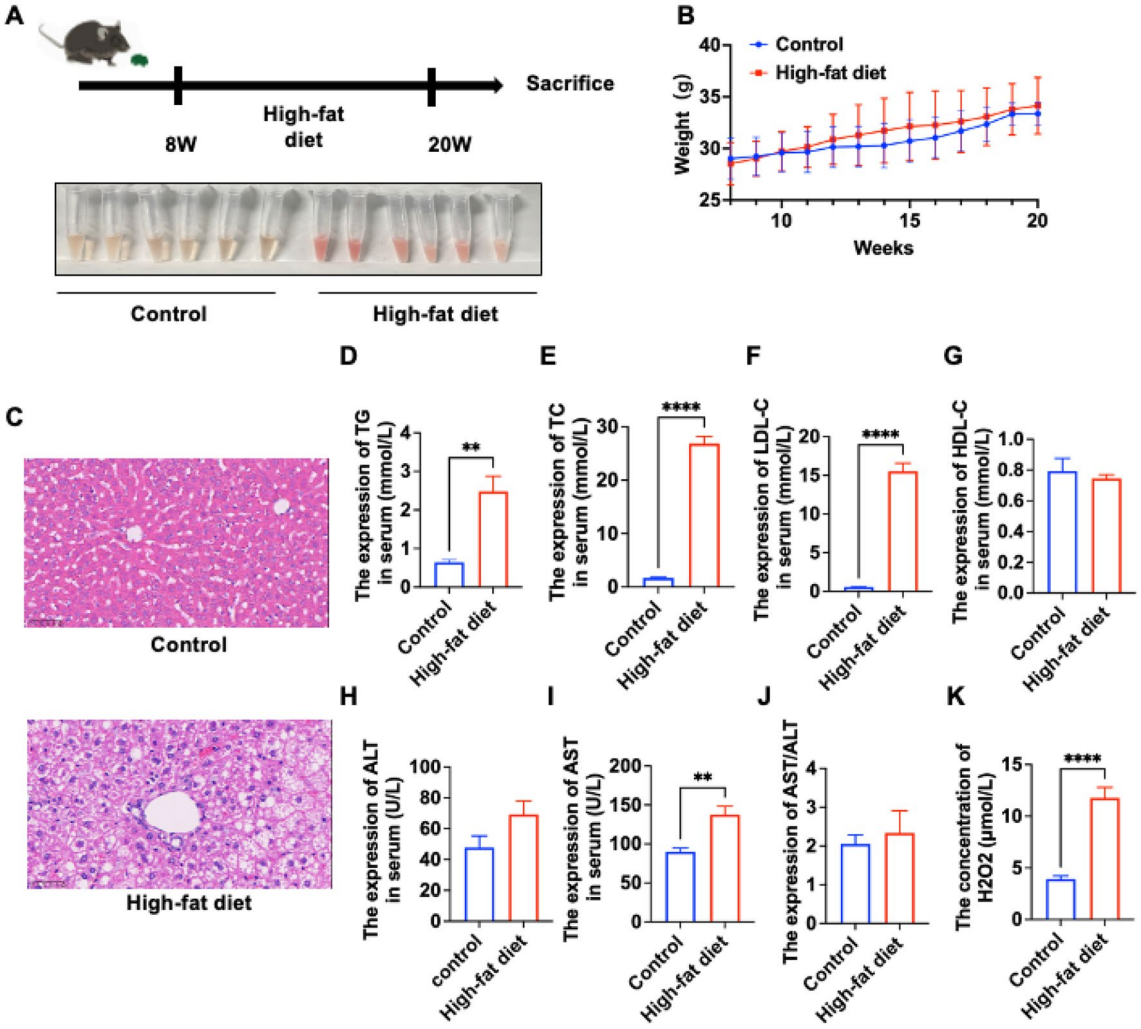
Modeling of hyperlipidemia. (**A**) Pattern diagram of established hyperlipidemia. The photos below show serum collected from both control and high-fat-diet groups taken at 20 weeks (n = 6); (**B**) weight monitoring chart (from 8 to 20 weeks, n = 6); (**C**) liver HE (magnification ×400) of control and high-fat diet groups; (**D–K**) serum results of serum TG (total triglycerides), TC (total cholesterol), LDL-C (low density lipoprotein), HDL-C (high density lipoprotein), ALT (glutamic pyruvic transaminase), AST (glutamic oxaloacetic transaminase), AST/ALT, H_2_O_2_, respectively (**D**–**J** results are from the Laboratory Department of the Affiliated Hospital of Southwest Medical University, **K** from the H_2_O_2_ kit). (** is P < 0.01; *** is P < 0.001; **** is P < 0.0001, high-fat diet vs control).

We first chose the most classical way of modeling hyperlipidemia by feeding a high-fat diet to ApoE^−/−^ mice. As shown in Fig. 1, we started feeding ApoE^−/−^ mice from 8 week (weighing about 28 g) to the end of 20 week, a total of 12 weeks (Fig. 1A); ApoE^−/−^ mice in the control group were fed a normal diet. The mice were in good general health and showed a gradual increase in body weight (Fig. 1B). We obtained serum by taking blood from the eye and centrifuging the supernatant to collect it. It was observed under white light that the serum of the group fed high-fat chow was more turbid, unclear and viscous than that of the control group. Detection of lipid-related indexes by mouse serum revealed that TC, TG and LDL-c were elevated in the model group, and there was a statistically significant difference compared with the control group, while HDL-c was not different from the control group (Fig. 1D–G). This result confirmed the success of our hyperlipidemia model, in which the model group has severe lipid content abnormalities and can be used as a modeling modality for the present experiment. To investigate the changes in the liver under hyperlipidemia and whether it produces changes in liver injury, we compared the HE images of the liver (Fig. 1C), which was morphologically normal in the control mice with no fat accumulation. In the model mice, the liver tissue was disorganized, the cells were swollen, there was a large amount of fat accumulation, and the nuclei were shifted to the edge by lipid droplets. Because we found that hyperlipidemia produces damage to the liver, we further tested the liver function AST, ALT and AST/ALT, and found that the expression difference of AST appeared in the control and model groups and the difference was statistically significant (Fig. 1H–J). Because there is relevant literature suggesting that liver injury is related to H_2_O_2_-induced OS, we examined the changes of H_2_O_2_ in it using serum from mice, and the results were as expected, with an increase in the amount of H_2_O_2_ in the model group compared to the control group (Fig. 1K). From the results of animals with hyperlipidemia, we can find that the content of H_2_O_2_ can be increased in the hyperlipidemic state, which provides a basis for the later use of H_2_O_2_ to simulate one of the oxidative stress models of hyperlipidemia in vitro.

### Determination of H_2_O_2_ injury concentration

**Figure 2:**
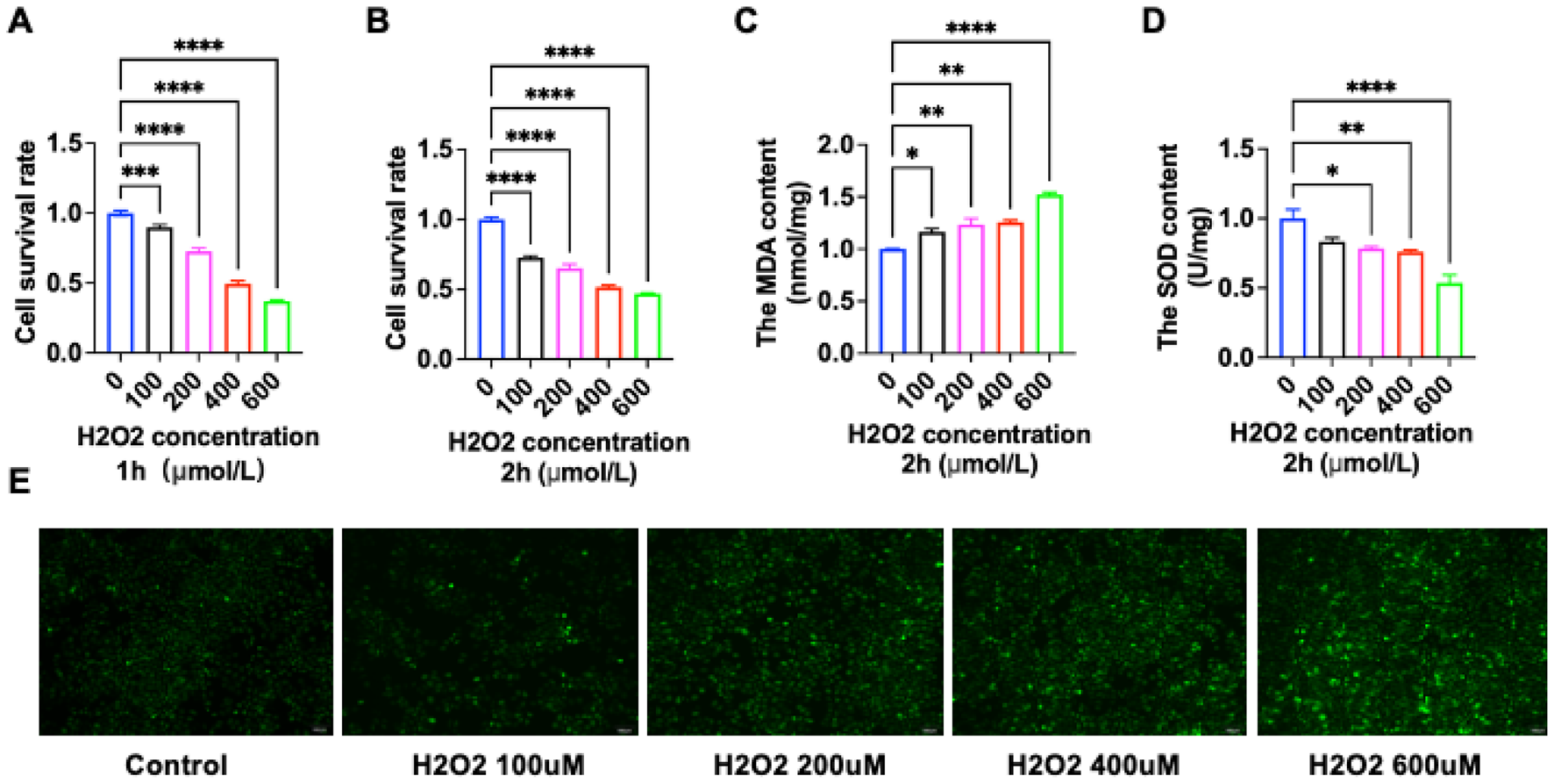
Determination of H_2_O_2_ damage concentration. (**A**) Survival rate of LO2 cells at 1 h with different H_2_O_2_ concentrations tested by Cell Counting Kit-8 (CCK8); (**B**) survival rate of LO2 cells at 2 h with different H_2_O_2_ concentrations tested by CCK8; (**C**) MDA level of LO2 cells at 2 h with different H_2_O_2_ concentrations tested by Lipid Peroxidation MDA Assay Kit; (**D**) SOD level of LO2 cells at 2 h with different H_2_O_2_ concentrations tested by Total Superoxide Dismutase Assay Kit with WST-8; (**E**) fluorescence microscope ROS level of LO2 cells at 2 h with different H_2_O_2_ concentrations; green color is the fluorescence of DCFH-DA at 488 nm entering the cells. (* is P < 0.05; ** is P < 0.01; *** is P < 0.001; **** is P < 0.0001 vs 0 μmol/L).

H_2_O_2_-induced oxidative stress is one of the main mechanisms of hepatocyte injury, which can cause hepatocytes to generate excessive ROS, resulting in hepatocyte injury or even death. The results of our in vivo experiments reveal that the serum of hyperlipidemia produces large amounts of H_2_O_2_. In order to model oxidative stress in hepatocytes in vitro, we intervened with the product of oxidative stress, H_2_O_2_. To utilize the inhibitory effect of H_2_O_2_ on the growth of L02 cells and to find out the optimal dose for inducing L02 cell damage. We observed the changes in the survival rate of L02 cells after treatment with different mass concentrations of H_2_O_2_, and the results are shown in Fig. 2A,B. H_2_O_2_ significantly reduced the survival rate of L02 cells, and when the concentration of H_2_O_2_ was 400 μmol/L and the action time was 2 h, the survival rate of L02 cells was about 51.52%, which was close to the half inhibition rate (Fig. 2B). Next, we further detected the OS-related indexes under the concentration gradient of H_2_O_2_. The green fluorescence intensity of the labeled DCFH-DA probe increased with increasing H_2_O_2_ concentration, suggesting that the accumulation of ROS (Fig. 2E) was concentration-dependent with H_2_O_2_; the expression of another oxidative representative marker, MDA (Fig. 2C), increased, also in a concentration-dependent manner with H_2_O_2_; and the expression level of SOD was negatively correlated with H_2_O_2_ (Fig. 2D). Therefore, in the subsequent study, the model preparation condition was selected as 400 μmol/L H_2_O_2_ treated L02 cells for 2 h.

### Effect of UDCA on the survival of LO2

**Figure 3:**
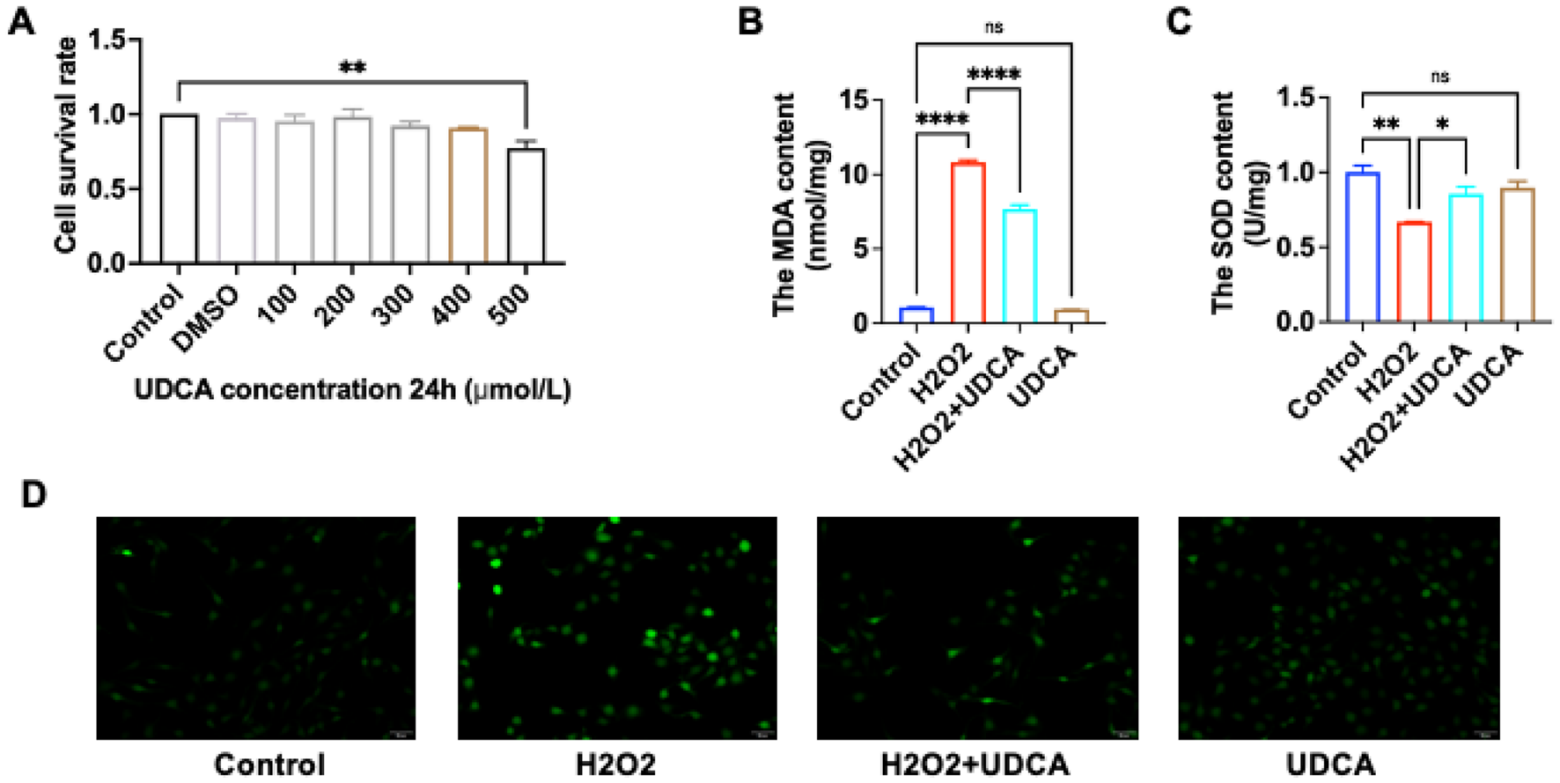
Treatment of H_2_O_2_-induced L02 injury model by UDCA. (**A**) Survival rate of LO2 cells at 24 h with different UDCA concentrations tested by CCK8; (**B**–**D**) are the levels of MDA, SOD and ROS in LO2 cells in the four groups of control, 400 μmol/L H_2_O_2_ treatment for 2 h, 400 μmol/L H_2_O_2_ 2 h + UDCA 24 h, and UDCA 24 h. (* is P < 0.05; ** is P < 0.01; **** is P < 0.0001 vs 0 μmol/L).

To study the effect of UDCA dose on the growth of L02 cells and to determine the safe use dose of UDCA, the changes in cell survival rate when L02 cells were treated with different concentrations of UDCA were observed, and the results are shown in Fig. 3A. When the concentration of UDCA was in the range of 100–400 μmol/L, the survival rate of L02 cells was higher than 90%, and the effect on cell growth was not obvious, which can be regarded as no significant cytotoxicity; whereas, when the concentration of UDCA reached 500 μmol/L, the survival rate of LO2 cells was 77.2%, which had cytotoxic effect. Therefore, 400 μmol/L was selected as the safe dose of UDCA intervention in this experiment for subsequent studies.

### Effect of UDCA on the level of H_2_O_2_-induced oxidative stress in L02 cells

Previous results have shown that injury to hepatocytes with H_2_O_2_ results in severe oxidative stress within hepatocytes and triggers lipid peroxidation of cell membranes, as well as an increase in the intracellular production of ROS. The effect of UDCA on ROS levels in H_2_O_2_-injured L02 cells is shown in Fig. 3D. There was no significant difference in green fluorescence between the UDCA group and Control group, but the intensity of green fluorescence of H_2_O_2_ + UDCA labeled with DCFH-DA probe was reduced compared with that of the H_2_O_2_ group, suggesting that UDCA was able to reduce H_2_O_2_-induced ROS generation in L02 cells, suggesting that the UDCA pretreatment reduced the level of oxidative stress in L02 cells induced by H_2_O_2_. We then examined the levels of MDA (Fig. 3B) and SOD (Fig. 3C), and there was no significant difference in MDA and SOD between the UDCA group and the Control group, but H_2_O_2_ + UDCA decreased MDA and increased SOD compared to the H_2_O_2_ group, and the differences were all statistically different. This experimental result suggests that pretreatment of L02 with 400 μmol/L UDCA reverses the H_2_O_2_-induced changes.

### Improvement of oxidative stress levels under hyperlipidemia by UDCA in vivo

**Figure 4:**
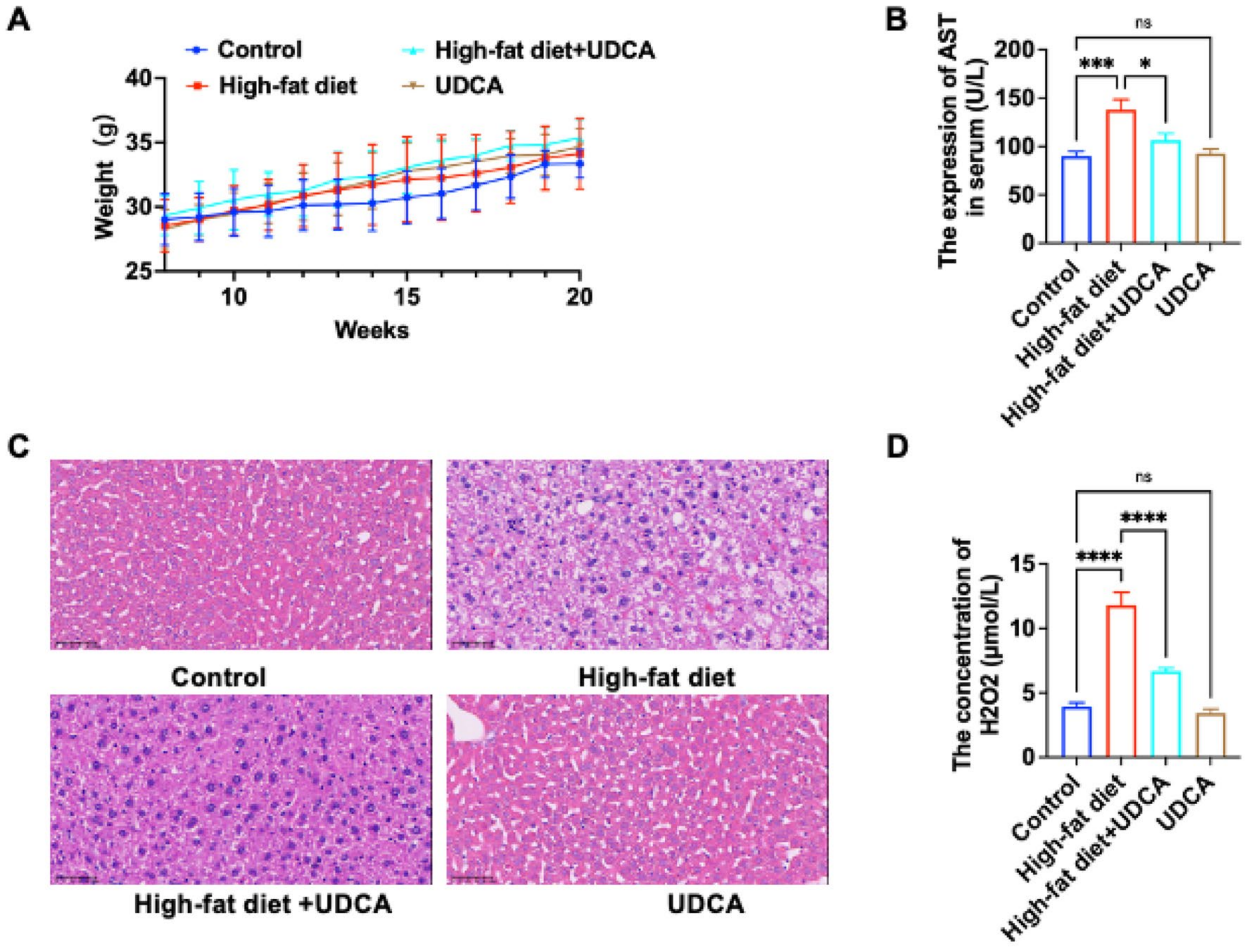
UDCA for treatment of liver injury under hyperlipidemia. (**A**) Control, high-fat diet for 12 weeks, high-fat diet for 12 weeks + 50 mg/kg UDCA drinking water for 12 weeks, and 50 mg/kg UDCA drinking water for 12 weeks weight-monitoring graphs; (**B**) results of serum AST test in the four groups; (**C**) liver HE graphs in the four groups (magnification of ×400); (**D**) serum H_2_O_2_ levels in the four groups. (* is P < 0.05; *** is P < 0.001; **** is P < 0.0001 vs control).

To validate the protective effect of UDCA against liver injury under hyperlipidemia model in vivo as well. Our treatment group added 50 mg/kg UDCA drug inside the drinking water along with a high-fat diet and set up a drug-only group drinking only UDCA. During the treatment period, we detected the weight changes in the mice (Fig. 4A), a positive trend of steady growth, which did not cause discomfort or death in the mice, confirming the safety of the drug. In detecting the improvement of liver AST, the high-fat + UCDA group showed significant improvement compared with the high-fat model group (Fig. 4B). In the HE results of the liver, UDCA alone did not damage the liver, and the liver cell swelling was significantly reduced, and vacuolated lipid droplets were significantly reduced in the high-fat + UCDA group compared with the high-fat model group of mice, which indicated that the consumption of UDCA reduced the accumulation of liver fat (Fig. 4C). Further testing of serum H_2_O_2_ content, UDCA alone did not change the serum H_2_O_2_ content, while H_2_O_2_ was significantly reduced in the high-fat + UCDA group compared with the high-fat model group of mice, which indicates that under the pathological conditions of hyperlipidemia, treatment with UDCA can reduce the level of serum H_2_O_2_ and decrease the degree of hepatic injury it induces (Fig. 4D).

### Effect of UDCA on H_2_O_2_-induced expression of inflammatory-related proteins in L02 cells

**Figure 5:**
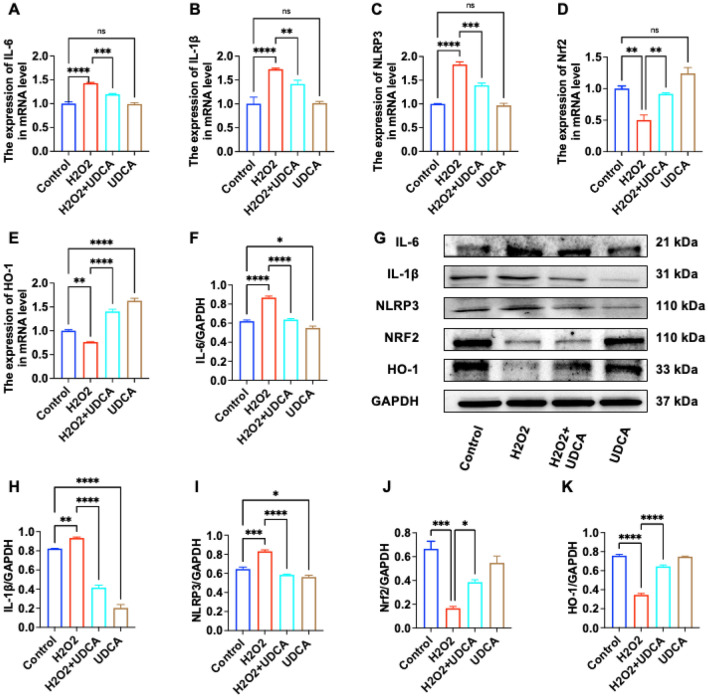
Effect of UDCA on the expression of anti-inflammatory proteins in L02. (**A–E**) The results of mRNA detection in LO2 cells for the four groups of Control, 400 μmol/L H_2_O_2_ treatment for 2 h, 400 μmol/L H_2_O_2_ 2 h + UDCA 24 h, and UDCA 24 h; (**G**) the result of WB, the samples derive from the same experiment and that gels were processed in parallel. The original blots are shown in Supplementary Figures; (**F**,**H–K**) are the bar charts of the G graph. (* is P < 0.05; ** is P < 0.01; *** is P < 0.001; **** is P < 0.0001 vs control).

First, we examined the classical inflammatory indicators IL-1β, IL-6 and NLRP3 at the mRNA level, and the levels of IL-1β, IL-6 and NLRP3 were elevated in the L02 cells of the H_2_O_2_ model group compared with those of the normal group (P < 0.05), confirming the successful establishment of the cellular model, and that H_2_O_2_ could cause the elevation of the inflammatory indicators on the mRNA level of L02 cells. On the basis of this result, we further examined the protein levels of these genes (Fig. 5F–K), and the results were consistent with their mRNA levels, and the protein expression levels of IL-1β, IL-6, and NLRP3 were elevated in L02 cells in the H_2_O_2_ model group compared with the normal group (P < 0.05), and H_2_O_2_ could cause the protein levels of L02 cells on the elevation of inflammatory indexes. Then, we detected the downregulation of anti-inflammatory protein expression such as cellular NRF2 and HO-1 (P < 0.05); the levels of IL-1β, IL-6 and NLRP3 were decreased in L02 cells in the H_2_O_2_ + UDCA group compared to the H_2_O_2_ group (P < 0.05). Anti-inflammatory proteins such as NRF2 and HO-1 were up-regulated (P < 0.05). Thus, we suggest from our experimental results that the reparative effect of UDCA on H_2_O_2_-induced LO2 injury may be through increased expression of NRF2/HO-1 anti-inflammatory molecules.

## Discussion

Hepatocellular injury is a common pathological basis of many liver diseases^9,32^. One of its potentially important mechanisms is ROS-induced oxidative stress, which underlies all forms of hepatocellular injury and death. ROS accumulation and a significant increase in inflammatory response have been shown to play a crucial role in the onset and progression of hepatic injury. The major sources of ROS are mitochondrial and cytochrome P450 enzymes in hepatocytes, Kupffer cells and neutrophils^33,34^. Normal cells tightly control intracellular ROS levels and maintain a balance between oxidants and antioxidant molecules. An imbalance in this process disrupts the structure–function relationship of hepatocytes in different parts of the body at the cellular and molecular levels. Consistent with previous literature, our experiments also suggested that oxidative damage of liver tissue caused by hyperlipidemia and is accompanied by elevated serum H_2_O_2_ levels, it also confirmed the important role of oxidative stress in hyperlipidemia induced hepatic injury. However, most of the current research has focused on lipid-lowering and anti-inflammatory treatments, ignoring the importance of oxidative imbalance in the hyperlipidemic process. Therefore, exploring potential molecular mechanisms of oxidative stress and seeking new therapeutic strategies are of great clinical value for further elucidating the pathogenesis of hyperlipidemia induced liver injury and improving treatment outcomes. H_2_O_2_, an endogenous ROS in aerobic cells, is a toxic substance that can induce OS^35^. However, many studies conducted over the past two decades have provided substantial evidence that H_2_O_2_ is a diffusible intracellular signaling messenger and common oxidative stressor, which readily crosses cell membranes and leads to hepatocellular cell injury and even death.

In the present study, using H_2_O_2_-induced L02 cell injury^36,37^, we investigated the protective effect of UDCA against oxidative stress injury in hepatocytes. The results showed that H_2_O_2_ reduced the viability of L02 cells, elevated the levels of cellular oxidation products ROS and MDA, and decreased the level of antioxidant SOD, indicating that the model of oxidative damage in L02 cells was successfully constructed. After pretreatment of L02 cells with a safe dose of UDCA, the cellular ROS and MDA production in the UDCA group were significantly reduced, and the SOD level was significantly increased. Existing research confirms that UDCA protected hepatocytes from antagonise Arsenic-induced cytotoxicity by a mechanism that may have enhanced the levels of SOD and GSH, while intracellular ROS and MDA accumulation was reduced in vivo and in vitro^38^. Similarly, our results confirmed that UDCA plays an important protective role in H_2_O_2_-induced levels of oxidative stress in L02 cells. Its potential mechanism is reducing the levels of lipid peroxidation through the activation of antioxidant enzyme activities in the cells.

As demonstrated by countless NRF2 knockout studies in various organs such as lung, liver, kidney, and brain, dysregulation of NRF2 severely affects oxidant/ROS sensitivity and predisposes the system to several pathological changes with aberrant cytopathic properties^39^. The induced activation of NRF2 regulates several genes containing antioxidant response elements (AREs) and brings about their respective translations to counteract reactive radicals and establish homeostasis^40–42^. Therefore, our phenotyping experiments suggest that UDCA can activate the activity of intracellular antioxidant enzymes and inhibit the damage of lipid peroxidation to the membrane system, thus enhancing the tolerance of cells to oxidative stress. On this basis, the expression of the cellular protein NRF2 and its important target gene HO-1 was investigated, and it was found that UDCA promoted the expression of NRF2 and its downstream antioxidant enzyme HO-1, suggesting that the NRF2-associated pathway is a key mechanism by which UDCA exerts its antioxidant biological effects. The fact that UDCA can restore the antioxidant capacity of hepatocytes under oxidative stimulation is consistent with the findings of existing literature, no significant difference between UDCA alone and control is also consistent with the literature^38^. However, the inflammatory indexes were reduced with UDCA alone compared to the control group, which may be attributed to the role of pathways related to the inhibition of Akt/NF-κB and MAPK signaling pathways^43^. This suggests that UDCA may not only attenuate hepatic cholestatic disease but also reduce peroxidative damage in the liver under hyperlipidemia and protect the health of the liver.

## Conclusion

In the current study, we investigated the effect of UDCA on hepatocyte injury and potential molecular mechanisms in the hyperlipidemia mice and in LO2 cells induced by H_2_O_2_, respectively. Our results suggested that UDCA could attenuate the oxidative stress injury of hepatic cell injury, its potential mechanism is to reduce the levels of lipid peroxidation and enhance the anti-inflammatory and antioxidant capacity of NRF2. Nonetheless, there are still some shortcomings in our study. One is that we chose a single animal model to simulate liver injury in hyperlipidemia. In the subsequent experiments, we will use different animal models to further explore the protective mechanism of UDCA against liver cell damage. Moreover, the oxidative stress pathway in vivo is complex, involving endoplasmic reticulum, mitochondria, lysosomal metabolic abnormalities and other possibilities, which may become part of our future work.

### Supplementary Information

Supplementary Information 1.

Supplementary Information 2.

Supplementary Information 3.

Supplementary Information 4.

## Data Availability

All data supporting the findings described in this manuscript are available in the article and its Supplementary Information files.
