# Supplementary material for: Ameliorative effect and mechanism of ursodeoxycholic acid on hydrogen peroxide-induced hepatocyte injury
Source: Sci Rep. 2024 Feb 23;14:4446. doi: 10.1038/s41598-024-55043-3 (PMC10891090; doi:10.1038/s41598-024-55043-3)
Supplement: Supplementary file 1 — Supplementary Information 1. [file 41598_2024_55043_MOESM1_ESM.docx]

**
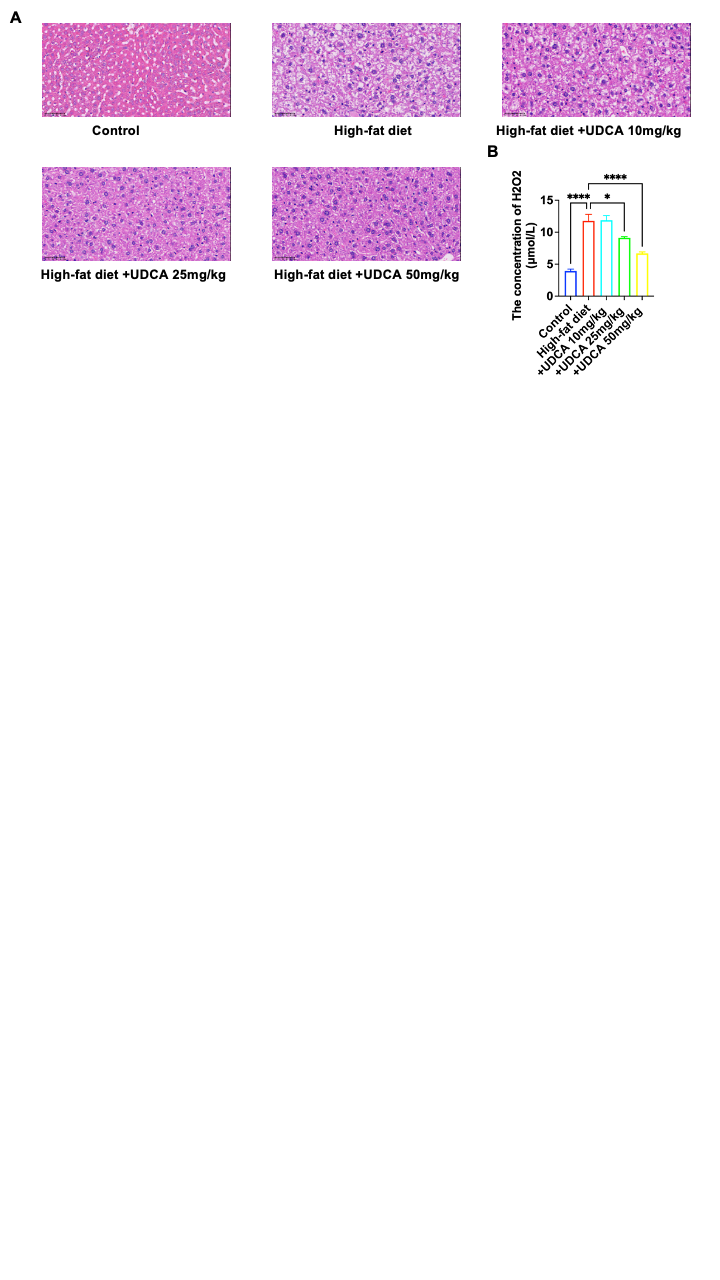
**

**Figure1.** **Treatment of liver injury with different concentrations of UDCA**

**A** Control, High-fat diet for 12 weeks, High-fat diet for 12 weeks + 10 mg/kg UDCA drinking water for 12 weeks, High-fat diet for 12 weeks + 25 mg/kg UDCA drinking water for 12 weeks, High-fat diet for 12 weeks + 50 mg/kg UDCA drinking water for 12 weeks, Liver in five groups HE diagrams (magnification 400X); **B** H2O2 levels of the five groups. (* is p<0.05; **** is p<0.0001)
