# Supplementary figures and images for: Ameliorative effect and mechanism of ursodeoxycholic acid on hydrogen peroxide-induced hepatocyte injury (part 2 of 2)
Source: Sci Rep. 2024 Feb 23;14:4446. doi: 10.1038/s41598-024-55043-3 (PMC10891090; doi:10.1038/s41598-024-55043-3)

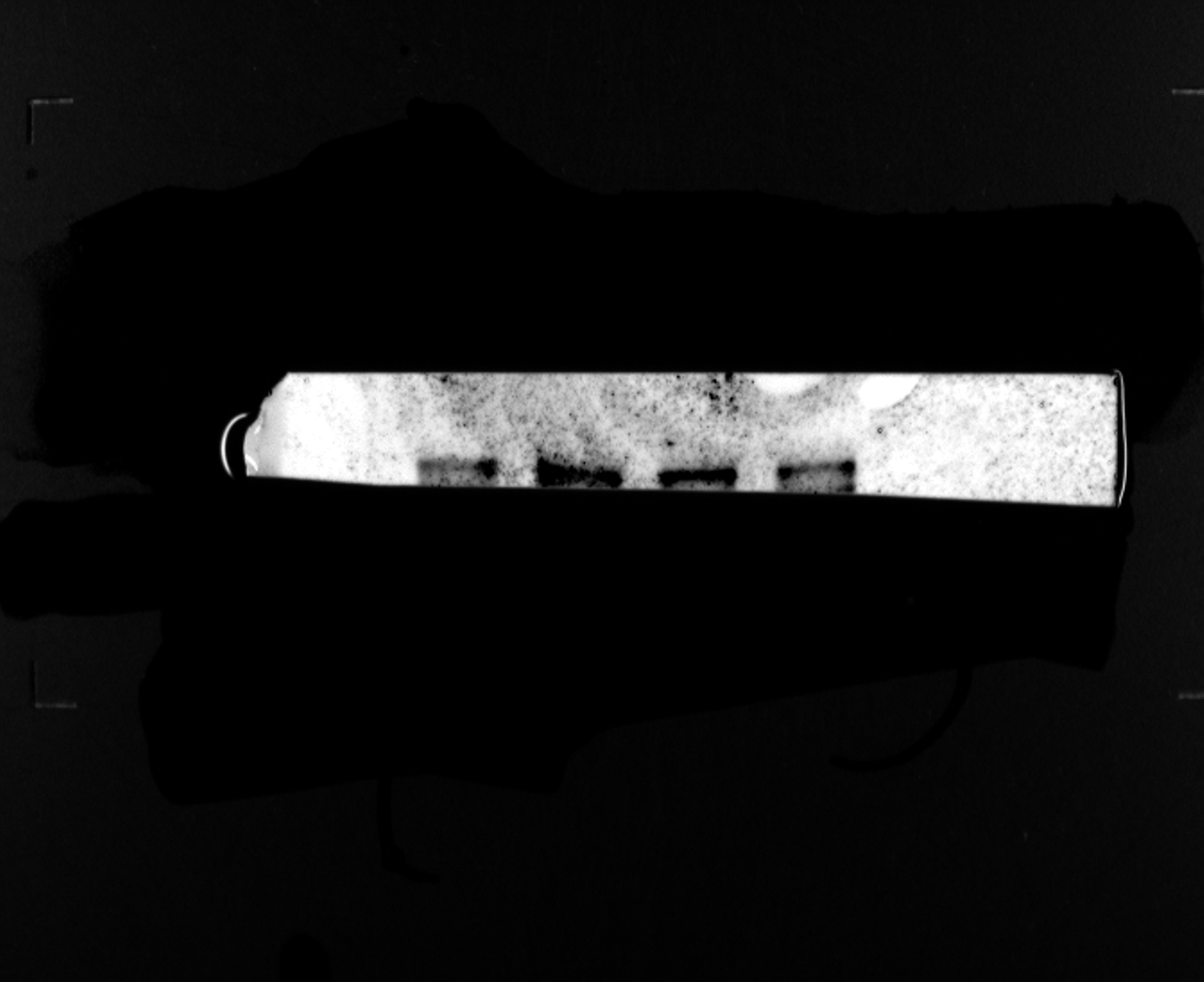

Supplement: Supplementary file 3 — Supplementary Information 3. [file 41598_2024_55043_MOESM3_ESM.zip › Supplementary material/Lo2-WB/IL-6/IL-6.2.Tif]

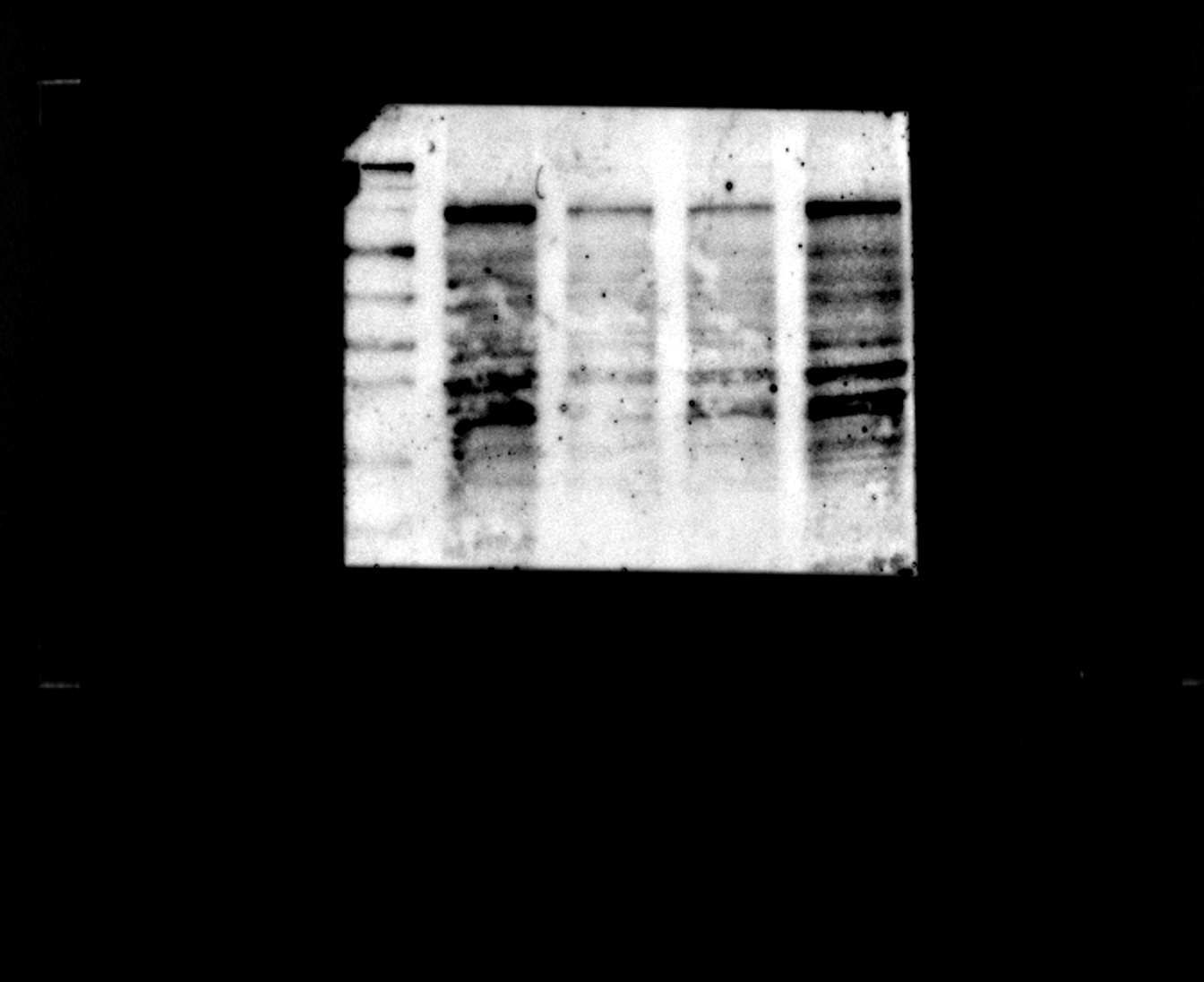

Supplement: Supplementary file 3 — Supplementary Information 3. [file 41598_2024_55043_MOESM3_ESM.zip › Supplementary material/Lo2-WB/NRF-2/NRF2.Tif]

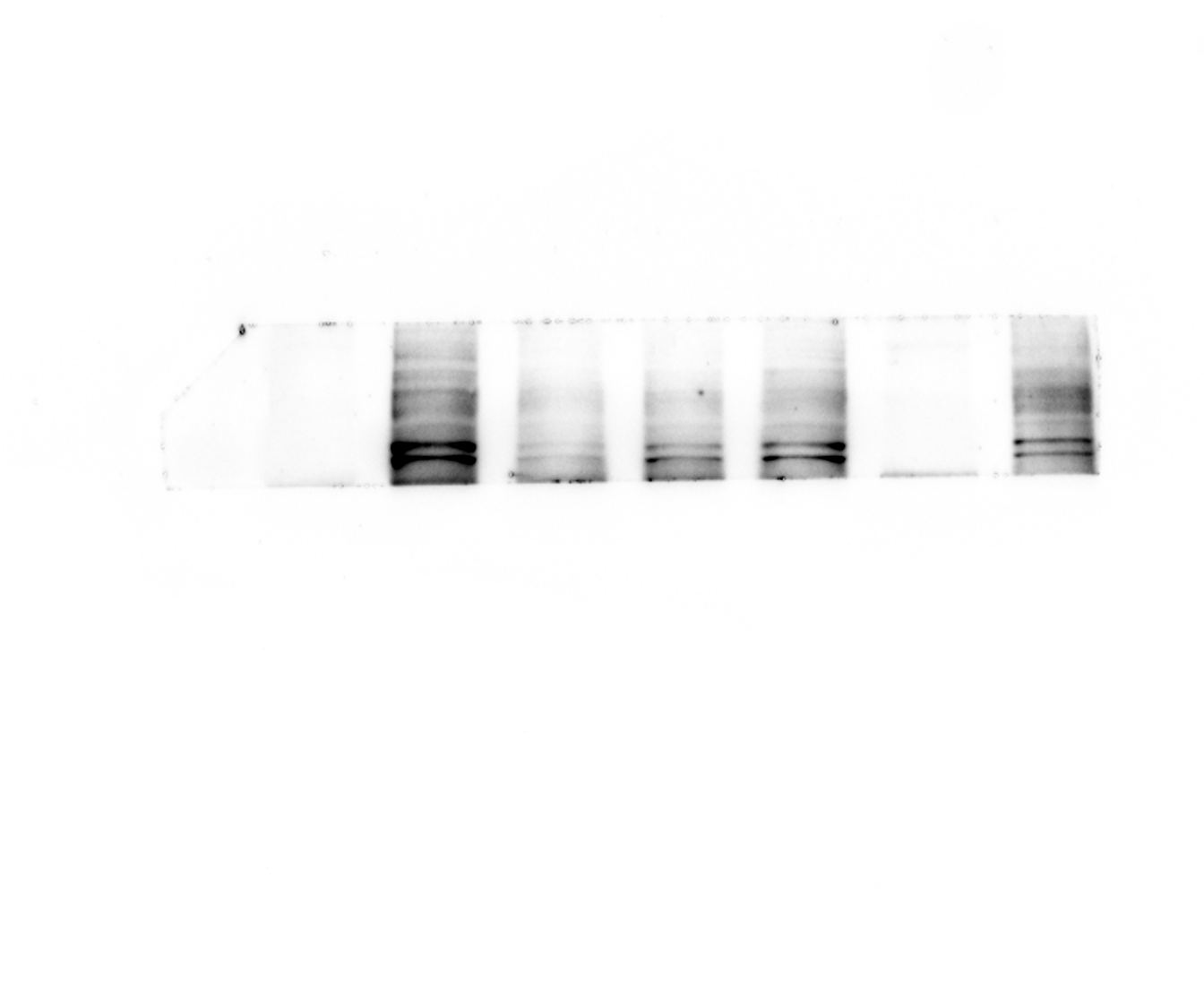

Supplement: Supplementary file 3 — Supplementary Information 3. [file 41598_2024_55043_MOESM3_ESM.zip › Supplementary material/Lo2-WB/NRF-2/NRF2.0.Tif]

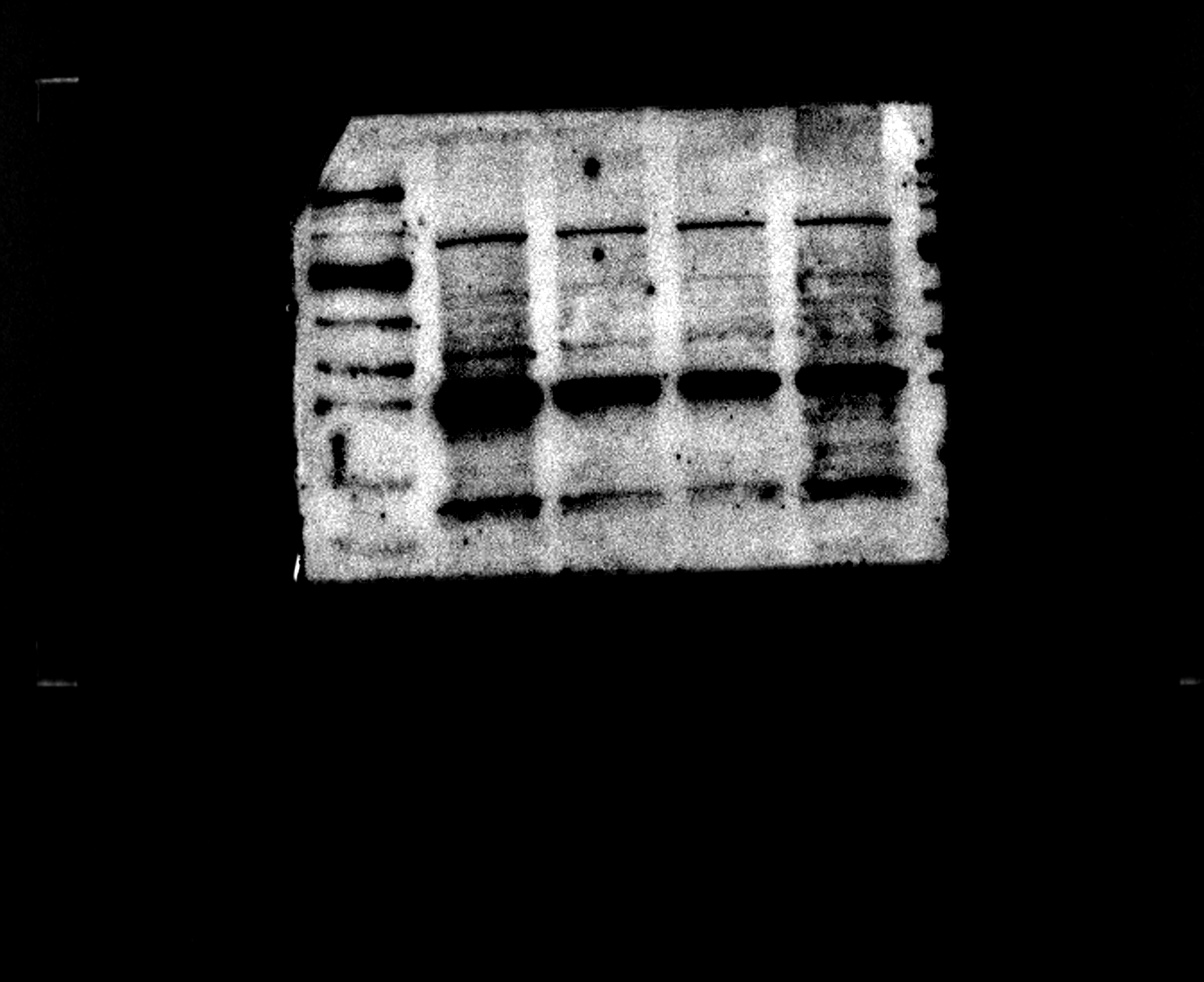

Supplement: Supplementary file 3 — Supplementary Information 3. [file 41598_2024_55043_MOESM3_ESM.zip › Supplementary material/Lo2-WB/NRF-2/NRF-2.Tif]

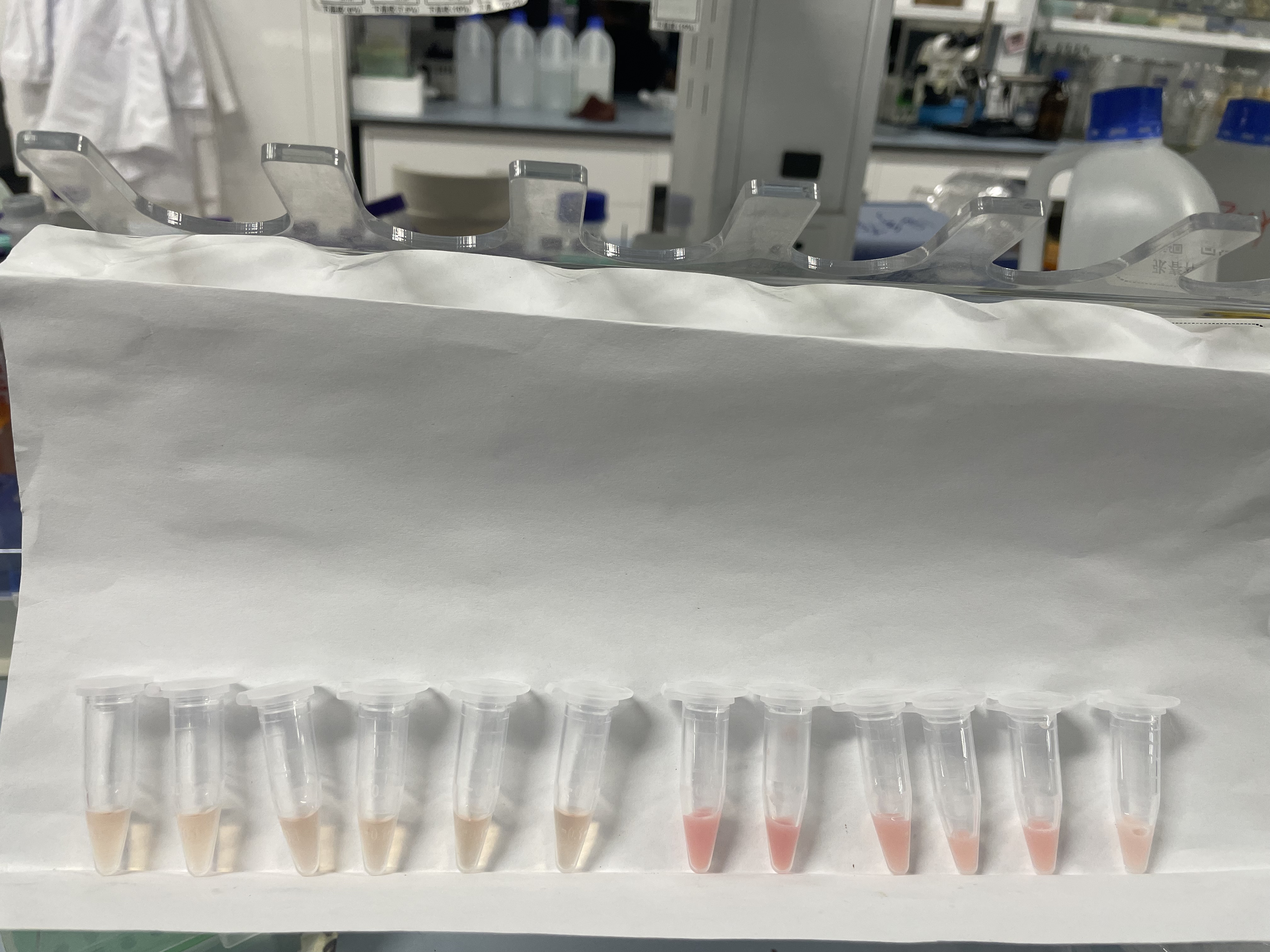

Supplement: Supplementary file 3 — Supplementary Information 3. [file 41598_2024_55043_MOESM3_ESM.zip › Supplementary material/Fig 1/τöƒσîû-ΦíÇΦäé/WechatIMG460.jpeg]

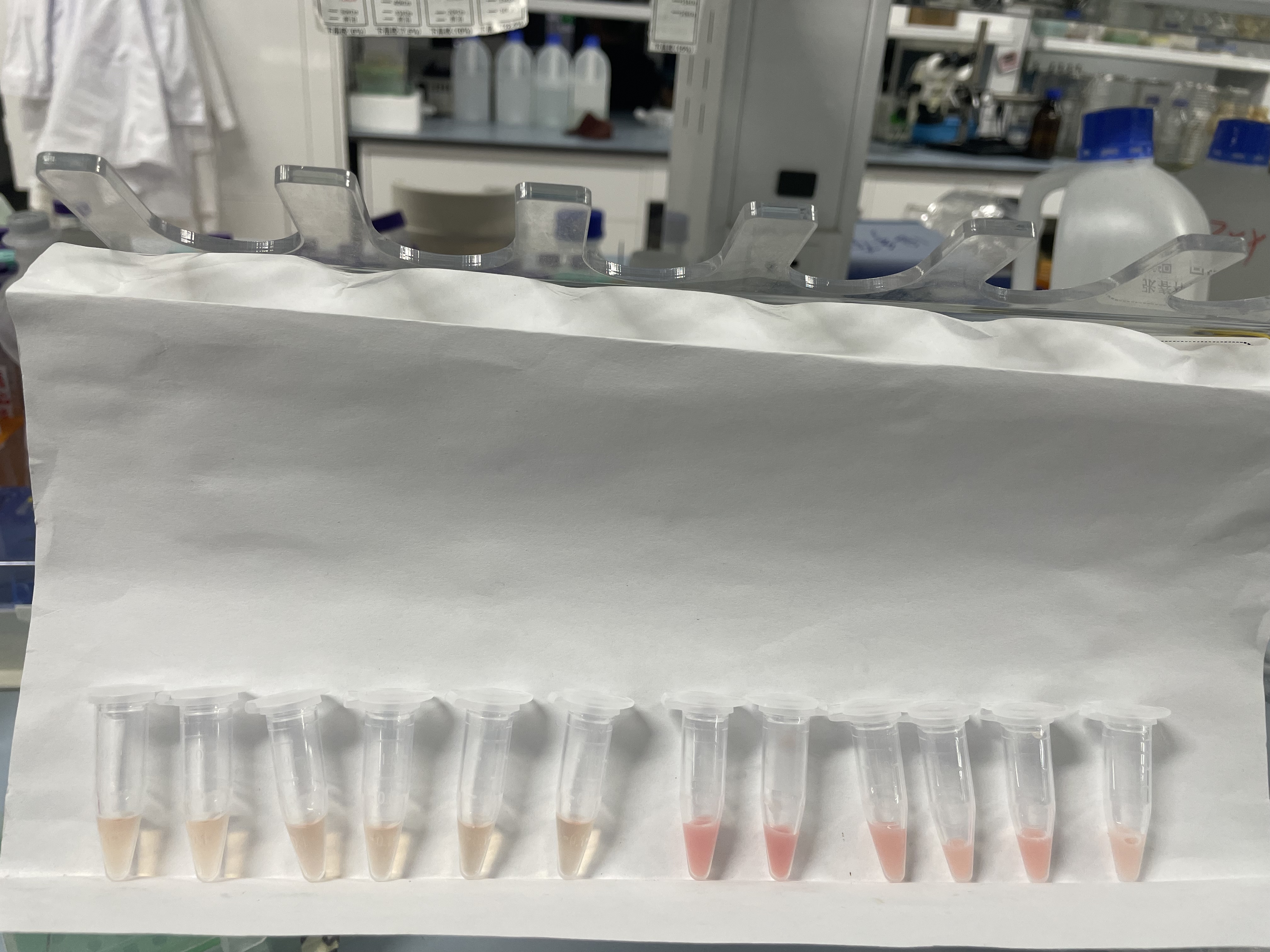

Supplement: Supplementary file 3 — Supplementary Information 3. [file 41598_2024_55043_MOESM3_ESM.zip › Supplementary material/Fig 1/τöƒσîû-ΦíÇΦäé/WechatIMG461.jpeg]

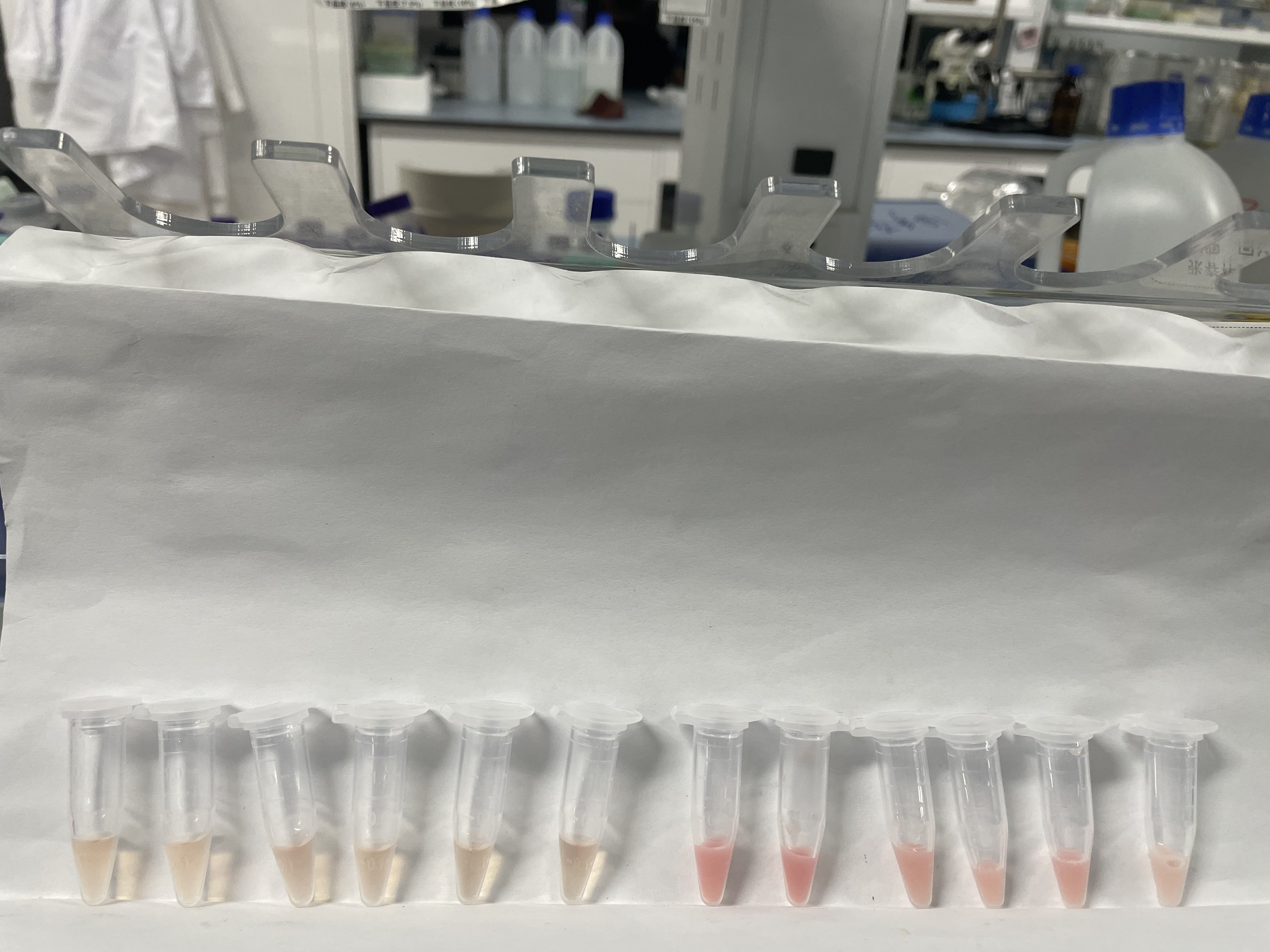

Supplement: Supplementary file 3 — Supplementary Information 3. [file 41598_2024_55043_MOESM3_ESM.zip › Supplementary material/Fig 1/τöƒσîû-ΦíÇΦäé/WechatIMG462.jpeg]

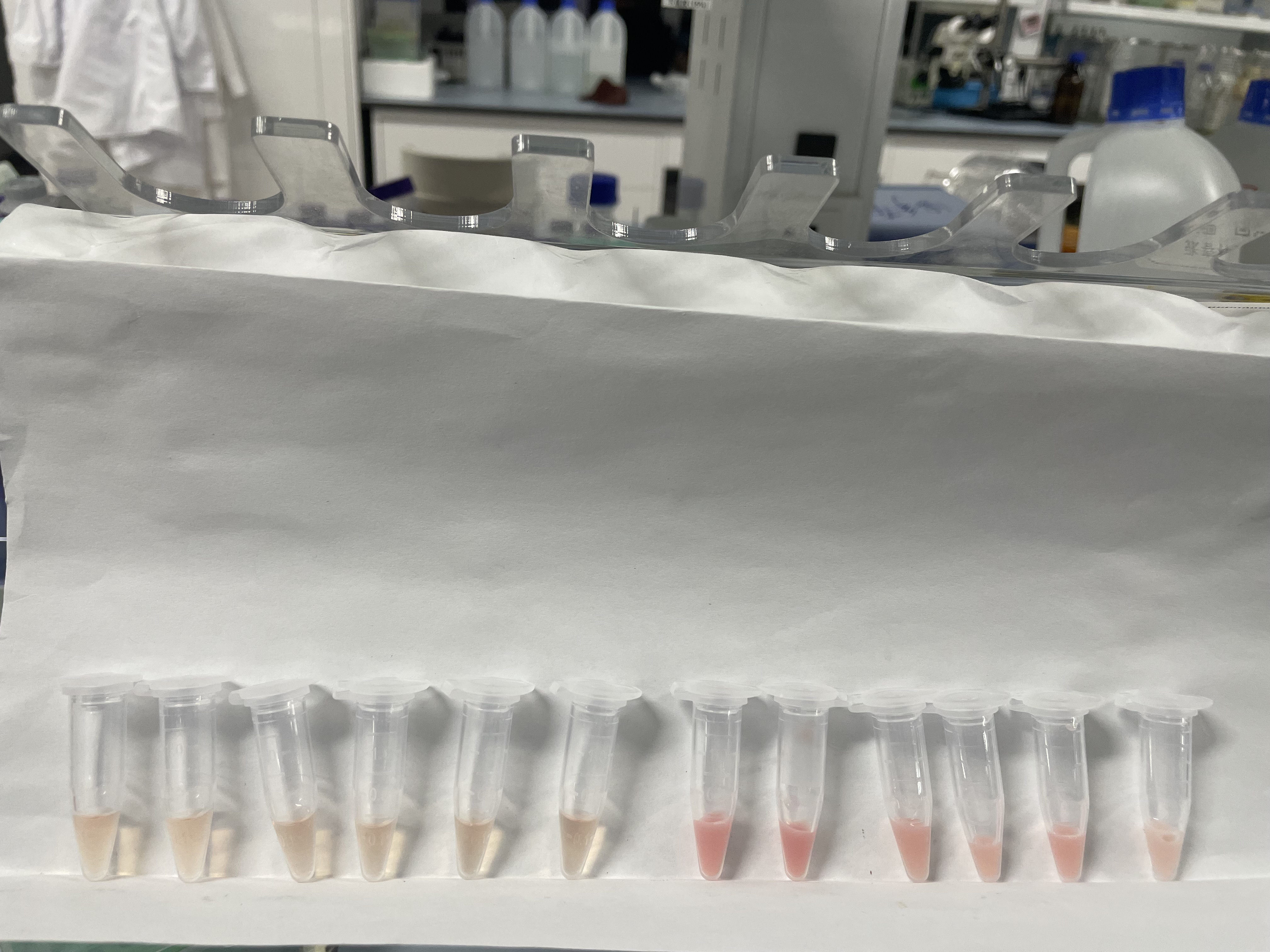

Supplement: Supplementary file 3 — Supplementary Information 3. [file 41598_2024_55043_MOESM3_ESM.zip › Supplementary material/Fig 1/τöƒσîû-ΦíÇΦäé/WechatIMG459.jpeg]

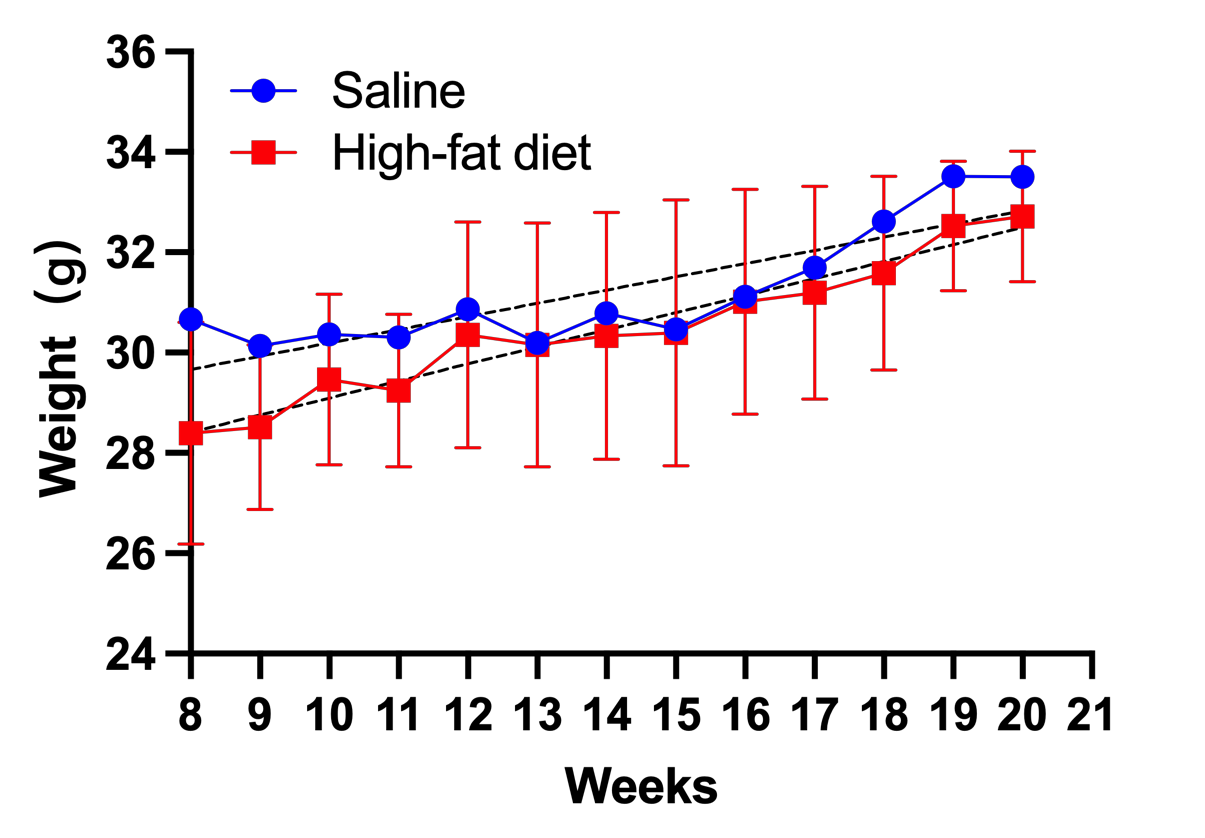

Supplement: Supplementary file 3 — Supplementary Information 3. [file 41598_2024_55043_MOESM3_ESM.zip › Supplementary material/Fig 1/Σ╜ôΘçì/high-fat.tiff]

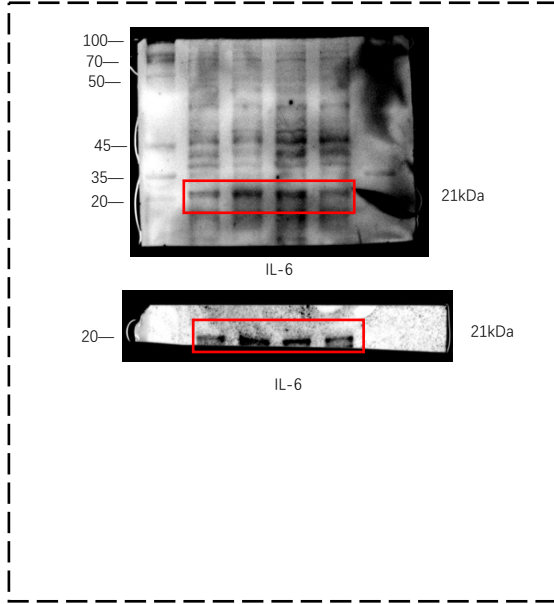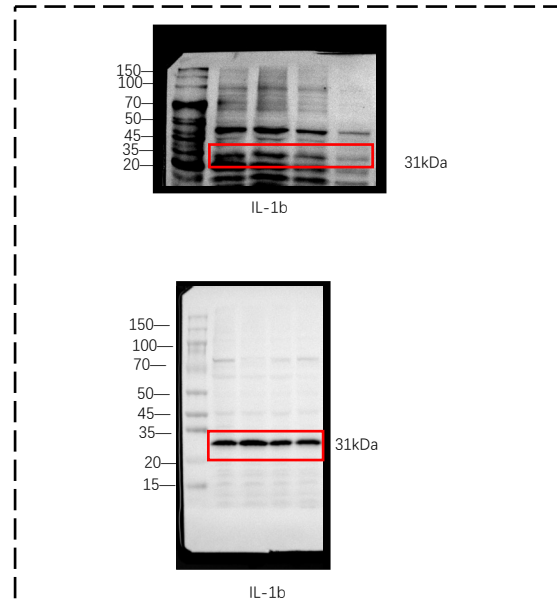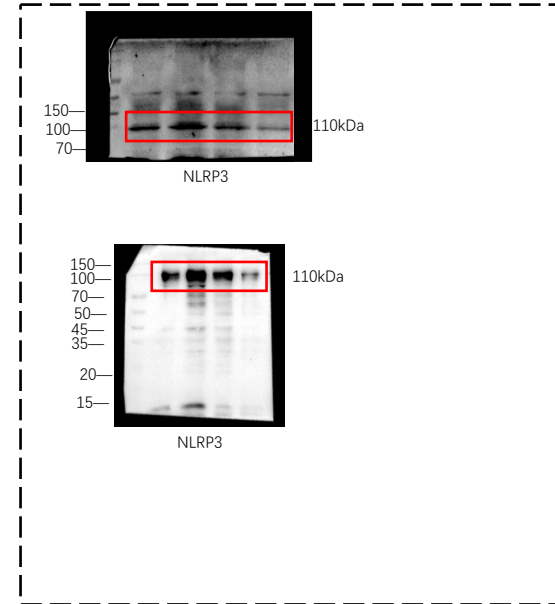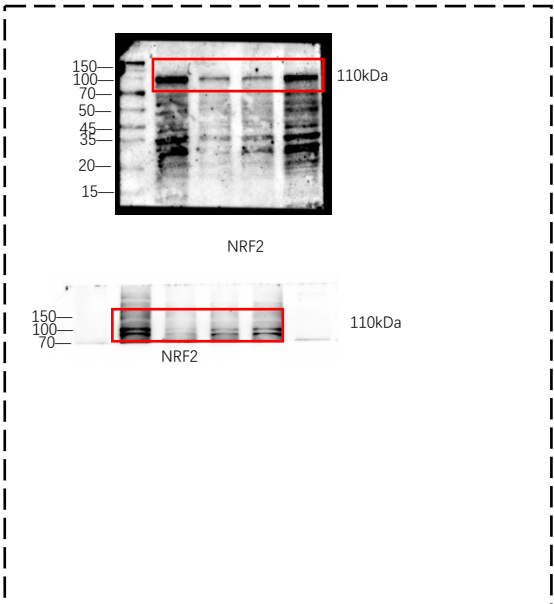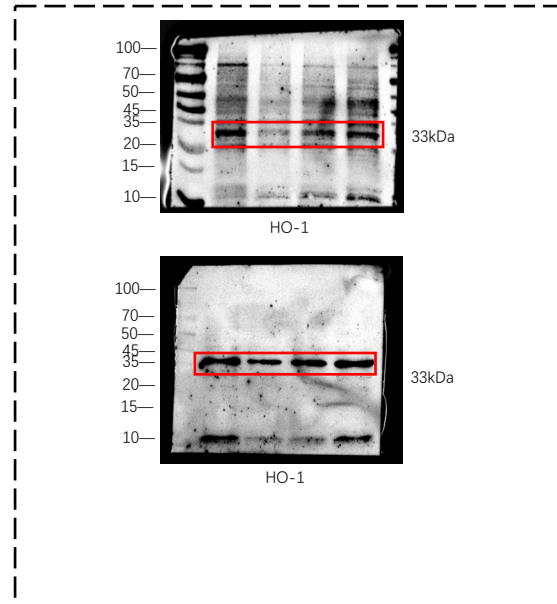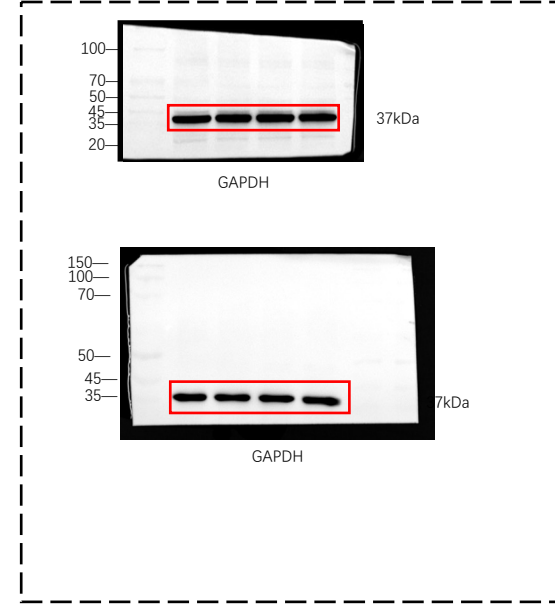

Supplement: Supplementary file 4 — Supplementary Information 4. [file 41598_2024_55043_MOESM4_ESM.pdf]
