# Supplementary figures and images for: Ameliorative effect and mechanism of ursodeoxycholic acid on hydrogen peroxide-induced hepatocyte injury (part 1 of 2)
Source: Sci Rep. 2024 Feb 23;14:4446. doi: 10.1038/s41598-024-55043-3 (PMC10891090; doi:10.1038/s41598-024-55043-3)

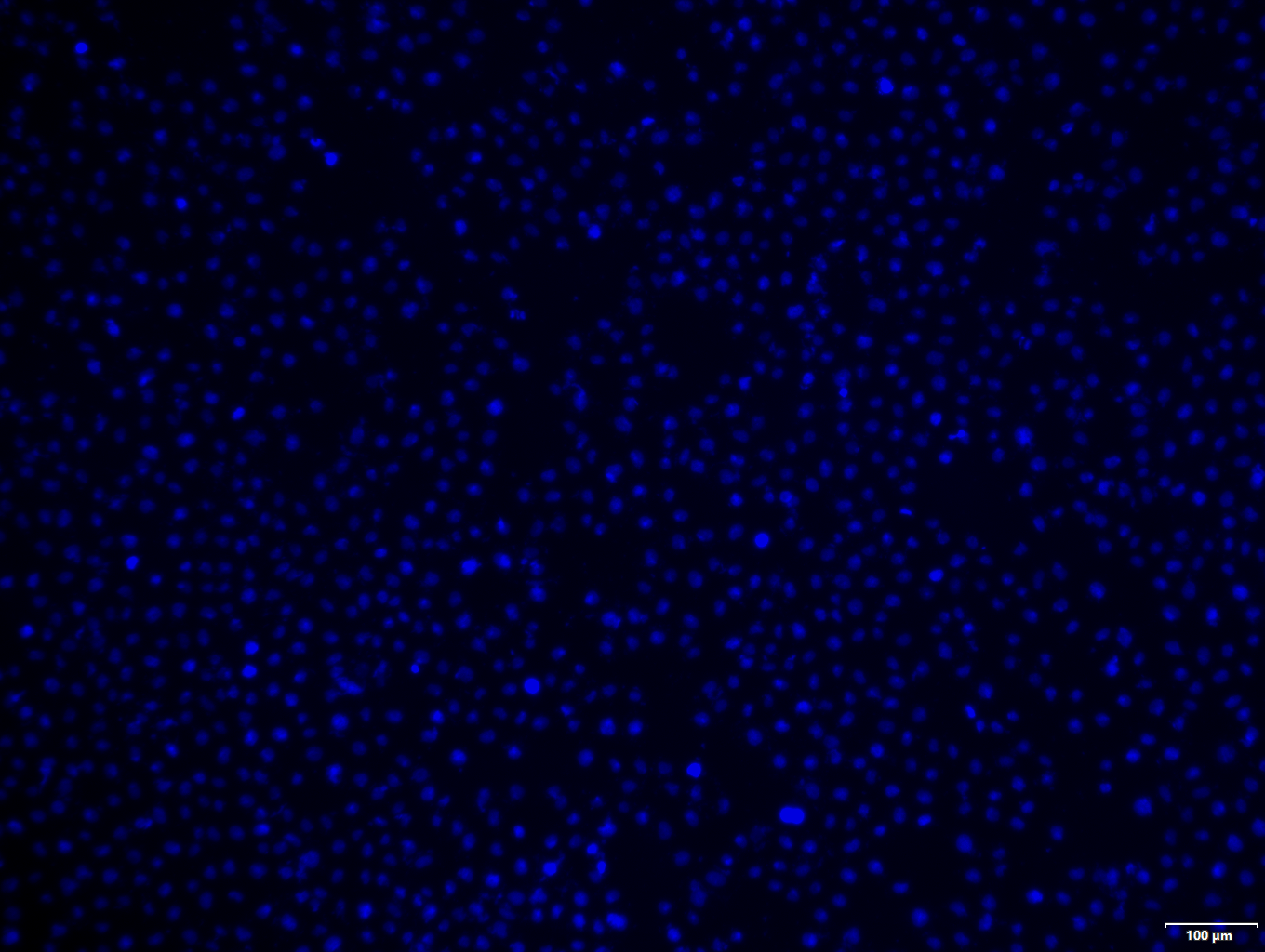

Supplement: Supplementary file 3 — Supplementary Information 3. [file 41598_2024_55043_MOESM3_ESM.zip › Supplementary material/ROS/LO2/1000-1.tif]

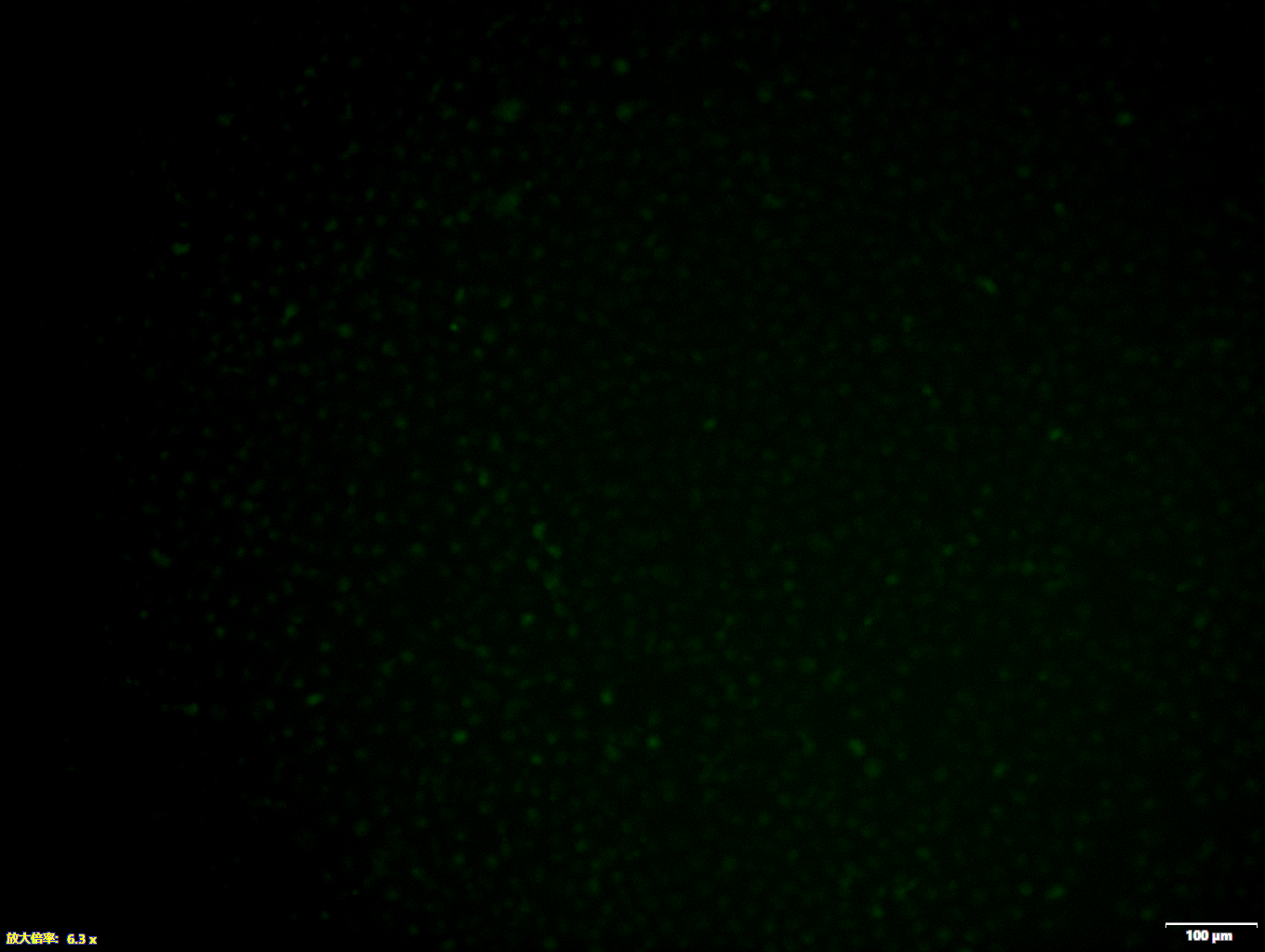

Supplement: Supplementary file 3 — Supplementary Information 3. [file 41598_2024_55043_MOESM3_ESM.zip › Supplementary material/ROS/LO2/con4.tif]

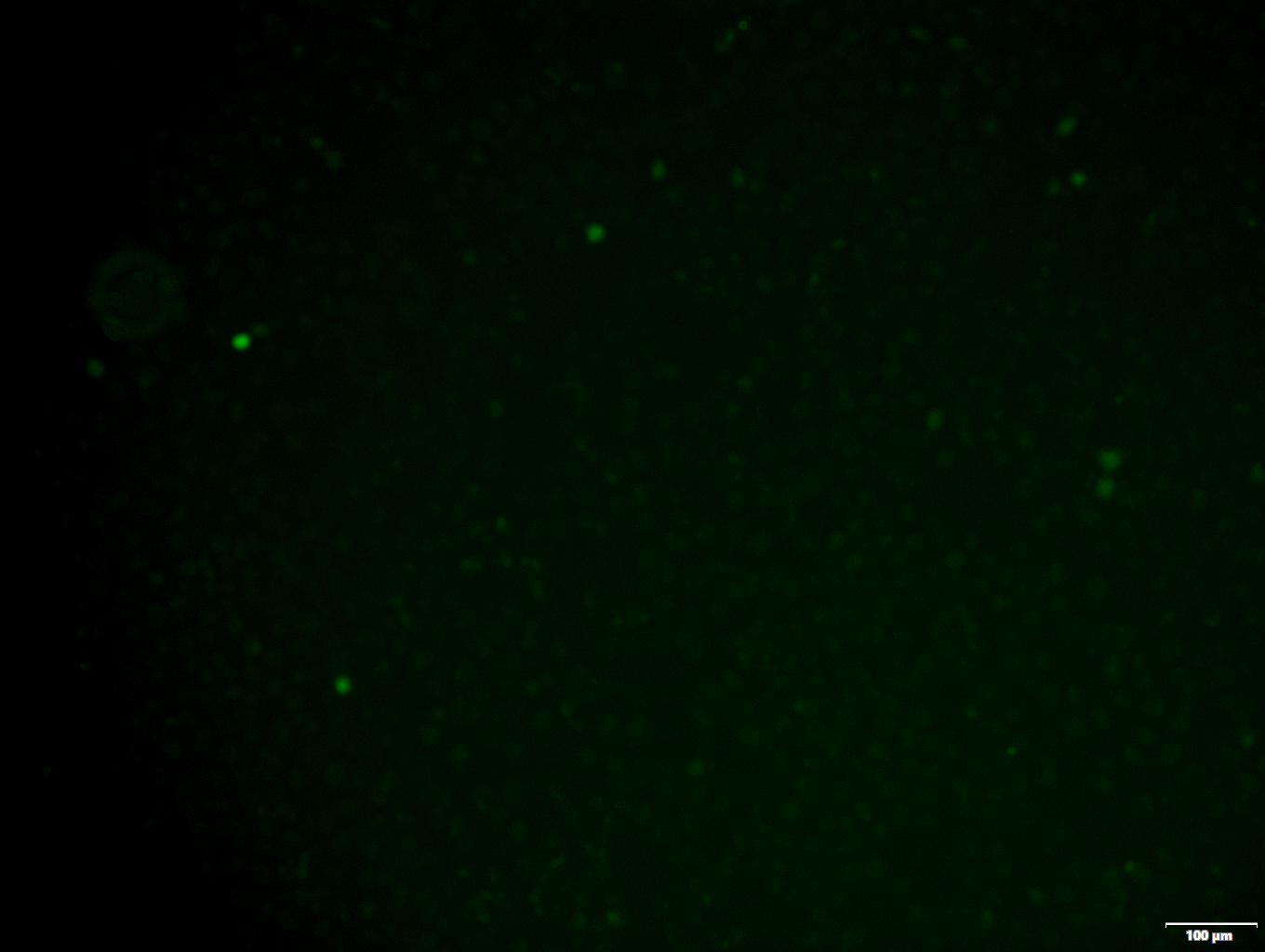

Supplement: Supplementary file 3 — Supplementary Information 3. [file 41598_2024_55043_MOESM3_ESM.zip › Supplementary material/ROS/LO2/1000-2.tif]

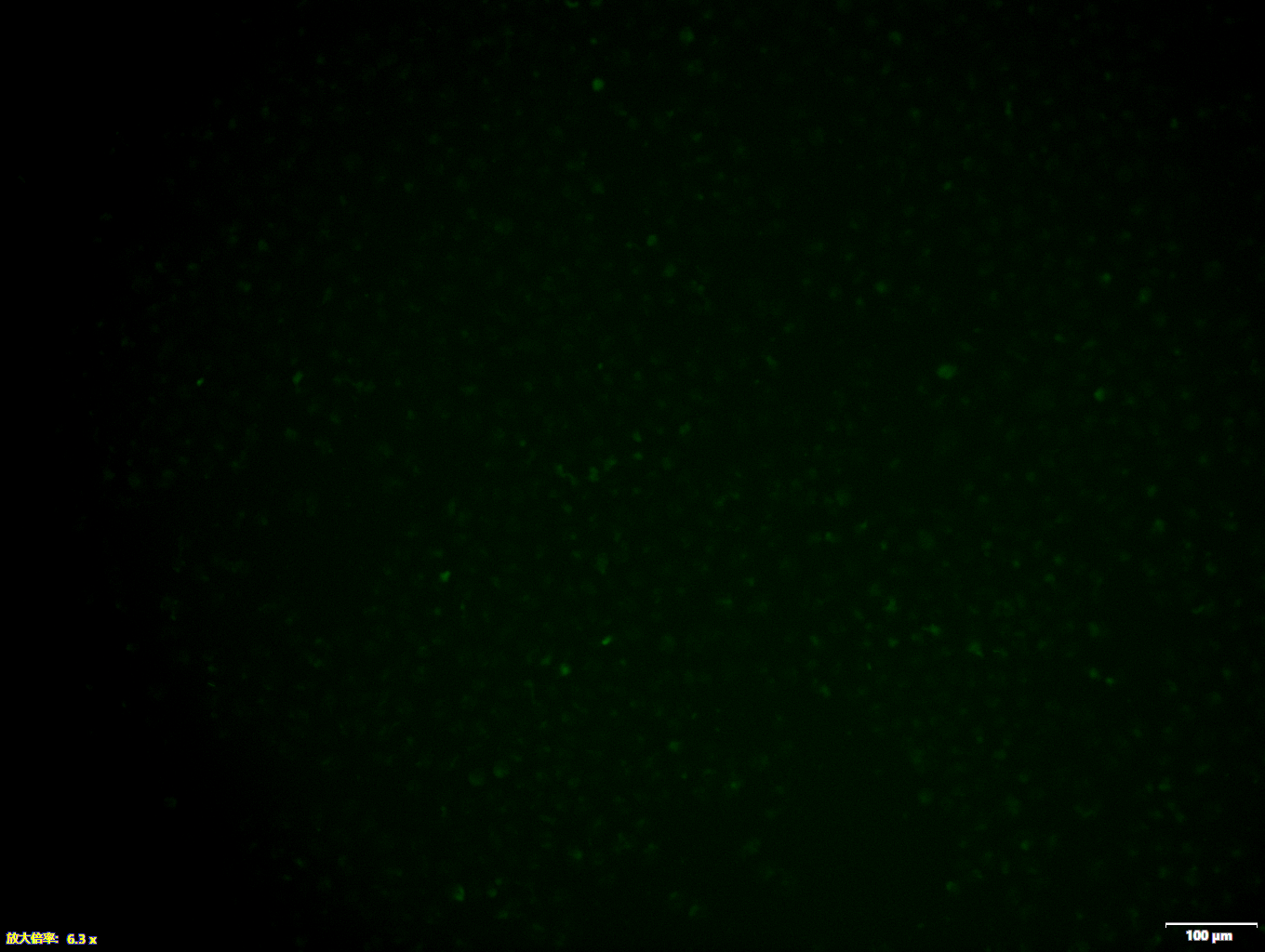

Supplement: Supplementary file 3 — Supplementary Information 3. [file 41598_2024_55043_MOESM3_ESM.zip › Supplementary material/ROS/LO2/con1.tif]

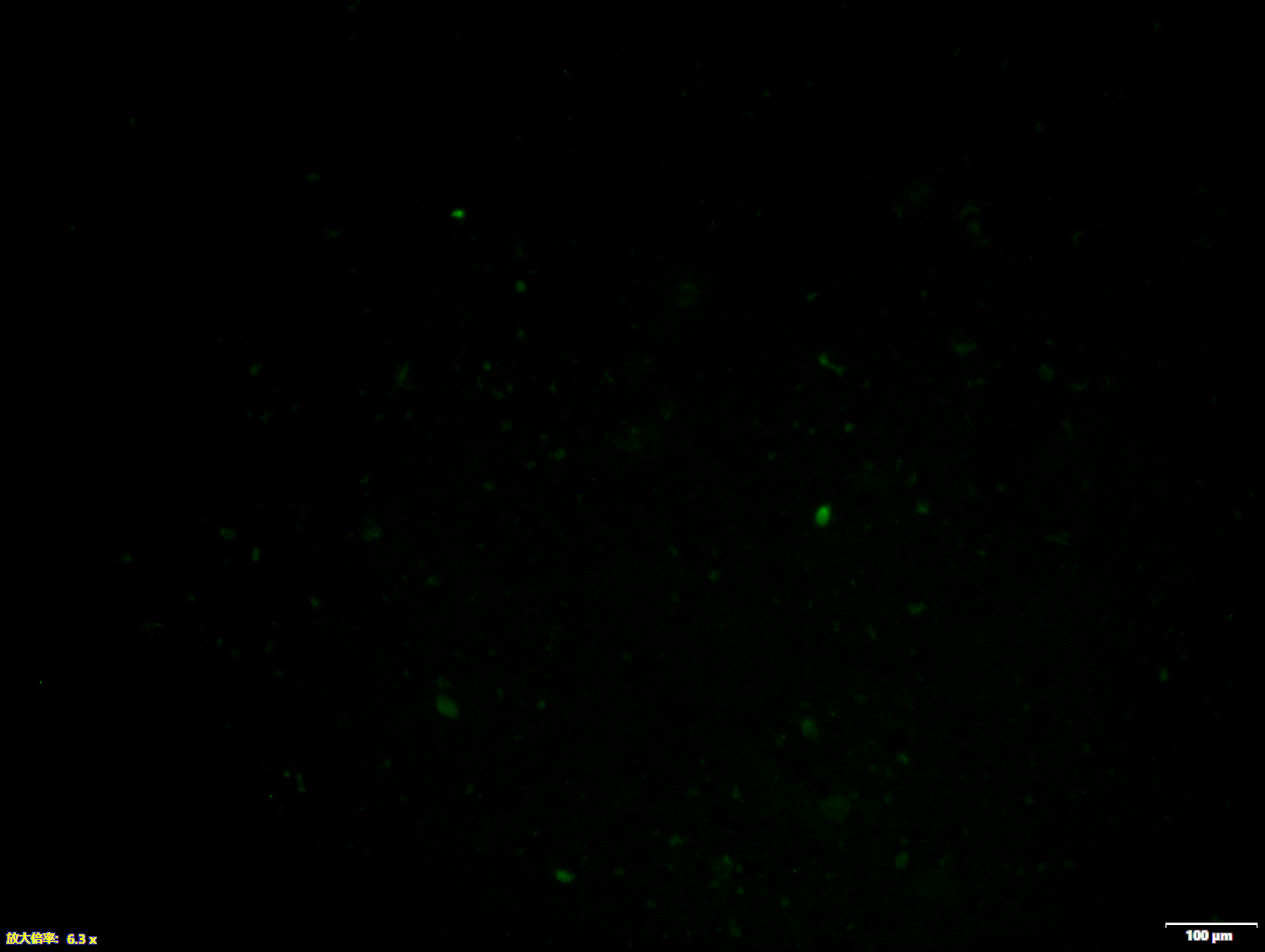

Supplement: Supplementary file 3 — Supplementary Information 3. [file 41598_2024_55043_MOESM3_ESM.zip › Supplementary material/ROS/LO2/200-2.tif]

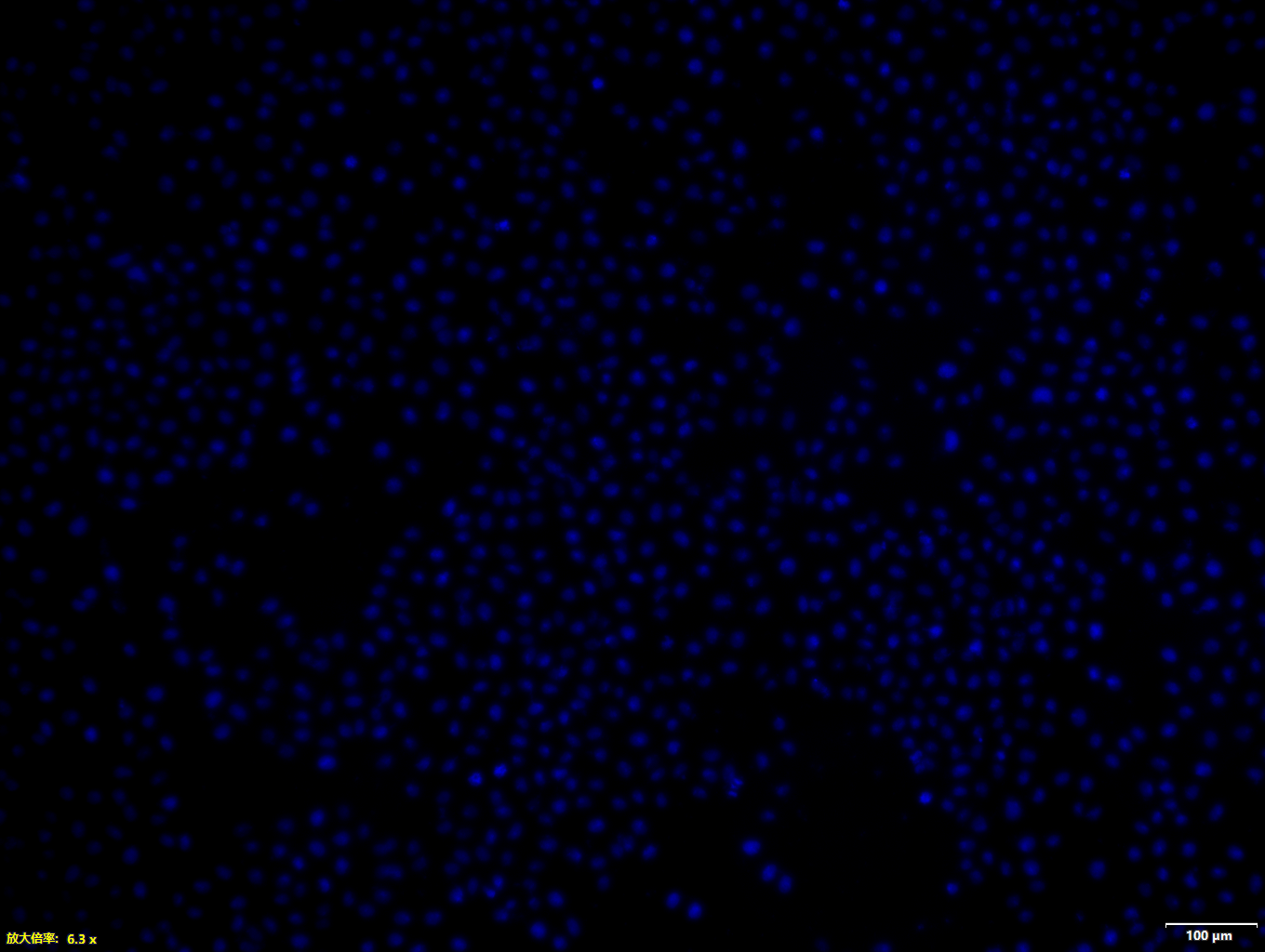

Supplement: Supplementary file 3 — Supplementary Information 3. [file 41598_2024_55043_MOESM3_ESM.zip › Supplementary material/ROS/LO2/con2.tif]

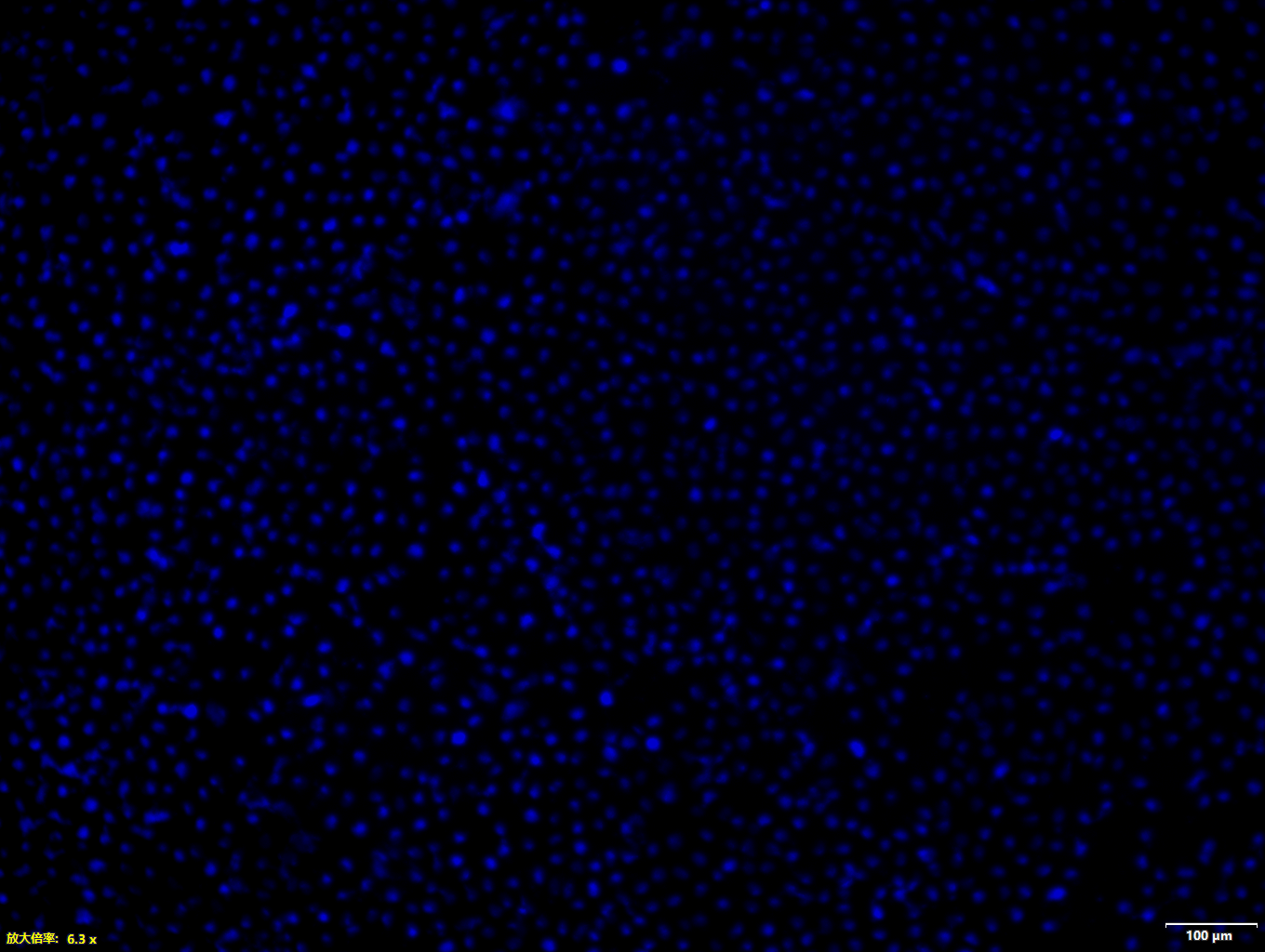

Supplement: Supplementary file 3 — Supplementary Information 3. [file 41598_2024_55043_MOESM3_ESM.zip › Supplementary material/ROS/LO2/con3.tif]

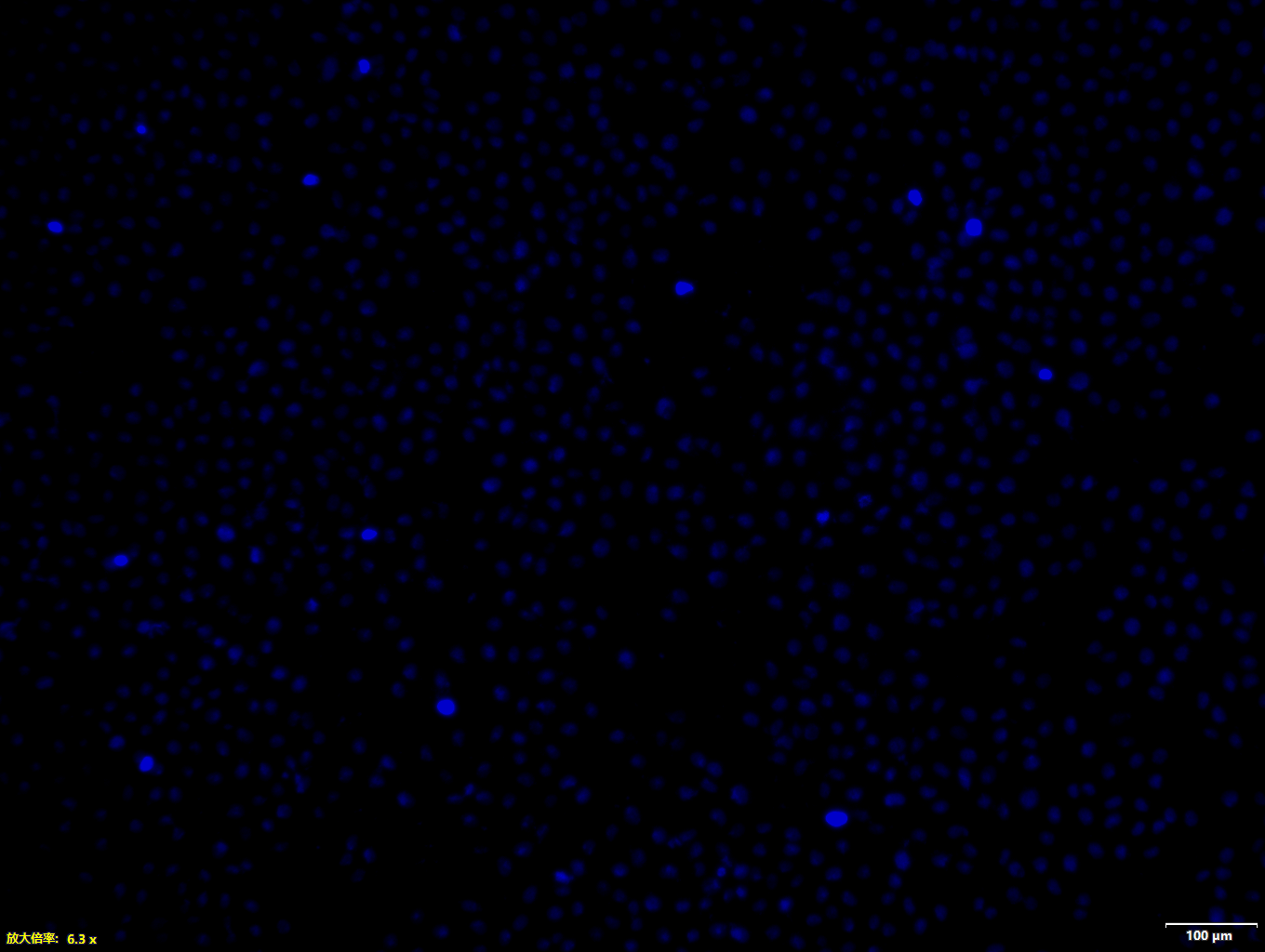

Supplement: Supplementary file 3 — Supplementary Information 3. [file 41598_2024_55043_MOESM3_ESM.zip › Supplementary material/ROS/LO2/200-1.tif]

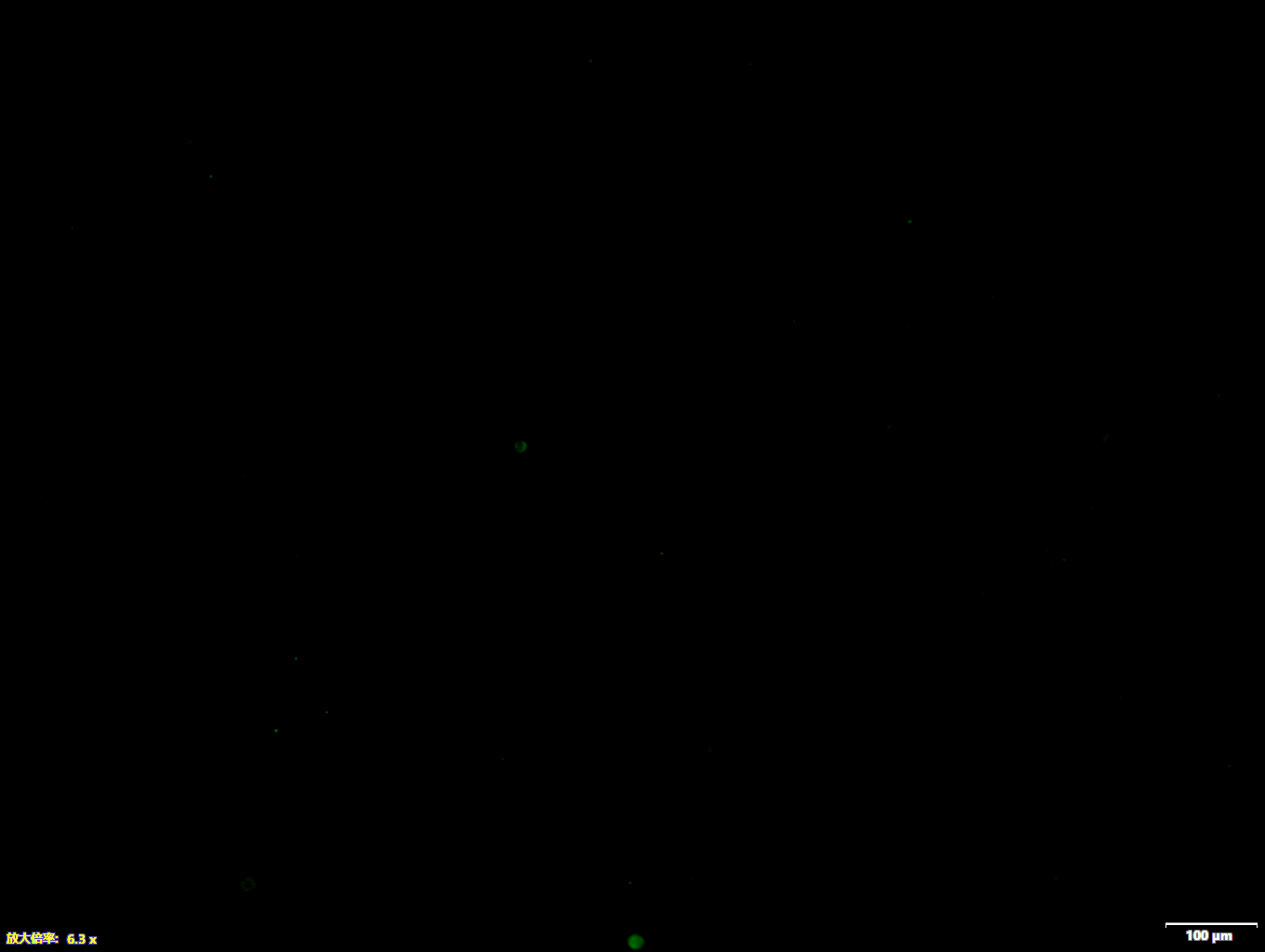

Supplement: Supplementary file 3 — Supplementary Information 3. [file 41598_2024_55043_MOESM3_ESM.zip › Supplementary material/ROS/LO2/400-2.tif]

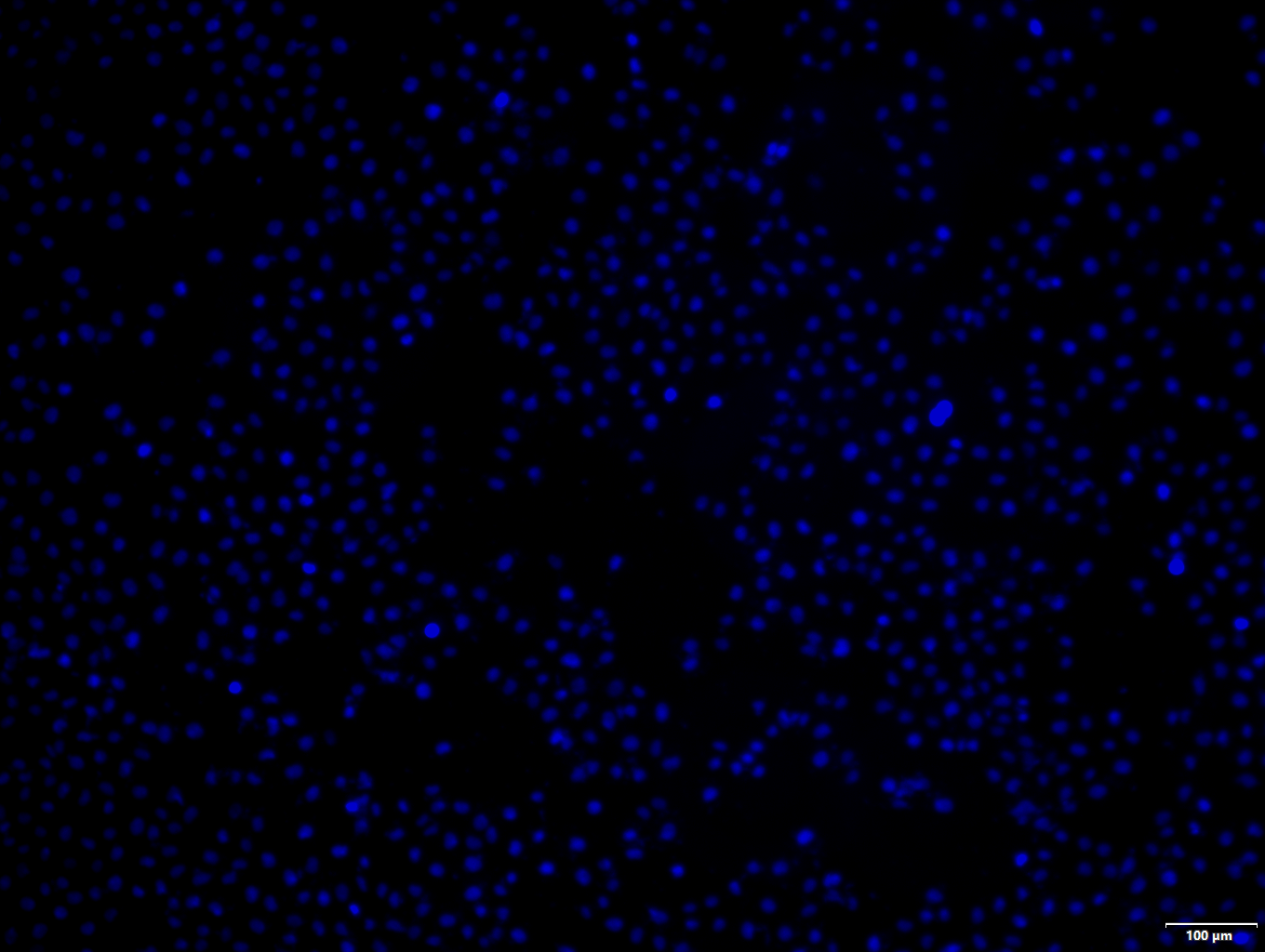

Supplement: Supplementary file 3 — Supplementary Information 3. [file 41598_2024_55043_MOESM3_ESM.zip › Supplementary material/ROS/LO2/8800-1.tif]

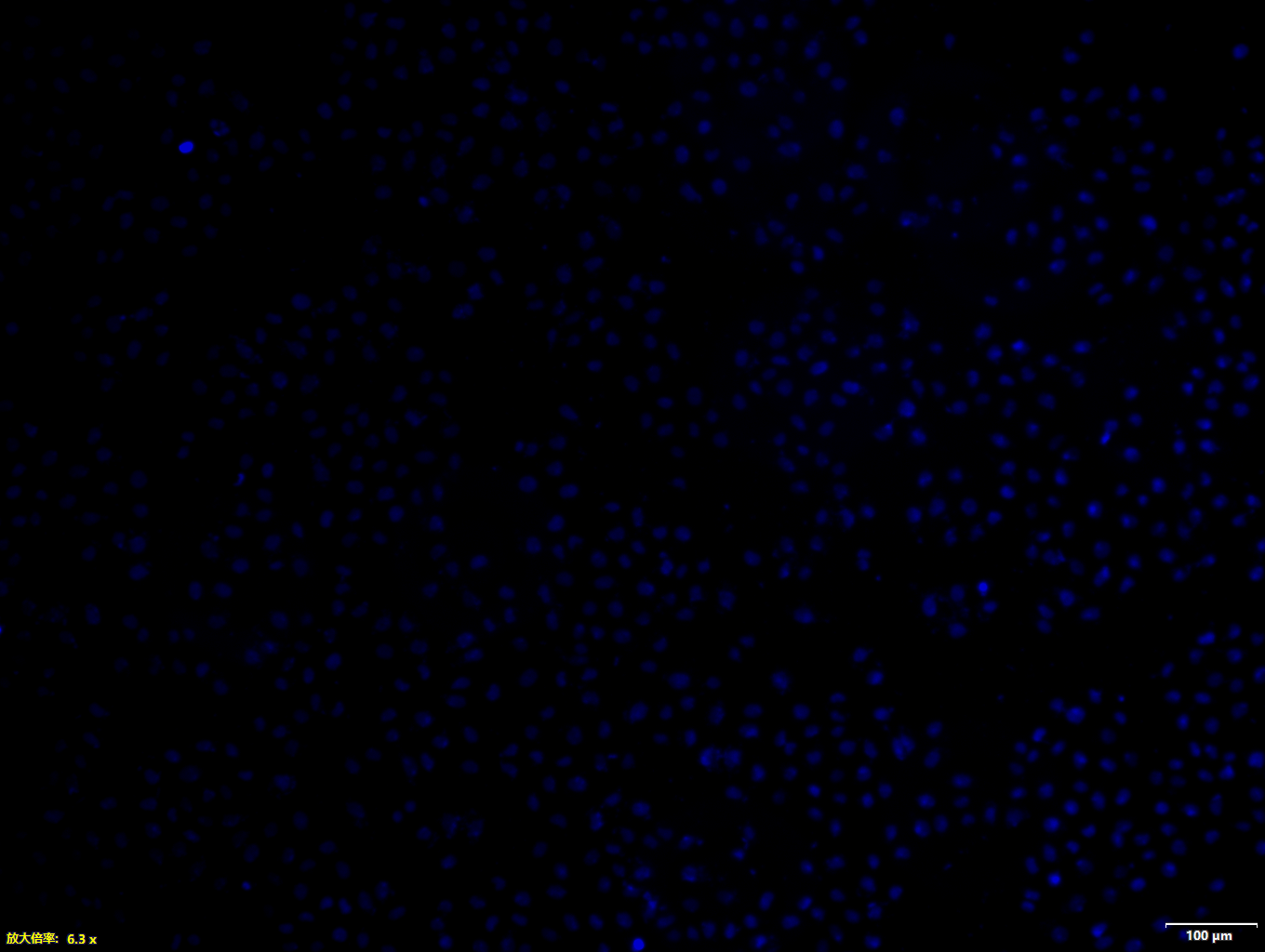

Supplement: Supplementary file 3 — Supplementary Information 3. [file 41598_2024_55043_MOESM3_ESM.zip › Supplementary material/ROS/LO2/400-1.tif]

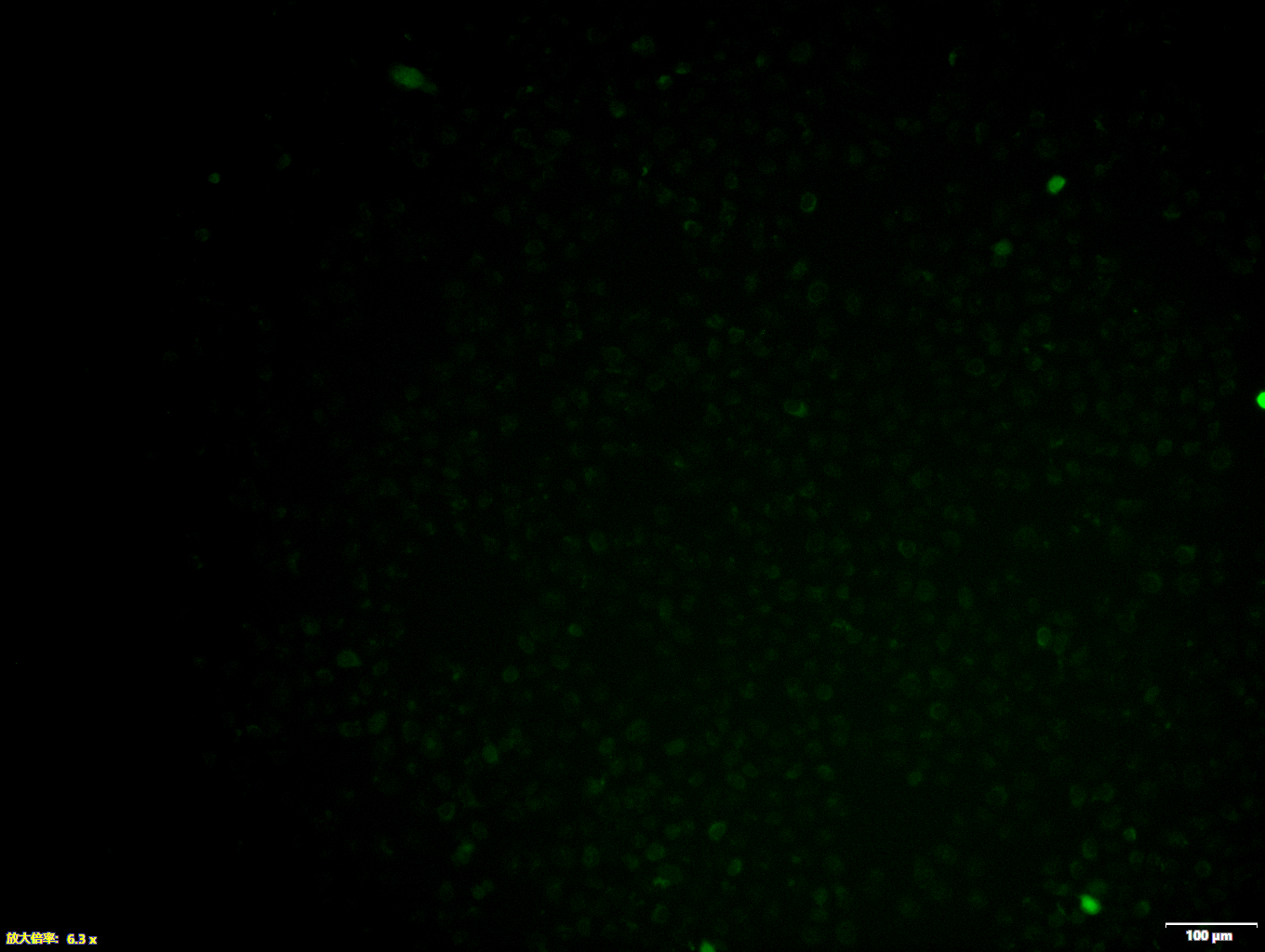

Supplement: Supplementary file 3 — Supplementary Information 3. [file 41598_2024_55043_MOESM3_ESM.zip › Supplementary material/ROS/LO2/600-2.tif]

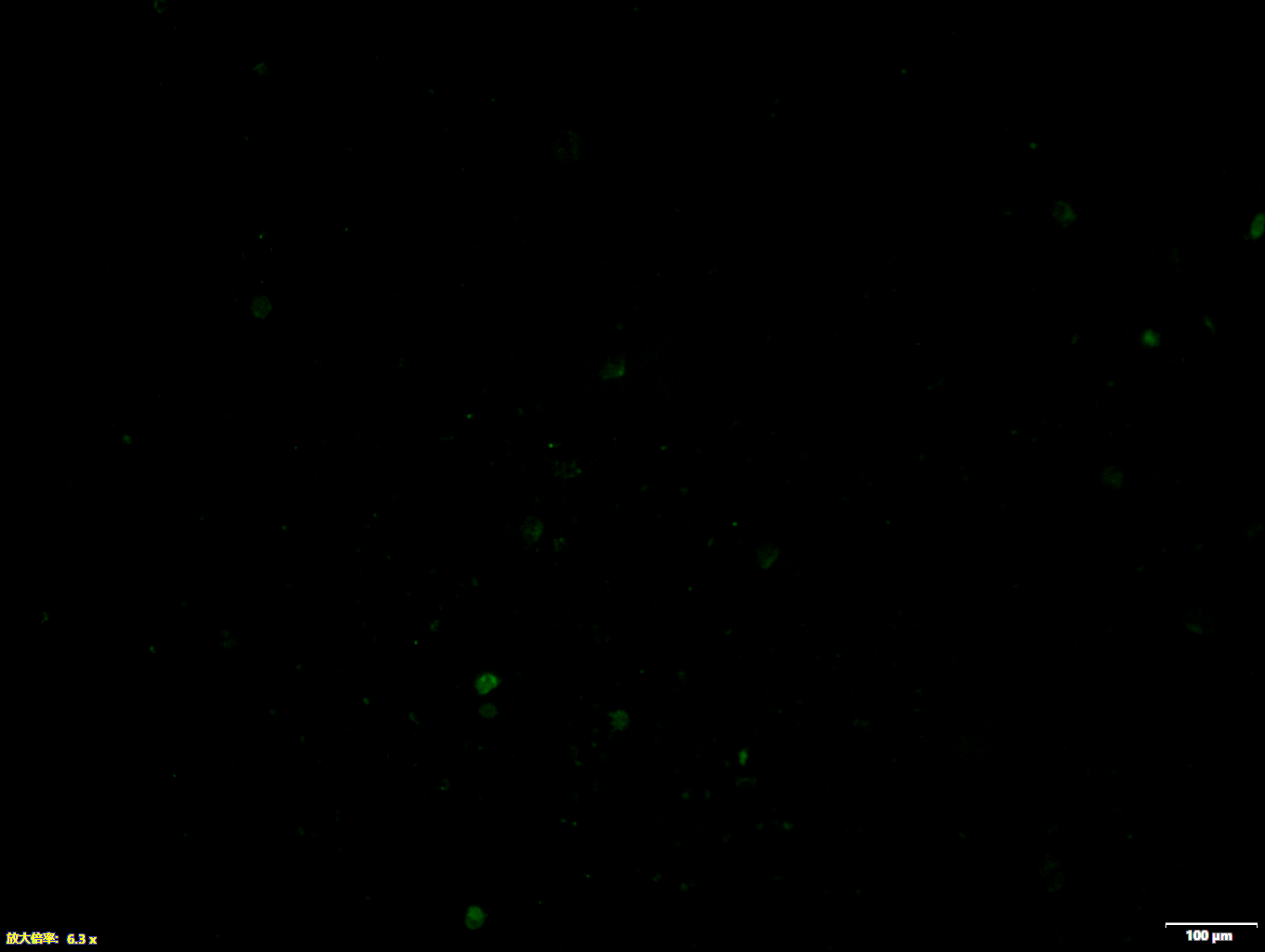

Supplement: Supplementary file 3 — Supplementary Information 3. [file 41598_2024_55043_MOESM3_ESM.zip › Supplementary material/ROS/LO2/+-2.tif]

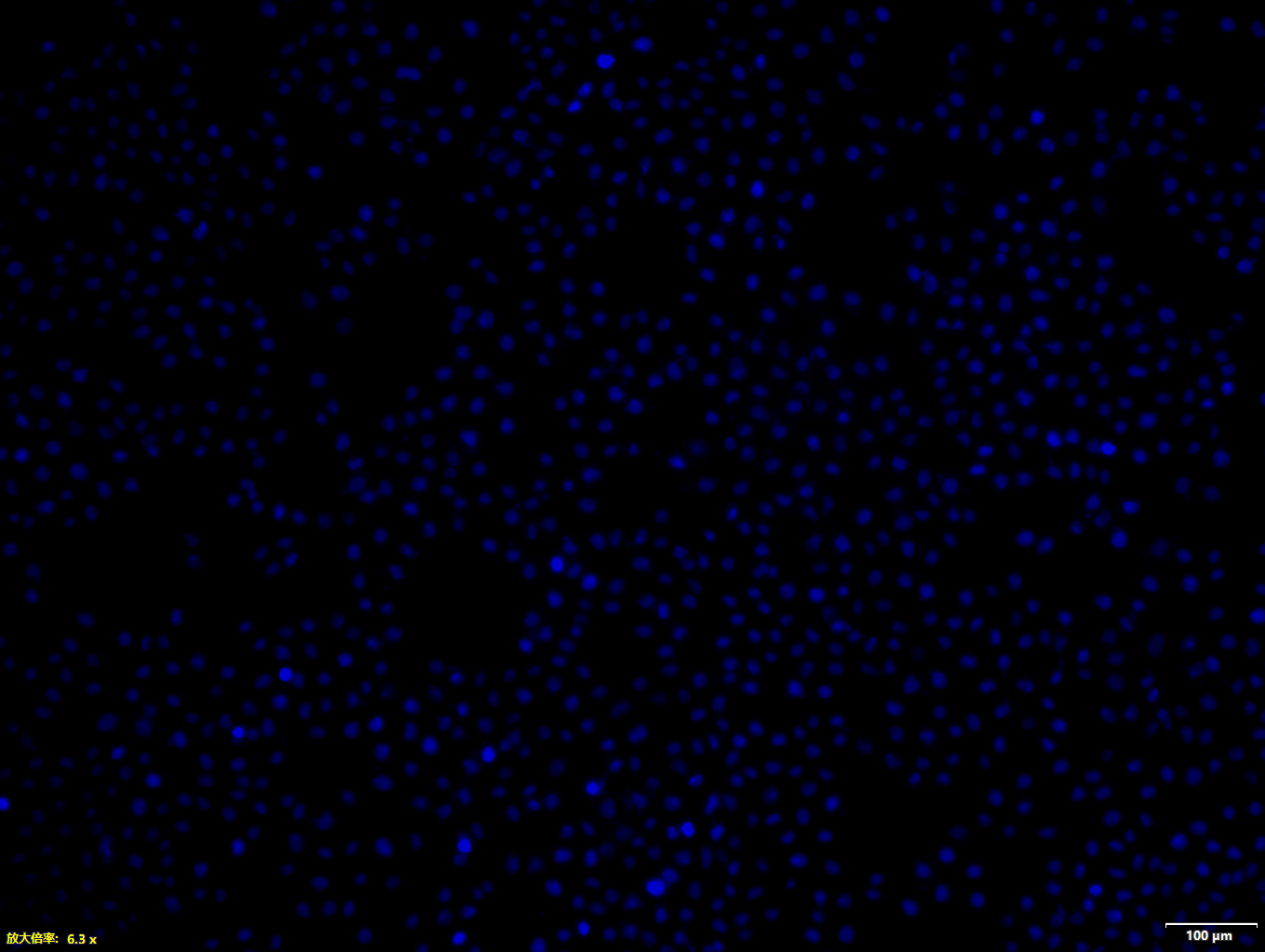

Supplement: Supplementary file 3 — Supplementary Information 3. [file 41598_2024_55043_MOESM3_ESM.zip › Supplementary material/ROS/LO2/600-1.tif]

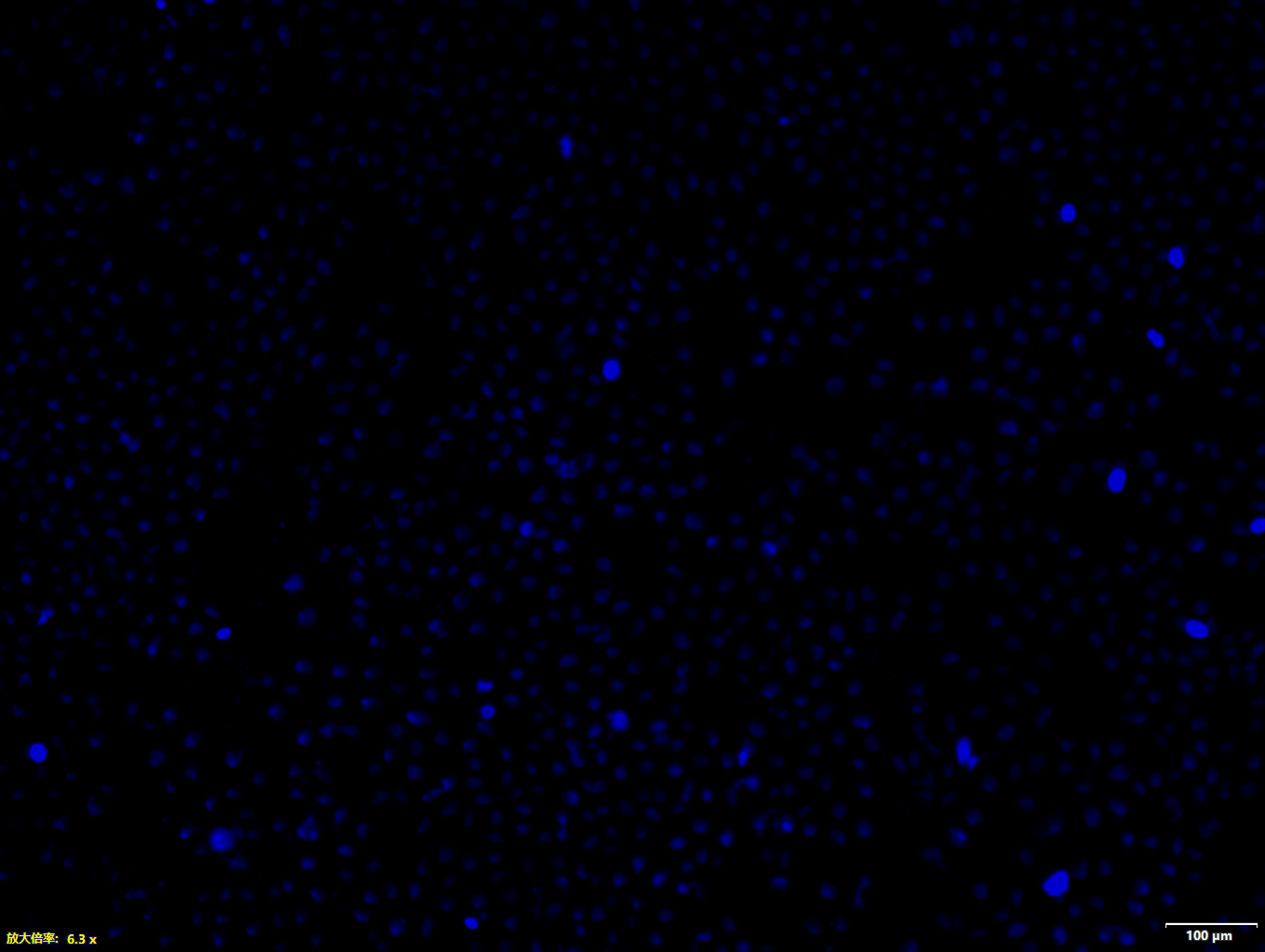

Supplement: Supplementary file 3 — Supplementary Information 3. [file 41598_2024_55043_MOESM3_ESM.zip › Supplementary material/ROS/LO2/+-1.tif]

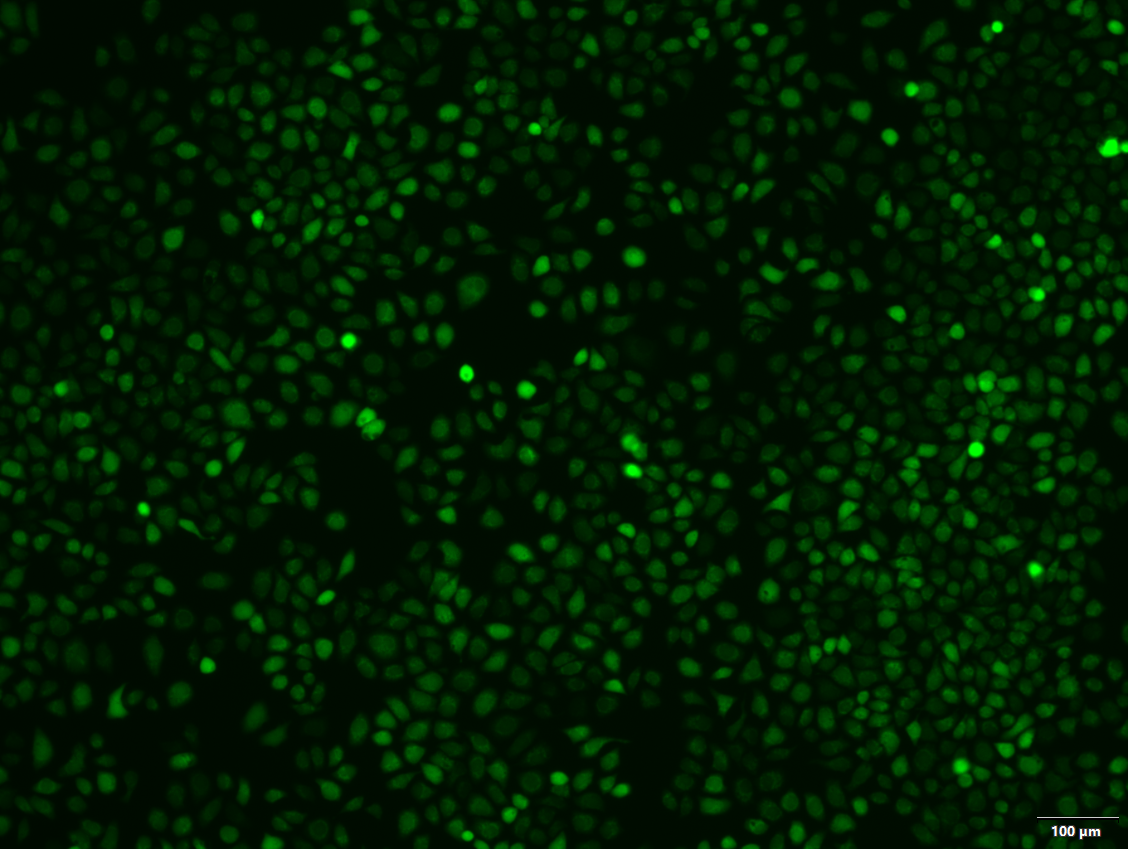

Supplement: Supplementary file 3 — Supplementary Information 3. [file 41598_2024_55043_MOESM3_ESM.zip › Supplementary material/ROS/20230222/200uM.tif]

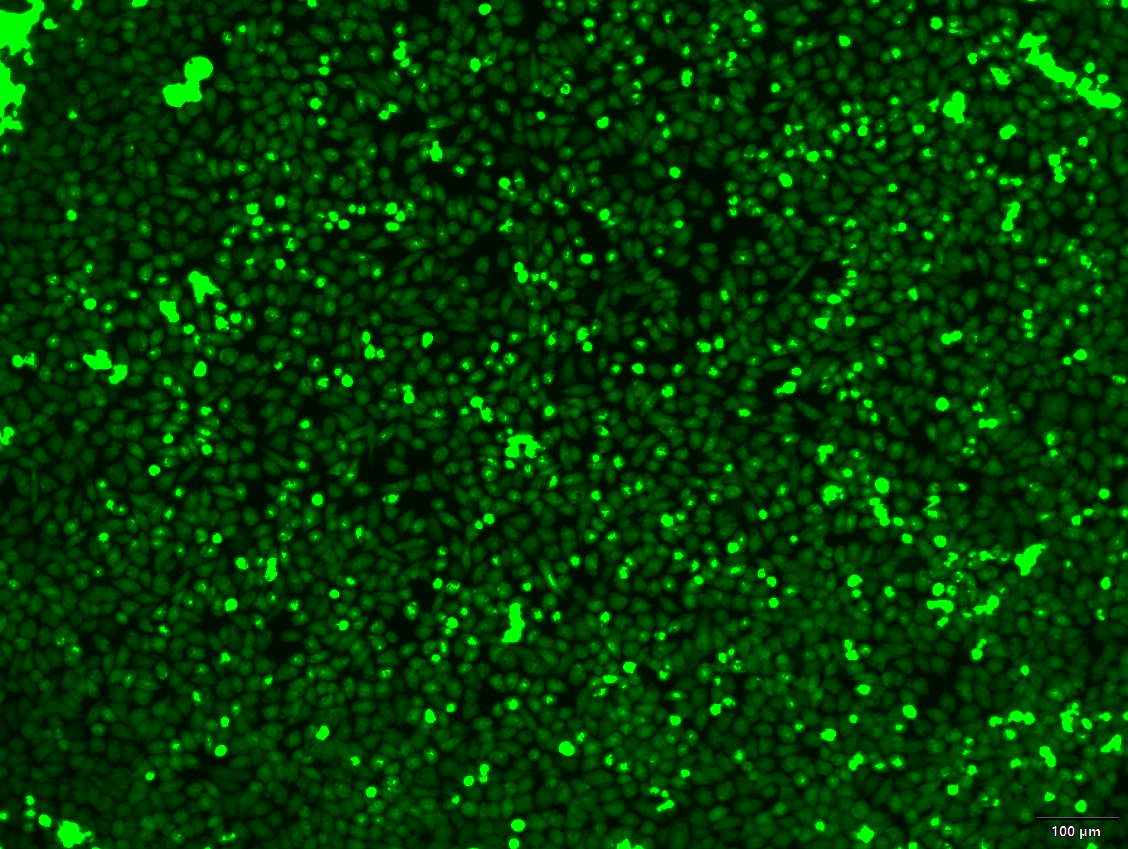

Supplement: Supplementary file 3 — Supplementary Information 3. [file 41598_2024_55043_MOESM3_ESM.zip › Supplementary material/ROS/20230222/+_01.tif]

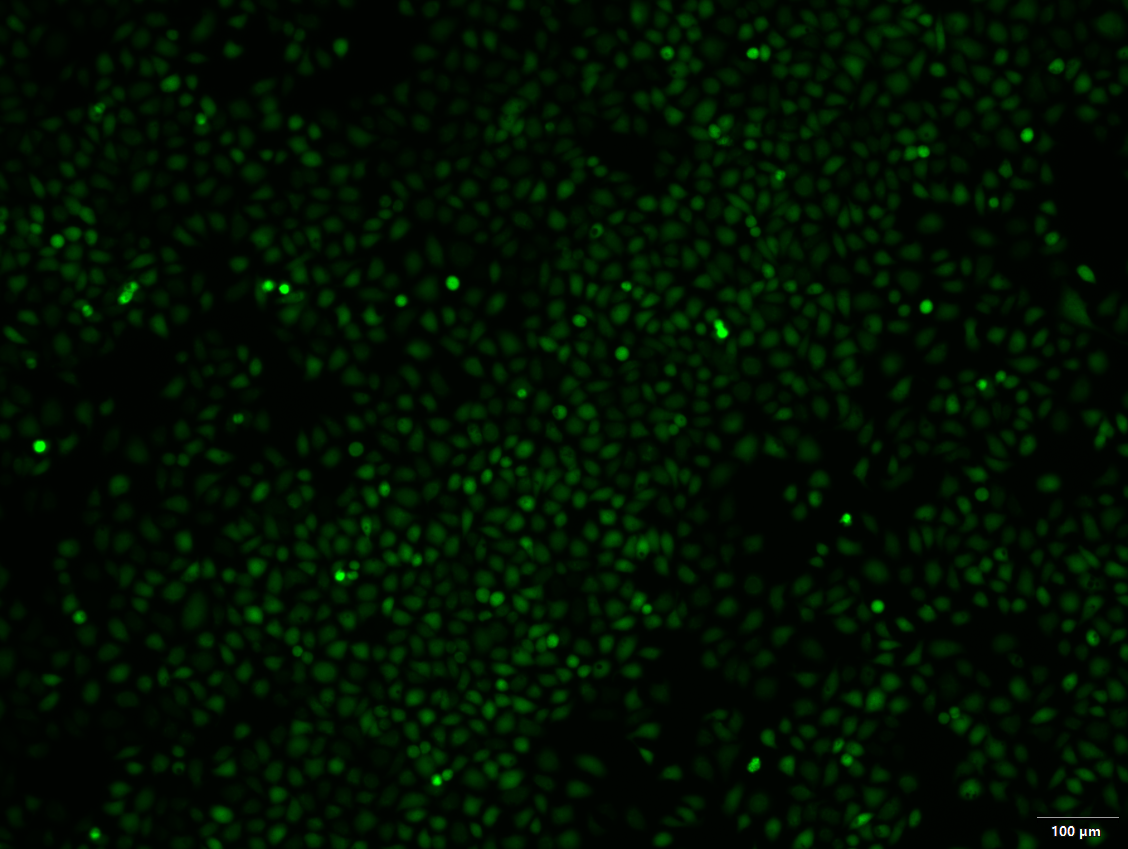

Supplement: Supplementary file 3 — Supplementary Information 3. [file 41598_2024_55043_MOESM3_ESM.zip › Supplementary material/ROS/20230222/control_01.tif]

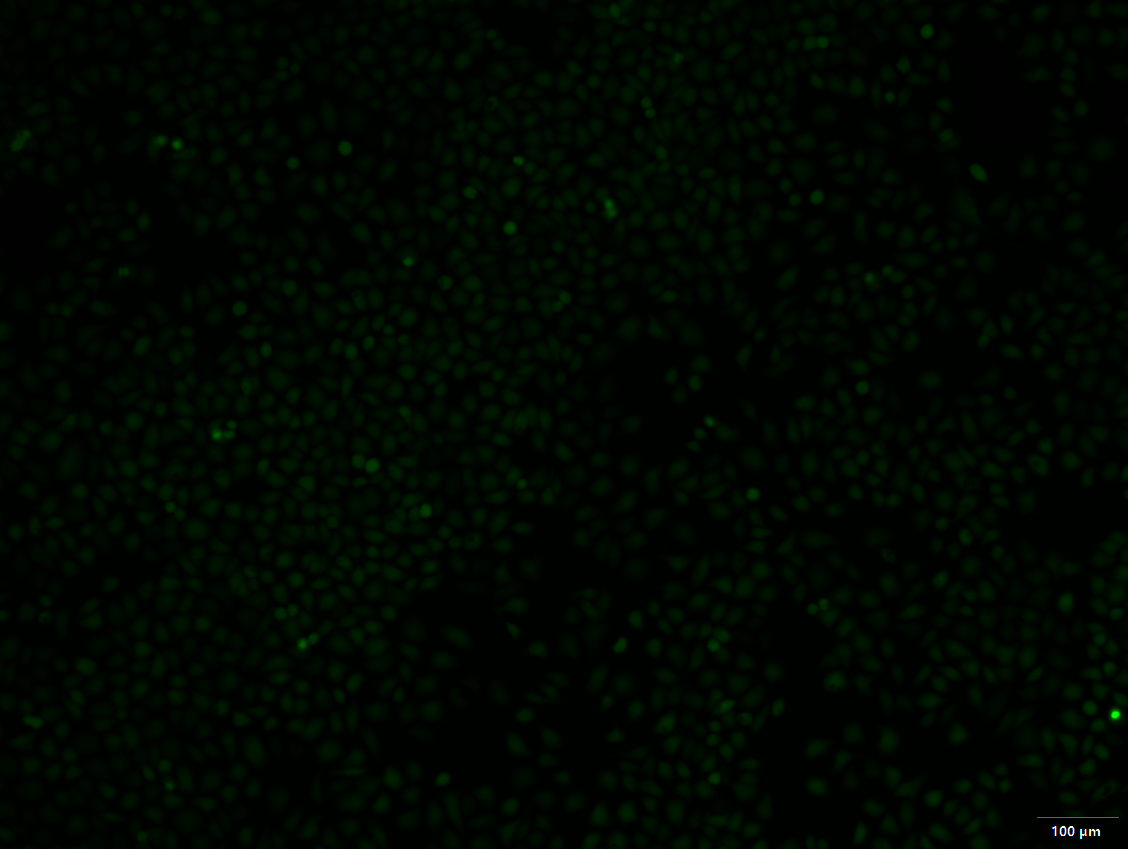

Supplement: Supplementary file 3 — Supplementary Information 3. [file 41598_2024_55043_MOESM3_ESM.zip › Supplementary material/ROS/20230222/control_02.tif]

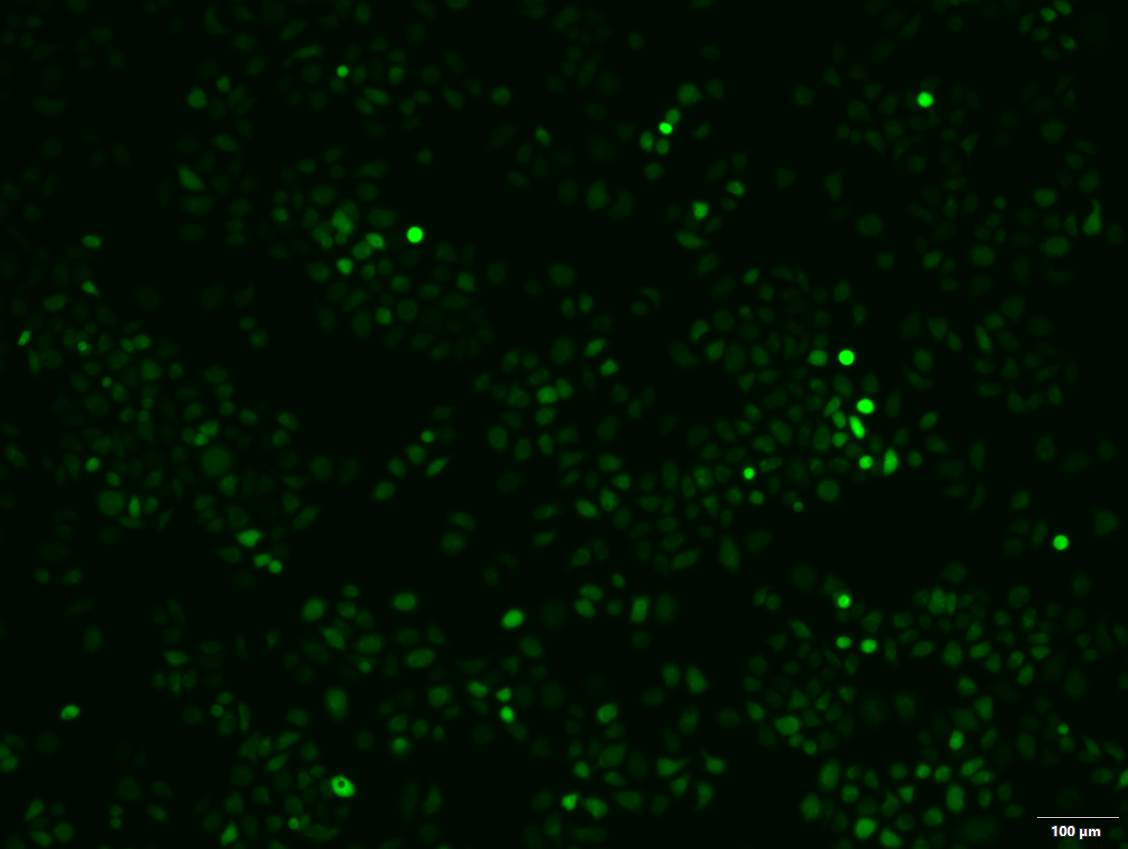

Supplement: Supplementary file 3 — Supplementary Information 3. [file 41598_2024_55043_MOESM3_ESM.zip › Supplementary material/ROS/20230222/100uM2.tif]

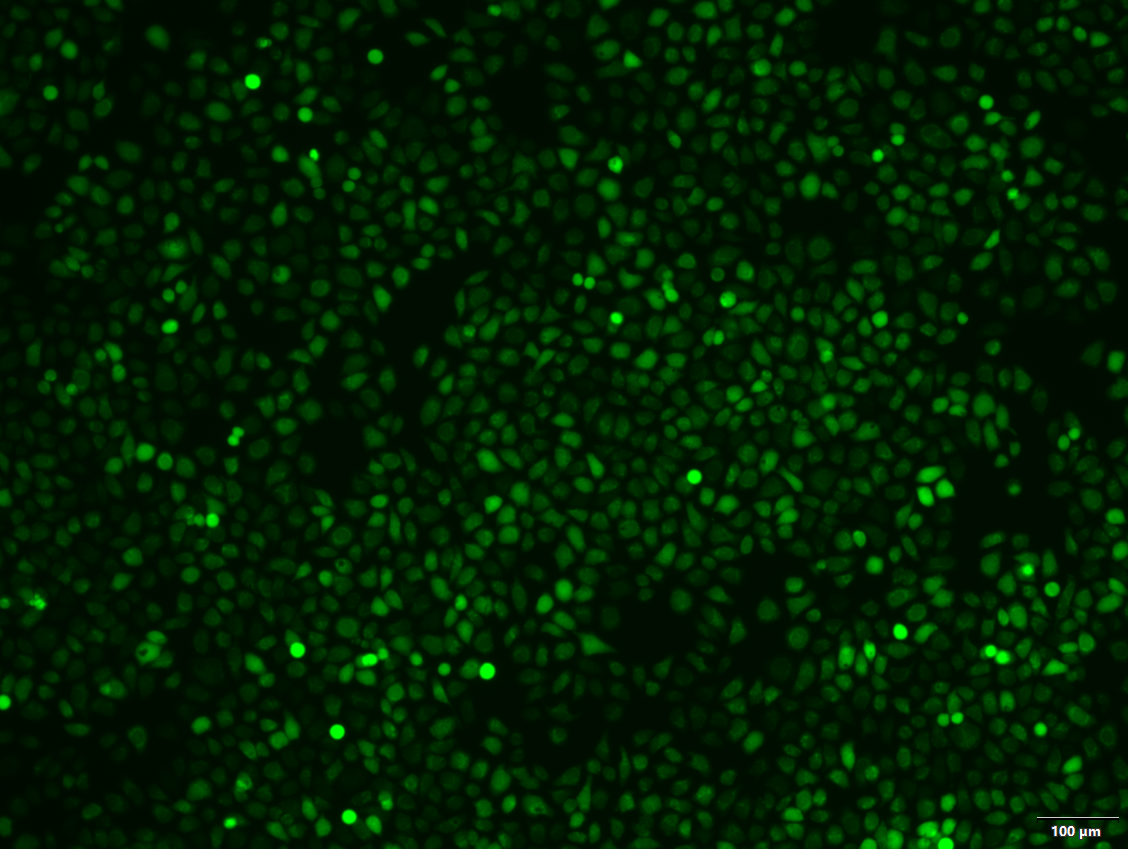

Supplement: Supplementary file 3 — Supplementary Information 3. [file 41598_2024_55043_MOESM3_ESM.zip › Supplementary material/ROS/20230222/400uM.tif]

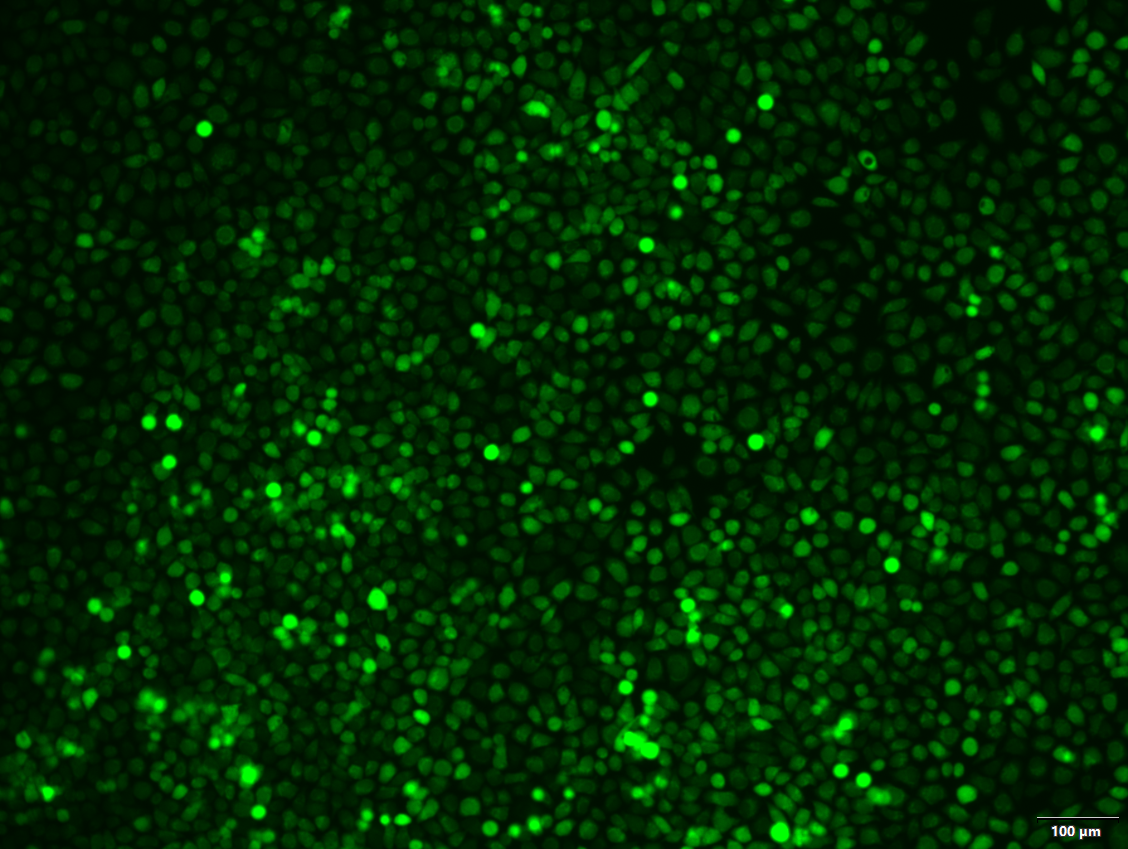

Supplement: Supplementary file 3 — Supplementary Information 3. [file 41598_2024_55043_MOESM3_ESM.zip › Supplementary material/ROS/20230222/600uM.tif]

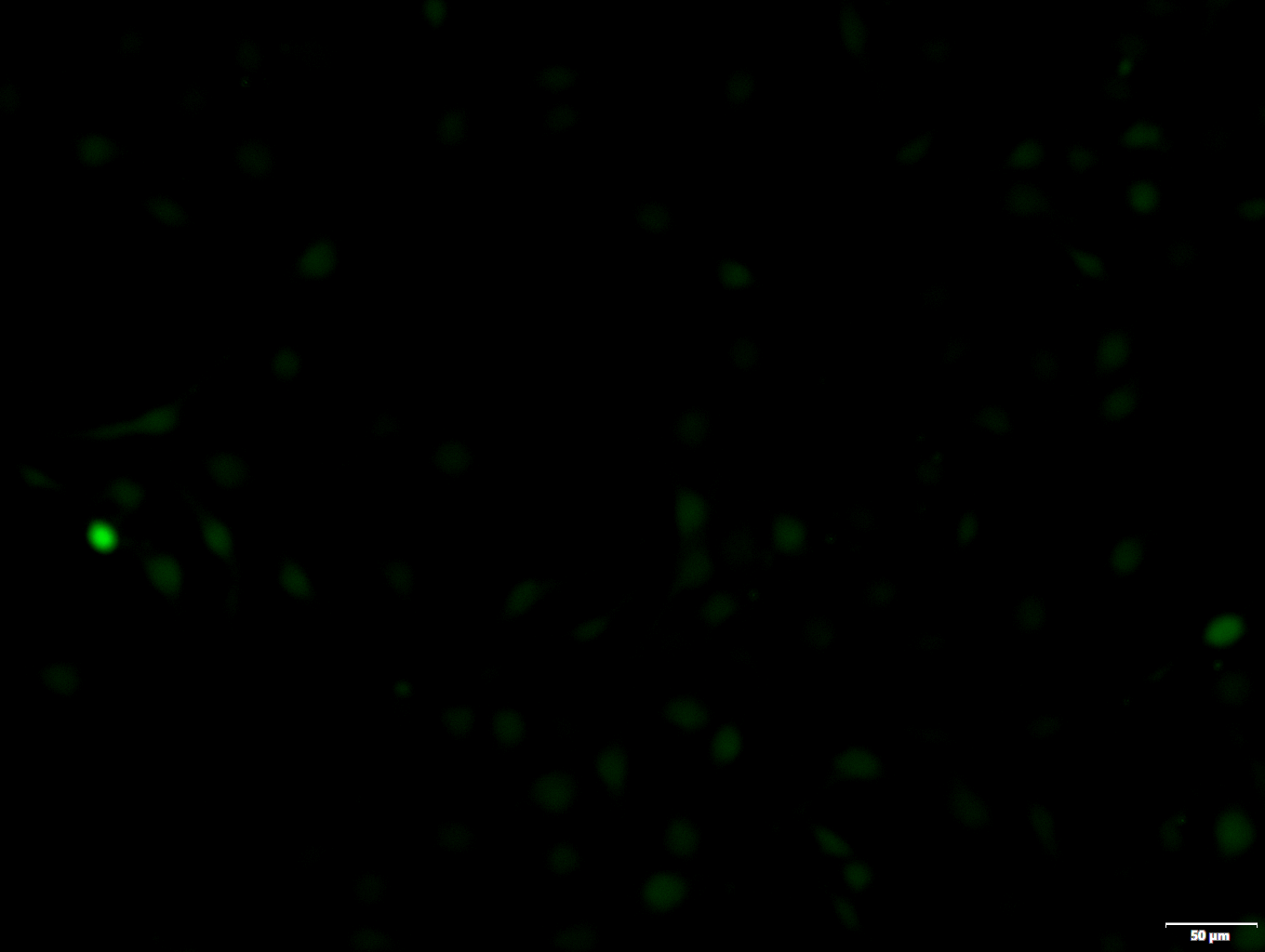

Supplement: Supplementary file 3 — Supplementary Information 3. [file 41598_2024_55043_MOESM3_ESM.zip › Supplementary material/ROS/20230416/Con4.tif]

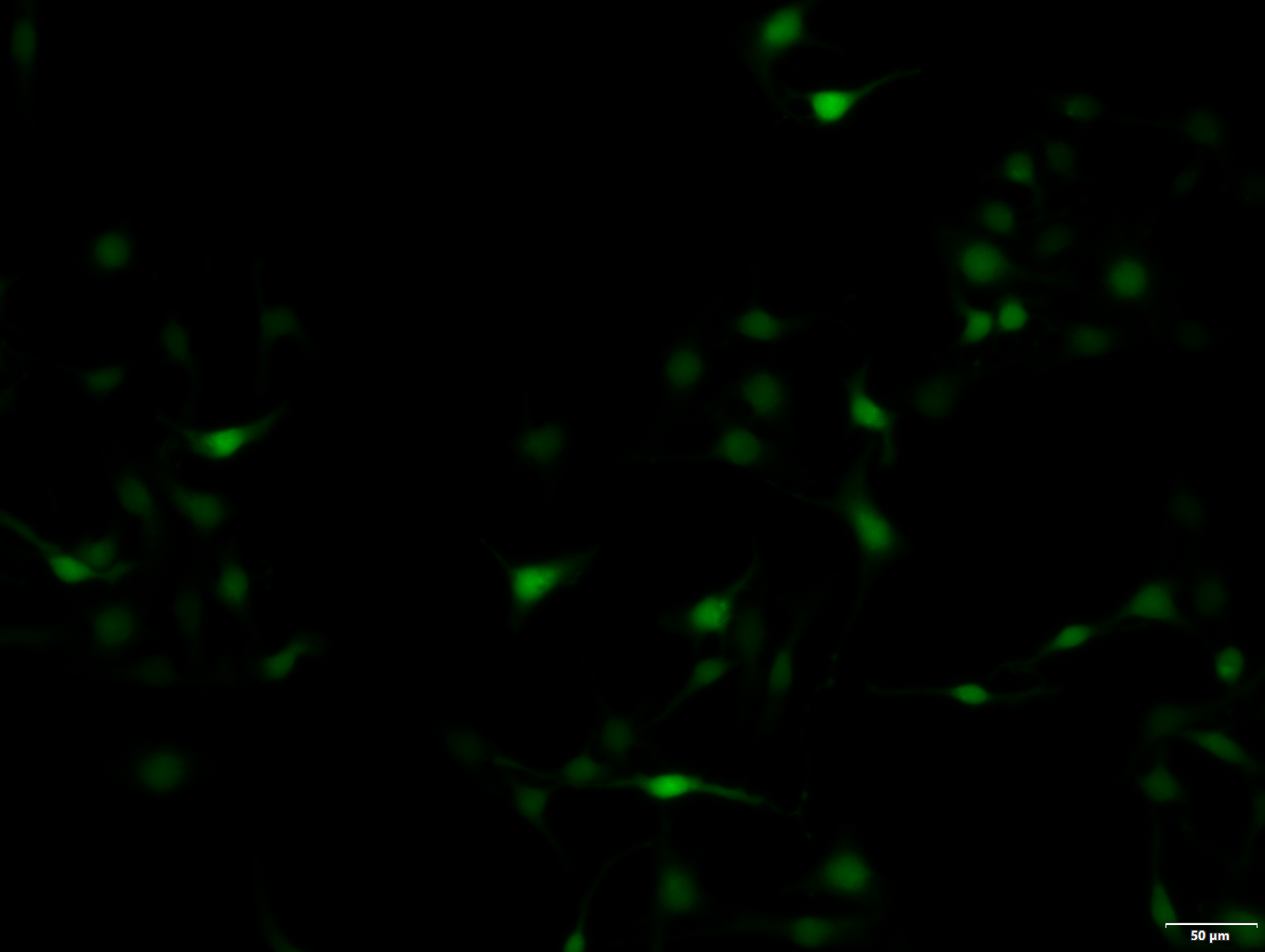

Supplement: Supplementary file 3 — Supplementary Information 3. [file 41598_2024_55043_MOESM3_ESM.zip › Supplementary material/ROS/20230416/H2O2+UDCA.tif]

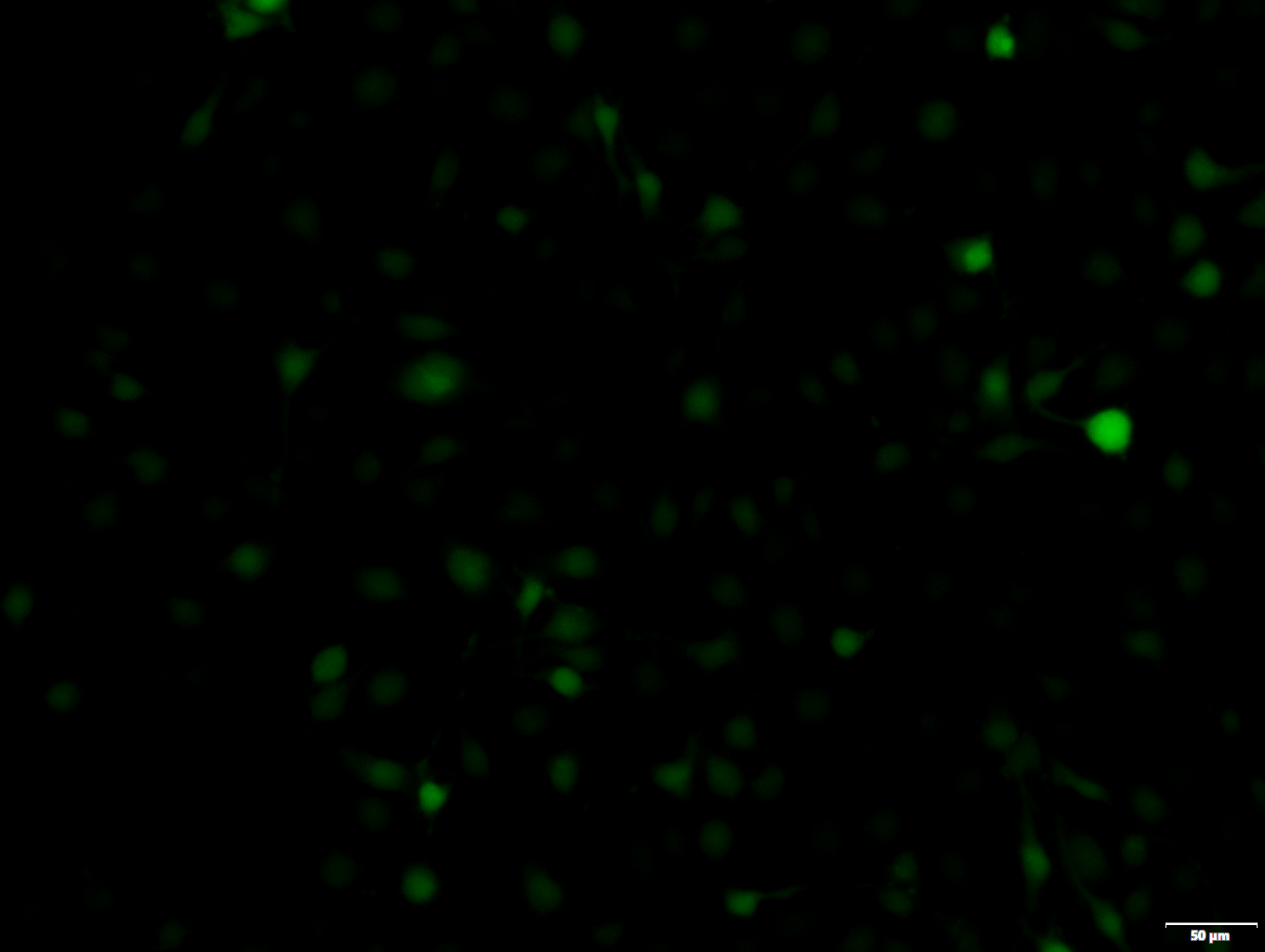

Supplement: Supplementary file 3 — Supplementary Information 3. [file 41598_2024_55043_MOESM3_ESM.zip › Supplementary material/ROS/20230416/Con2.tif]

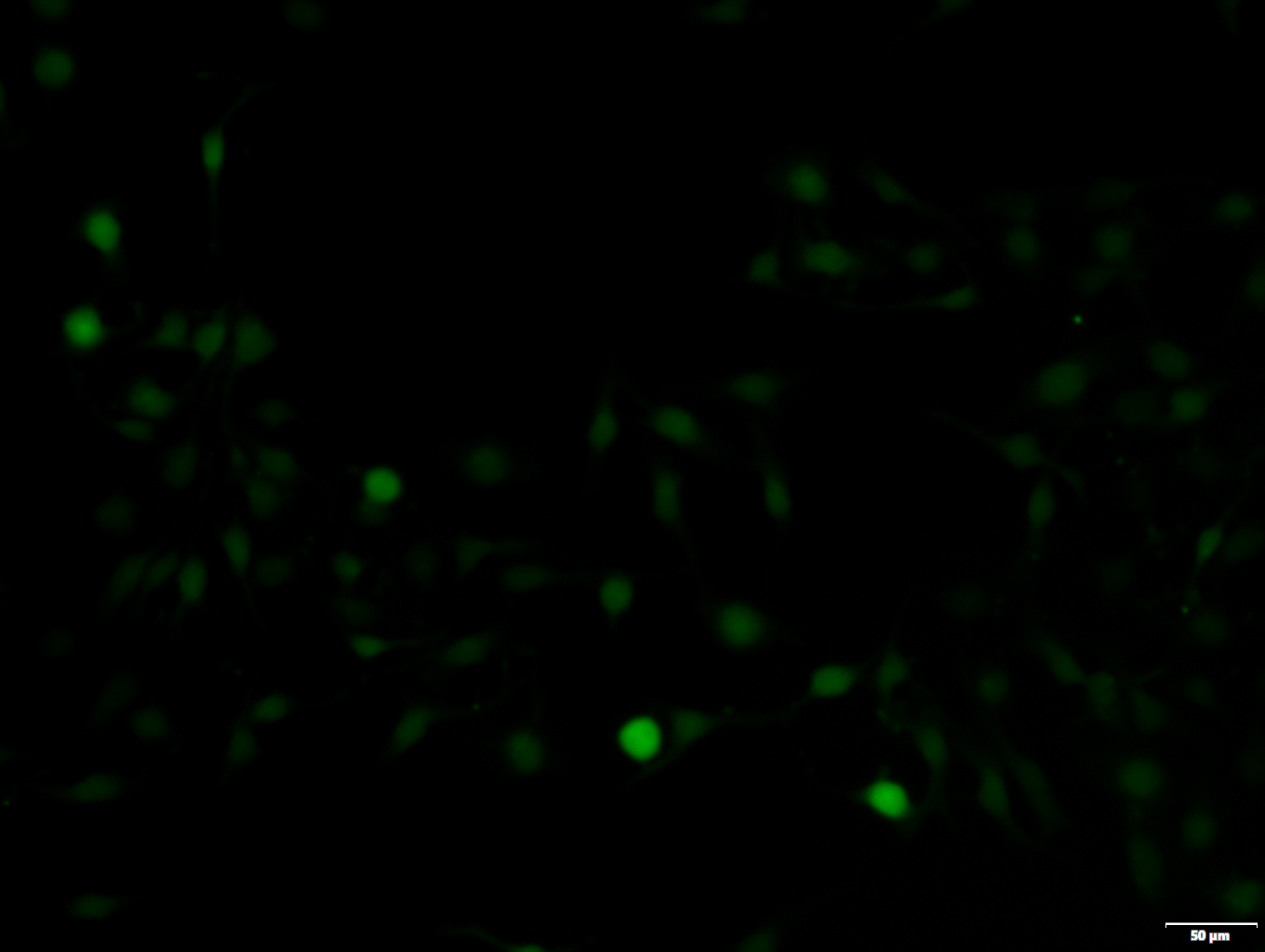

Supplement: Supplementary file 3 — Supplementary Information 3. [file 41598_2024_55043_MOESM3_ESM.zip › Supplementary material/ROS/20230416/UDCA3.tif]

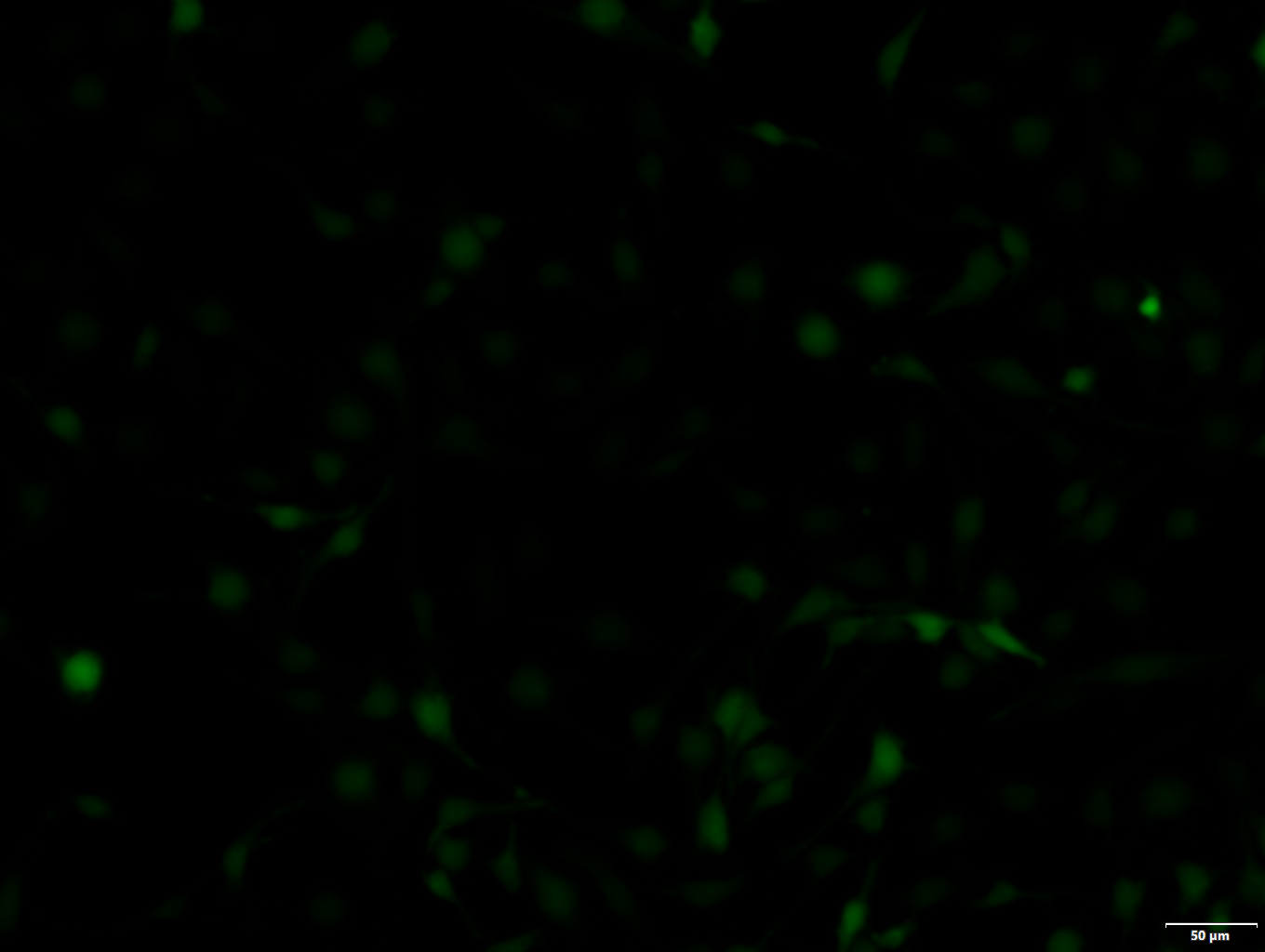

Supplement: Supplementary file 3 — Supplementary Information 3. [file 41598_2024_55043_MOESM3_ESM.zip › Supplementary material/ROS/20230416/UDCA2.tif]

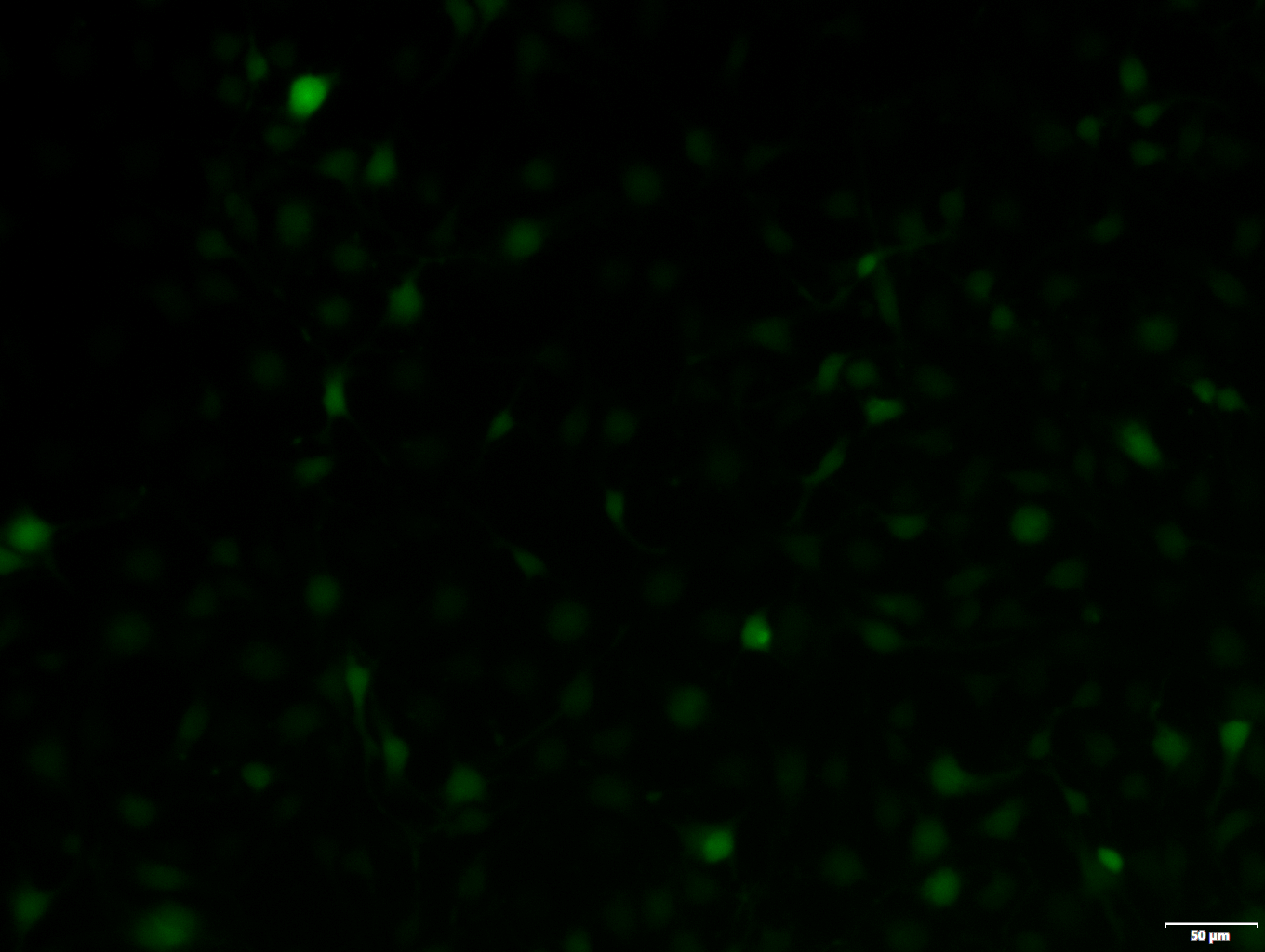

Supplement: Supplementary file 3 — Supplementary Information 3. [file 41598_2024_55043_MOESM3_ESM.zip › Supplementary material/ROS/20230416/Con3.tif]

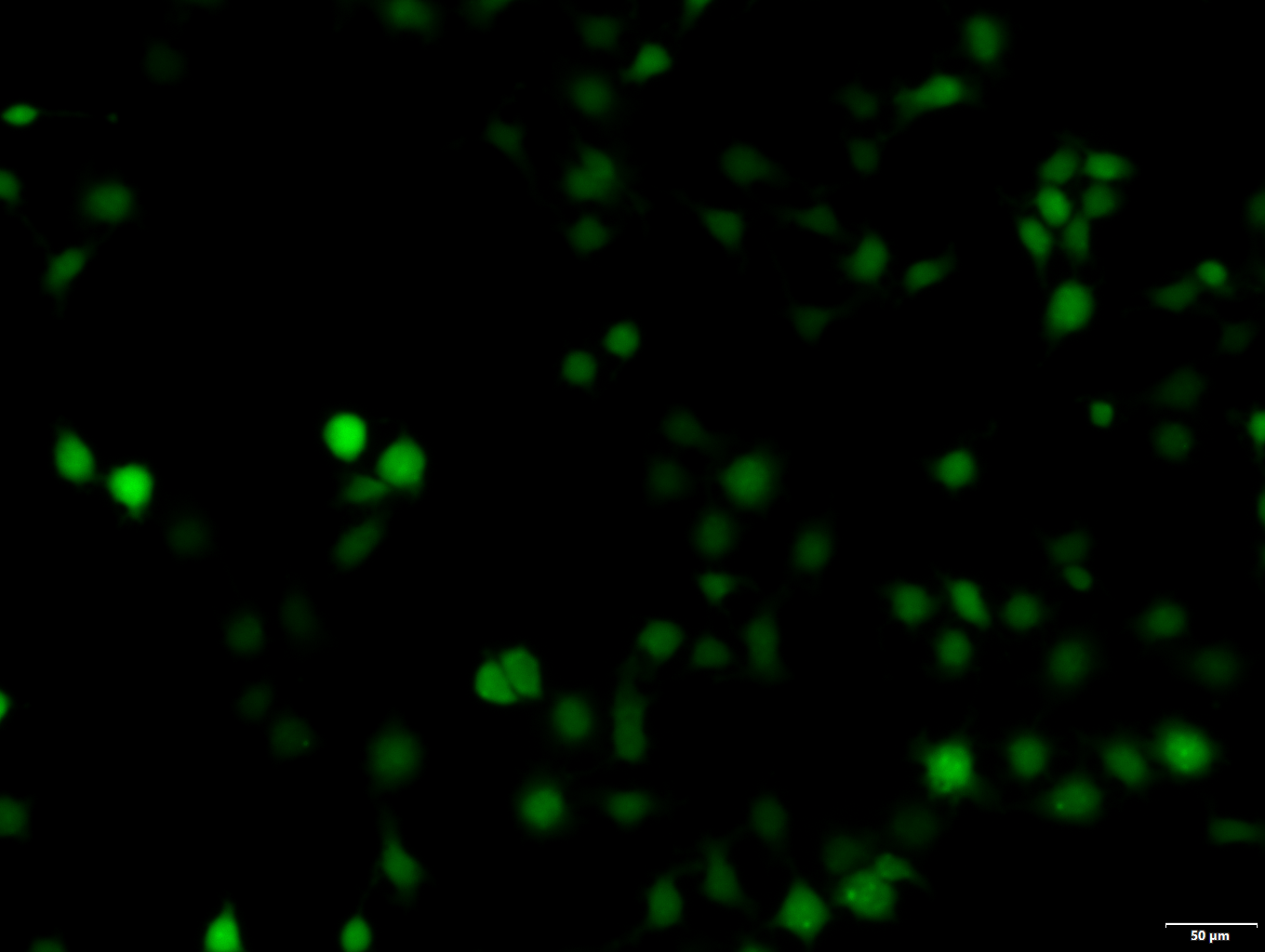

Supplement: Supplementary file 3 — Supplementary Information 3. [file 41598_2024_55043_MOESM3_ESM.zip › Supplementary material/ROS/20230416/H2O2-2.tif]

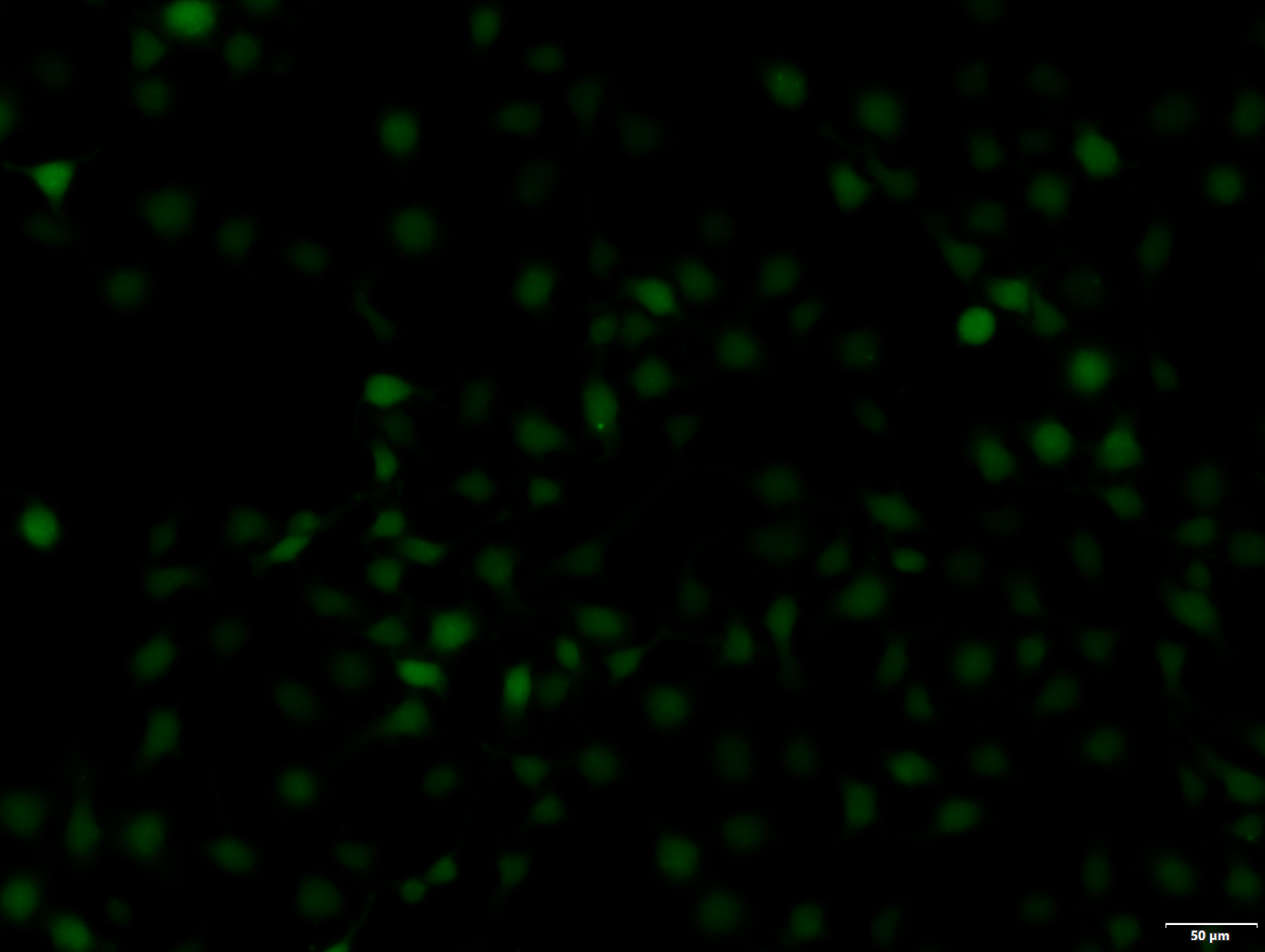

Supplement: Supplementary file 3 — Supplementary Information 3. [file 41598_2024_55043_MOESM3_ESM.zip › Supplementary material/ROS/20230416/UDCA.tif]

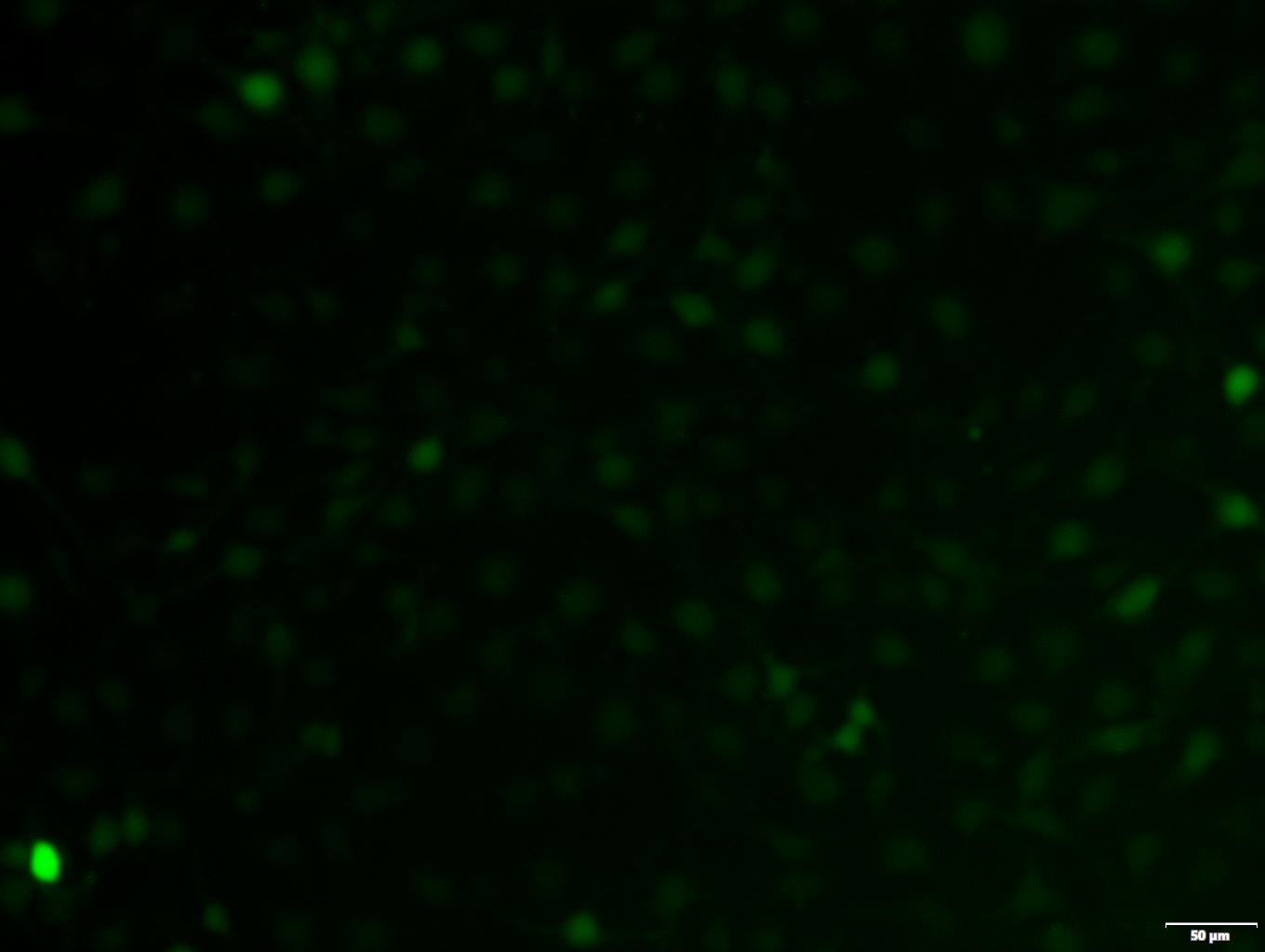

Supplement: Supplementary file 3 — Supplementary Information 3. [file 41598_2024_55043_MOESM3_ESM.zip › Supplementary material/ROS/20230416/H2O2+UDCA3.tif]

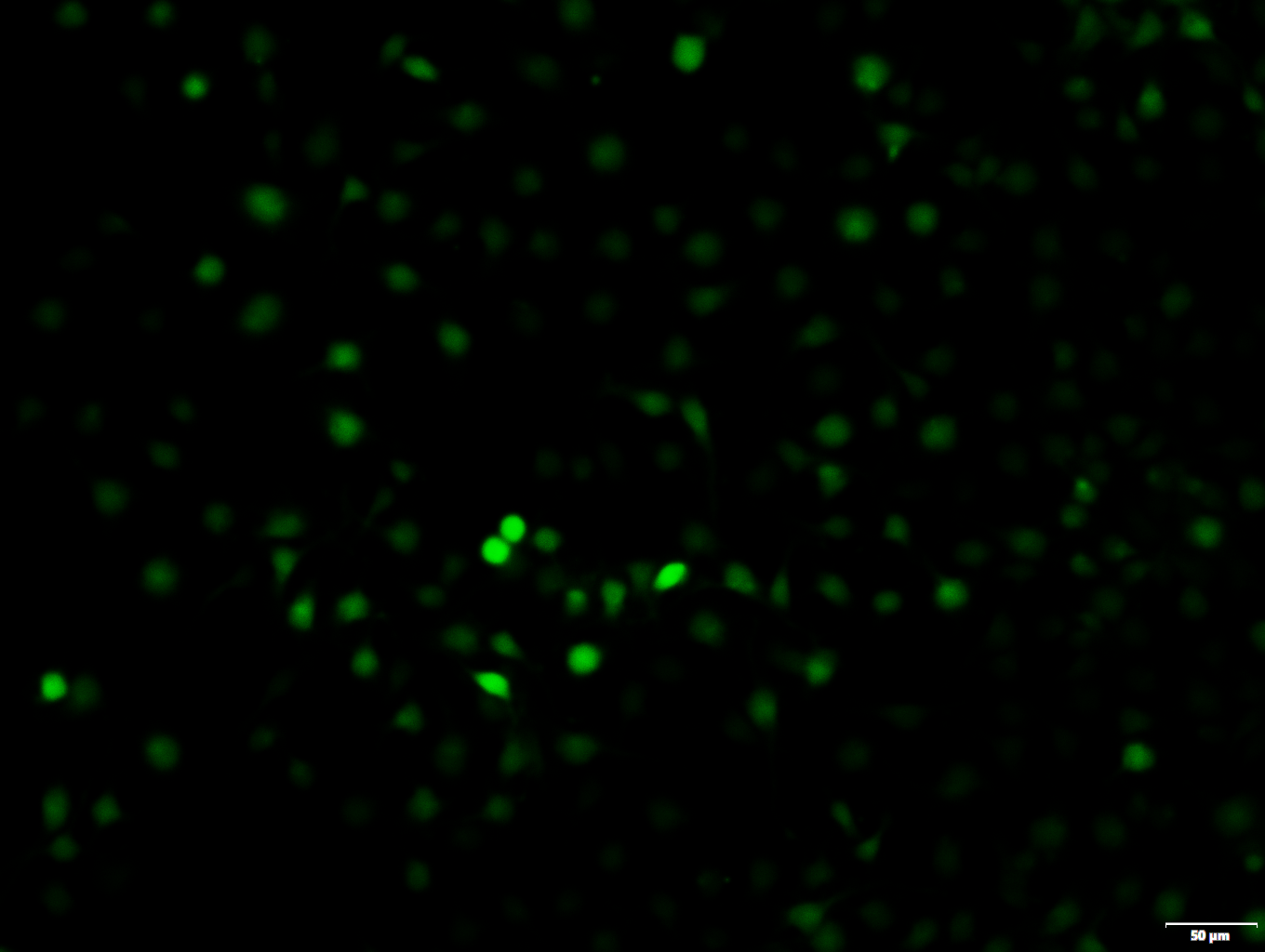

Supplement: Supplementary file 3 — Supplementary Information 3. [file 41598_2024_55043_MOESM3_ESM.zip › Supplementary material/ROS/20230416/H2O2+UDCA2.tif]

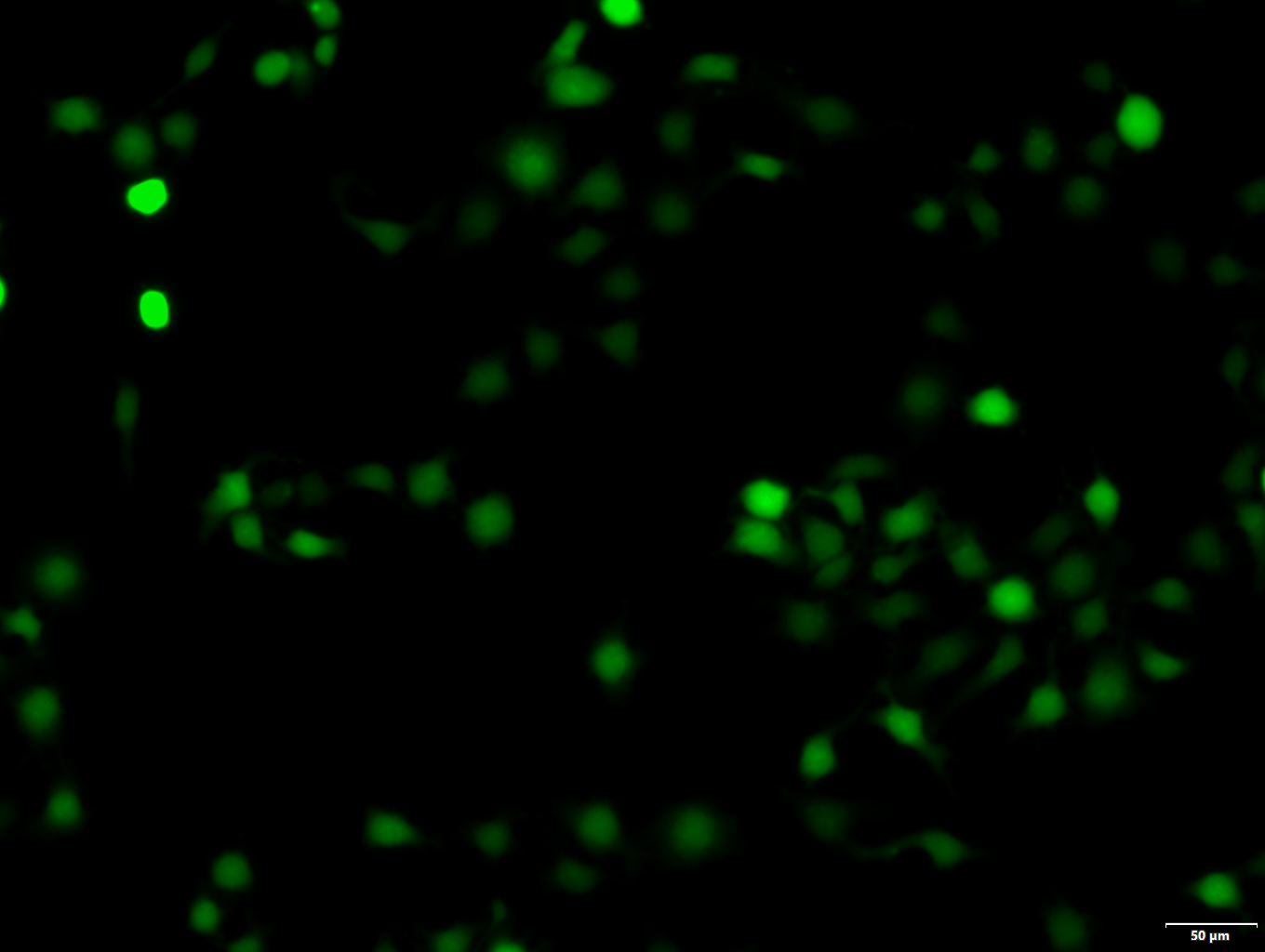

Supplement: Supplementary file 3 — Supplementary Information 3. [file 41598_2024_55043_MOESM3_ESM.zip › Supplementary material/ROS/20230416/H2O2.tif]

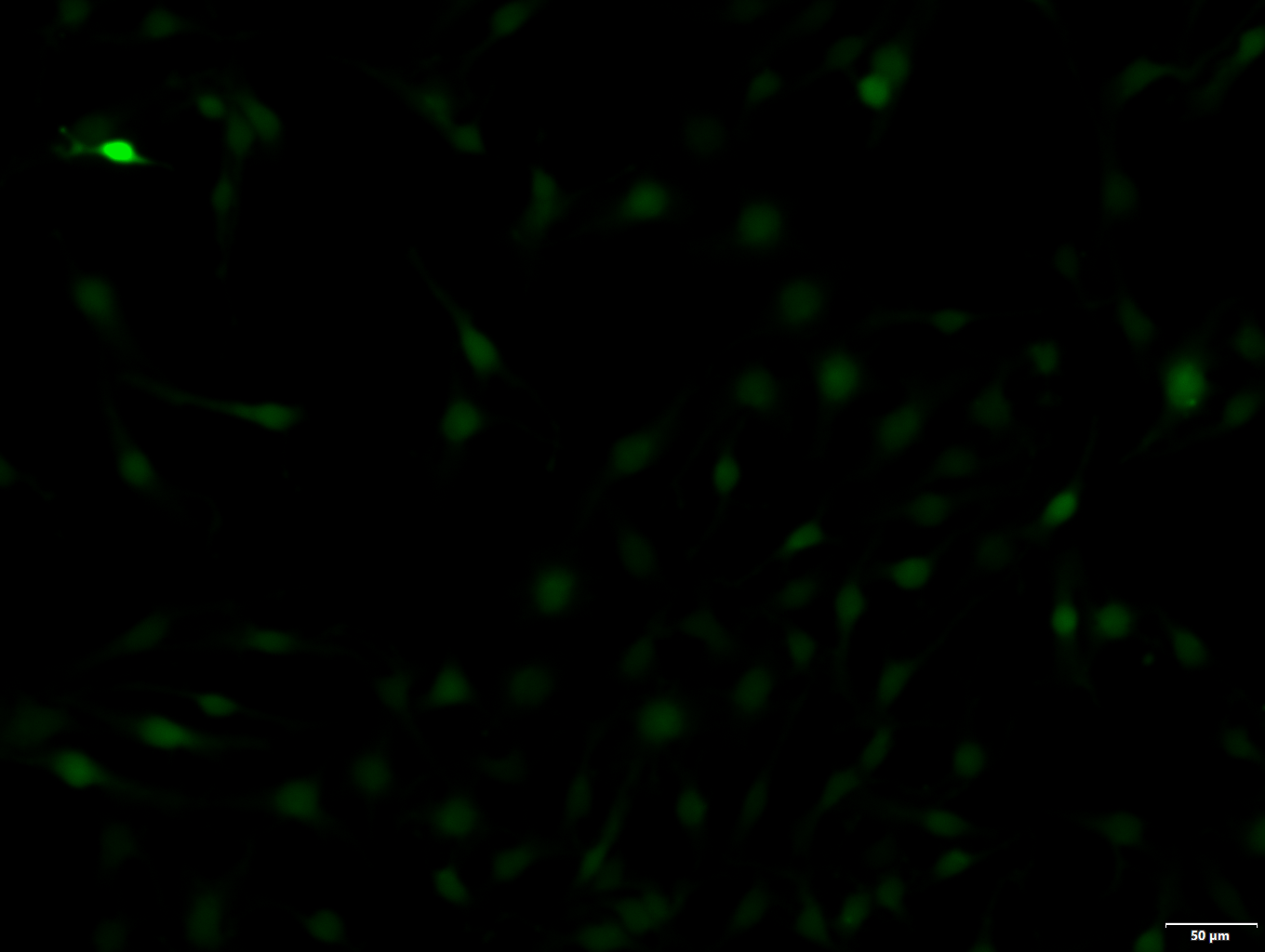

Supplement: Supplementary file 3 — Supplementary Information 3. [file 41598_2024_55043_MOESM3_ESM.zip › Supplementary material/ROS/20230416/Con.tif]

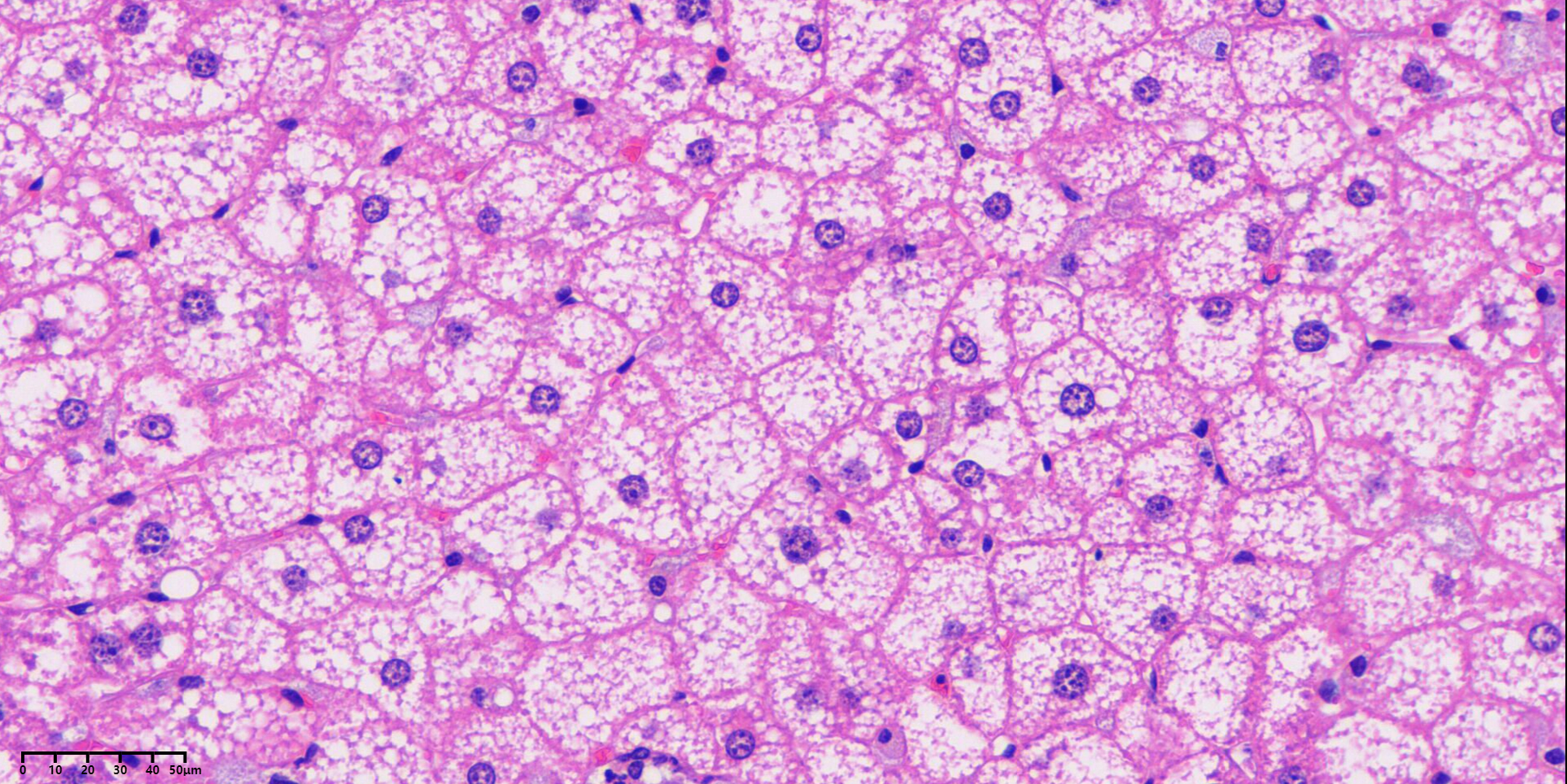

Supplement: Supplementary file 3 — Supplementary Information 3. [file 41598_2024_55043_MOESM3_ESM.zip › Supplementary material/HE/AS+UDCA 25/AS8.jpg]

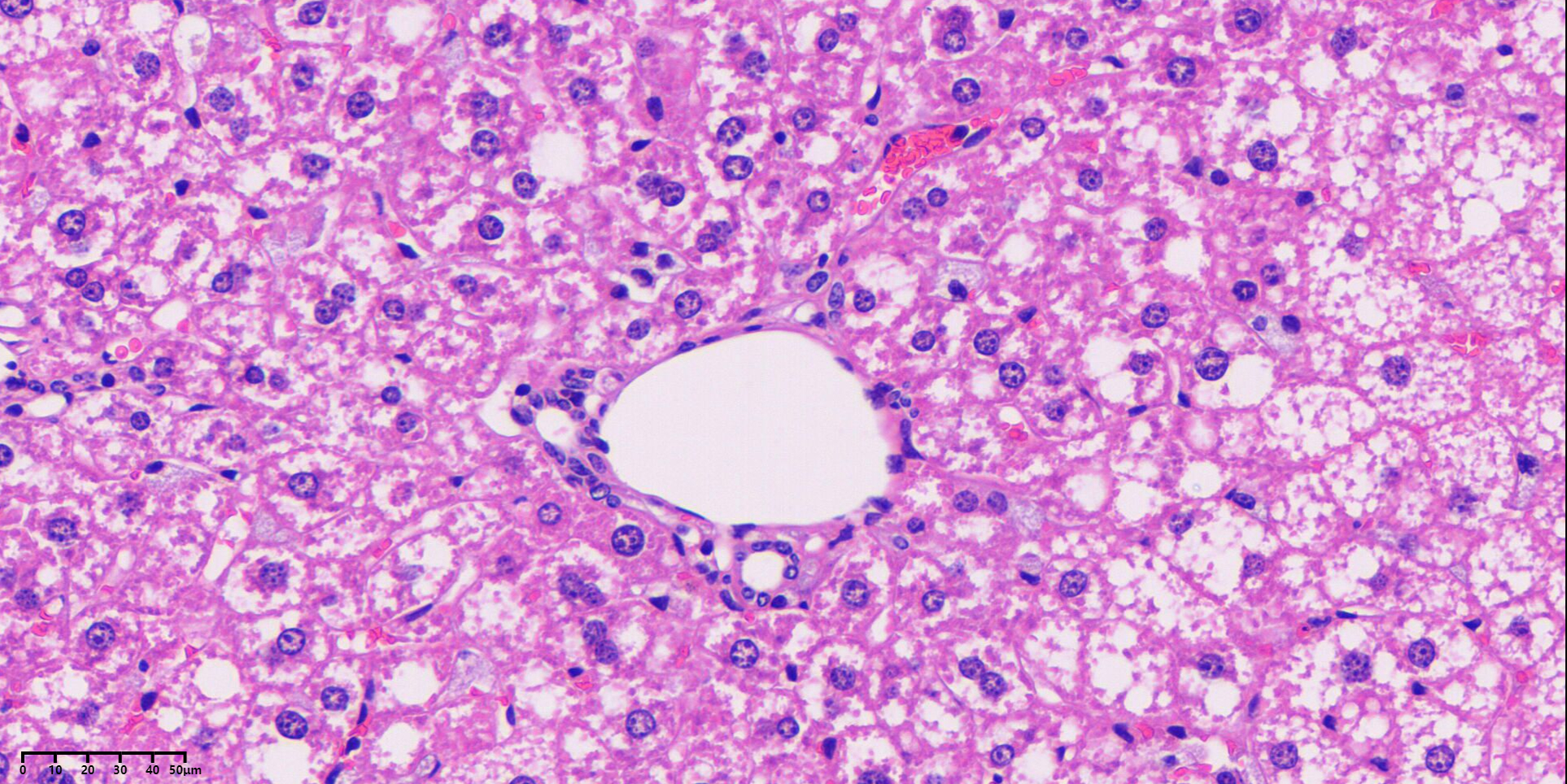

Supplement: Supplementary file 3 — Supplementary Information 3. [file 41598_2024_55043_MOESM3_ESM.zip › Supplementary material/HE/AS+UDCA 25/AS9.jpg]

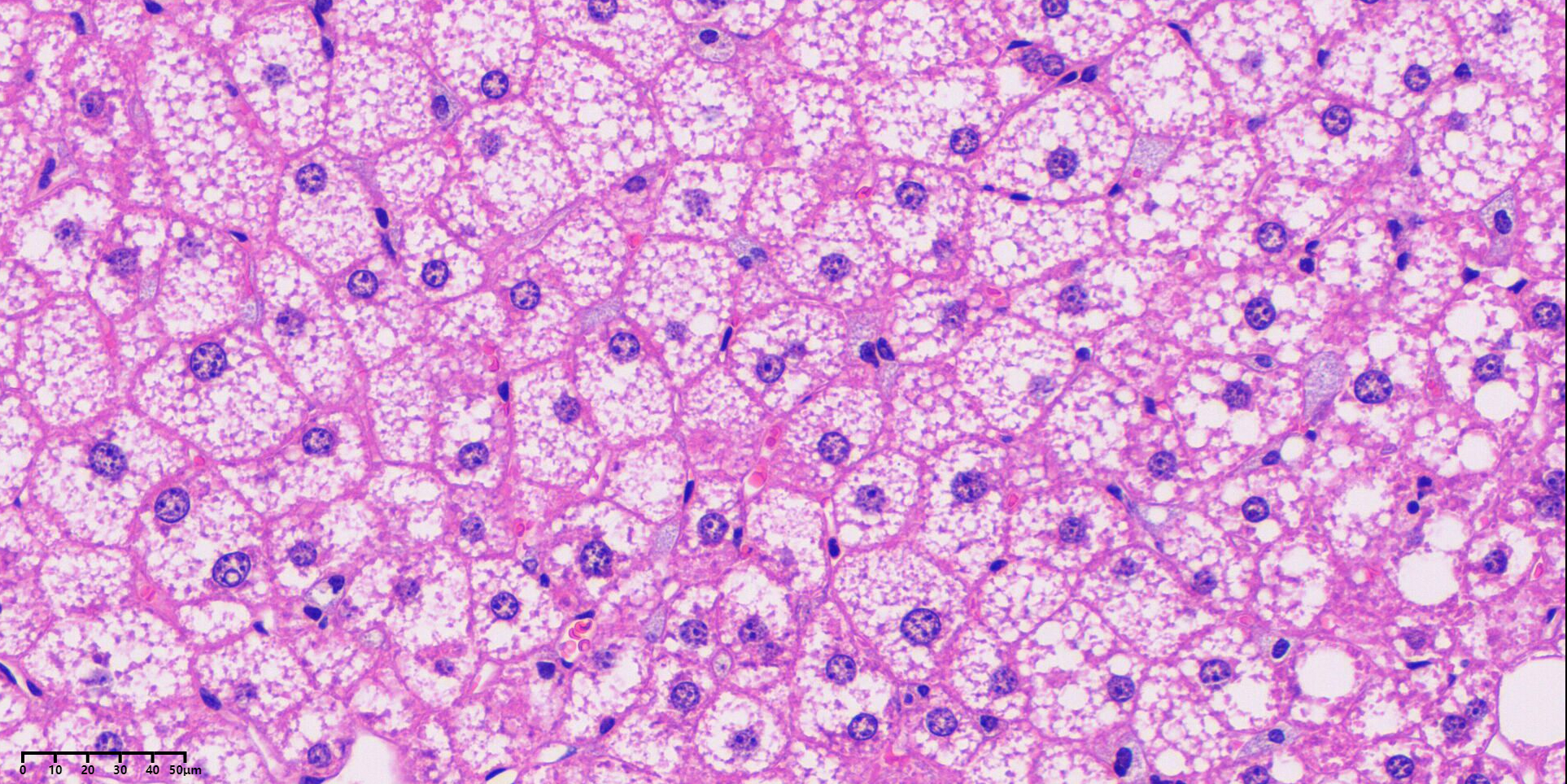

Supplement: Supplementary file 3 — Supplementary Information 3. [file 41598_2024_55043_MOESM3_ESM.zip › Supplementary material/HE/AS+UDCA 25/AS10.jpg]

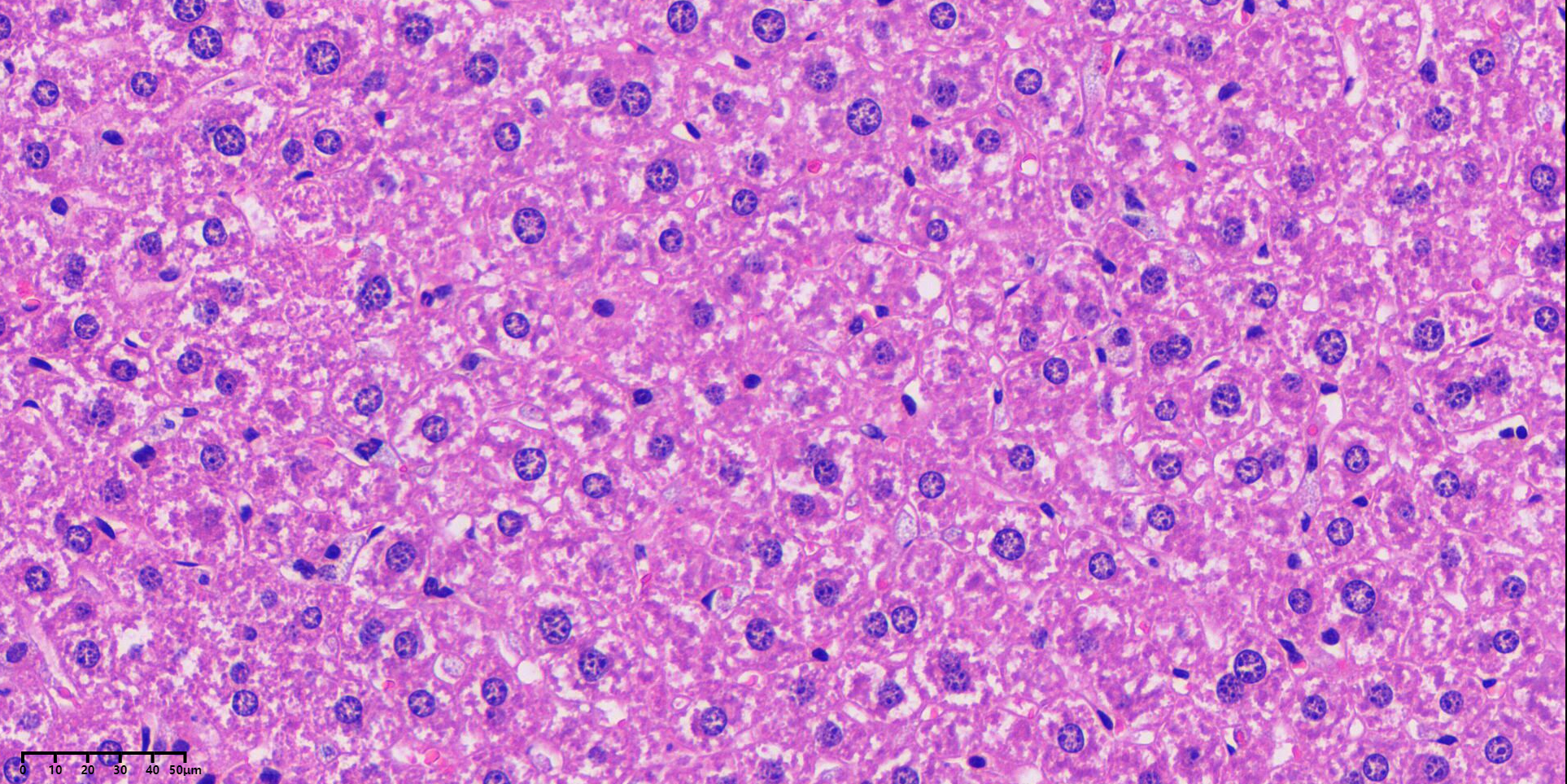

Supplement: Supplementary file 3 — Supplementary Information 3. [file 41598_2024_55043_MOESM3_ESM.zip › Supplementary material/HE/AS+UDCA 25/AS7.jpg]

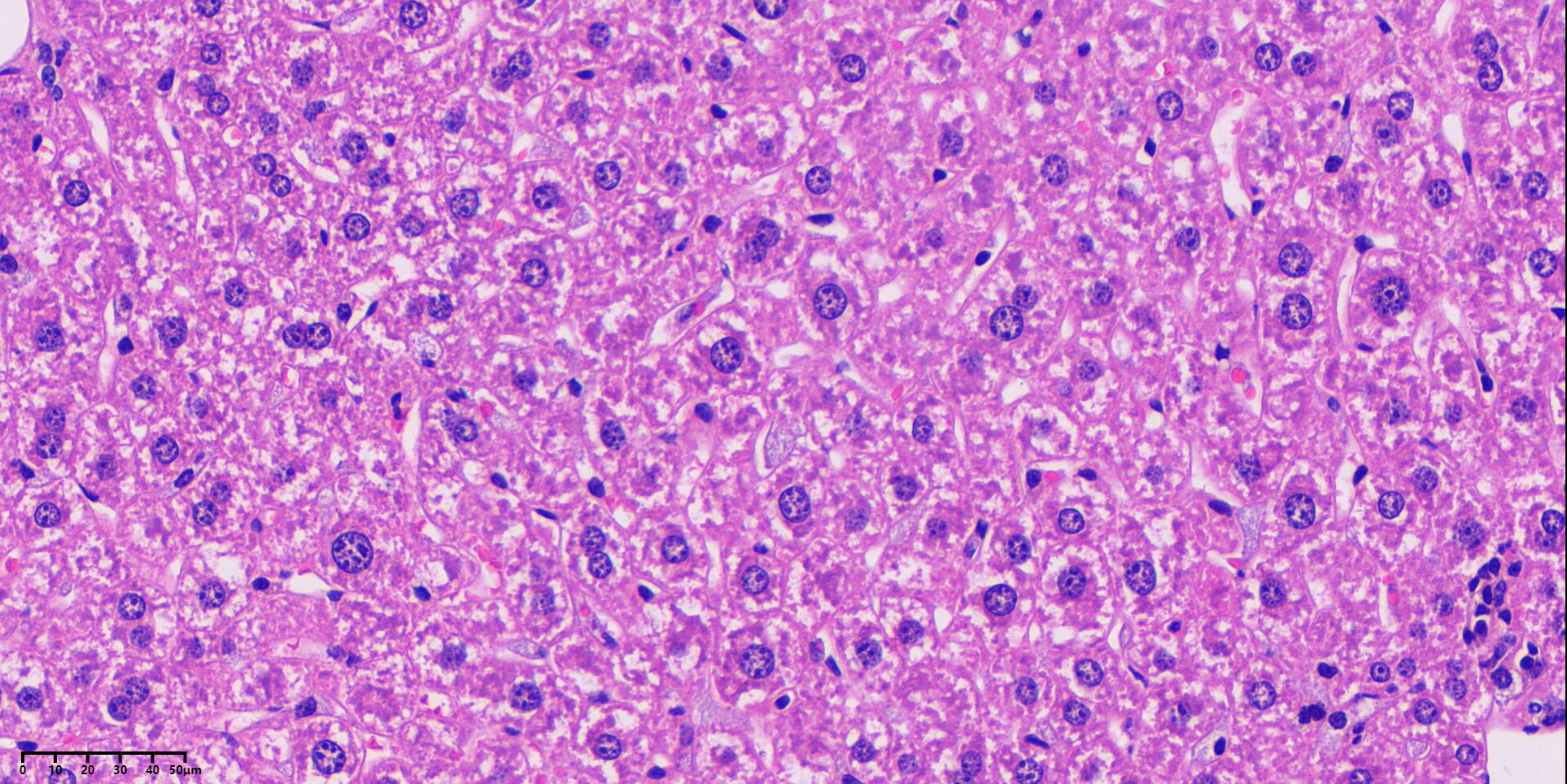

Supplement: Supplementary file 3 — Supplementary Information 3. [file 41598_2024_55043_MOESM3_ESM.zip › Supplementary material/HE/AS+UDCA 25/AS6.jpg]

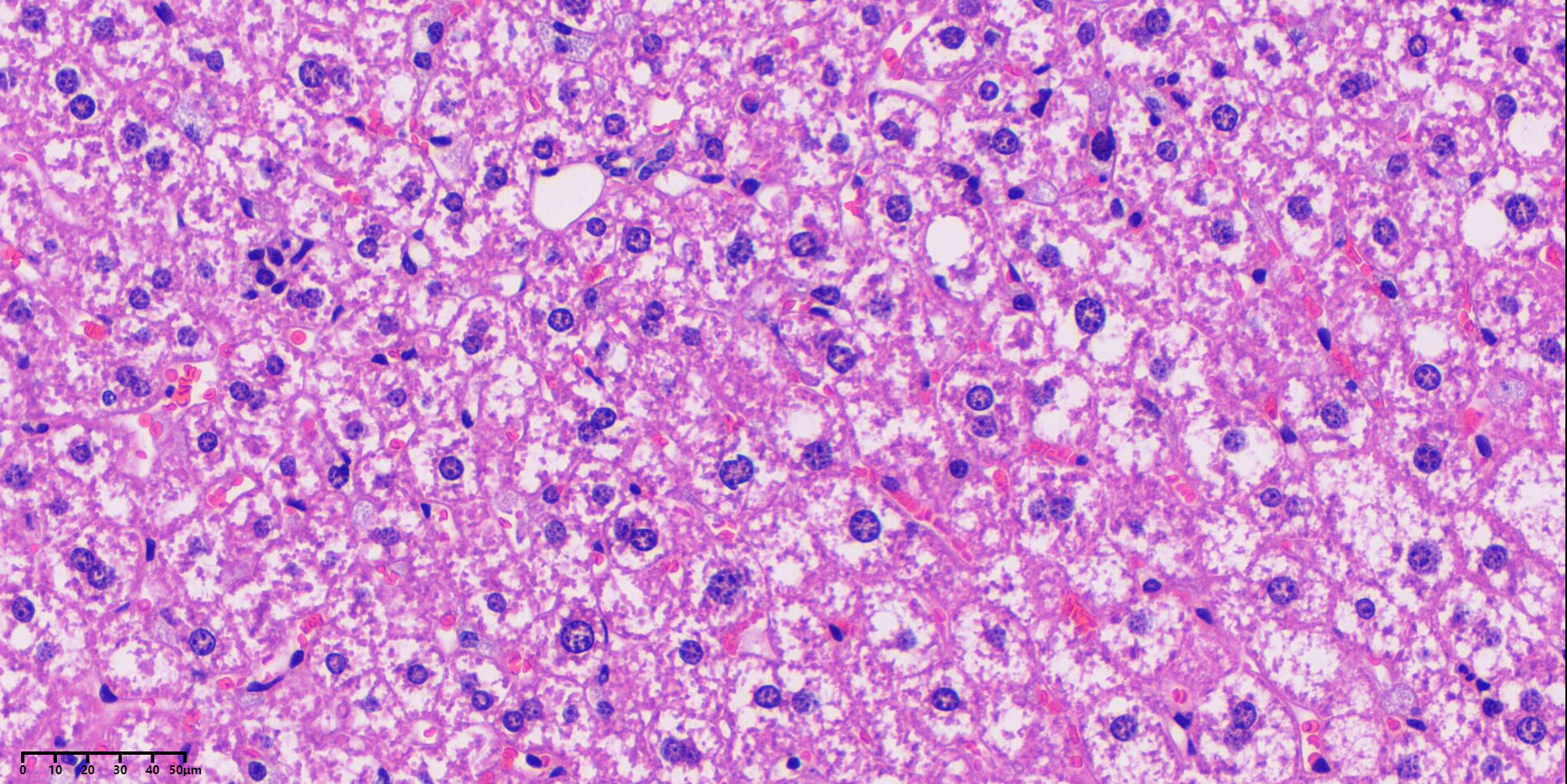

Supplement: Supplementary file 3 — Supplementary Information 3. [file 41598_2024_55043_MOESM3_ESM.zip › Supplementary material/HE/AS-model/AS18.jpg]

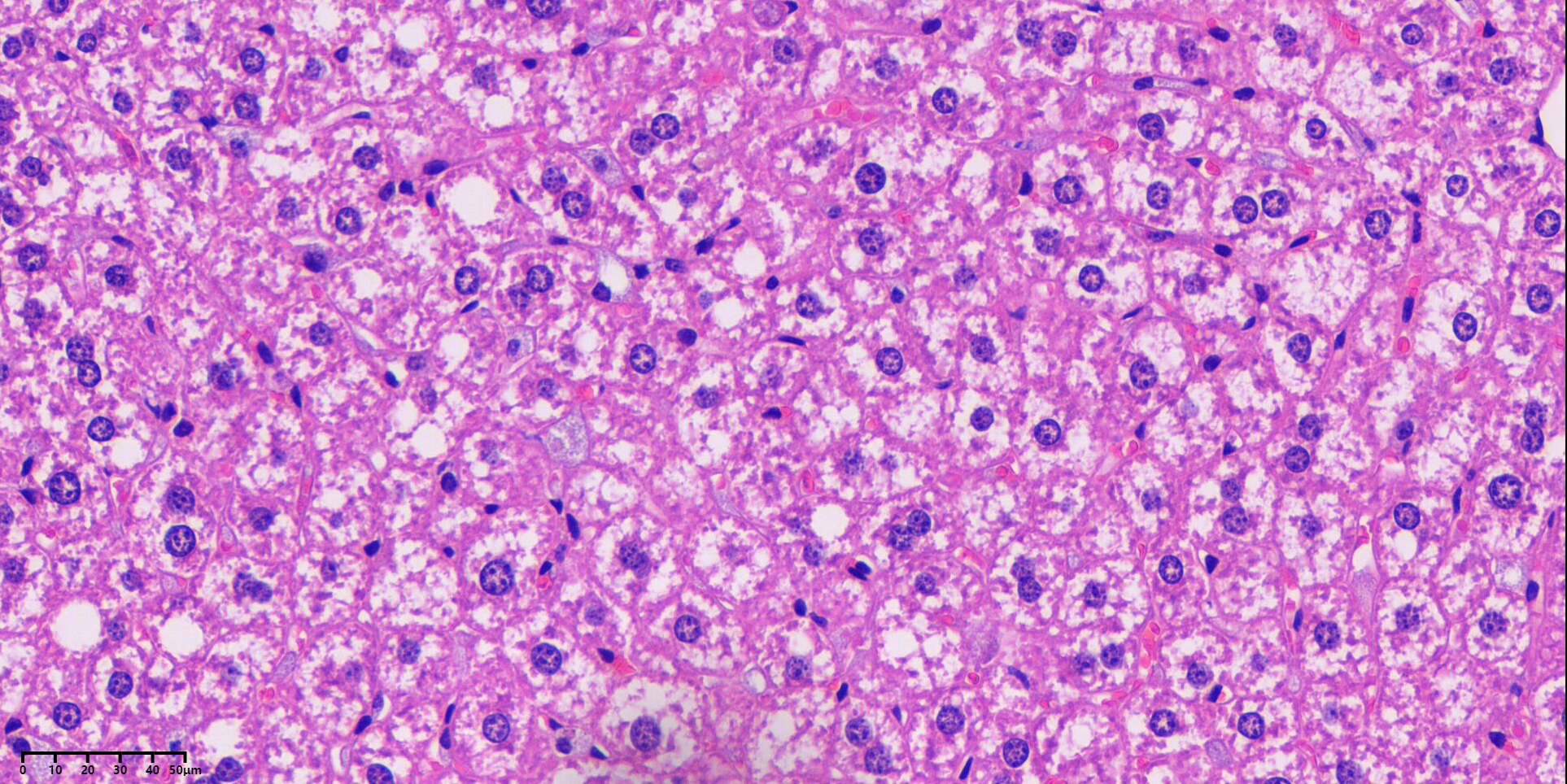

Supplement: Supplementary file 3 — Supplementary Information 3. [file 41598_2024_55043_MOESM3_ESM.zip › Supplementary material/HE/AS-model/AS19.jpg]

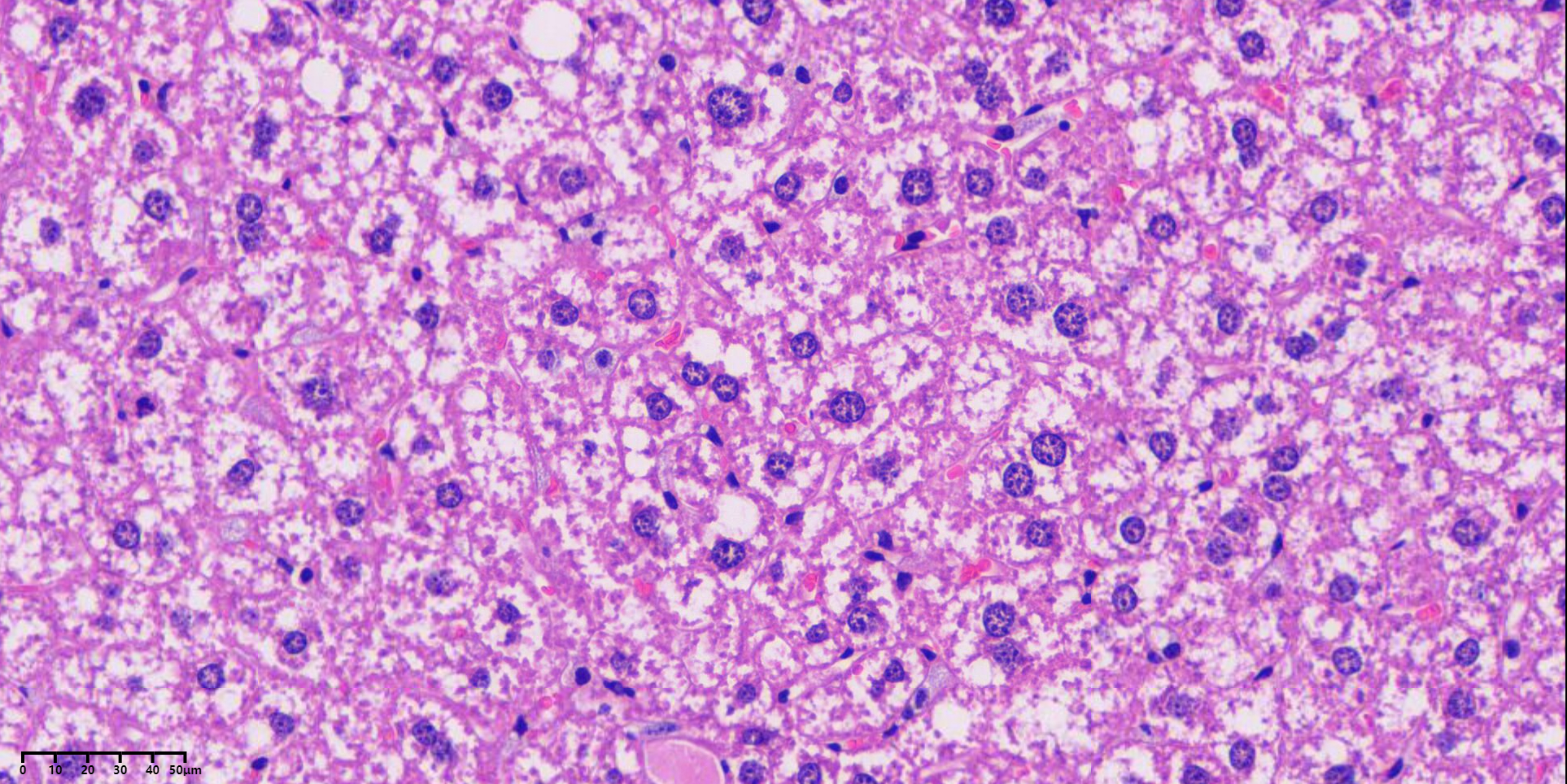

Supplement: Supplementary file 3 — Supplementary Information 3. [file 41598_2024_55043_MOESM3_ESM.zip › Supplementary material/HE/AS-model/AS20.jpg]

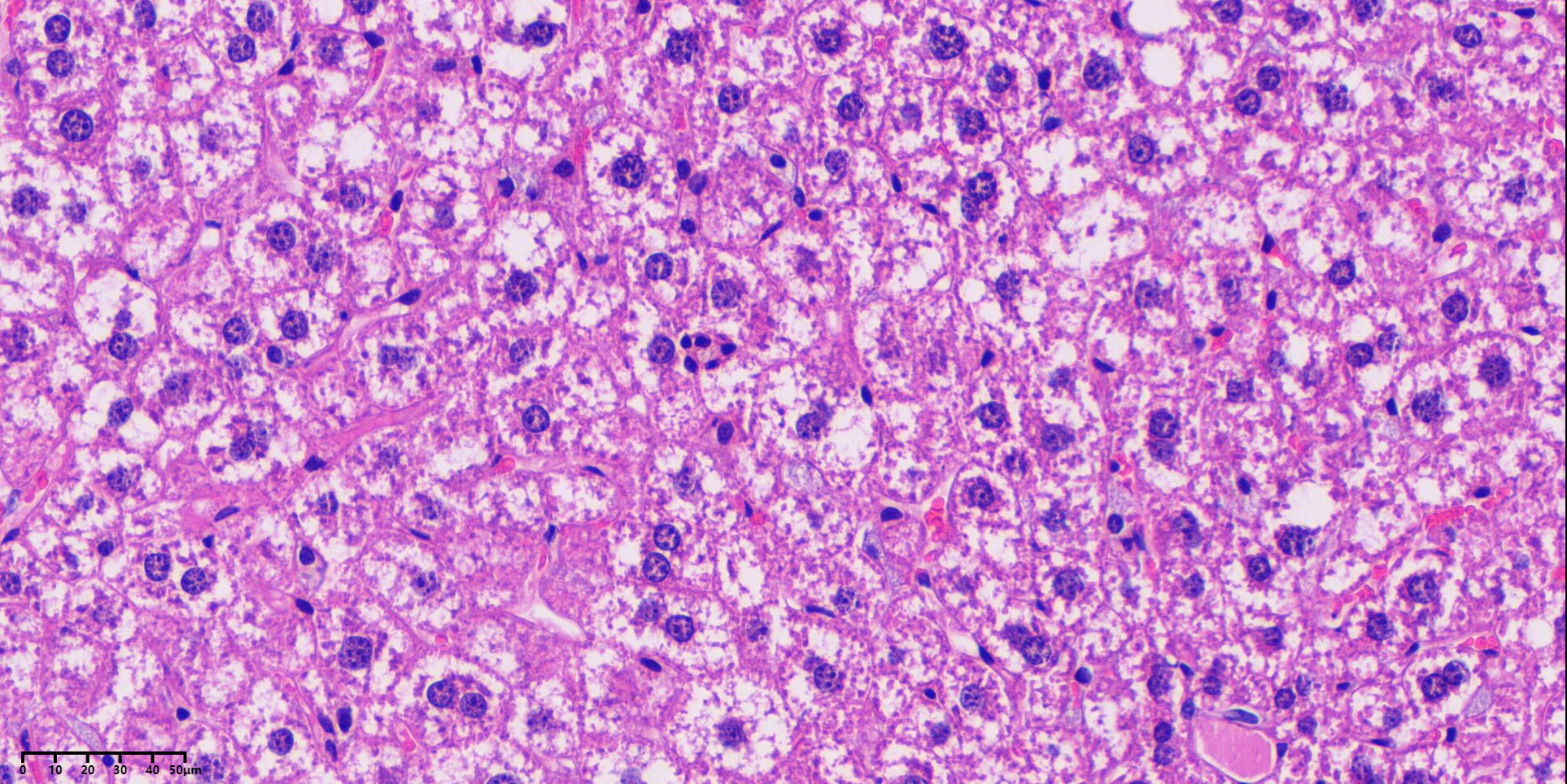

Supplement: Supplementary file 3 — Supplementary Information 3. [file 41598_2024_55043_MOESM3_ESM.zip › Supplementary material/HE/AS-model/AS17.jpg]

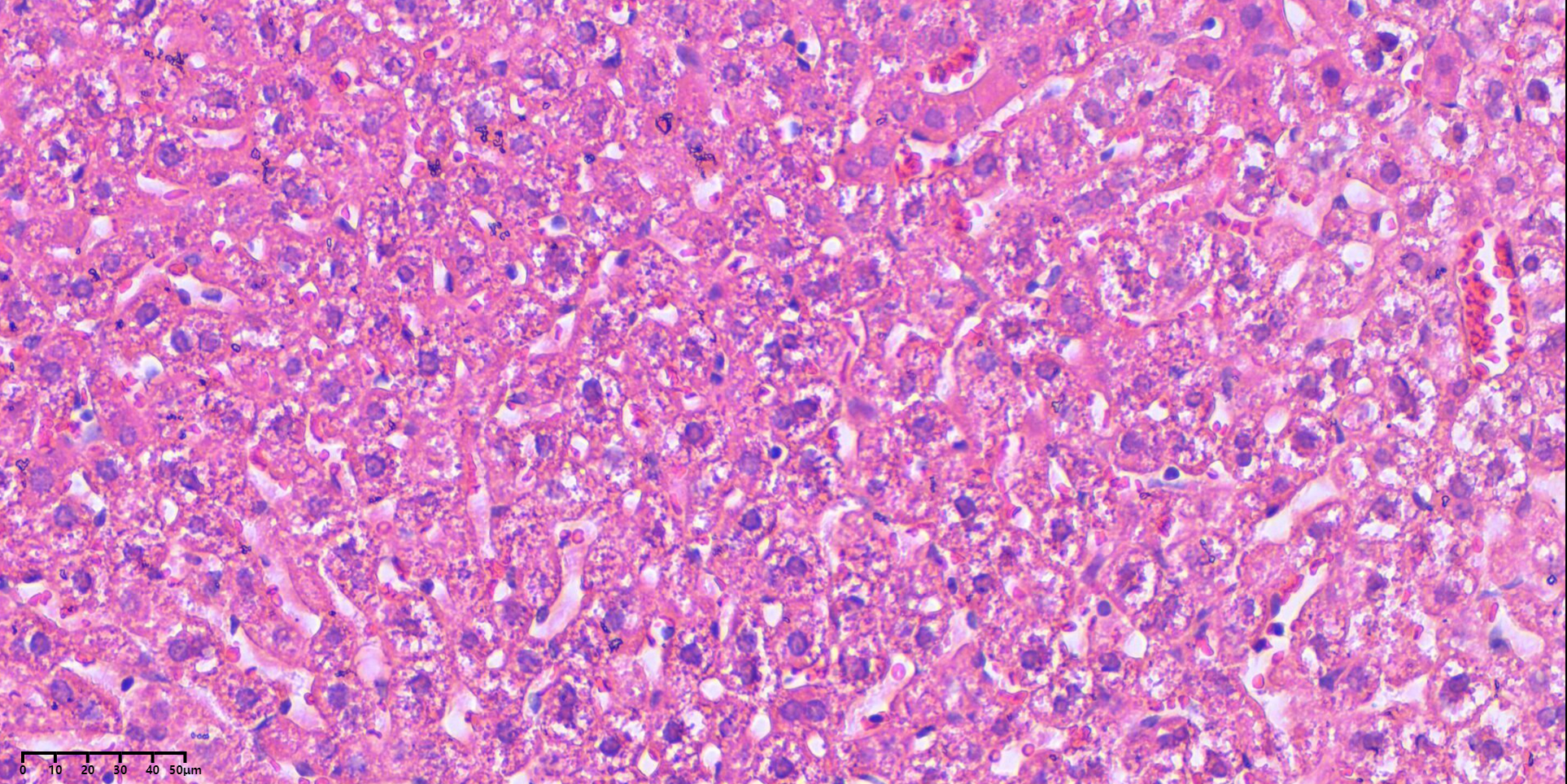

Supplement: Supplementary file 3 — Supplementary Information 3. [file 41598_2024_55043_MOESM3_ESM.zip › Supplementary material/HE/UDCA 25/zc5.jpg]

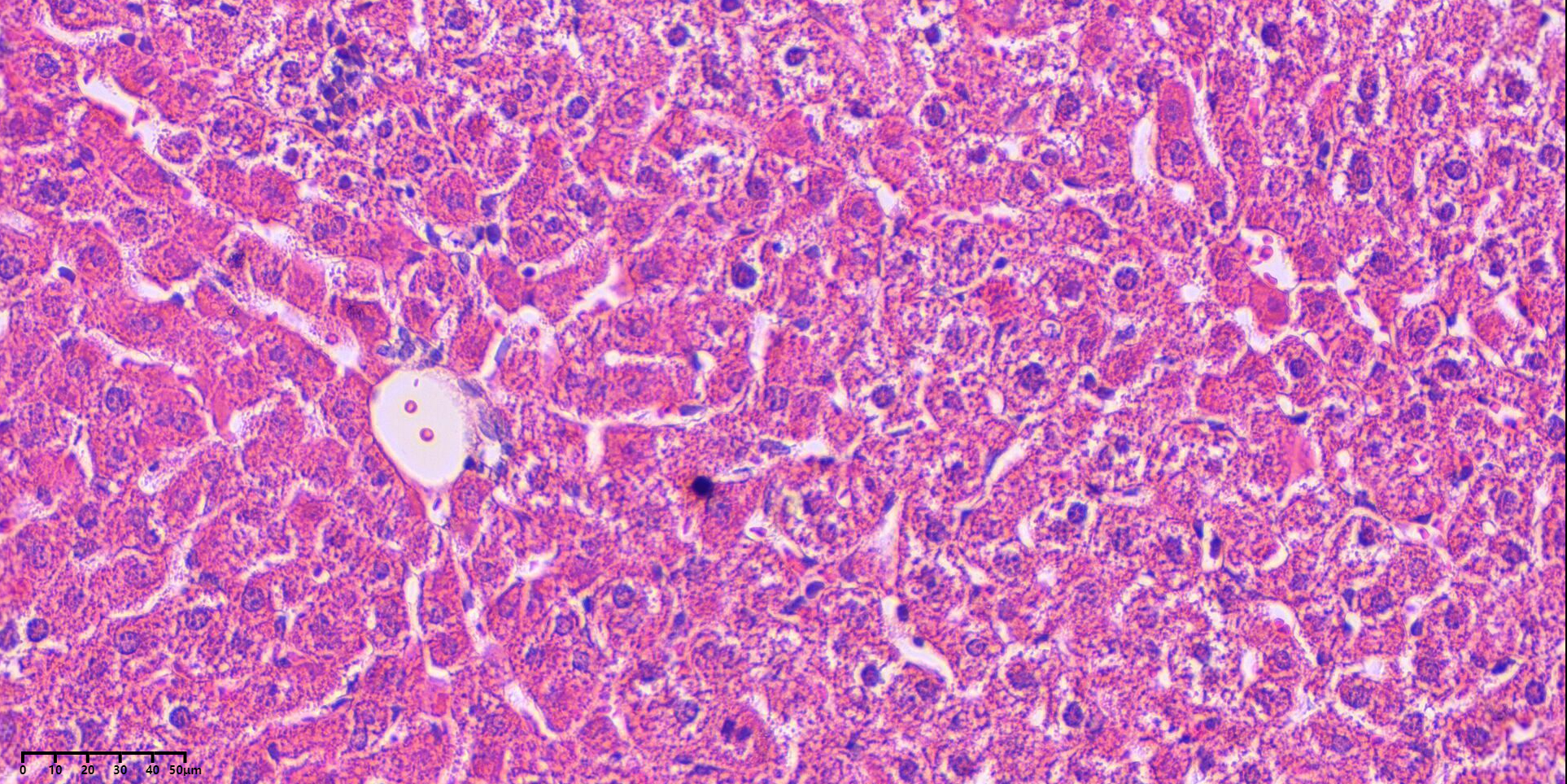

Supplement: Supplementary file 3 — Supplementary Information 3. [file 41598_2024_55043_MOESM3_ESM.zip › Supplementary material/HE/UDCA 25/zc4.jpg]

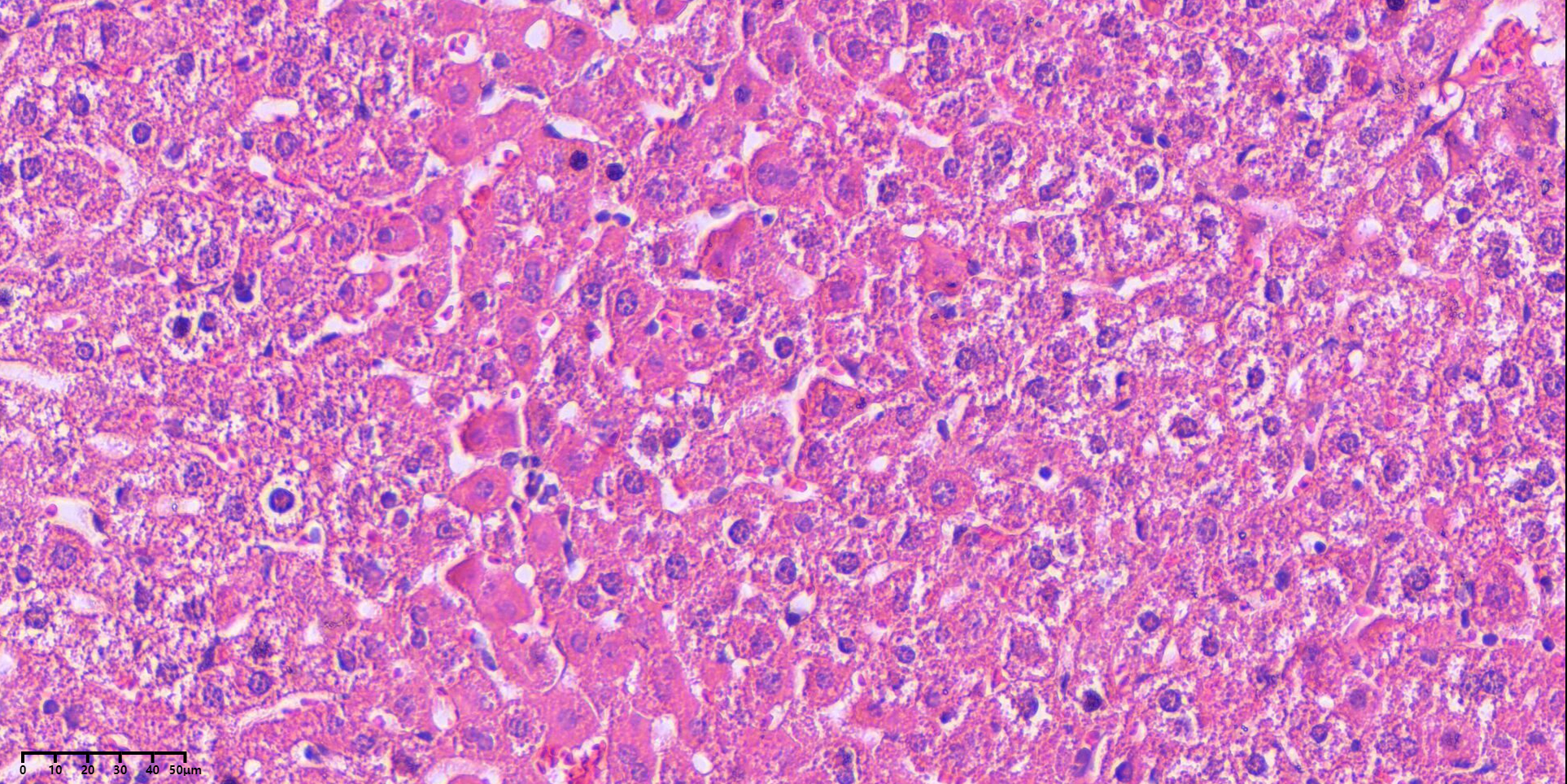

Supplement: Supplementary file 3 — Supplementary Information 3. [file 41598_2024_55043_MOESM3_ESM.zip › Supplementary material/HE/UDCA 25/zc3.jpg]

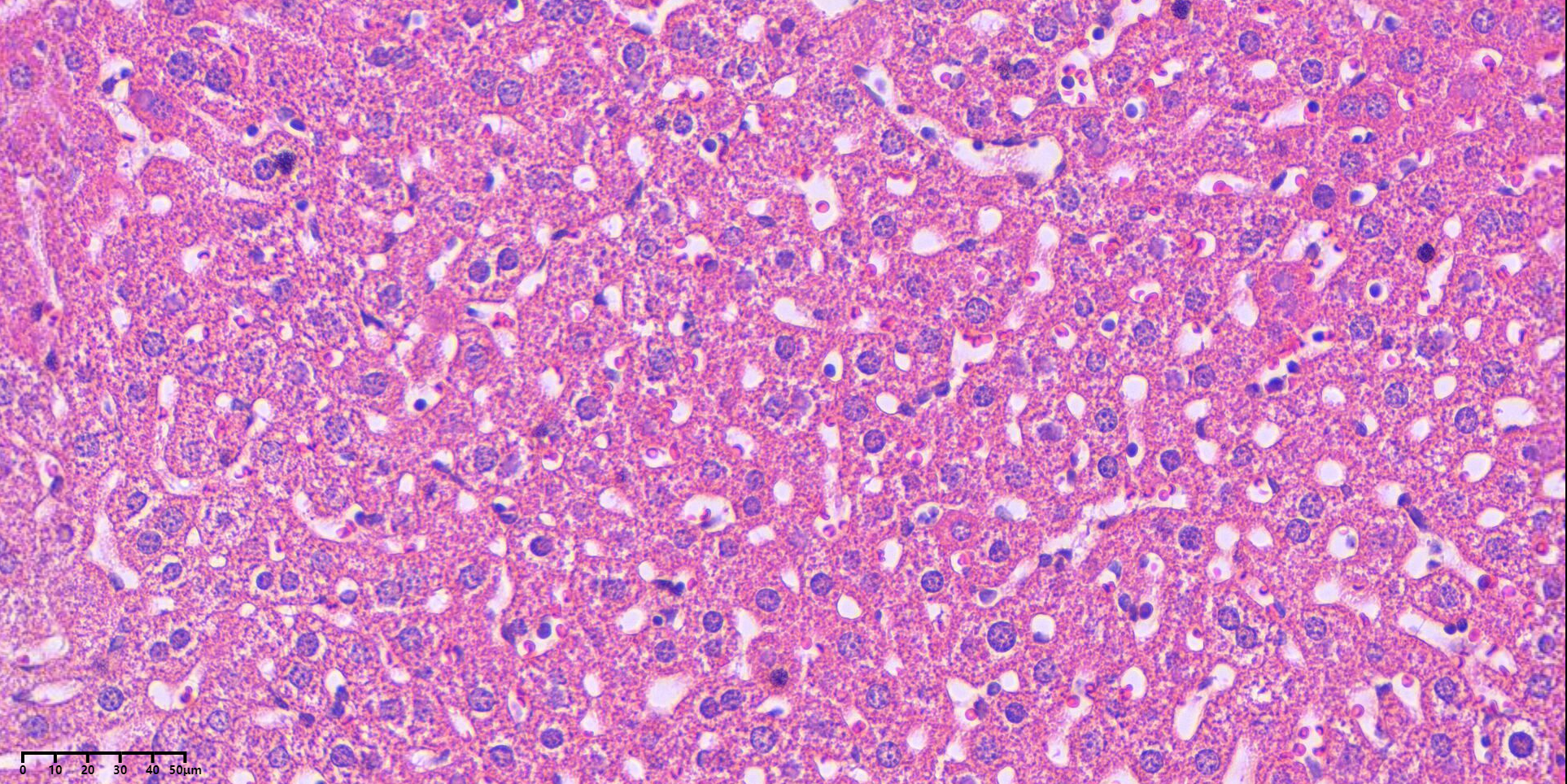

Supplement: Supplementary file 3 — Supplementary Information 3. [file 41598_2024_55043_MOESM3_ESM.zip › Supplementary material/HE/UDCA 25/zc2.jpg]

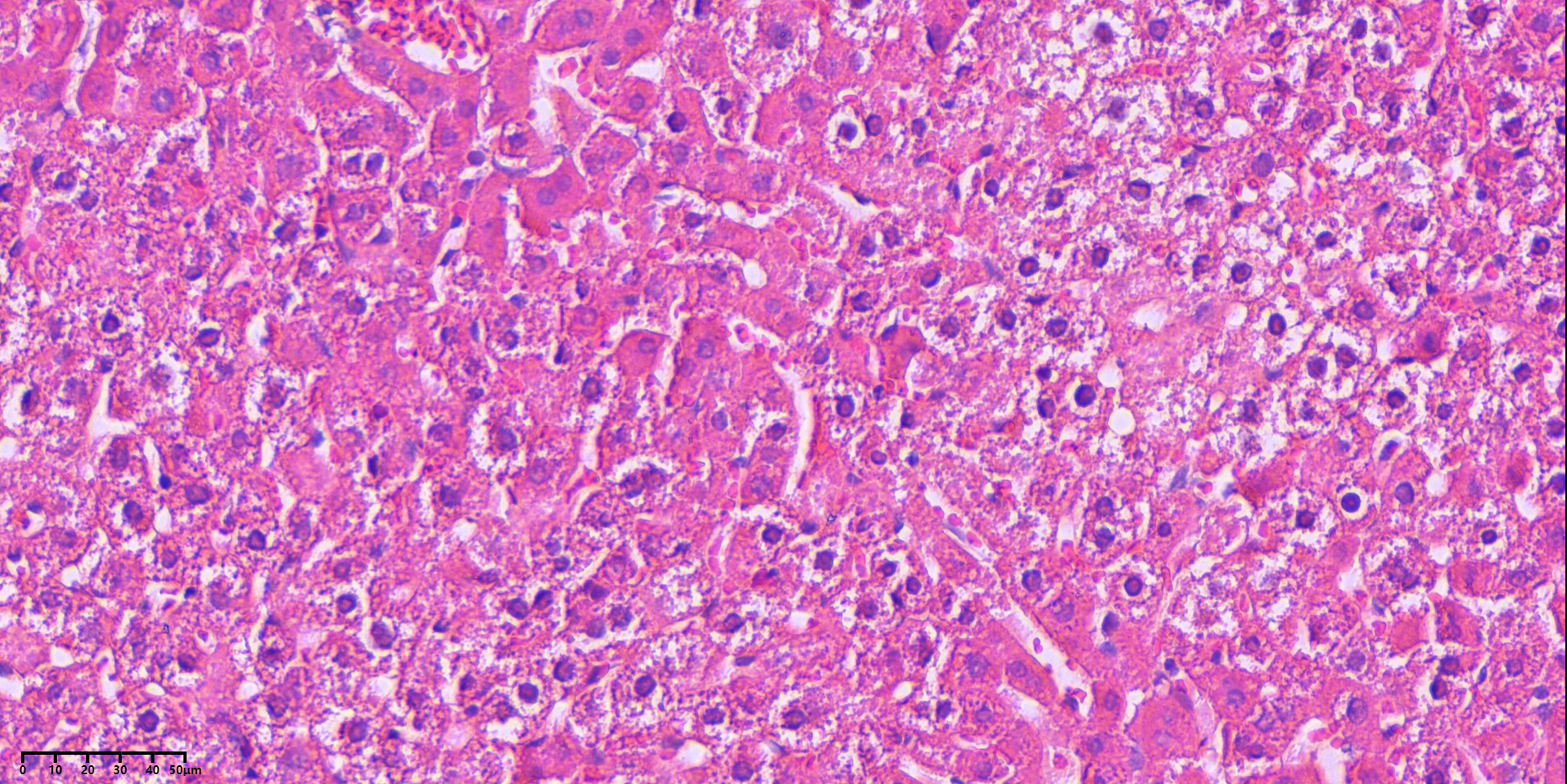

Supplement: Supplementary file 3 — Supplementary Information 3. [file 41598_2024_55043_MOESM3_ESM.zip › Supplementary material/HE/UDCA 25/zc1.jpg]

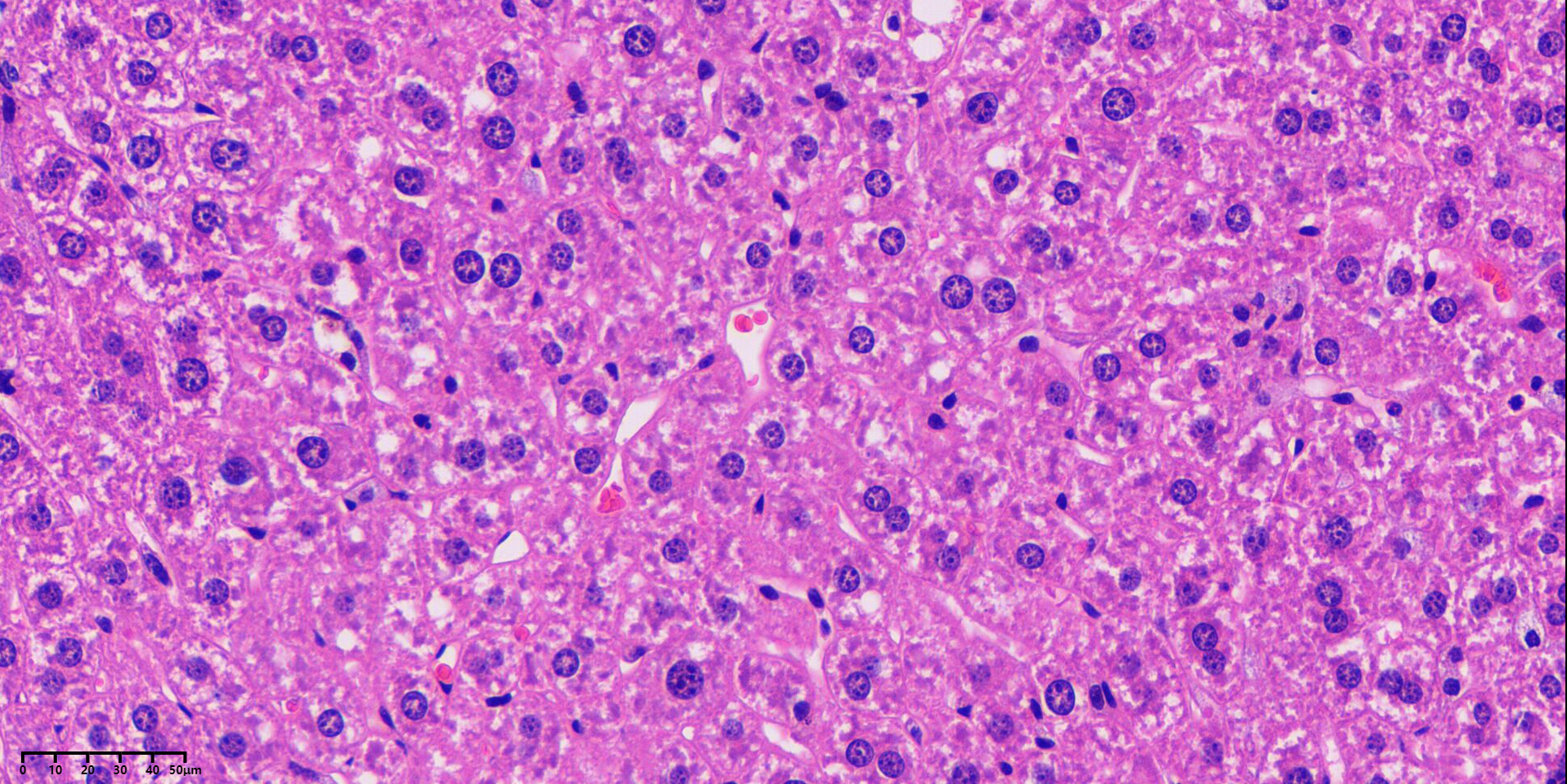

Supplement: Supplementary file 3 — Supplementary Information 3. [file 41598_2024_55043_MOESM3_ESM.zip › Supplementary material/HE/AS+UDCA 50/AS2.jpg]

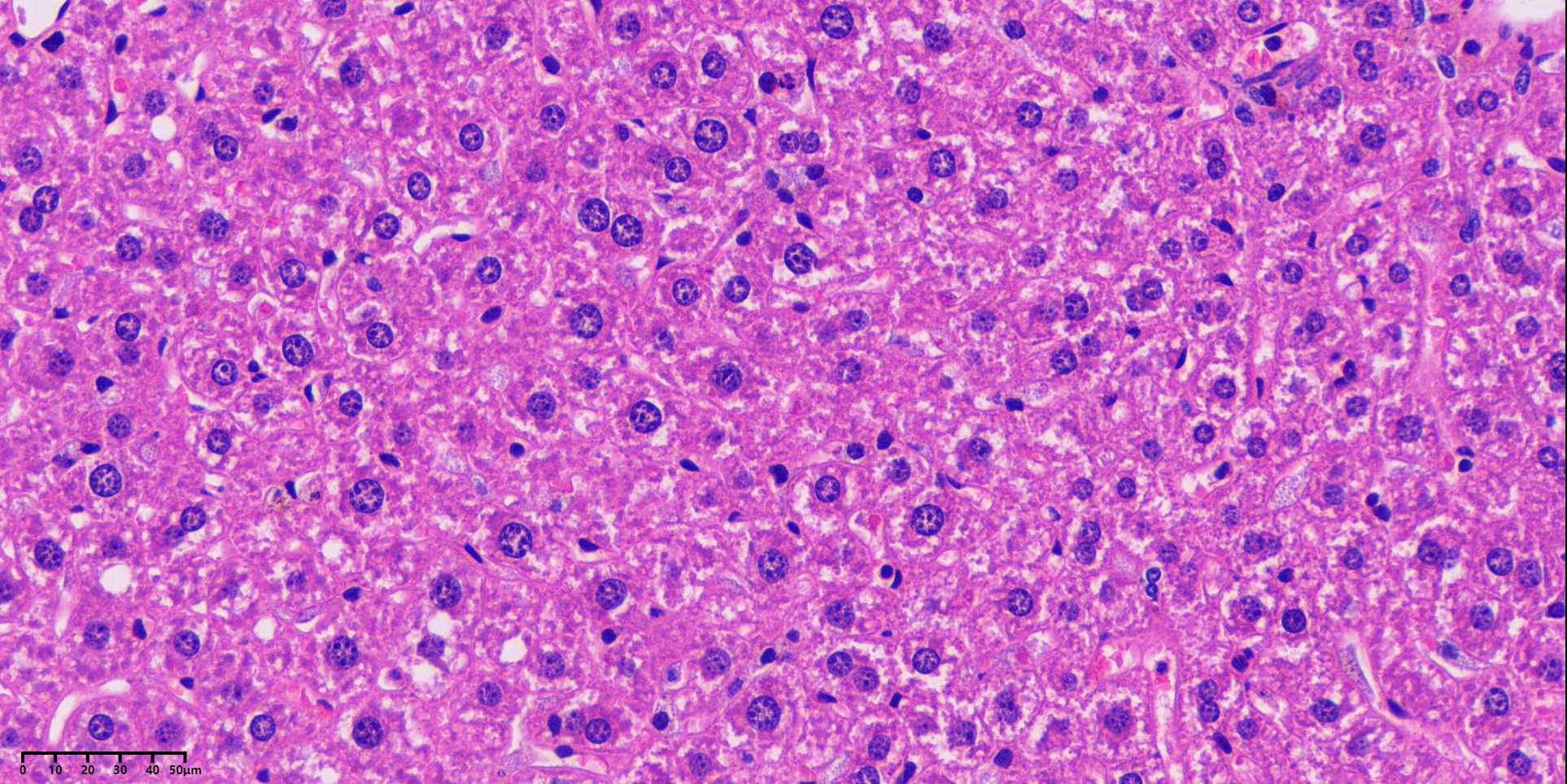

Supplement: Supplementary file 3 — Supplementary Information 3. [file 41598_2024_55043_MOESM3_ESM.zip › Supplementary material/HE/AS+UDCA 50/AS3.jpg]

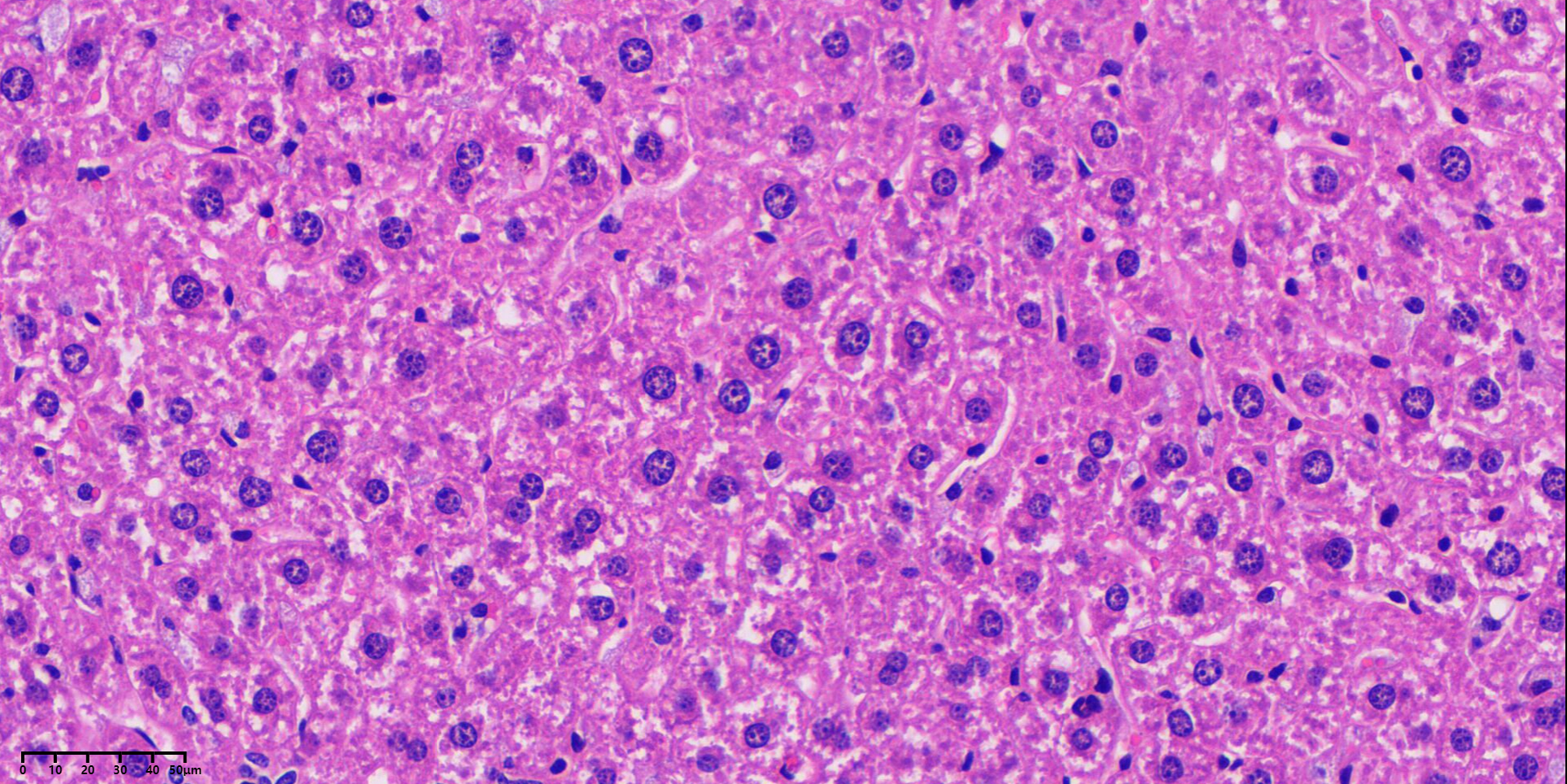

Supplement: Supplementary file 3 — Supplementary Information 3. [file 41598_2024_55043_MOESM3_ESM.zip › Supplementary material/HE/AS+UDCA 50/AS1.jpg]

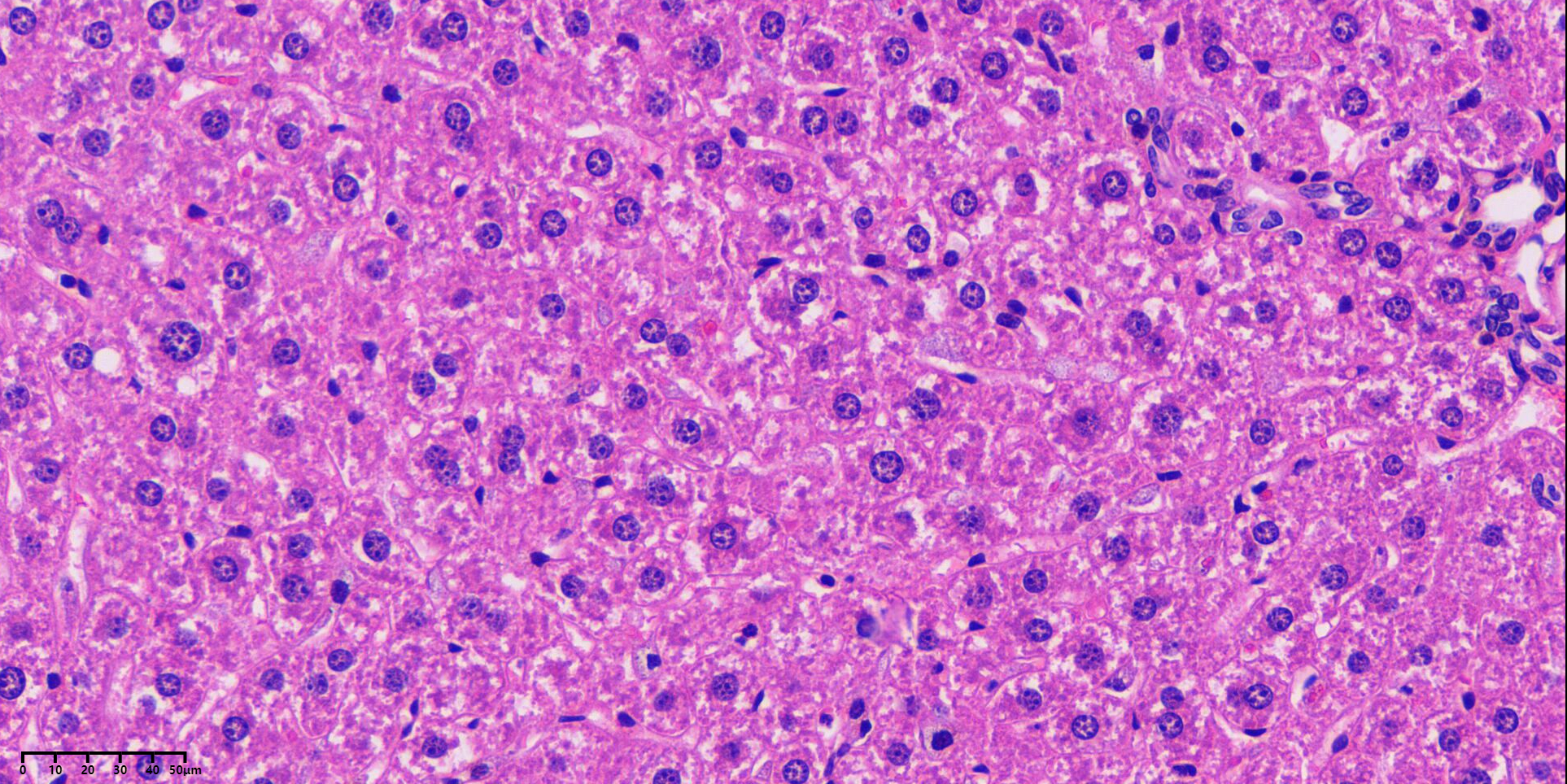

Supplement: Supplementary file 3 — Supplementary Information 3. [file 41598_2024_55043_MOESM3_ESM.zip › Supplementary material/HE/AS+UDCA 50/AS4.jpg]

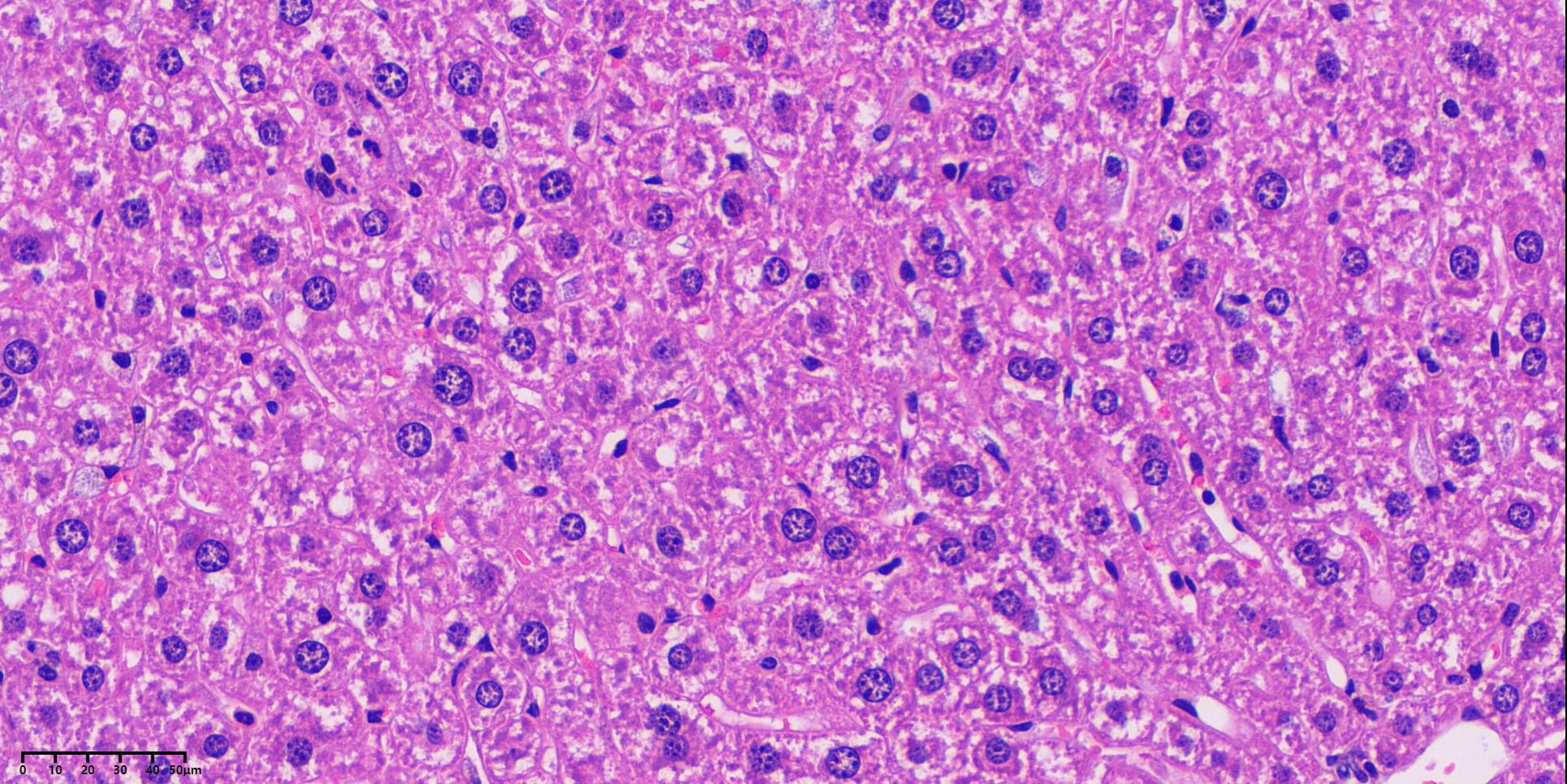

Supplement: Supplementary file 3 — Supplementary Information 3. [file 41598_2024_55043_MOESM3_ESM.zip › Supplementary material/HE/AS+UDCA 50/AS5.jpg]

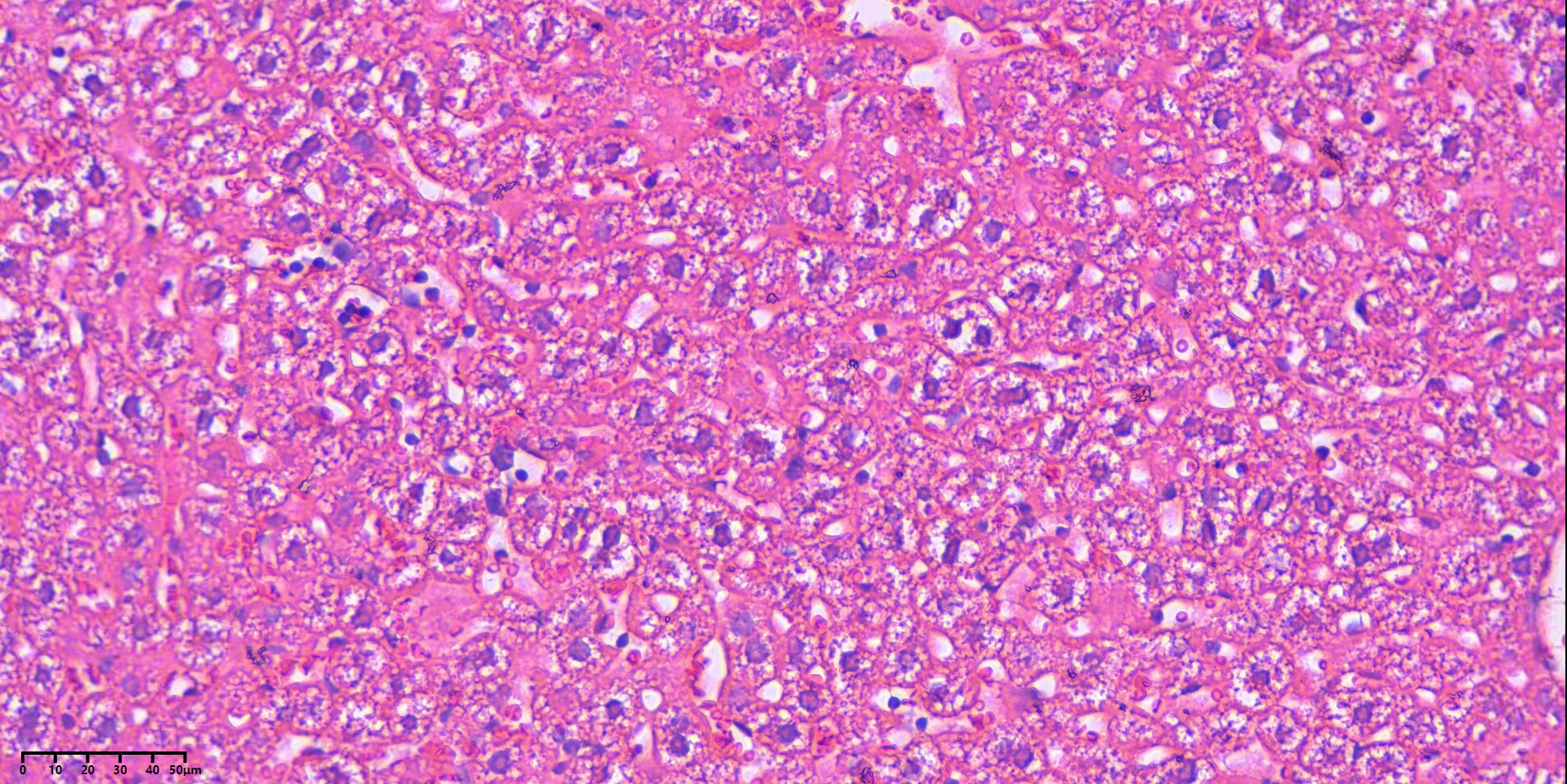

Supplement: Supplementary file 3 — Supplementary Information 3. [file 41598_2024_55043_MOESM3_ESM.zip › Supplementary material/HE/UCDA 50/zc6.jpg]

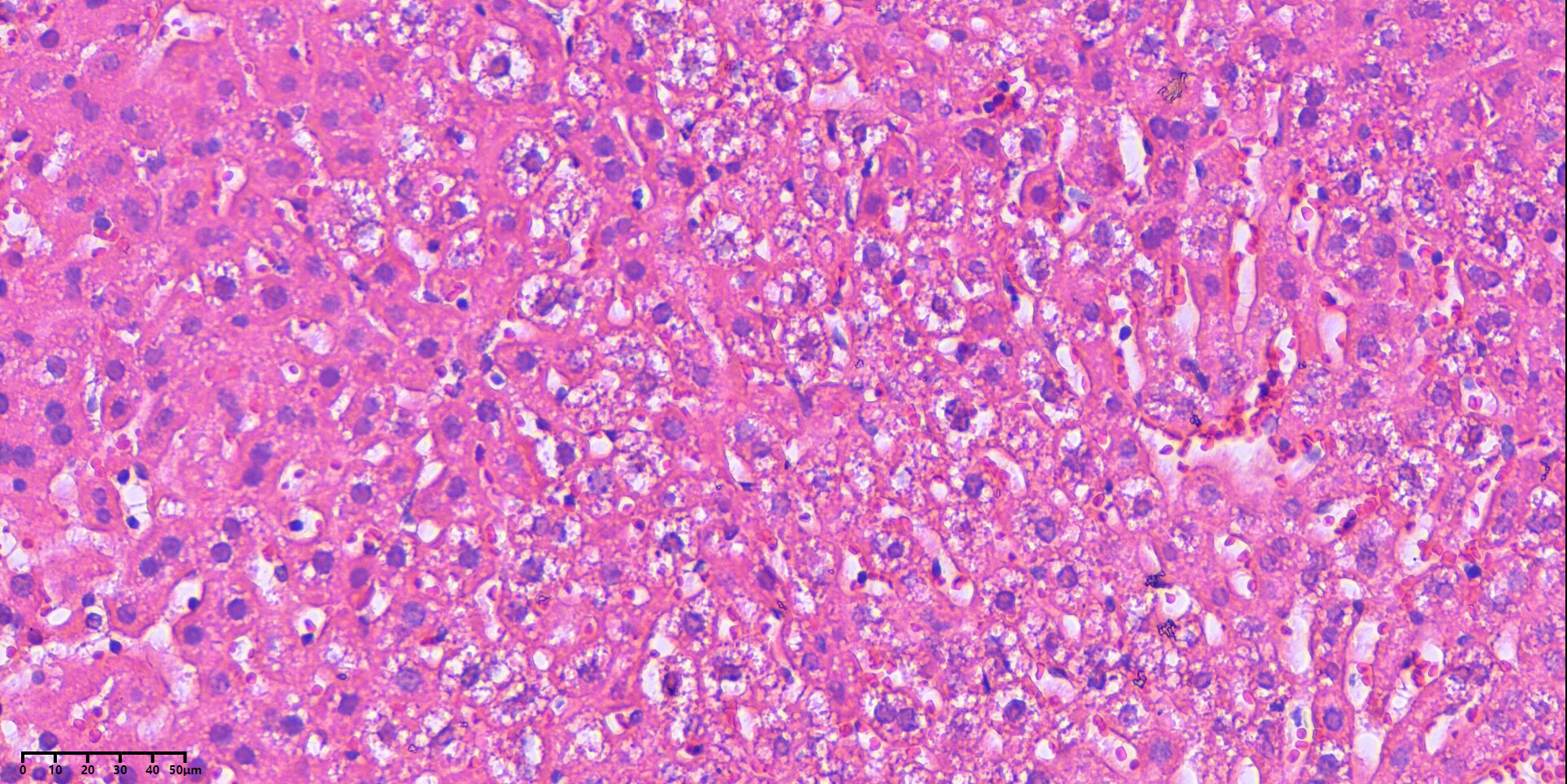

Supplement: Supplementary file 3 — Supplementary Information 3. [file 41598_2024_55043_MOESM3_ESM.zip › Supplementary material/HE/UCDA 50/zc7.jpg]

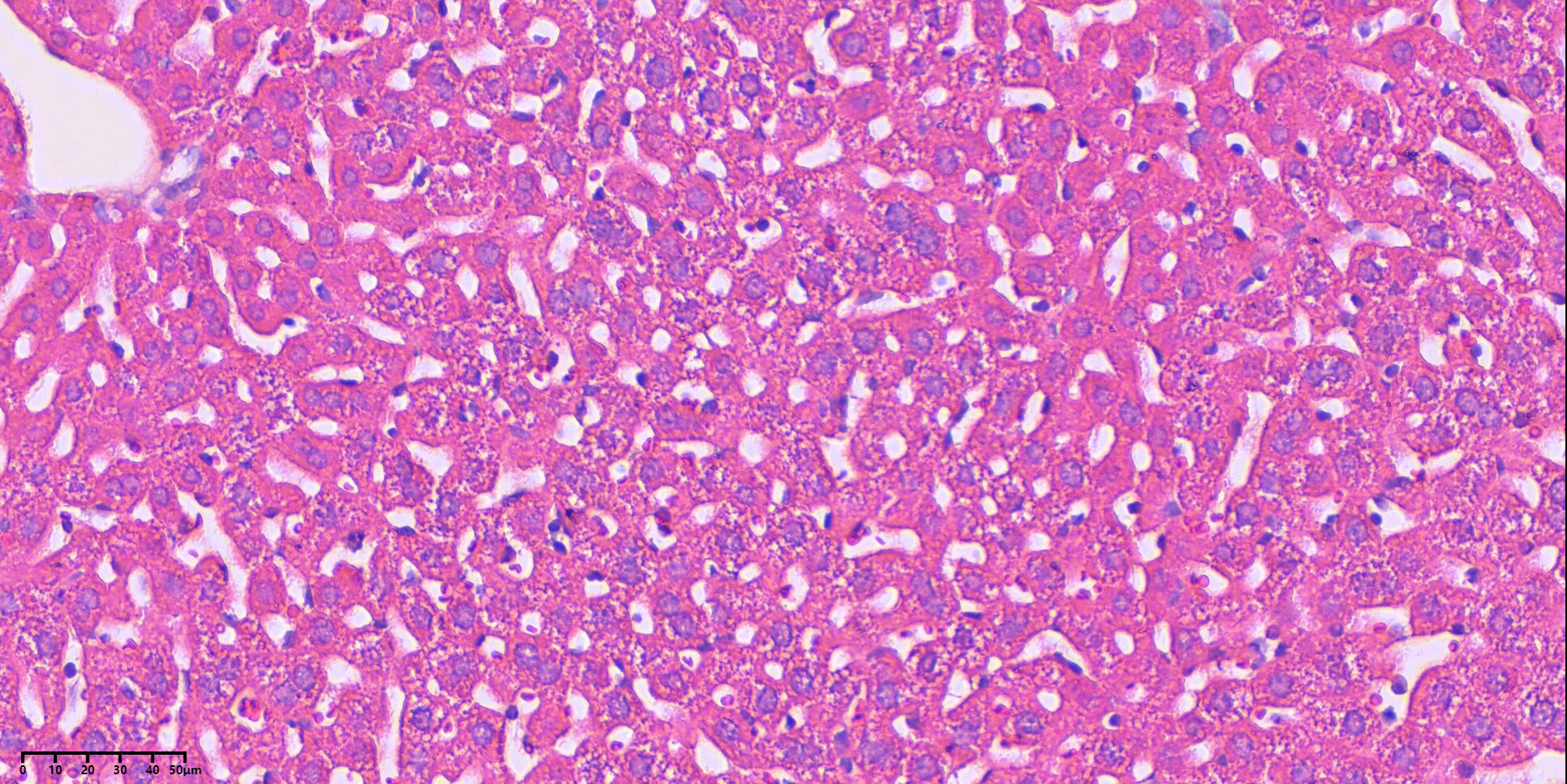

Supplement: Supplementary file 3 — Supplementary Information 3. [file 41598_2024_55043_MOESM3_ESM.zip › Supplementary material/HE/UCDA 50/zc10.jpg]

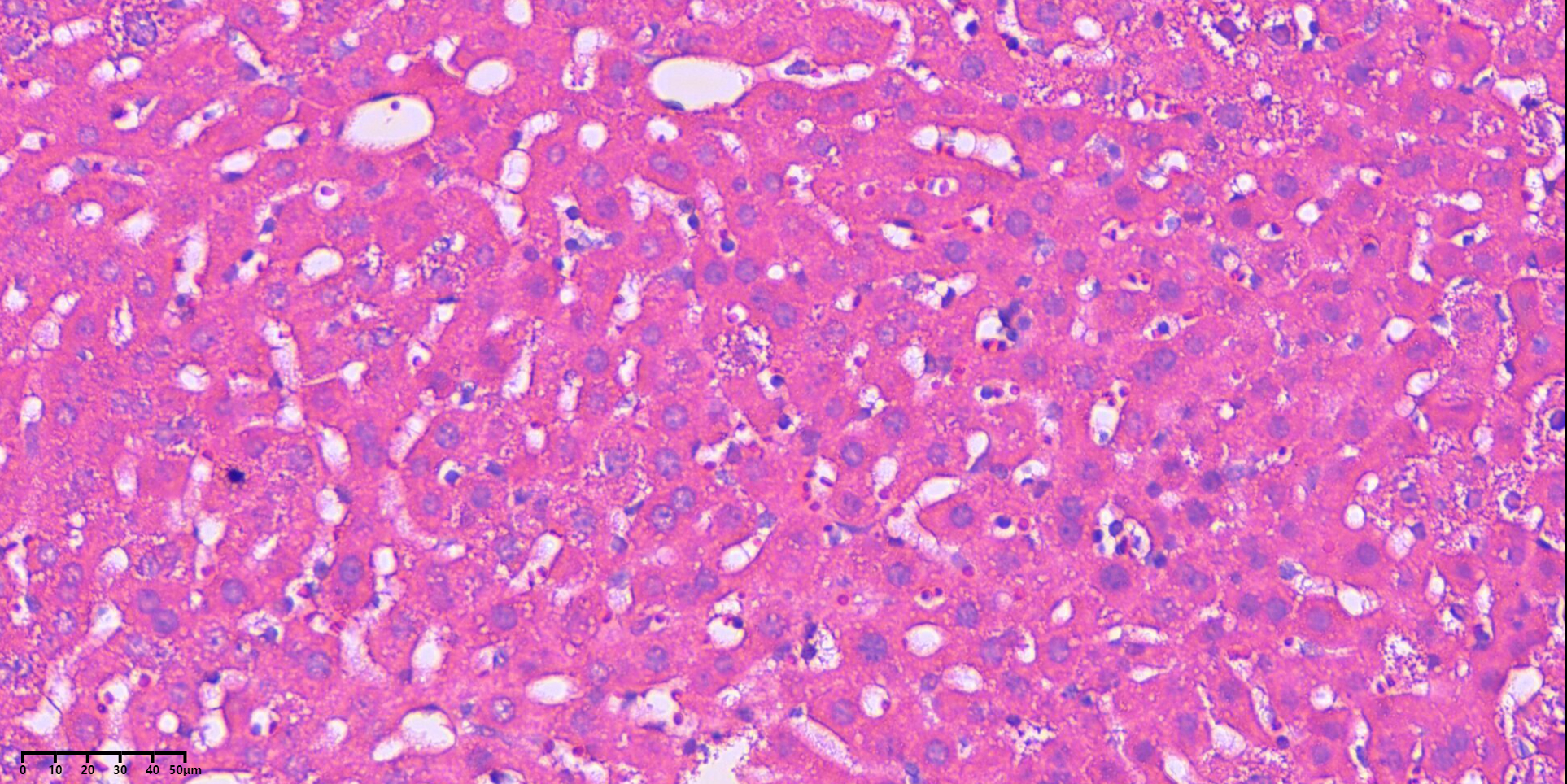

Supplement: Supplementary file 3 — Supplementary Information 3. [file 41598_2024_55043_MOESM3_ESM.zip › Supplementary material/HE/UCDA 50/zc9.jpg]

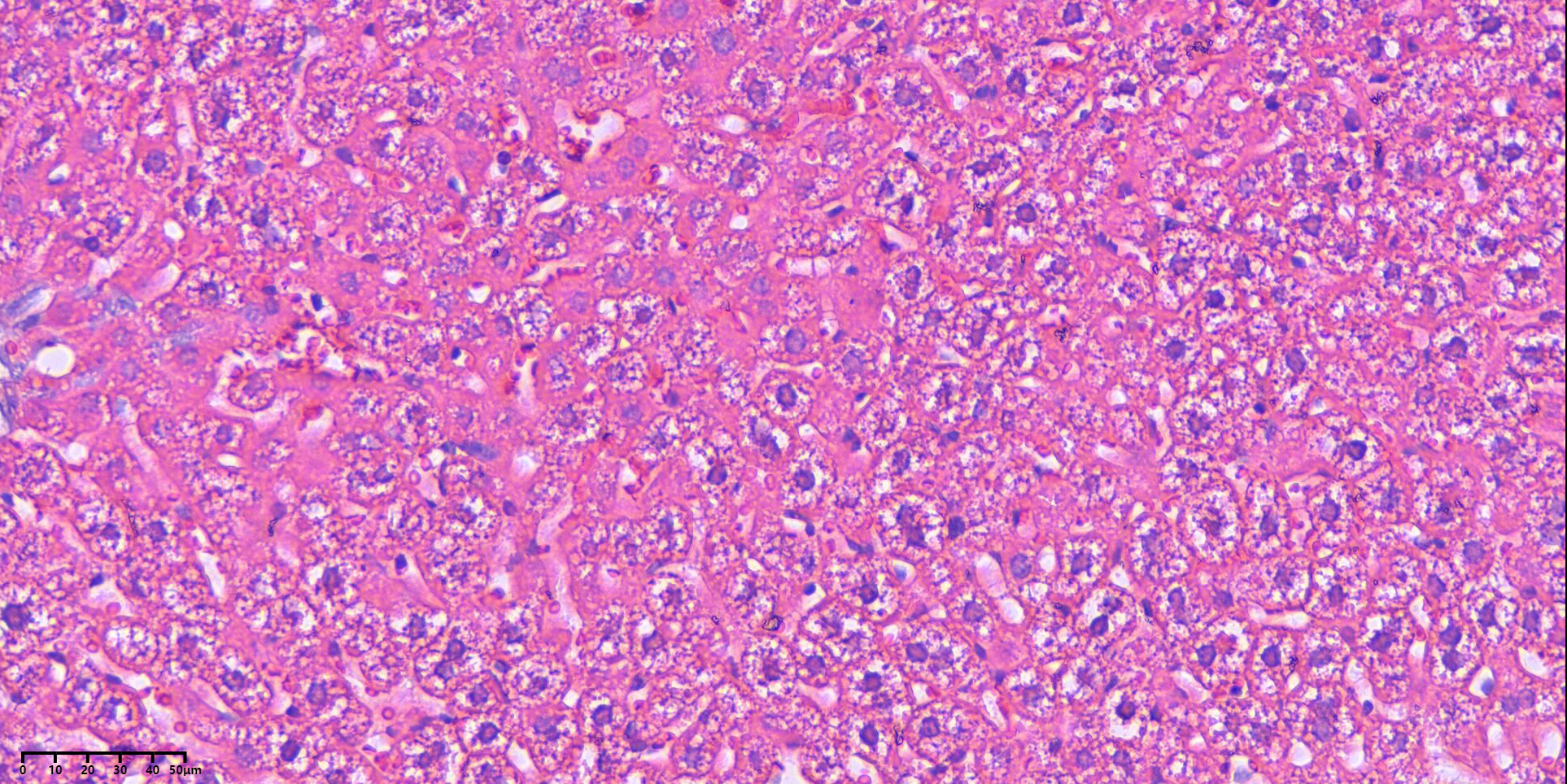

Supplement: Supplementary file 3 — Supplementary Information 3. [file 41598_2024_55043_MOESM3_ESM.zip › Supplementary material/HE/UCDA 50/zc8.jpg]

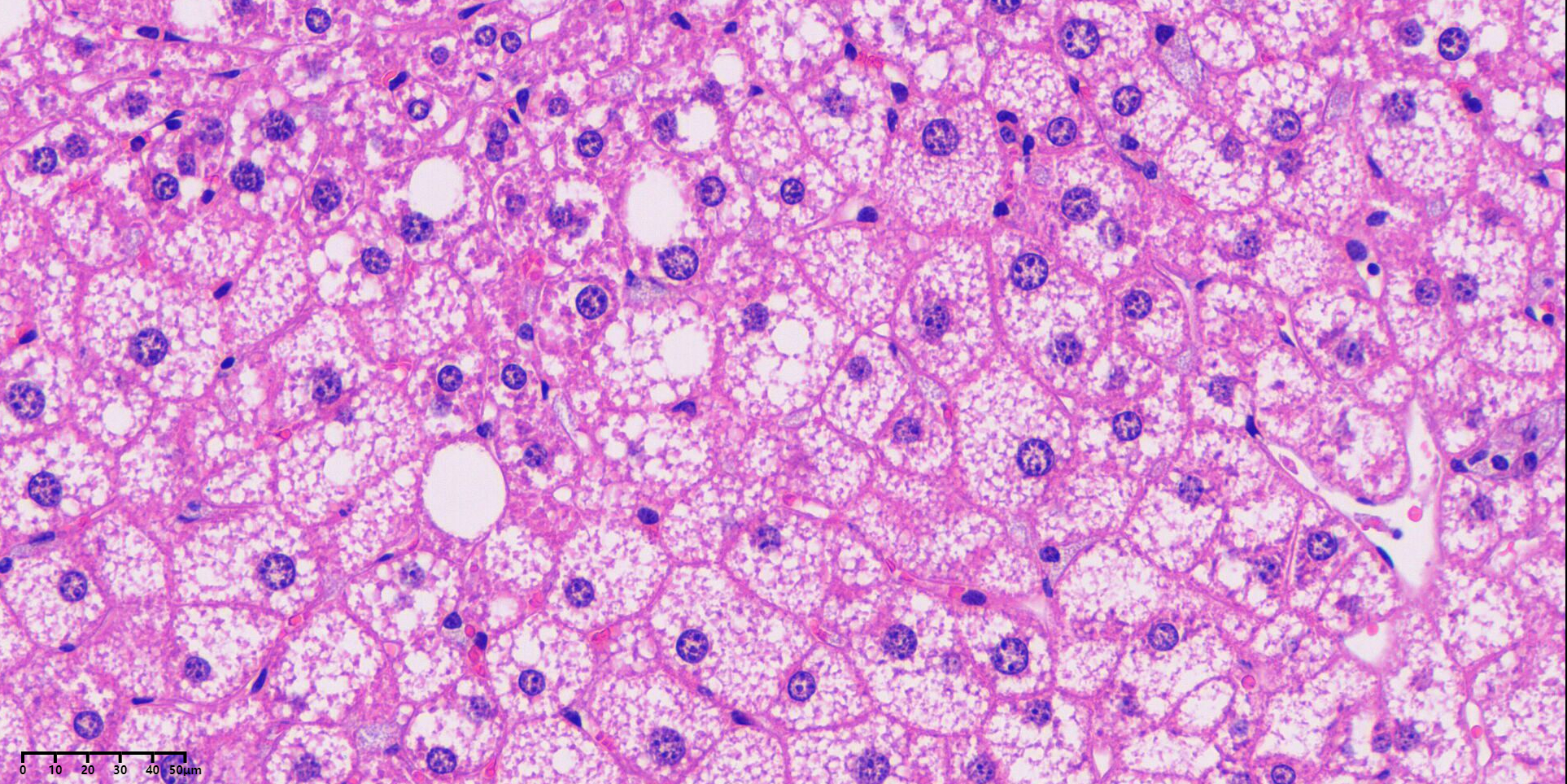

Supplement: Supplementary file 3 — Supplementary Information 3. [file 41598_2024_55043_MOESM3_ESM.zip › Supplementary material/HE/AS+UDCA 10/AS12.jpg]

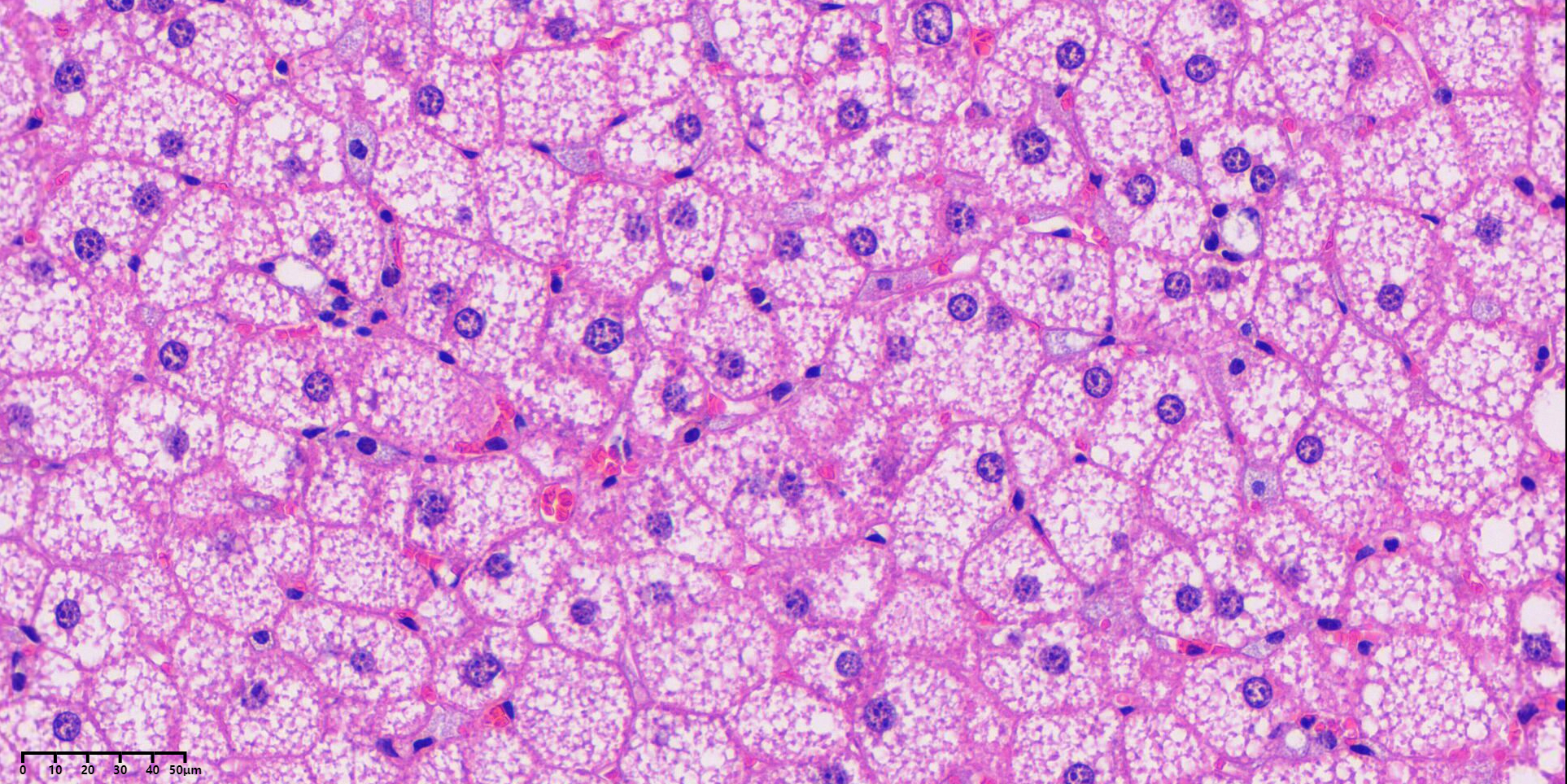

Supplement: Supplementary file 3 — Supplementary Information 3. [file 41598_2024_55043_MOESM3_ESM.zip › Supplementary material/HE/AS+UDCA 10/AS13.jpg]

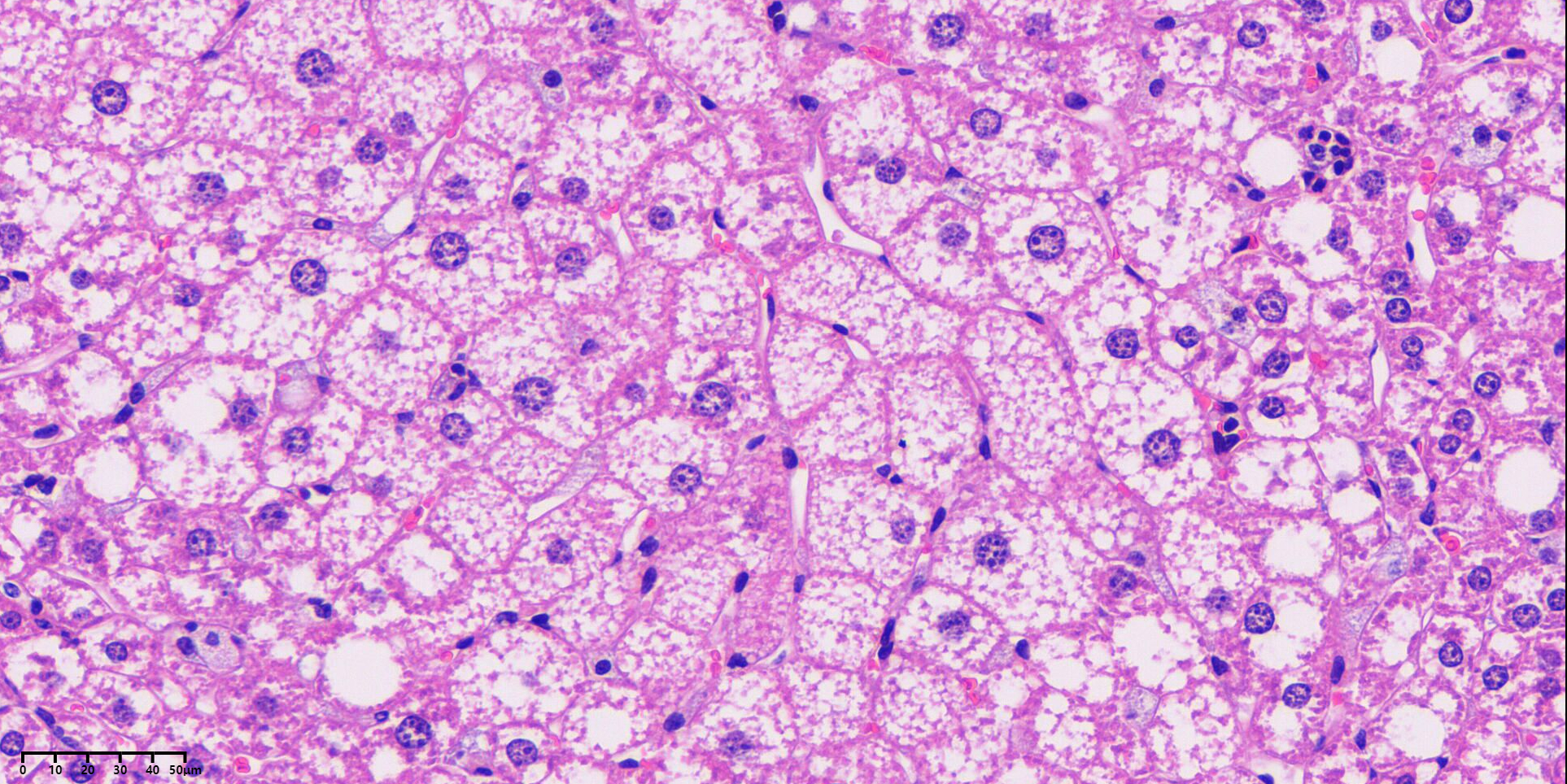

Supplement: Supplementary file 3 — Supplementary Information 3. [file 41598_2024_55043_MOESM3_ESM.zip › Supplementary material/HE/AS+UDCA 10/AS11.jpg]

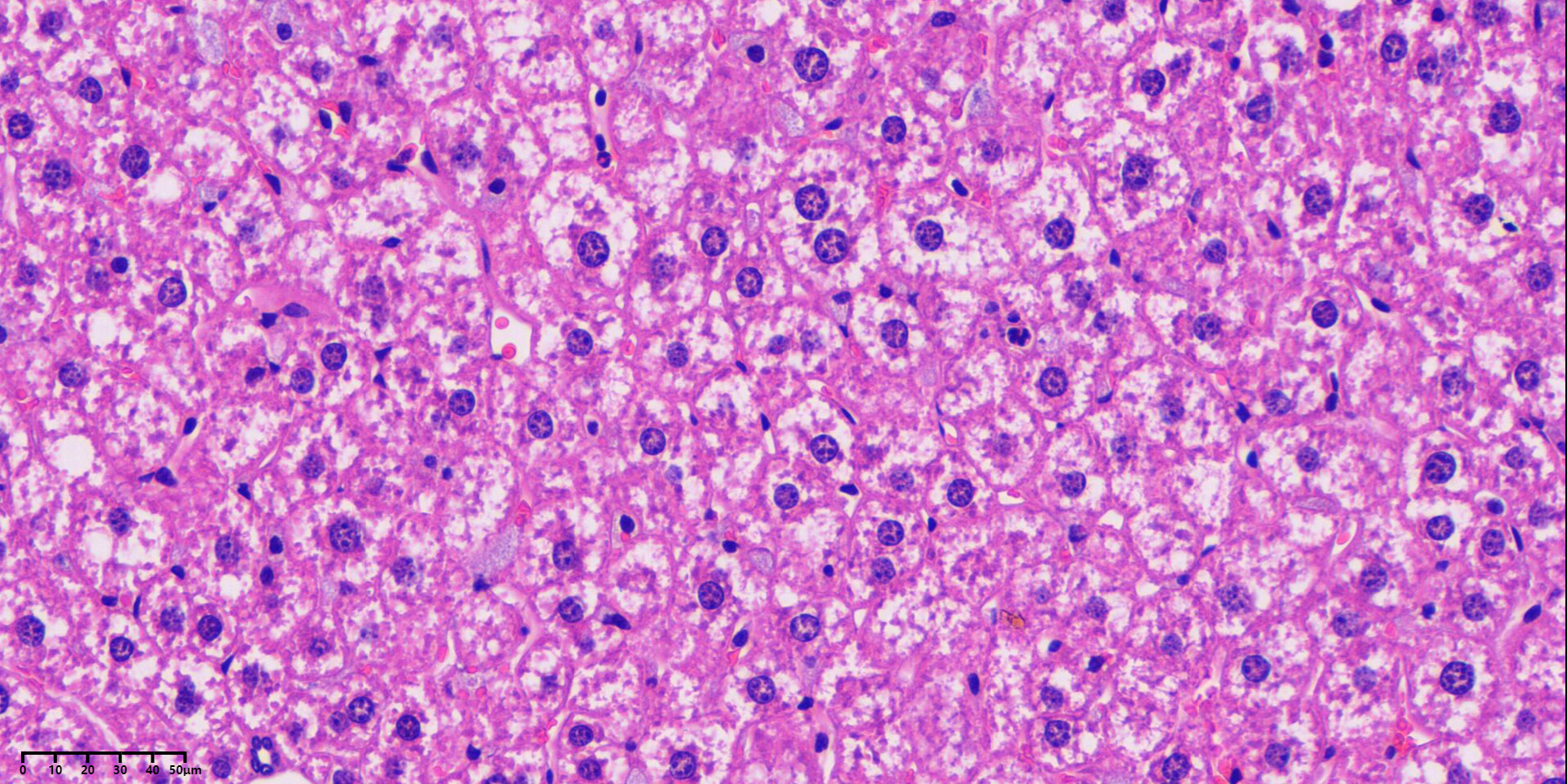

Supplement: Supplementary file 3 — Supplementary Information 3. [file 41598_2024_55043_MOESM3_ESM.zip › Supplementary material/HE/AS+UDCA 10/AS14.jpg]

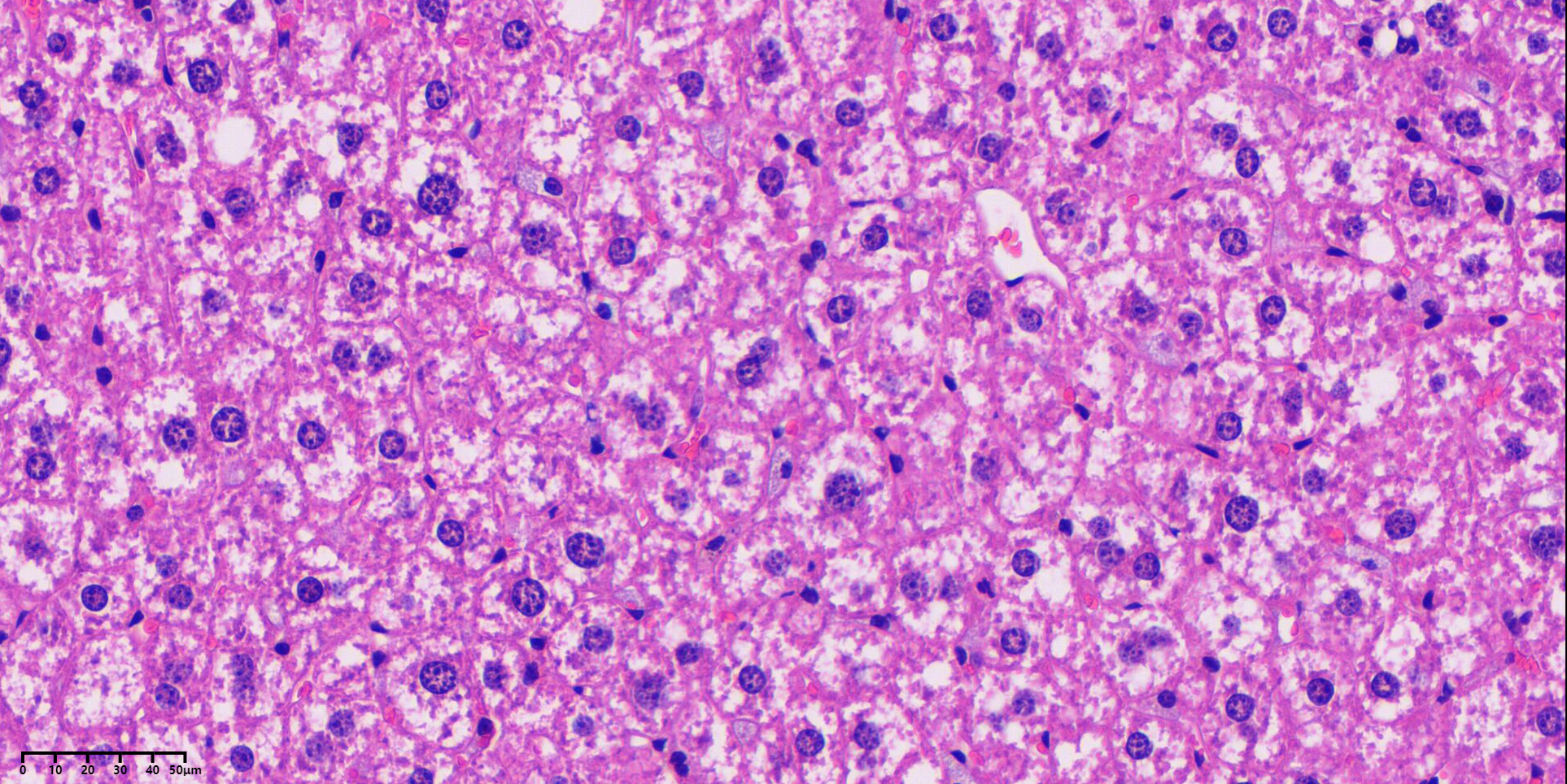

Supplement: Supplementary file 3 — Supplementary Information 3. [file 41598_2024_55043_MOESM3_ESM.zip › Supplementary material/HE/AS+UDCA 10/AS15.jpg]

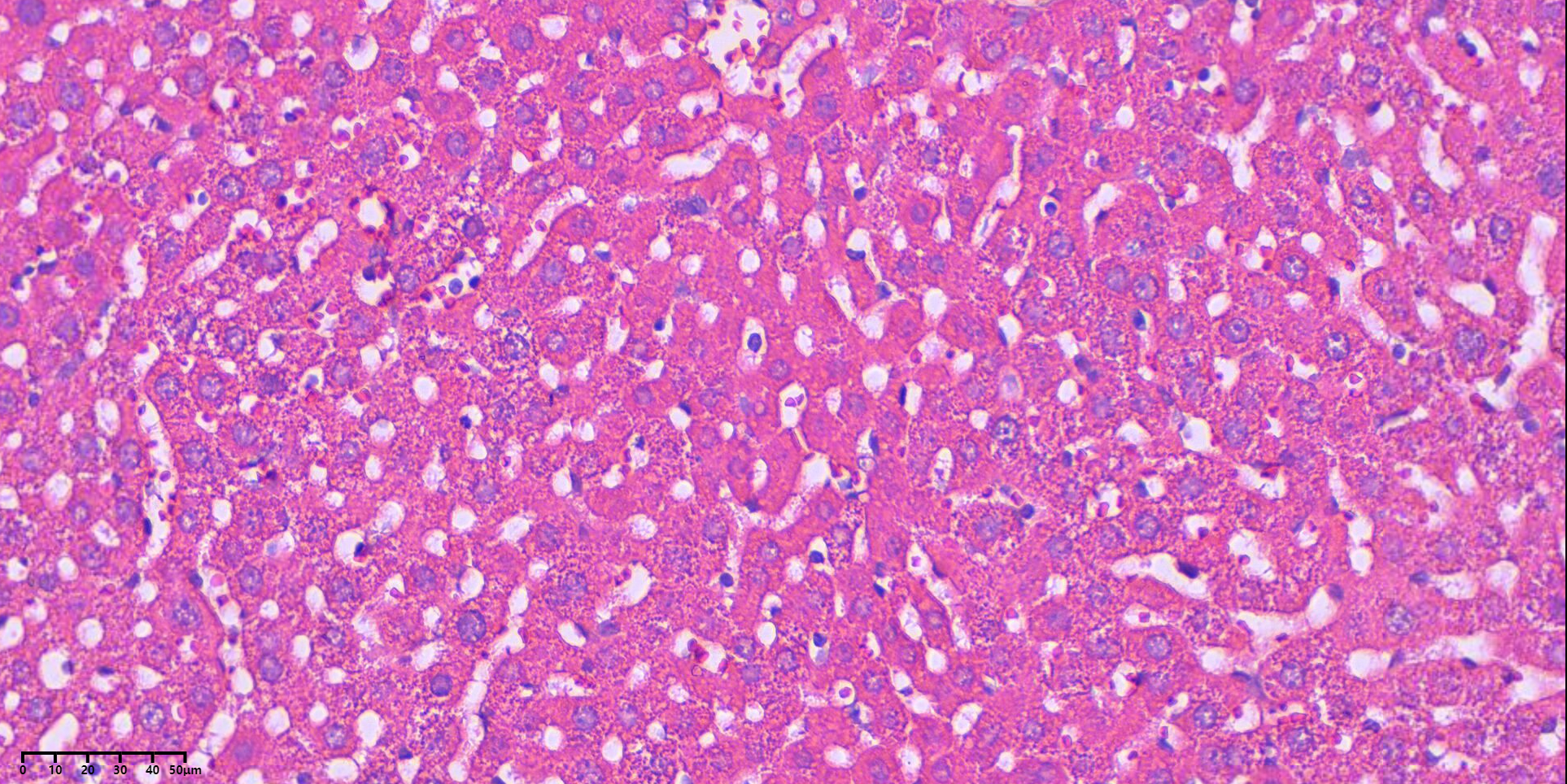

Supplement: Supplementary file 3 — Supplementary Information 3. [file 41598_2024_55043_MOESM3_ESM.zip › Supplementary material/HE/Control/zc15.jpg]

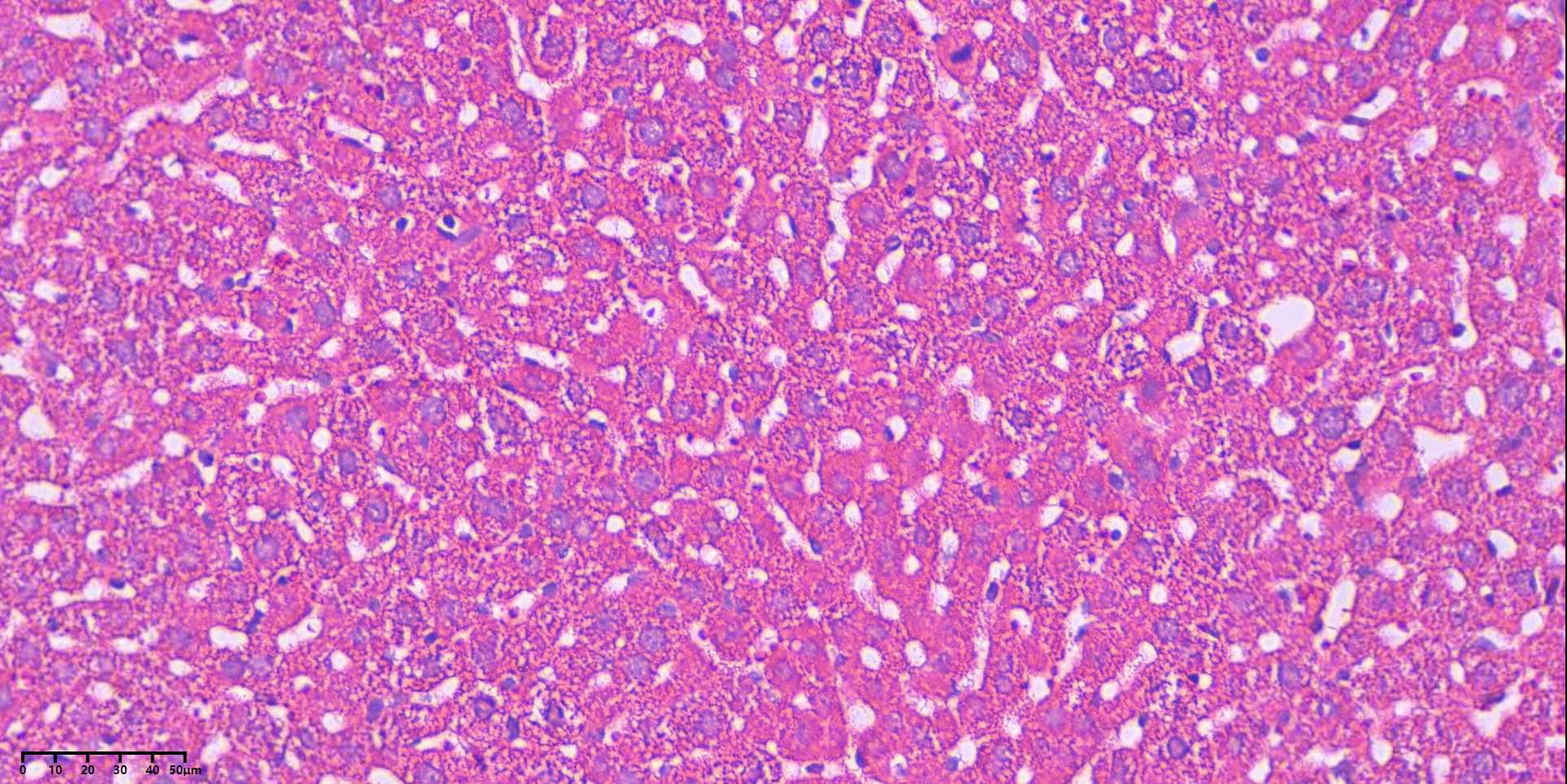

Supplement: Supplementary file 3 — Supplementary Information 3. [file 41598_2024_55043_MOESM3_ESM.zip › Supplementary material/HE/Control/zc14.jpg]

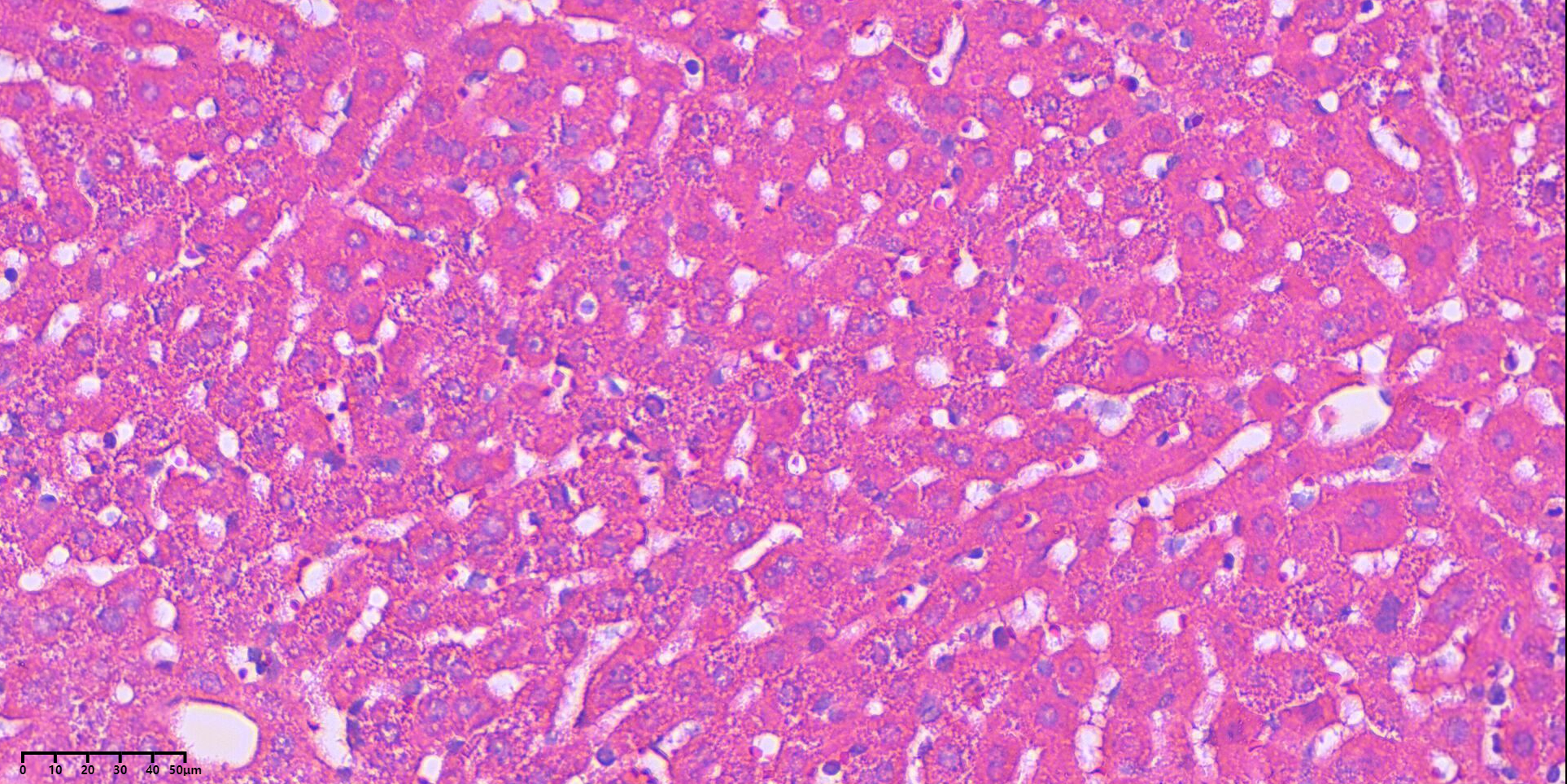

Supplement: Supplementary file 3 — Supplementary Information 3. [file 41598_2024_55043_MOESM3_ESM.zip › Supplementary material/HE/Control/zc13.jpg]

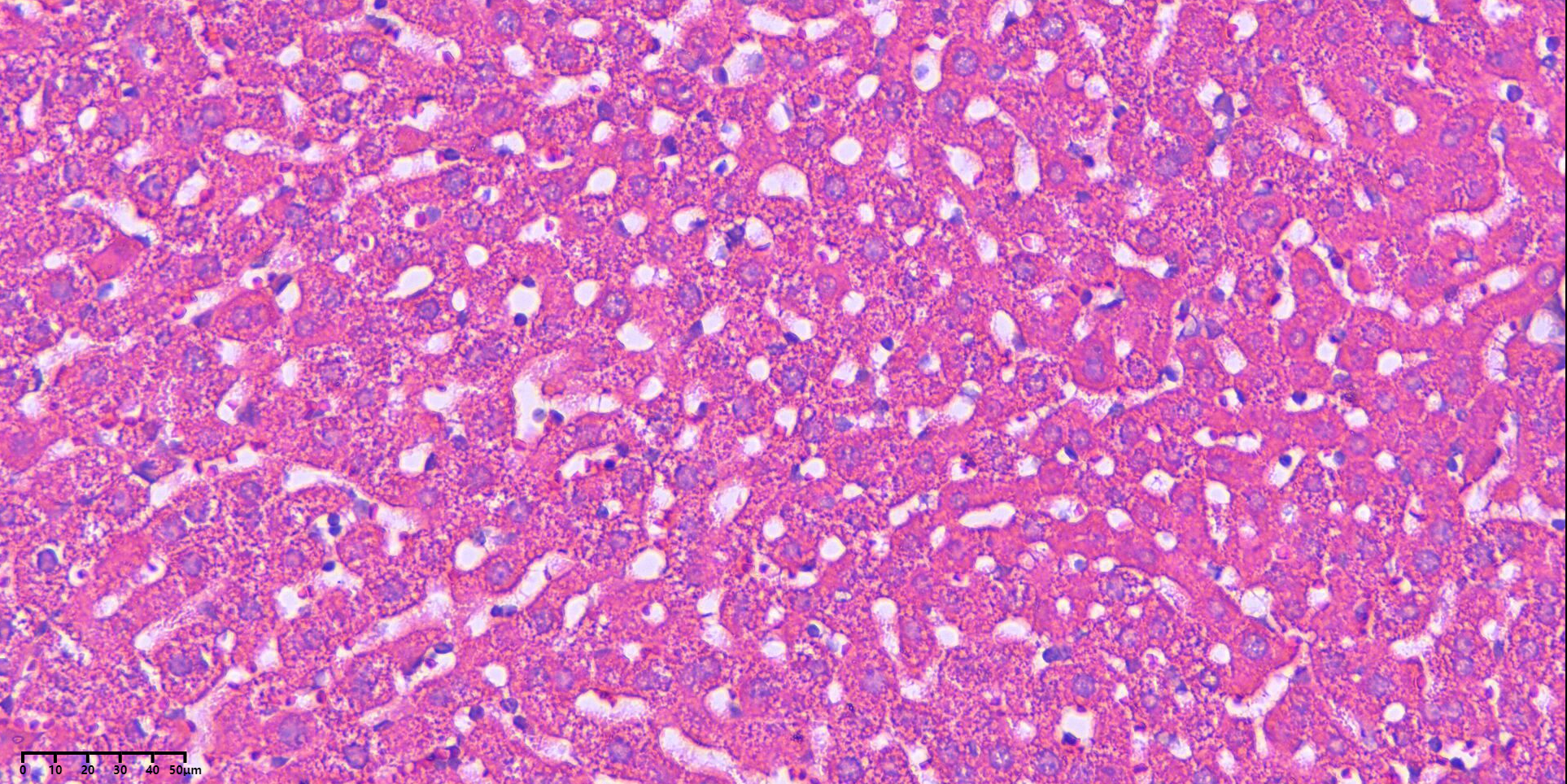

Supplement: Supplementary file 3 — Supplementary Information 3. [file 41598_2024_55043_MOESM3_ESM.zip › Supplementary material/HE/Control/zc12.jpg]

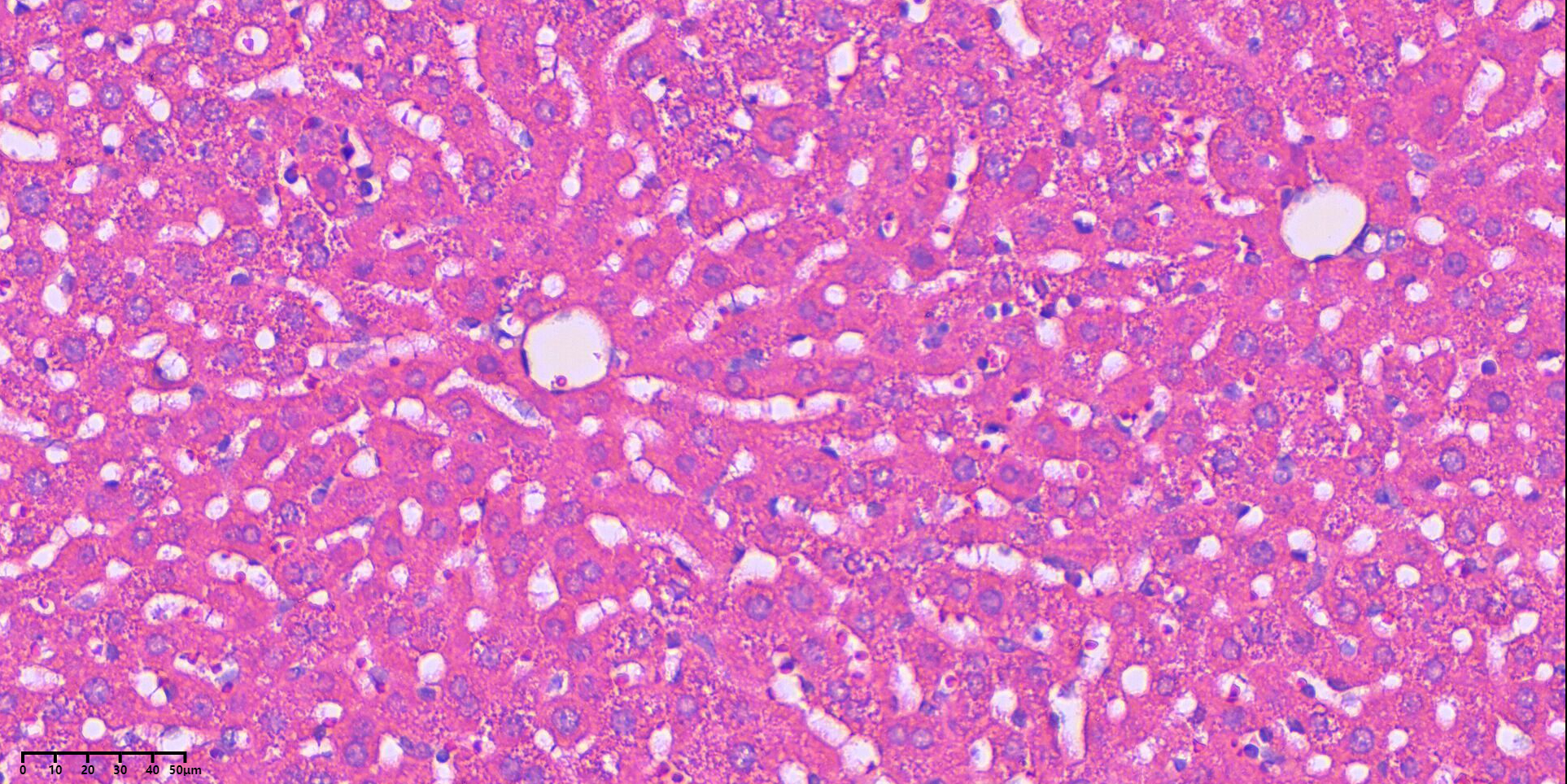

Supplement: Supplementary file 3 — Supplementary Information 3. [file 41598_2024_55043_MOESM3_ESM.zip › Supplementary material/HE/Control/zc11.jpg]

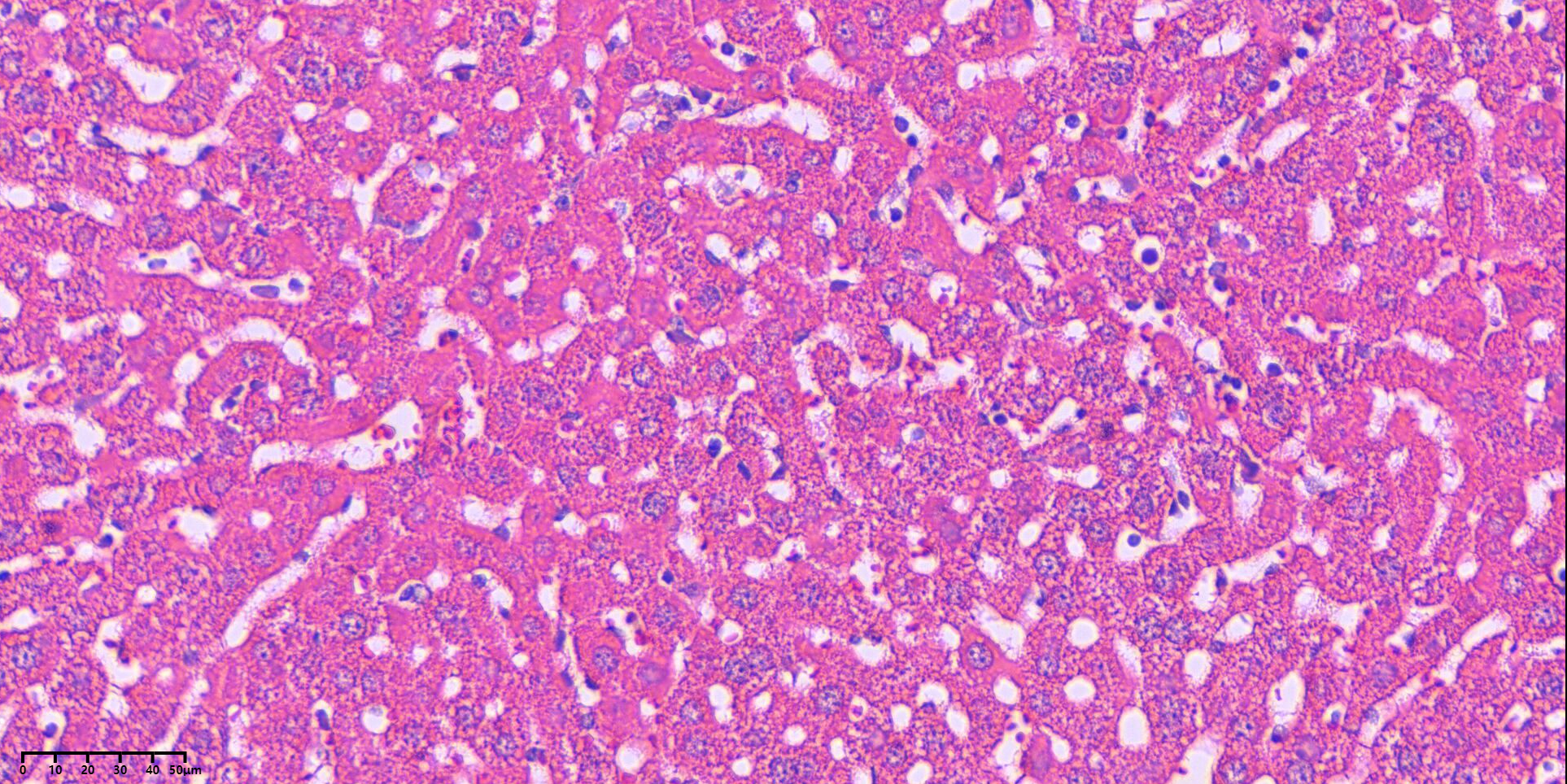

Supplement: Supplementary file 3 — Supplementary Information 3. [file 41598_2024_55043_MOESM3_ESM.zip › Supplementary material/HE/UDCA 10/zc20.jpg]

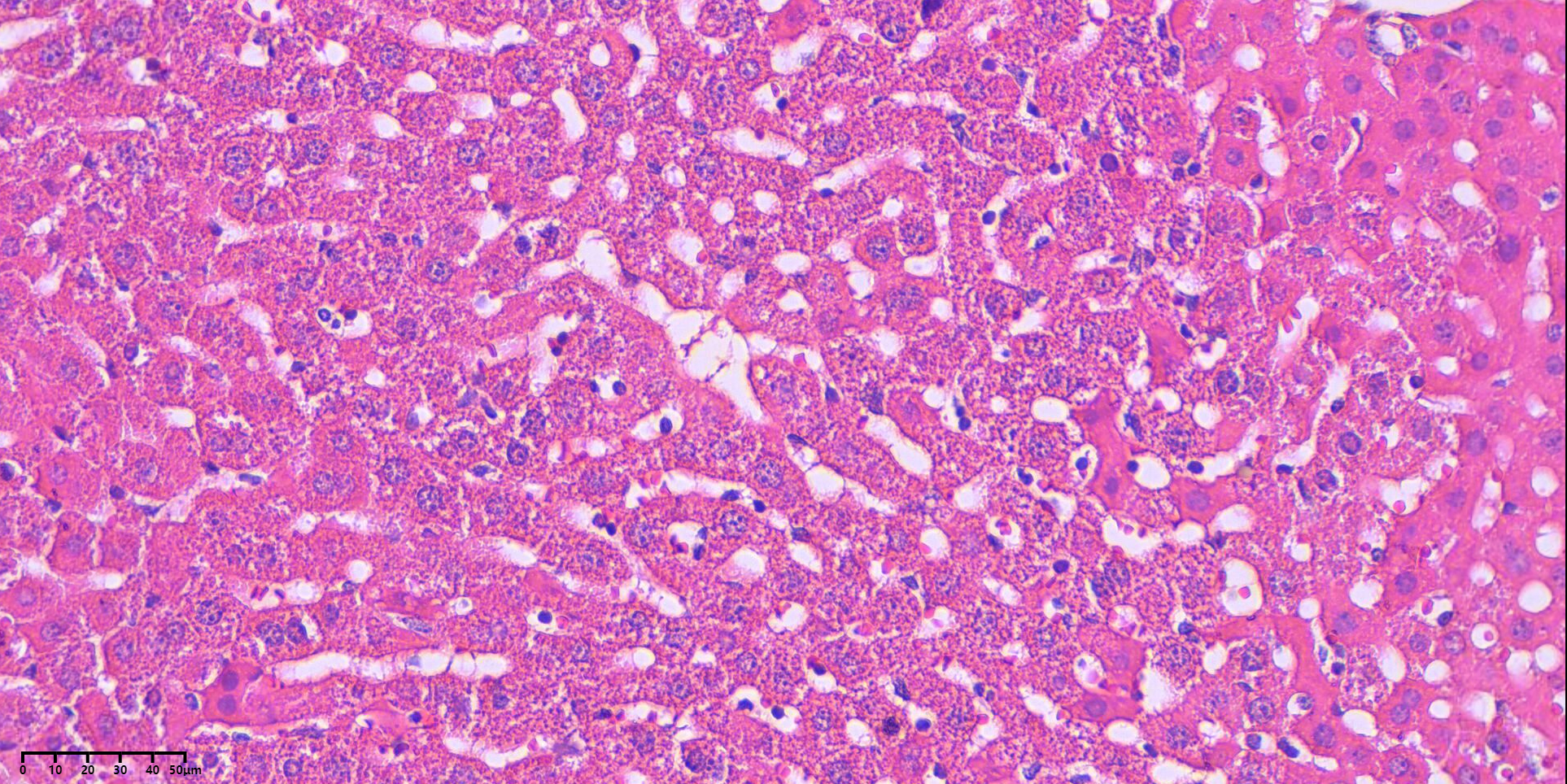

Supplement: Supplementary file 3 — Supplementary Information 3. [file 41598_2024_55043_MOESM3_ESM.zip › Supplementary material/HE/UDCA 10/zc21.jpg]

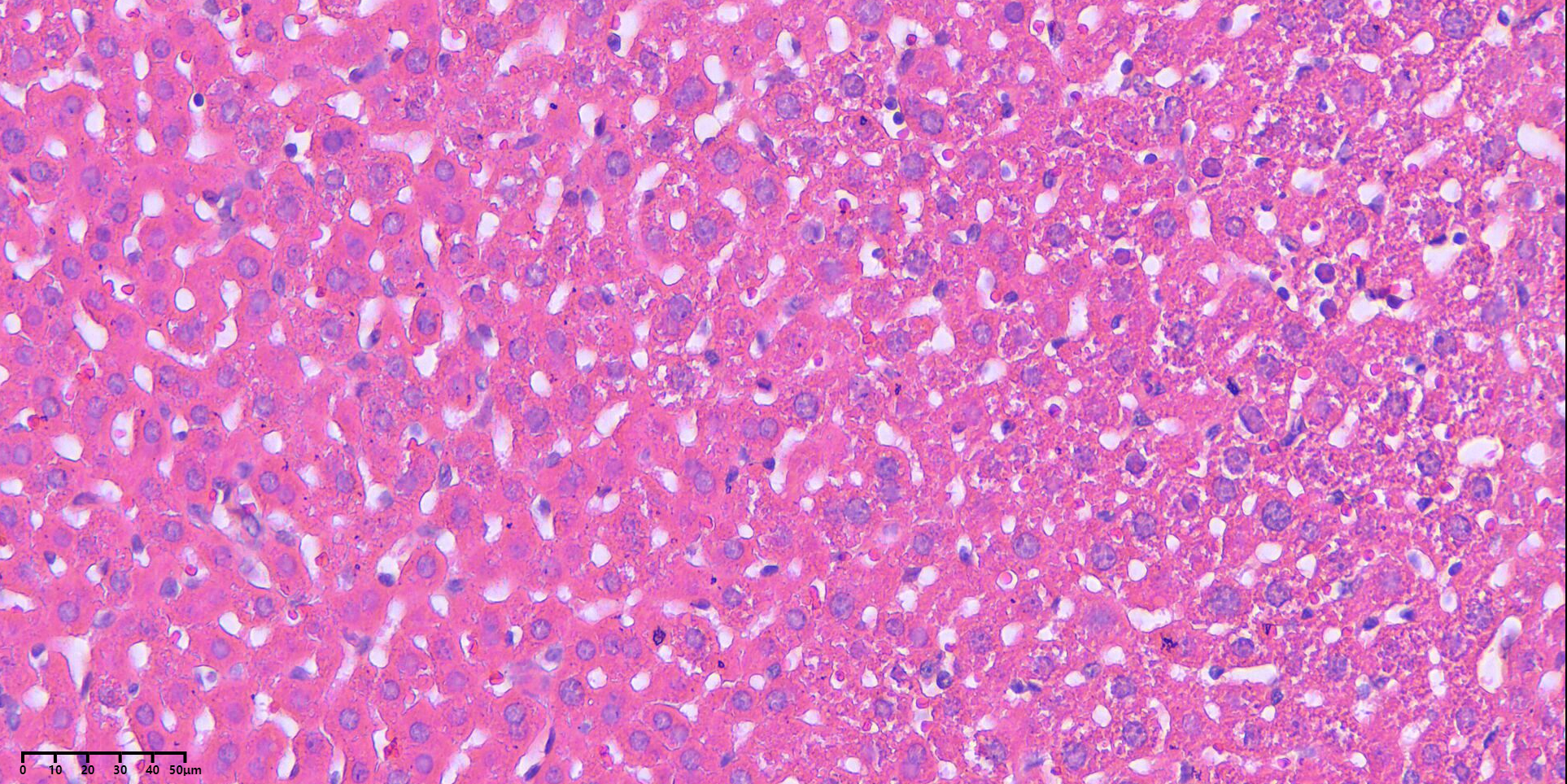

Supplement: Supplementary file 3 — Supplementary Information 3. [file 41598_2024_55043_MOESM3_ESM.zip › Supplementary material/HE/UDCA 10/zc19.jpg]

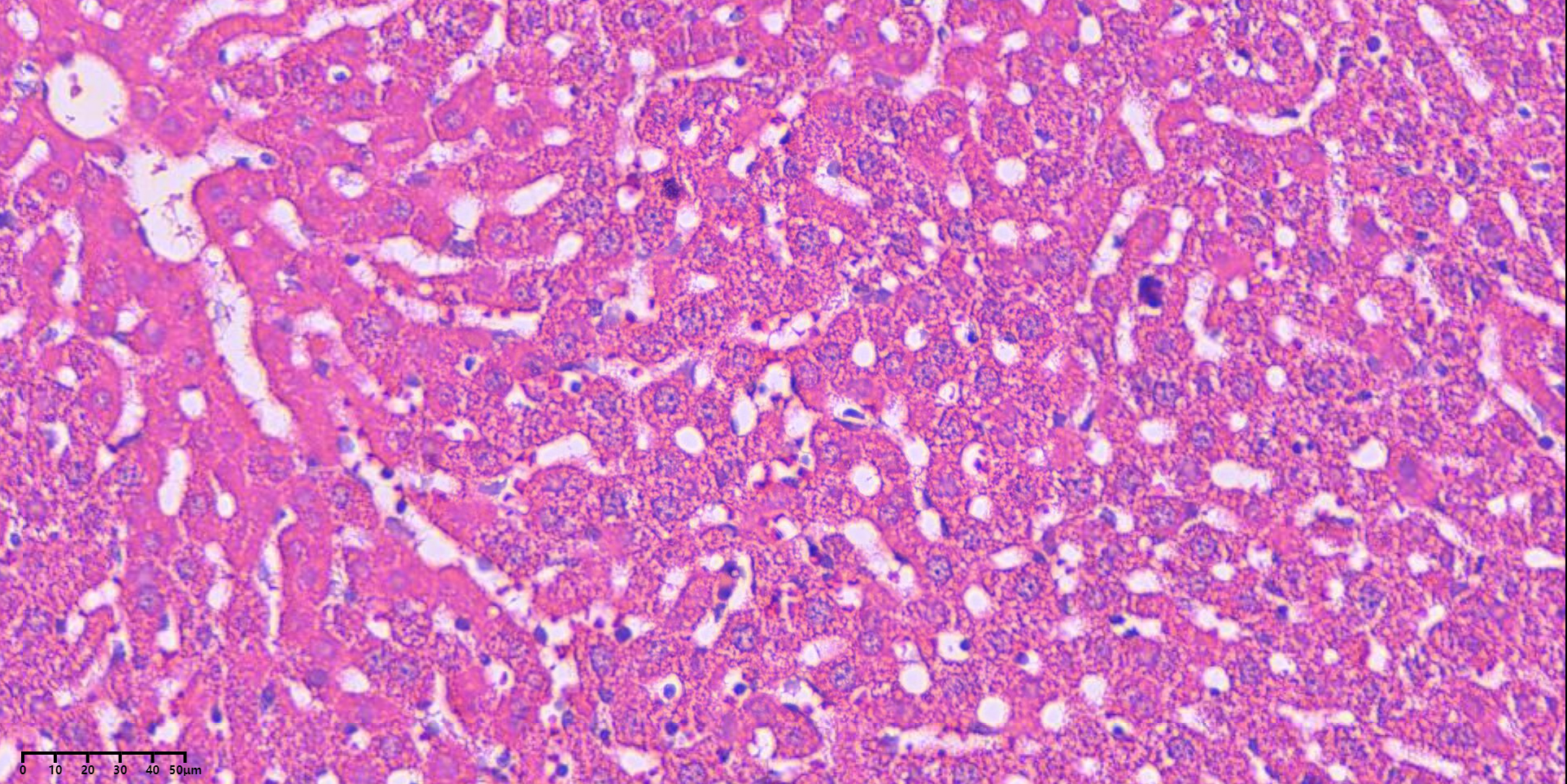

Supplement: Supplementary file 3 — Supplementary Information 3. [file 41598_2024_55043_MOESM3_ESM.zip › Supplementary material/HE/UDCA 10/zc18.jpg]

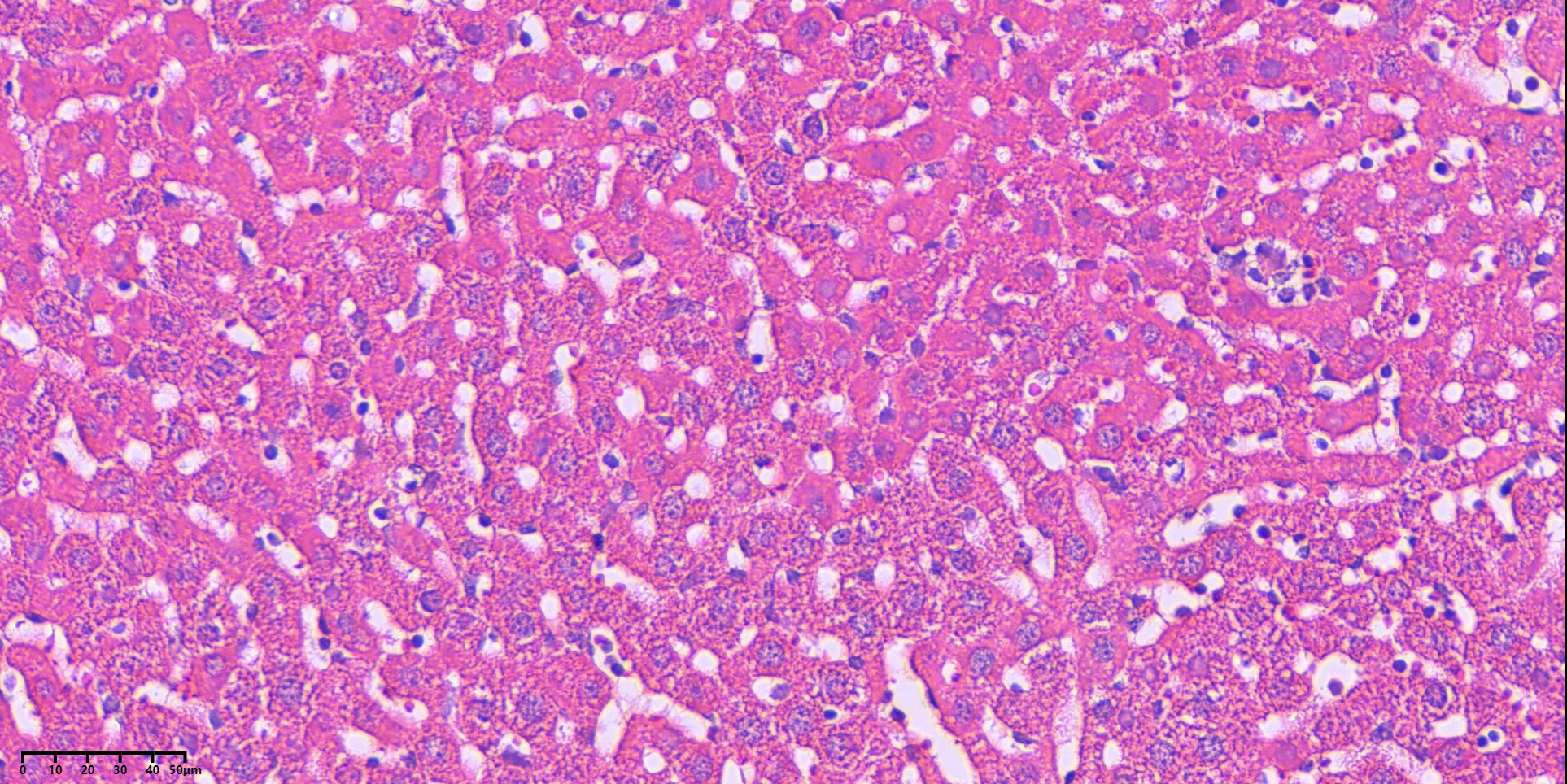

Supplement: Supplementary file 3 — Supplementary Information 3. [file 41598_2024_55043_MOESM3_ESM.zip › Supplementary material/HE/UDCA 10/zc16.jpg]

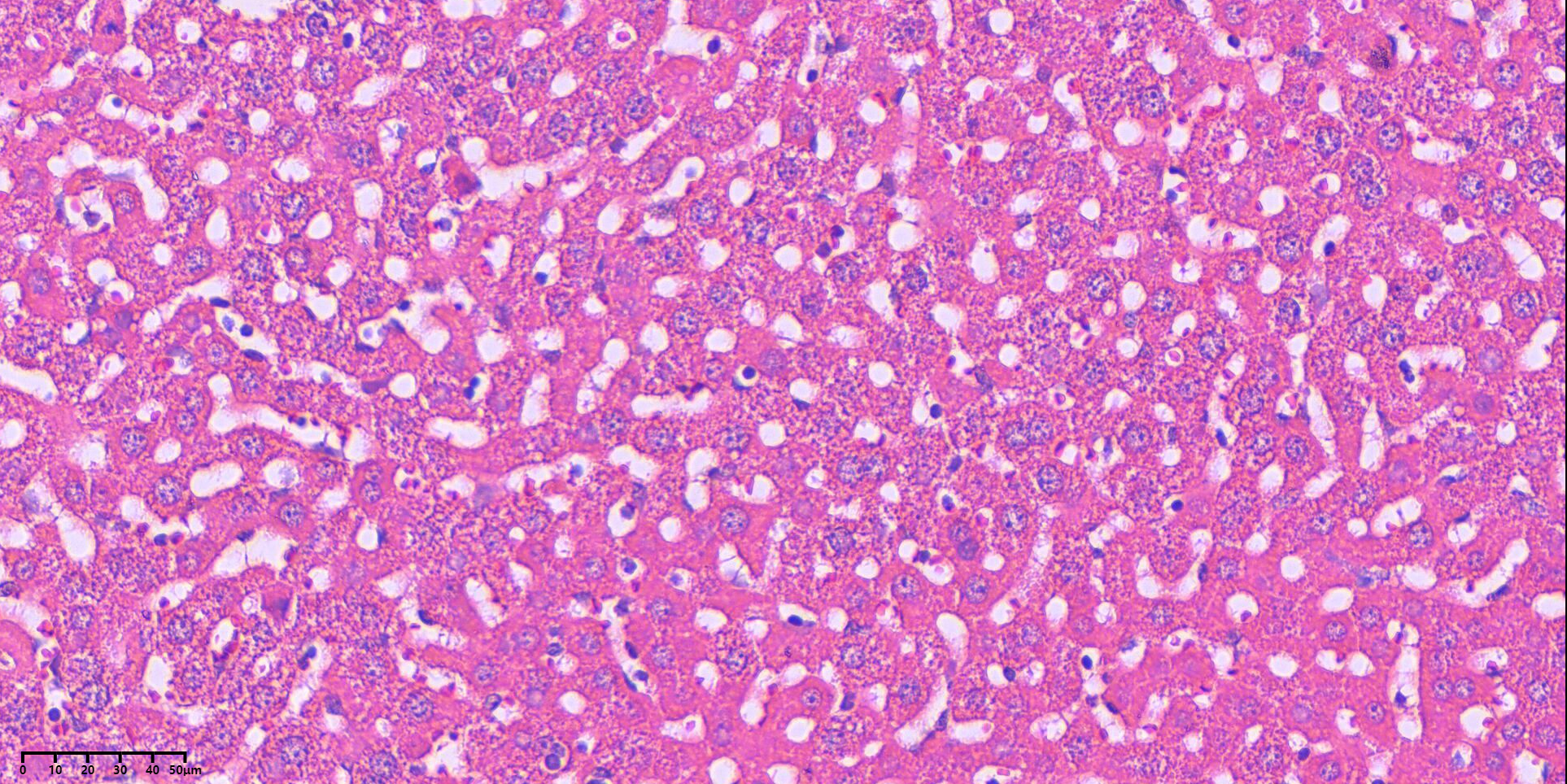

Supplement: Supplementary file 3 — Supplementary Information 3. [file 41598_2024_55043_MOESM3_ESM.zip › Supplementary material/HE/UDCA 10/zc17.jpg]

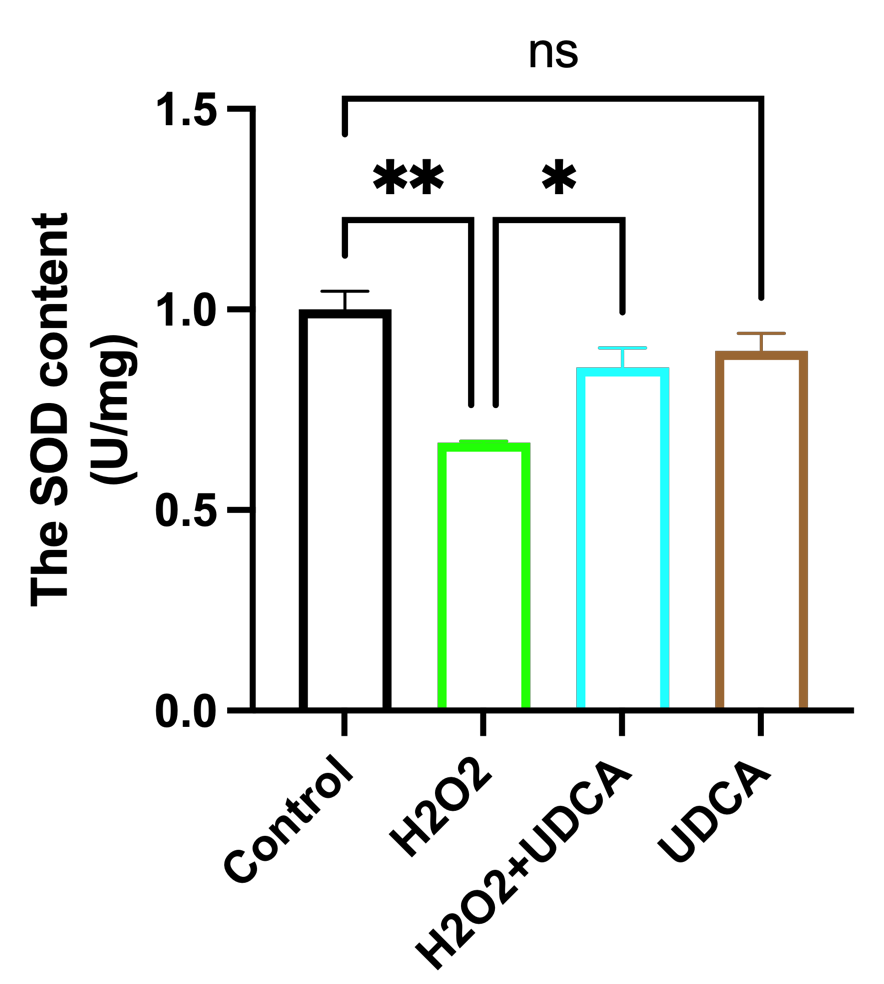

Supplement: Supplementary file 3 — Supplementary Information 3. [file 41598_2024_55043_MOESM3_ESM.zip › Supplementary material/Fig 2/SOD/SOD.tiff]

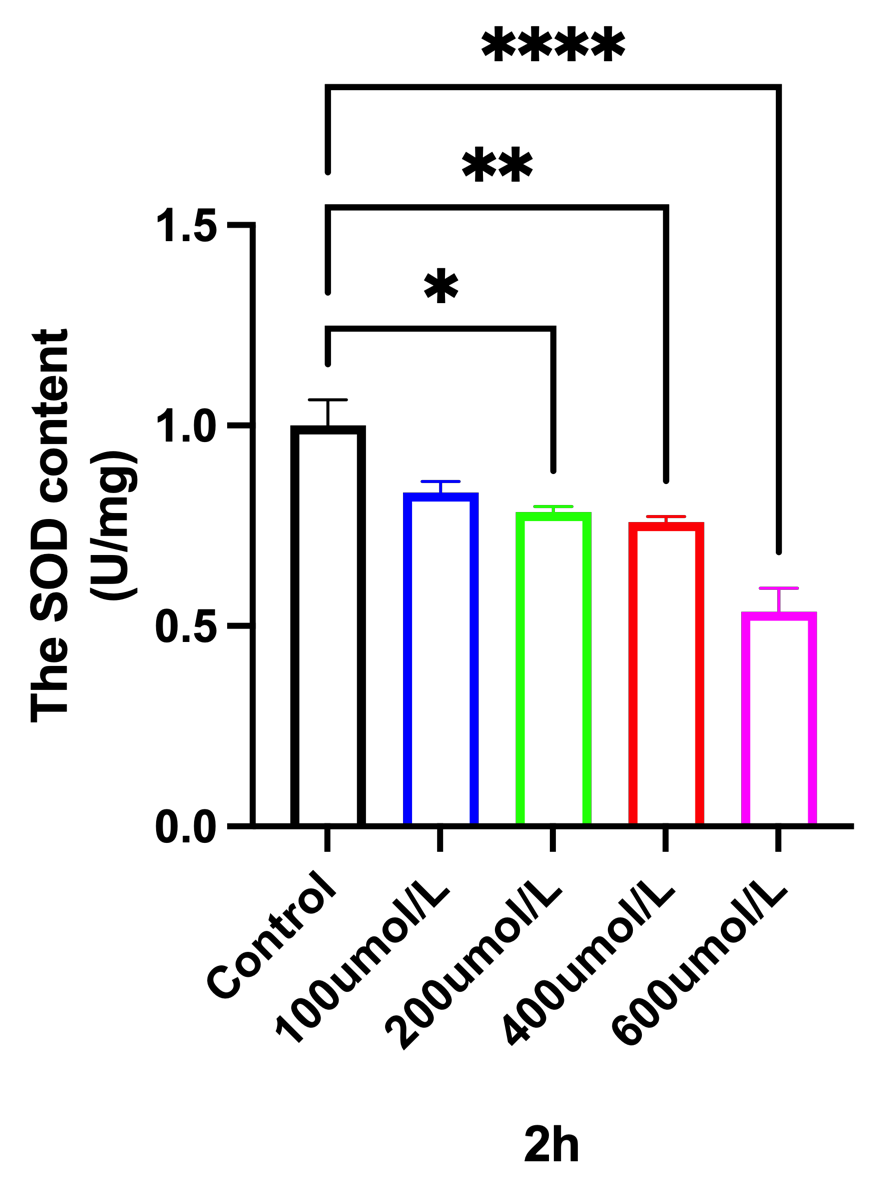

Supplement: Supplementary file 3 — Supplementary Information 3. [file 41598_2024_55043_MOESM3_ESM.zip › Supplementary material/Fig 2/SOD/μ╡ôσ║a.tiff]

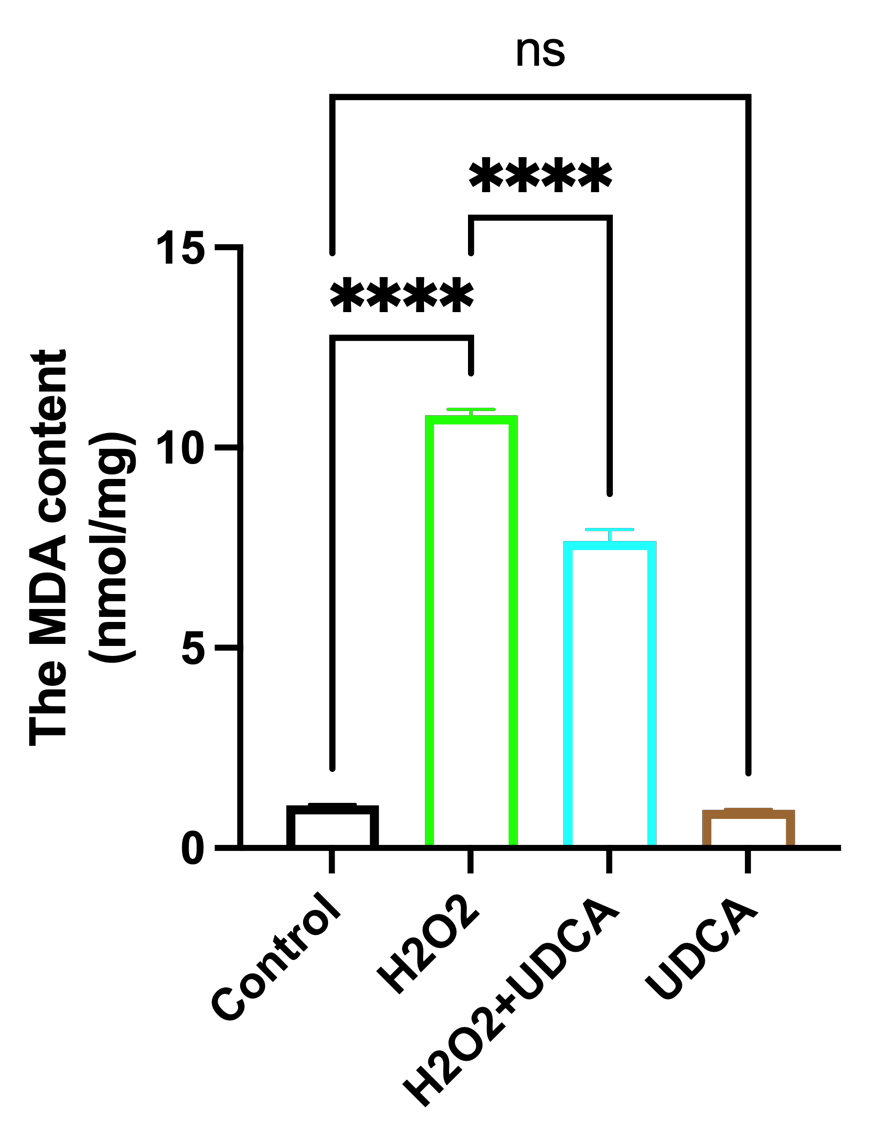

Supplement: Supplementary file 3 — Supplementary Information 3. [file 41598_2024_55043_MOESM3_ESM.zip › Supplementary material/Fig 2/MDA/MDA.tiff]

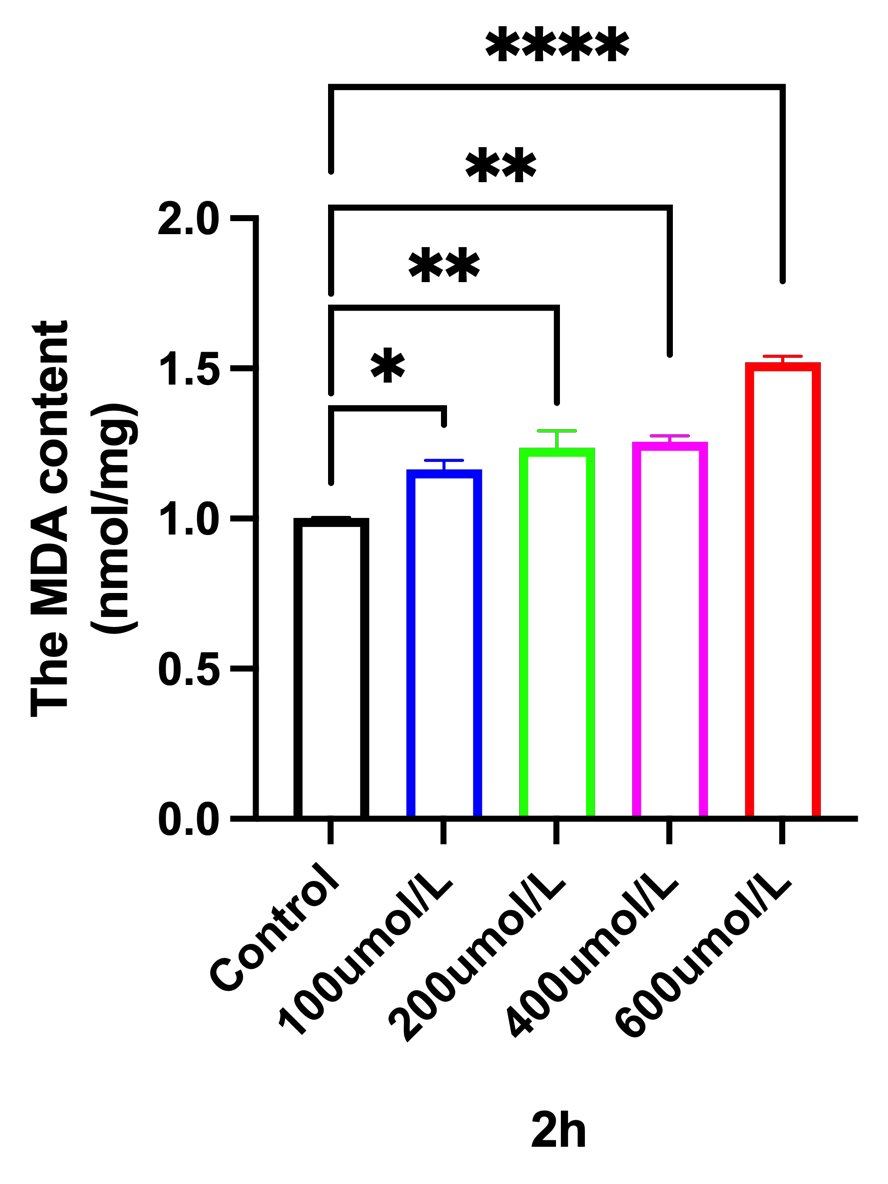

Supplement: Supplementary file 3 — Supplementary Information 3. [file 41598_2024_55043_MOESM3_ESM.zip › Supplementary material/Fig 2/MDA/μ╡ôσ║a.tiff]

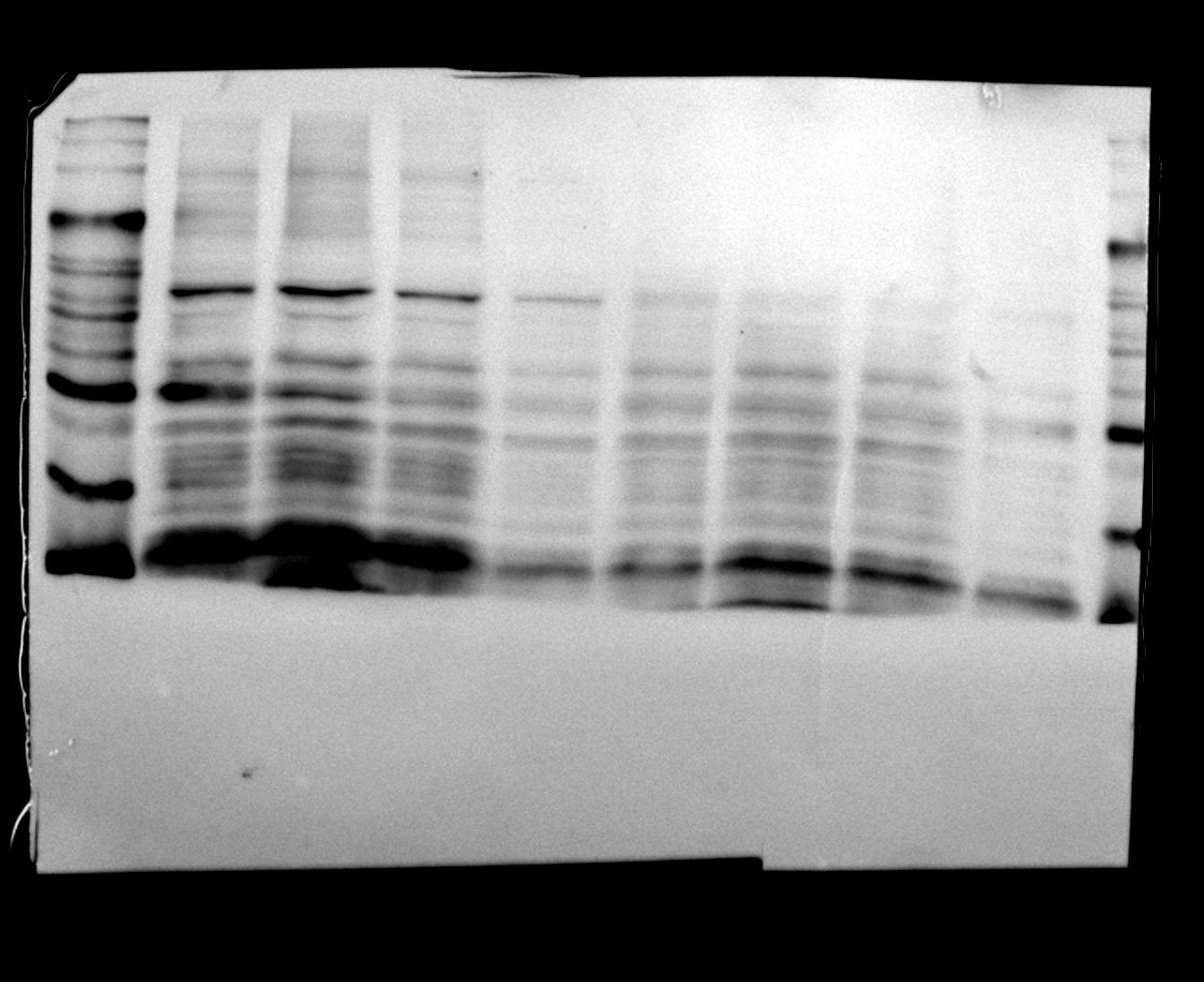

Supplement: Supplementary file 3 — Supplementary Information 3. [file 41598_2024_55043_MOESM3_ESM.zip › Supplementary material/Lo2-WB/IL-1B/1B.Tif]

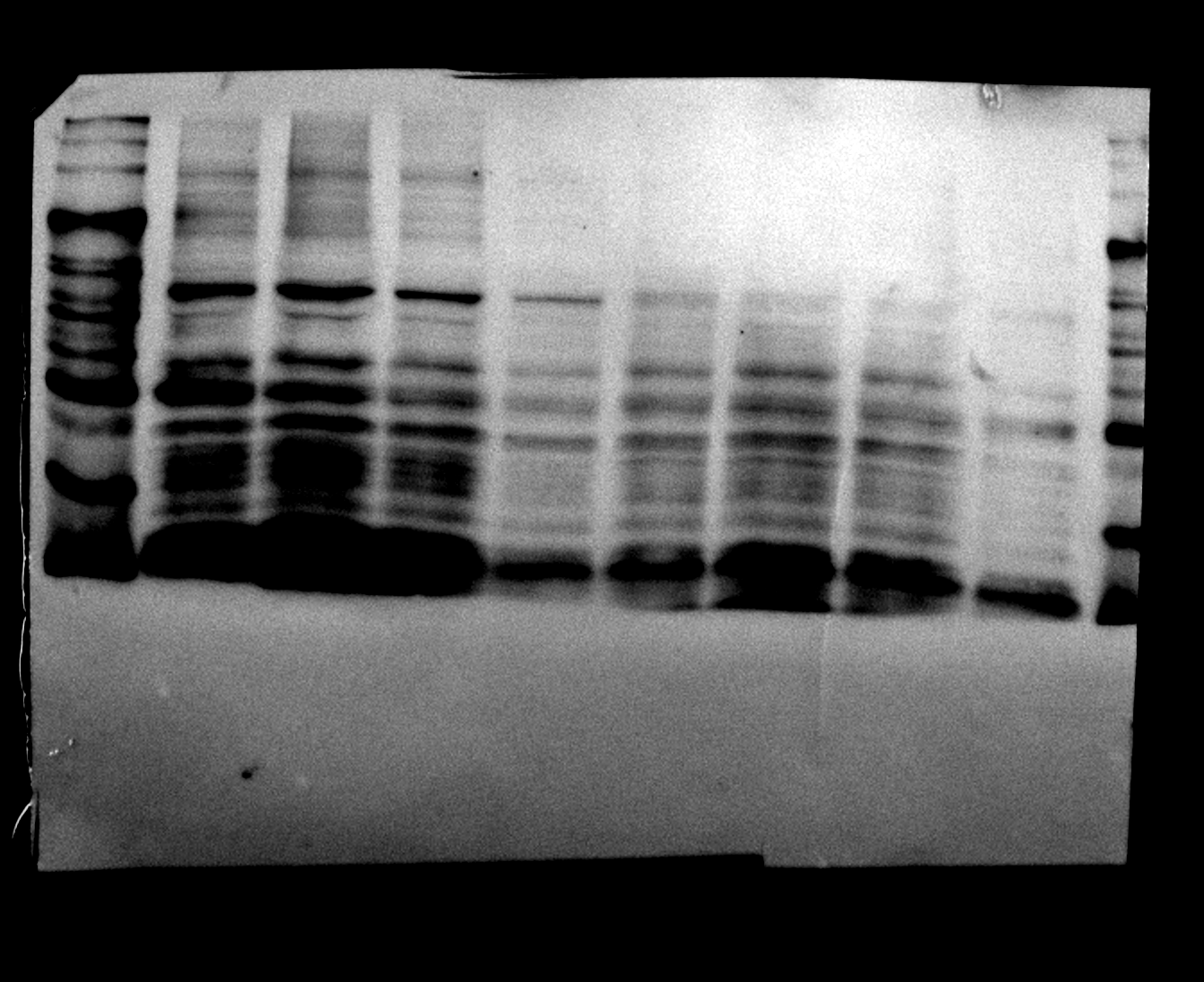

Supplement: Supplementary file 3 — Supplementary Information 3. [file 41598_2024_55043_MOESM3_ESM.zip › Supplementary material/Lo2-WB/IL-1B/1B.2.Tif]

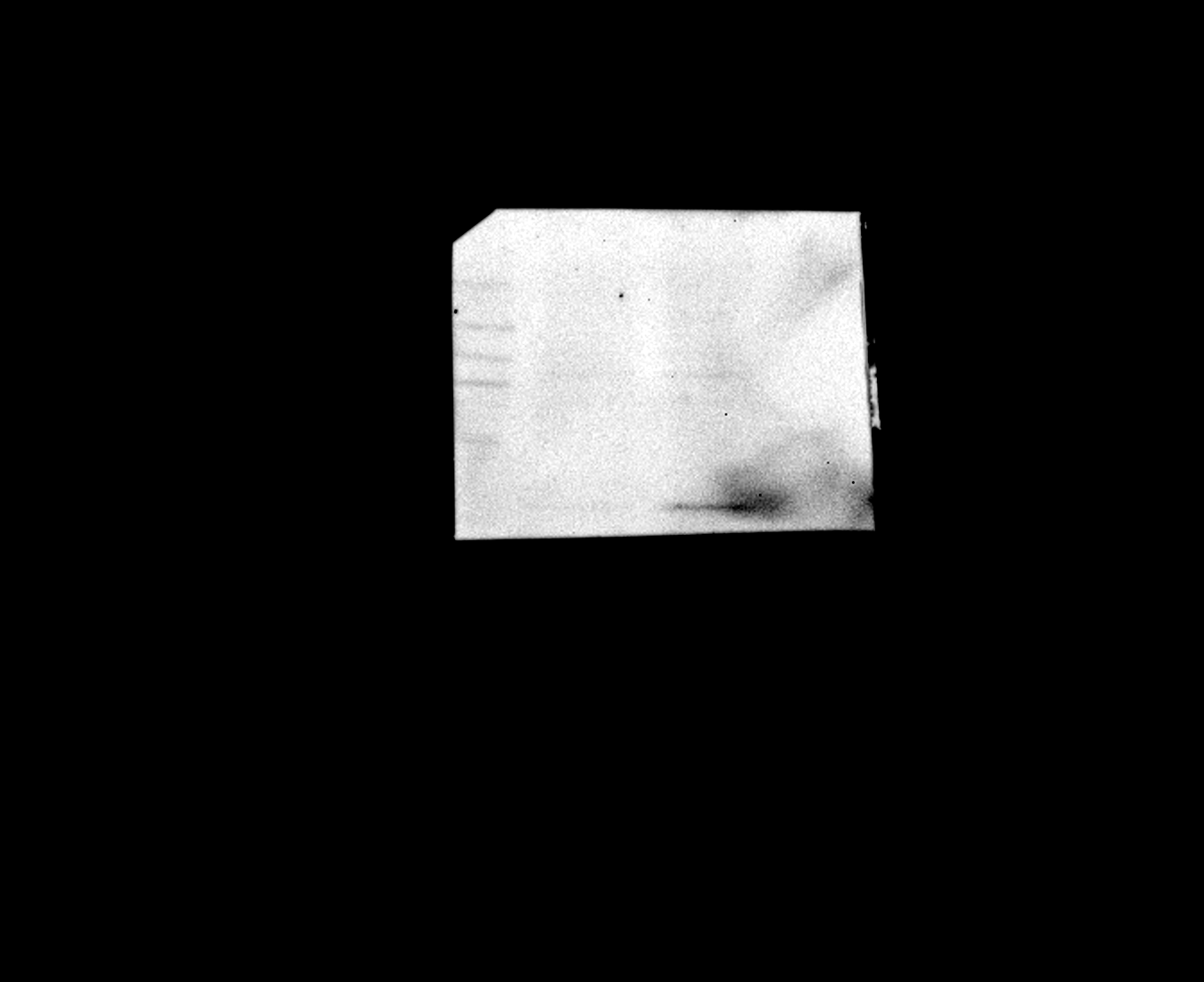

Supplement: Supplementary file 3 — Supplementary Information 3. [file 41598_2024_55043_MOESM3_ESM.zip › Supplementary material/Lo2-WB/IL-1B/IL-1B.Tif]

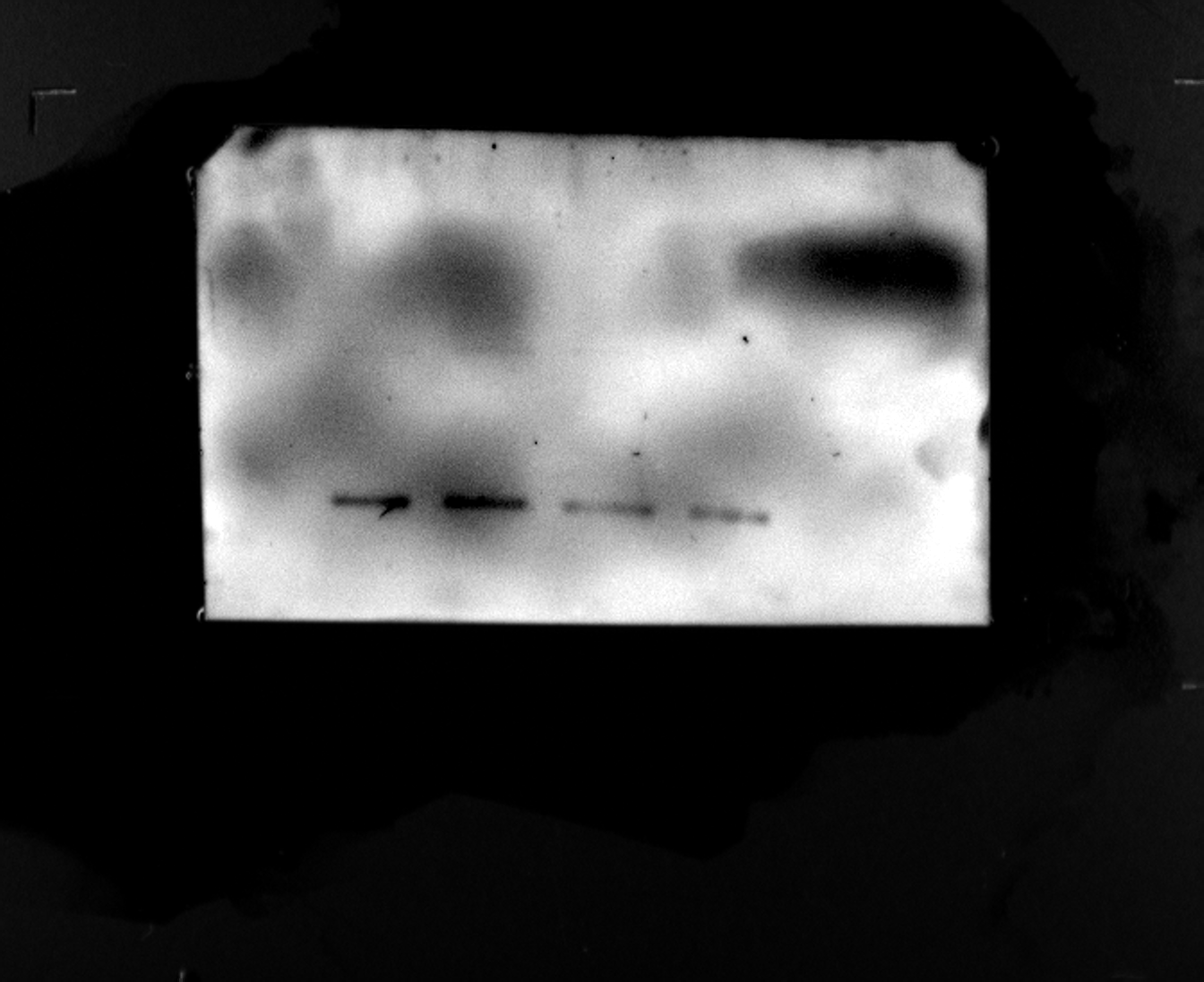

Supplement: Supplementary file 3 — Supplementary Information 3. [file 41598_2024_55043_MOESM3_ESM.zip › Supplementary material/Lo2-WB/IL-1B/ICAM1.Tif]

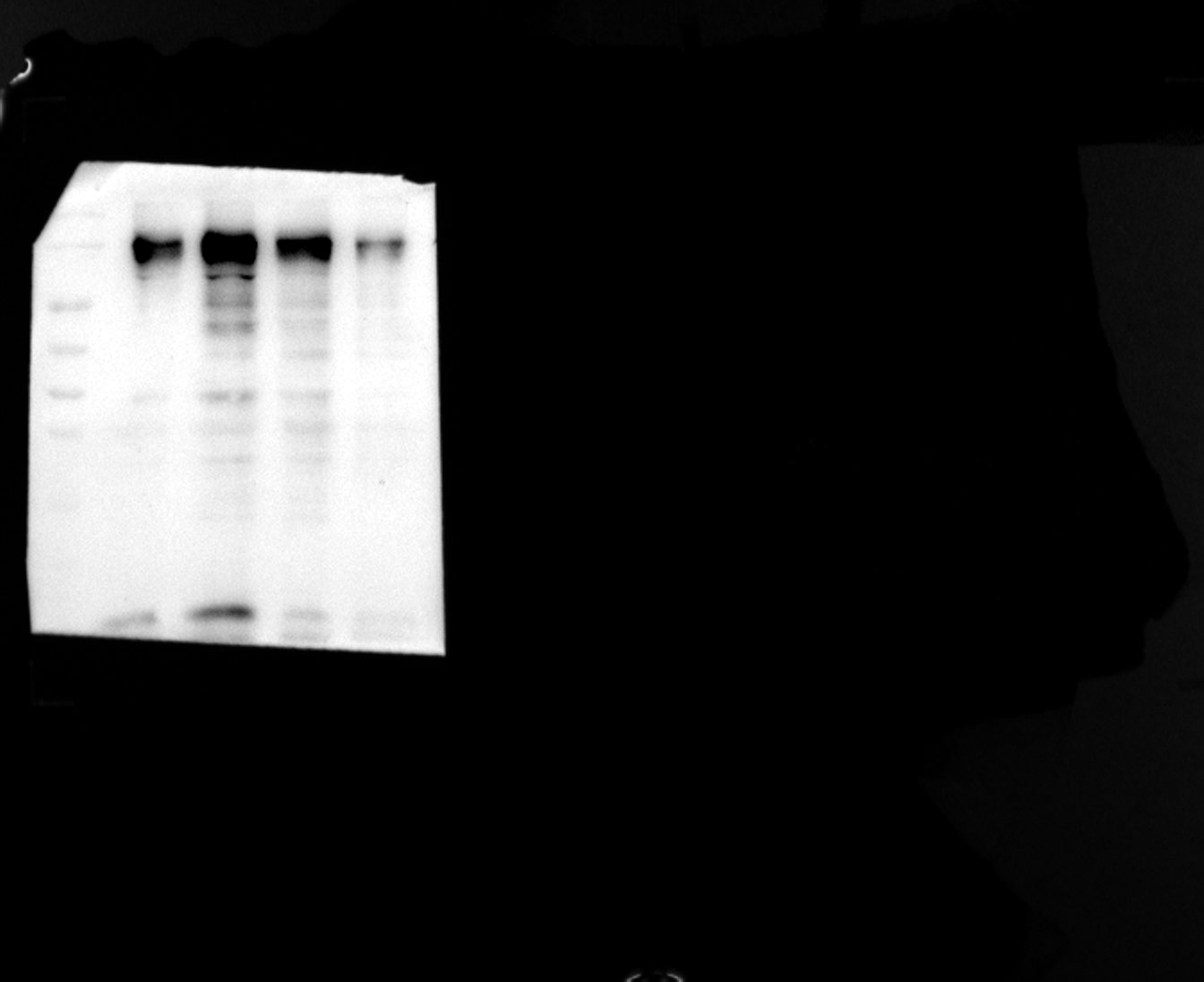

Supplement: Supplementary file 3 — Supplementary Information 3. [file 41598_2024_55043_MOESM3_ESM.zip › Supplementary material/Lo2-WB/NLRP3/NRF2.Tif]

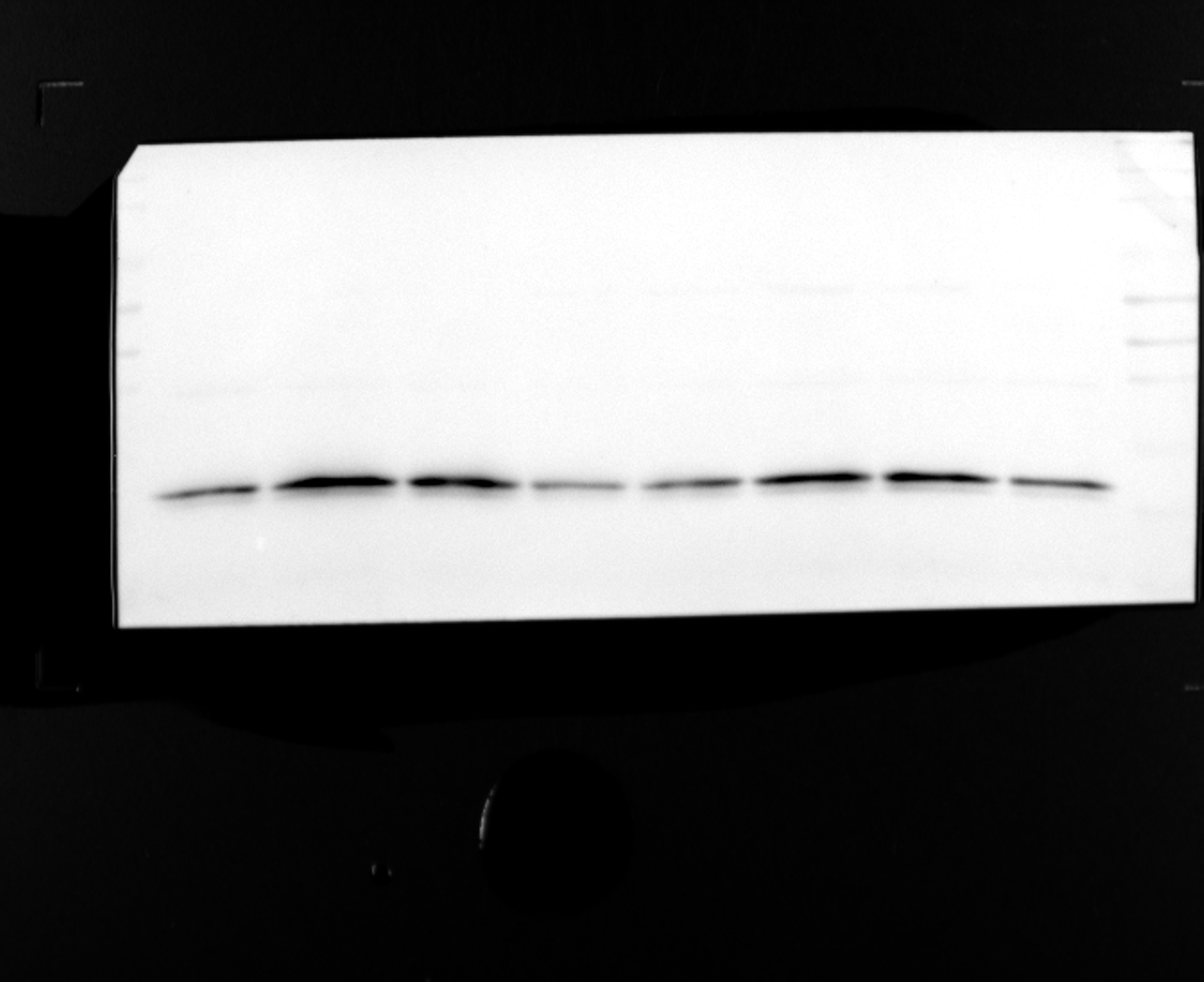

Supplement: Supplementary file 3 — Supplementary Information 3. [file 41598_2024_55043_MOESM3_ESM.zip › Supplementary material/Lo2-WB/NLRP3/NLRP3.Tif]

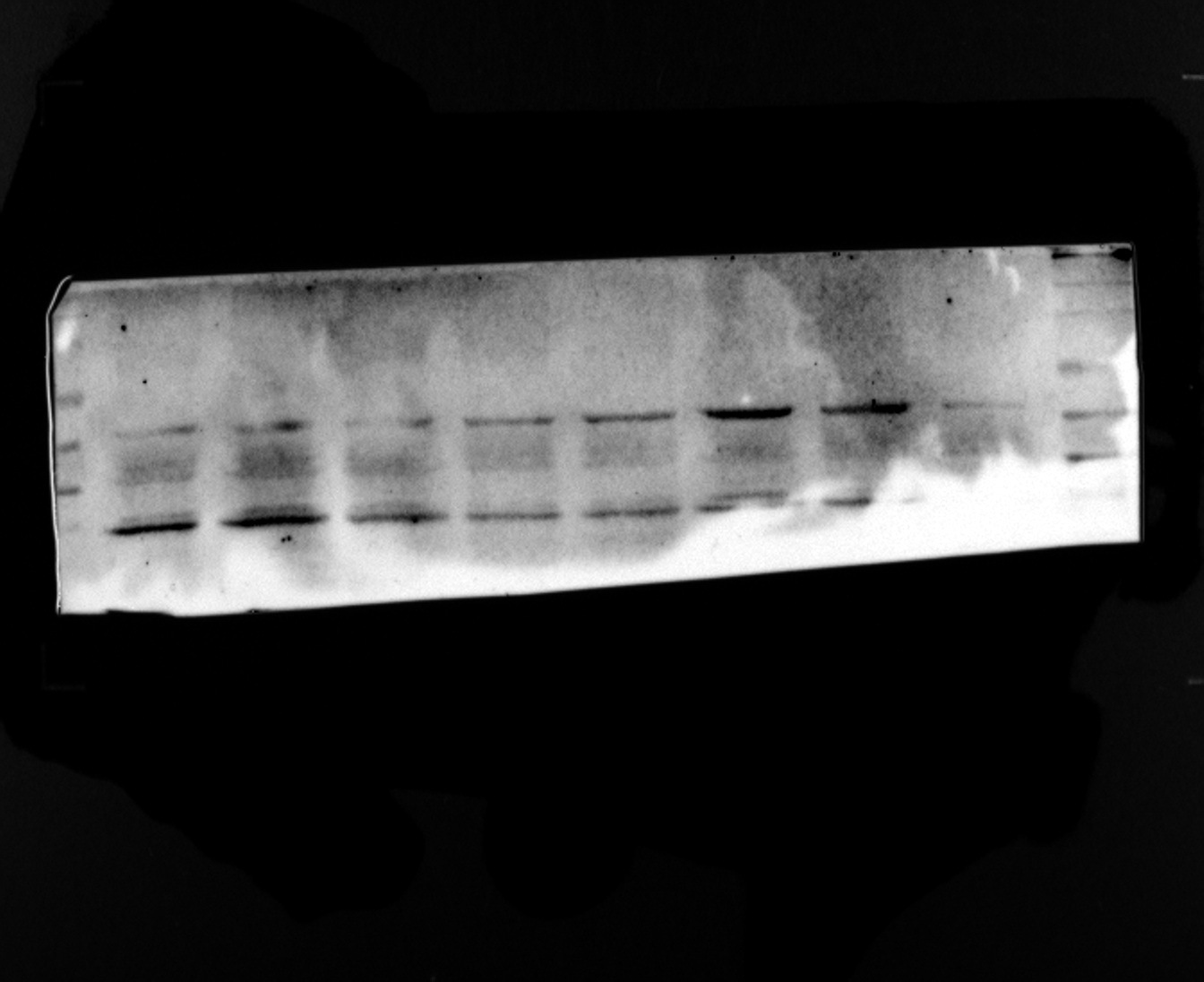

Supplement: Supplementary file 3 — Supplementary Information 3. [file 41598_2024_55043_MOESM3_ESM.zip › Supplementary material/Lo2-WB/NLRP3/NLRP3.2.Tif]

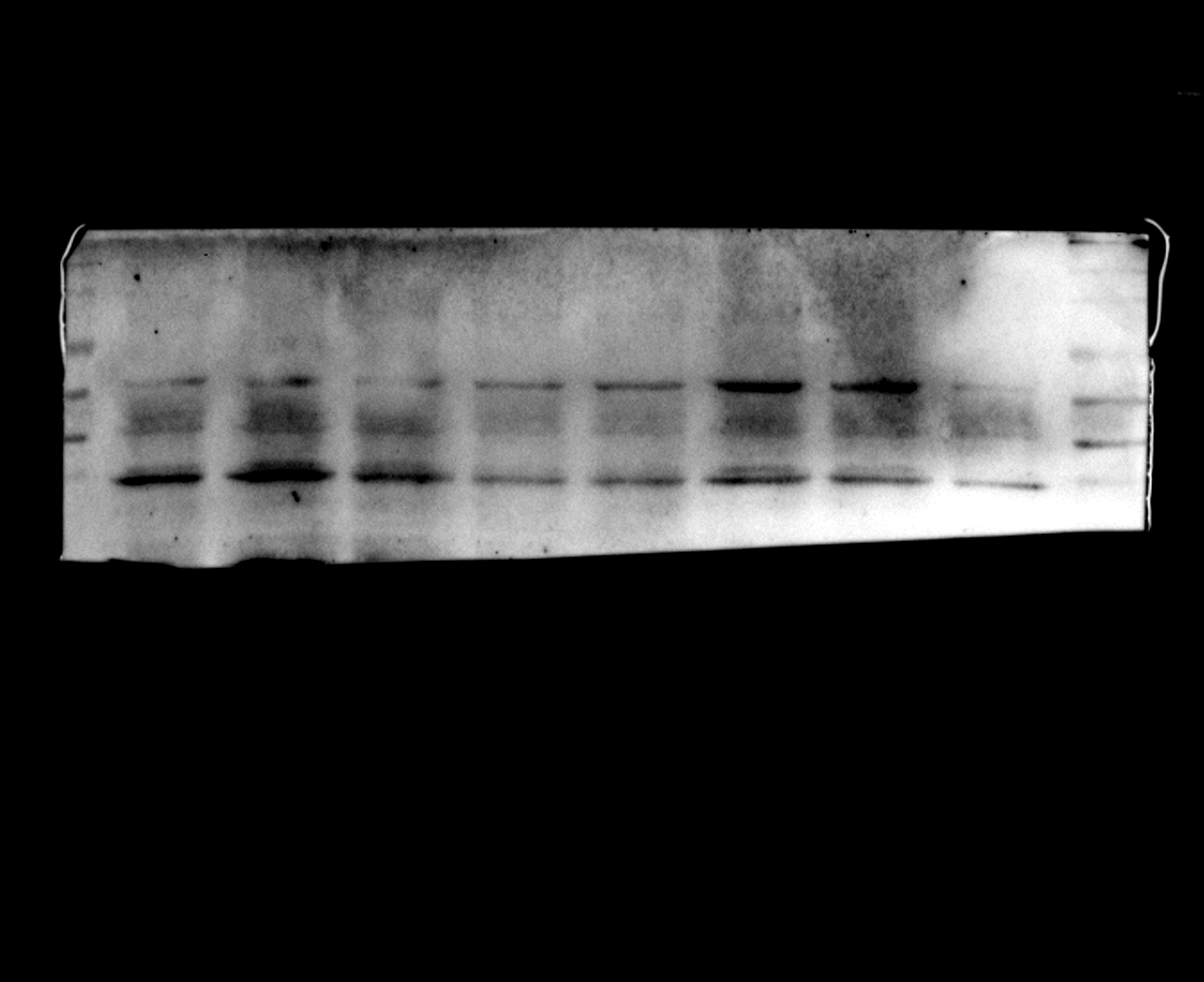

Supplement: Supplementary file 3 — Supplementary Information 3. [file 41598_2024_55043_MOESM3_ESM.zip › Supplementary material/Lo2-WB/NLRP3/NLRP3.3.Tif]

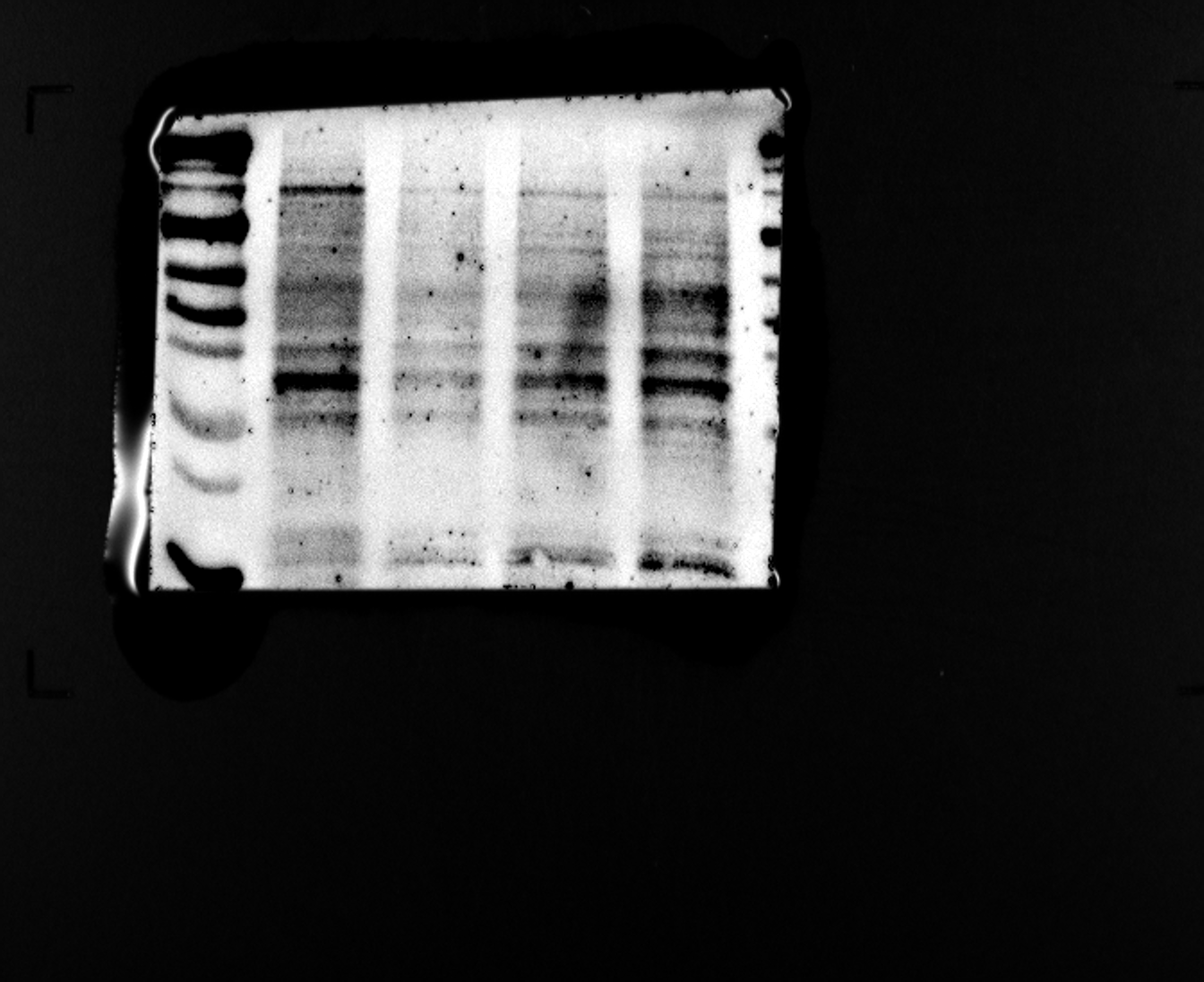

Supplement: Supplementary file 3 — Supplementary Information 3. [file 41598_2024_55043_MOESM3_ESM.zip › Supplementary material/Lo2-WB/HO-1/nrf2.Tif]

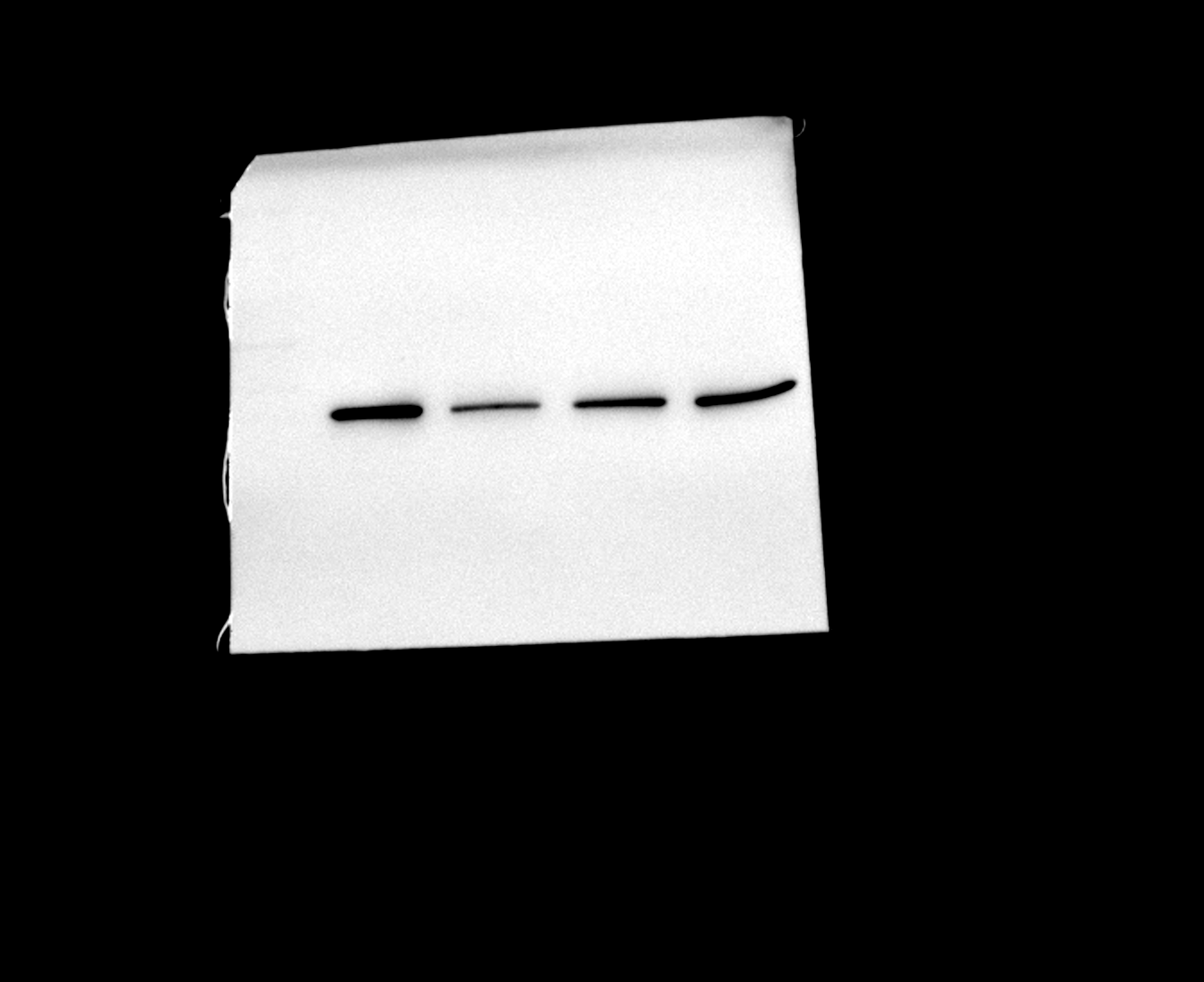

Supplement: Supplementary file 3 — Supplementary Information 3. [file 41598_2024_55043_MOESM3_ESM.zip › Supplementary material/Lo2-WB/HO-1/1πÇüHO-1.Tif]

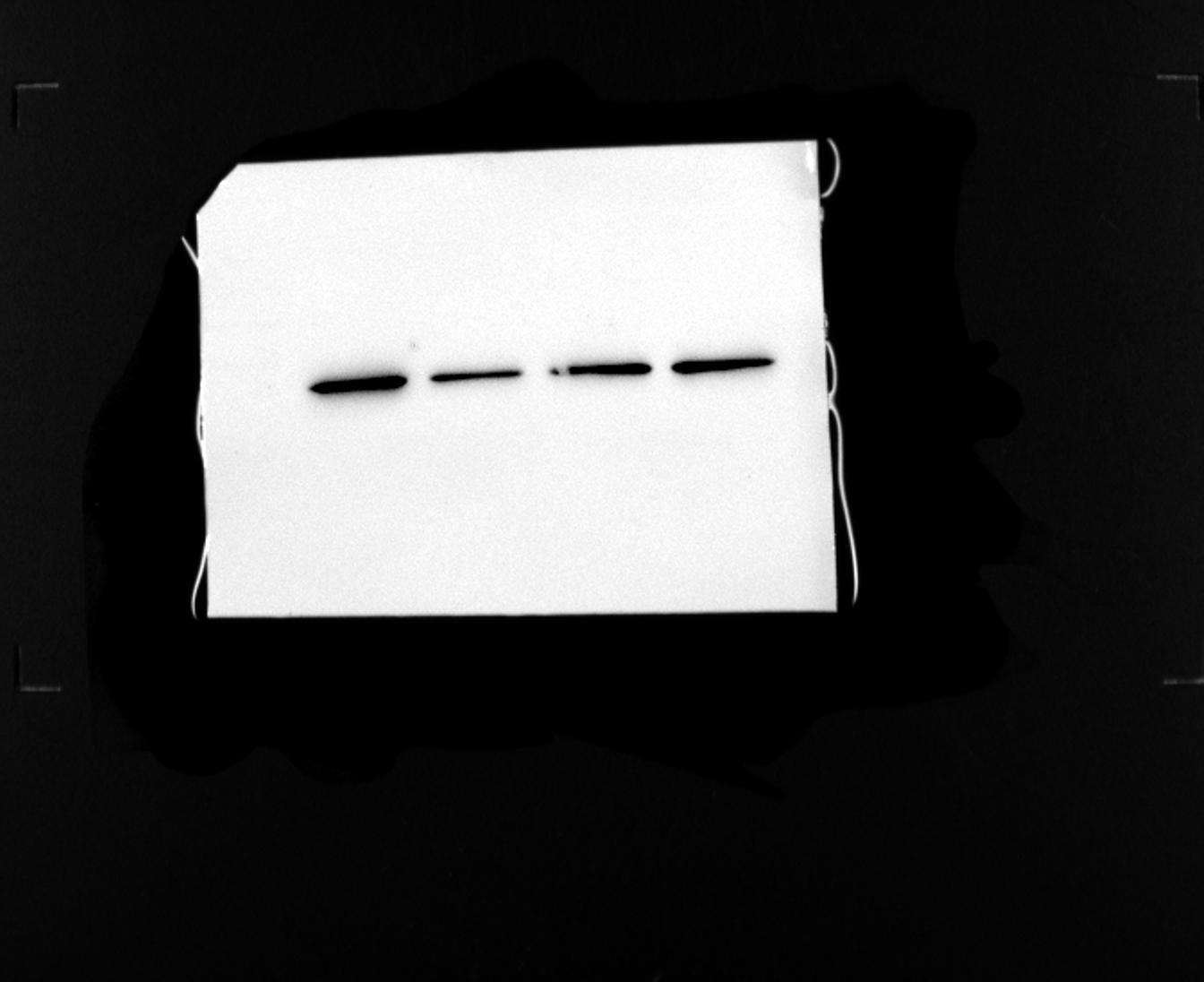

Supplement: Supplementary file 3 — Supplementary Information 3. [file 41598_2024_55043_MOESM3_ESM.zip › Supplementary material/Lo2-WB/HO-1/6πÇüHO-1.Tif]

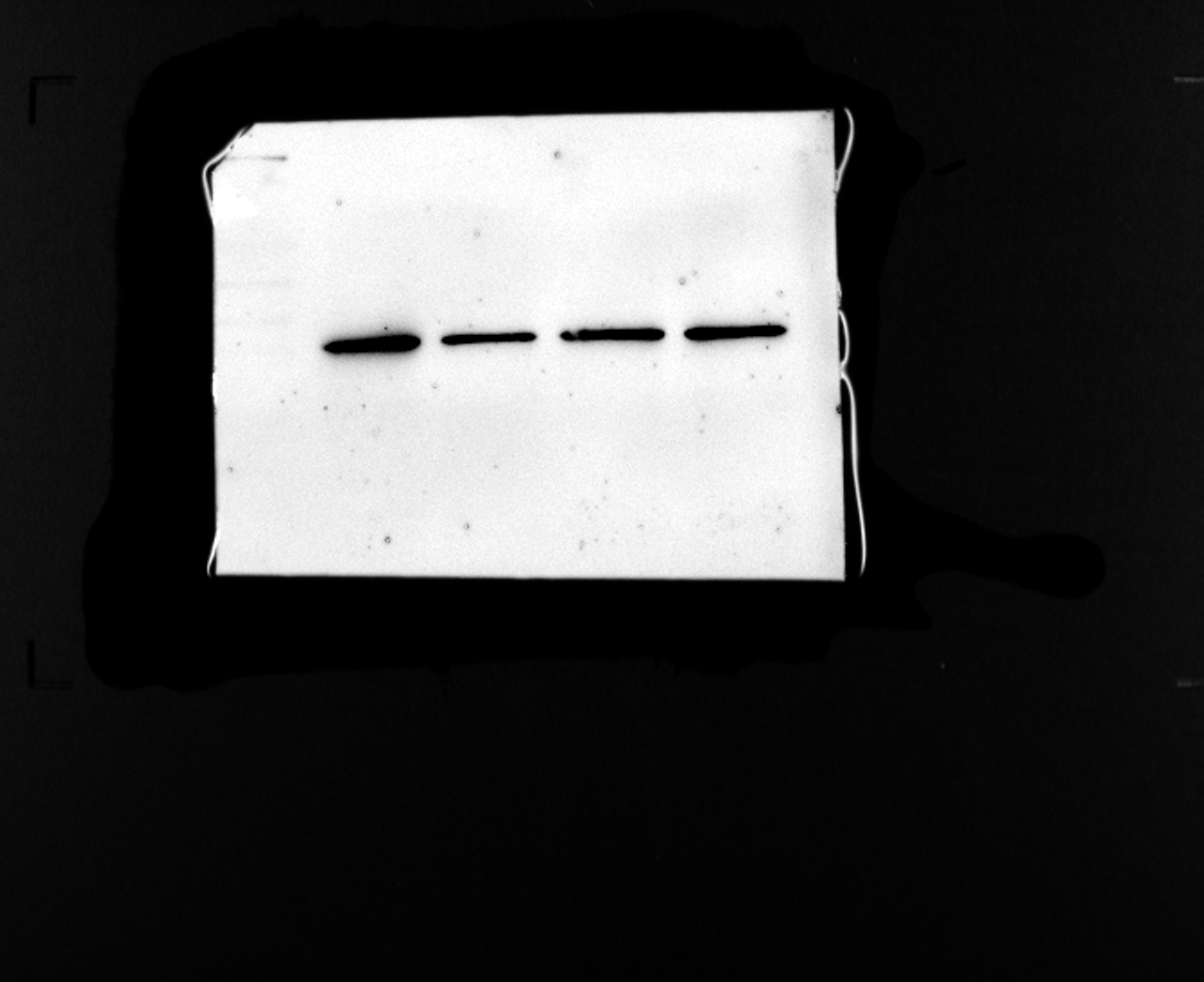

Supplement: Supplementary file 3 — Supplementary Information 3. [file 41598_2024_55043_MOESM3_ESM.zip › Supplementary material/Lo2-WB/HO-1/7πÇüHO-1.Tif]

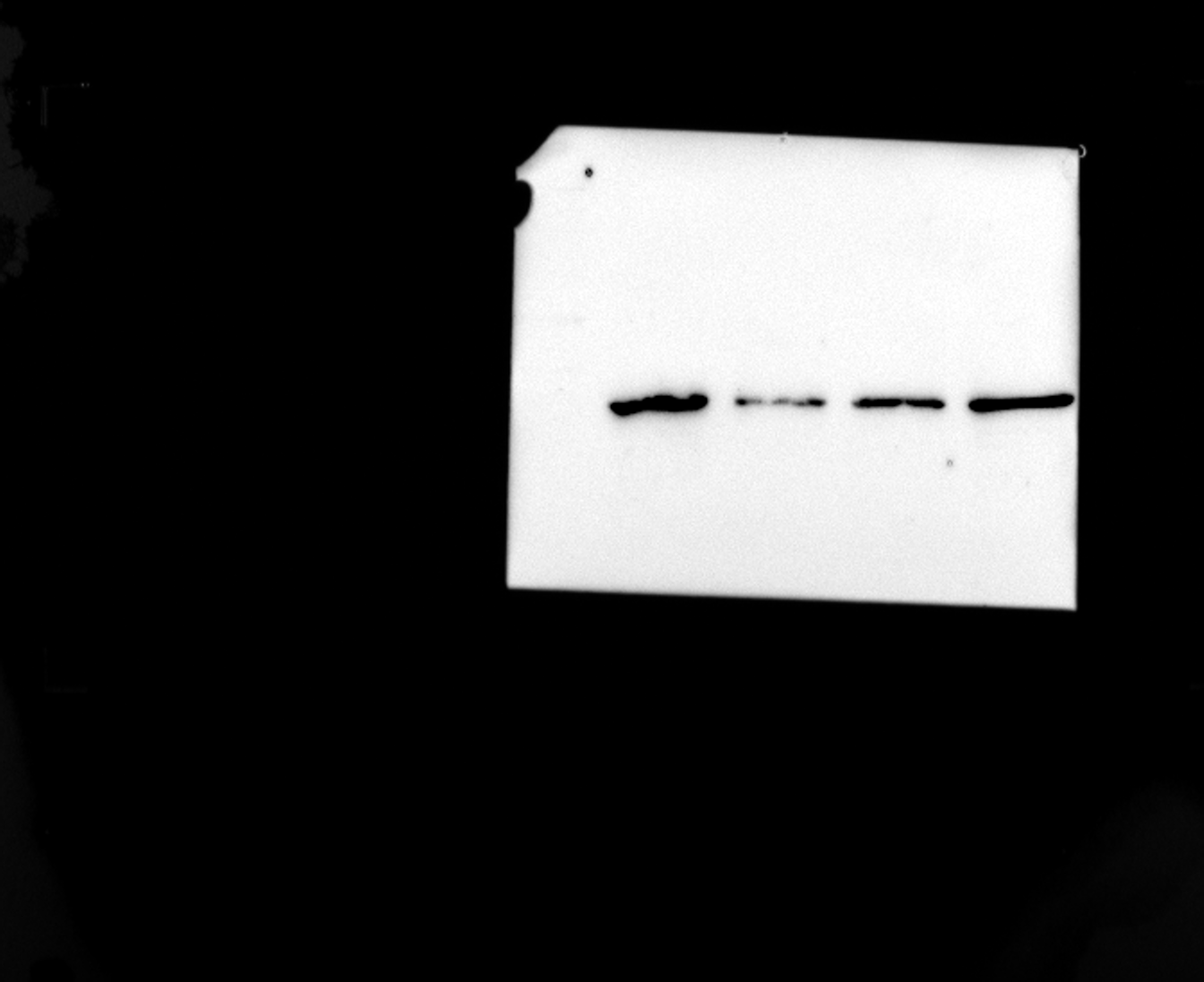

Supplement: Supplementary file 3 — Supplementary Information 3. [file 41598_2024_55043_MOESM3_ESM.zip › Supplementary material/Lo2-WB/HO-1/2πÇüHO-1.Tif]

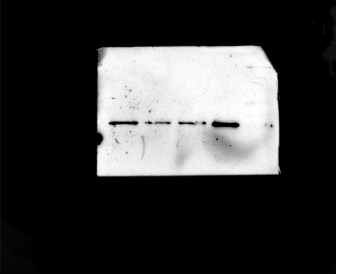

Supplement: Supplementary file 3 — Supplementary Information 3. [file 41598_2024_55043_MOESM3_ESM.zip › Supplementary material/Lo2-WB/HO-1/4πÇüHO-1.png]

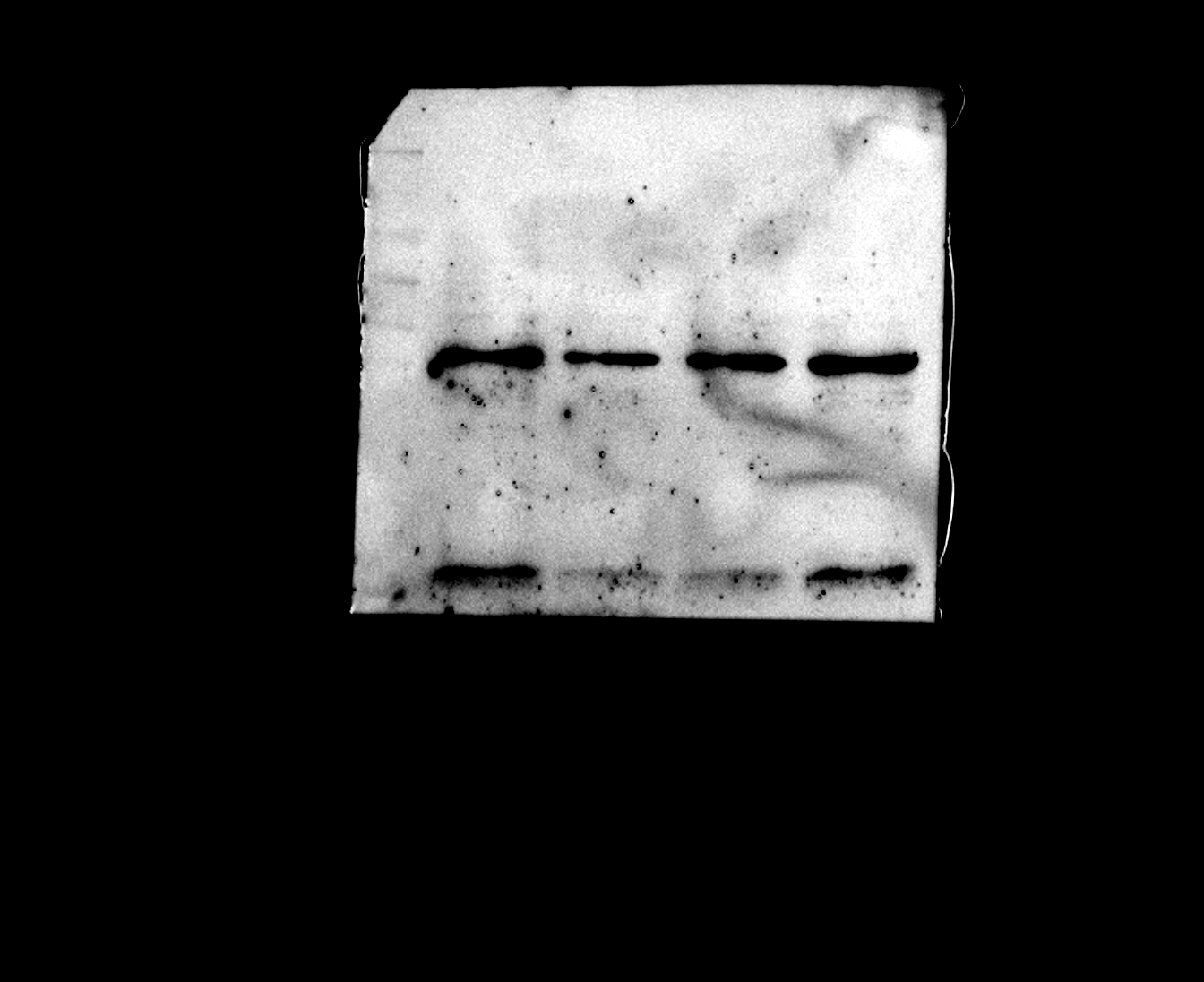

Supplement: Supplementary file 3 — Supplementary Information 3. [file 41598_2024_55043_MOESM3_ESM.zip › Supplementary material/Lo2-WB/HO-1/HO-1.Tif]

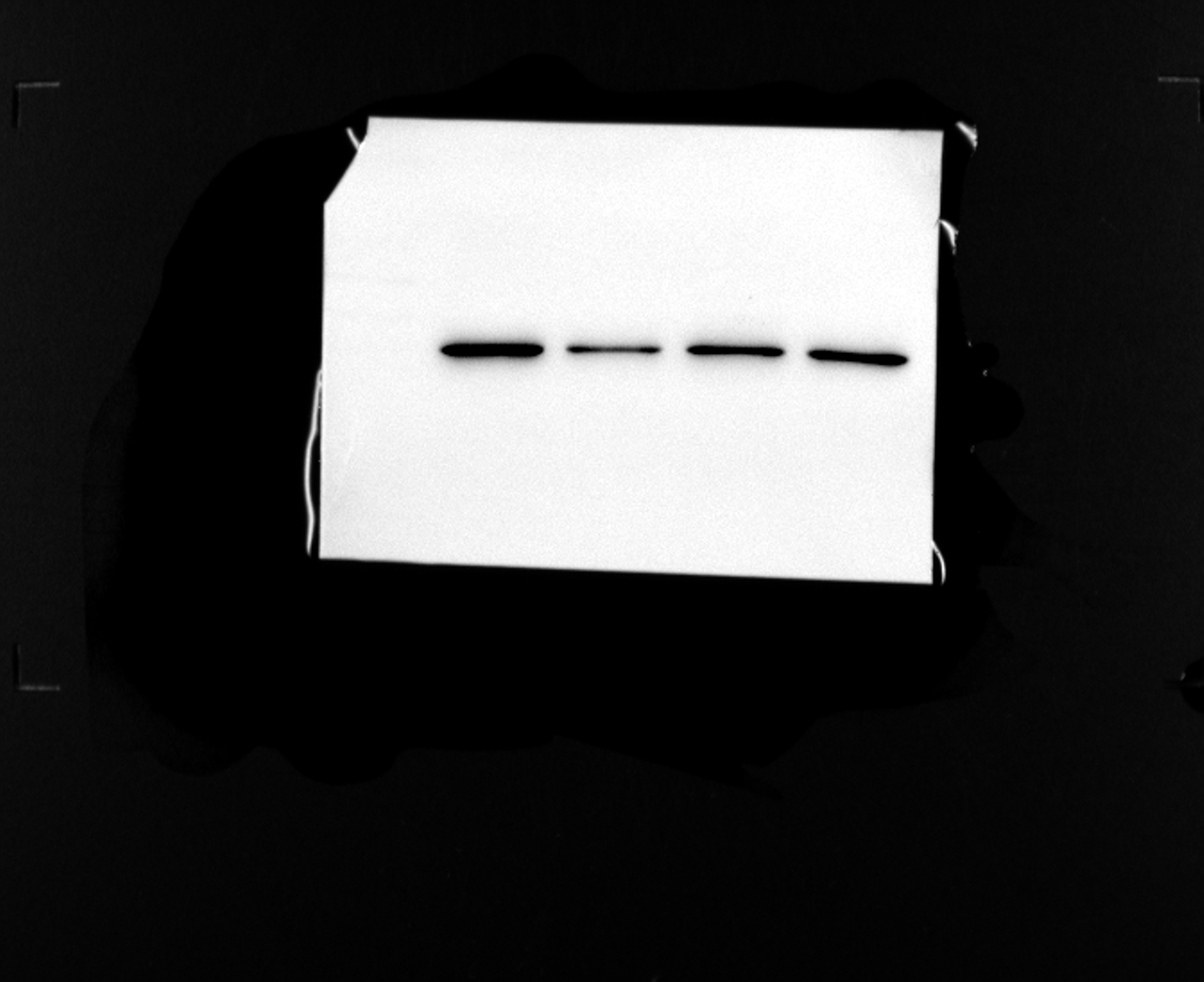

Supplement: Supplementary file 3 — Supplementary Information 3. [file 41598_2024_55043_MOESM3_ESM.zip › Supplementary material/Lo2-WB/HO-1/5πÇüHO-1.Tif]

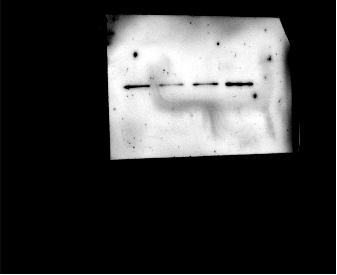

Supplement: Supplementary file 3 — Supplementary Information 3. [file 41598_2024_55043_MOESM3_ESM.zip › Supplementary material/Lo2-WB/HO-1/3πÇüHO-1.png]

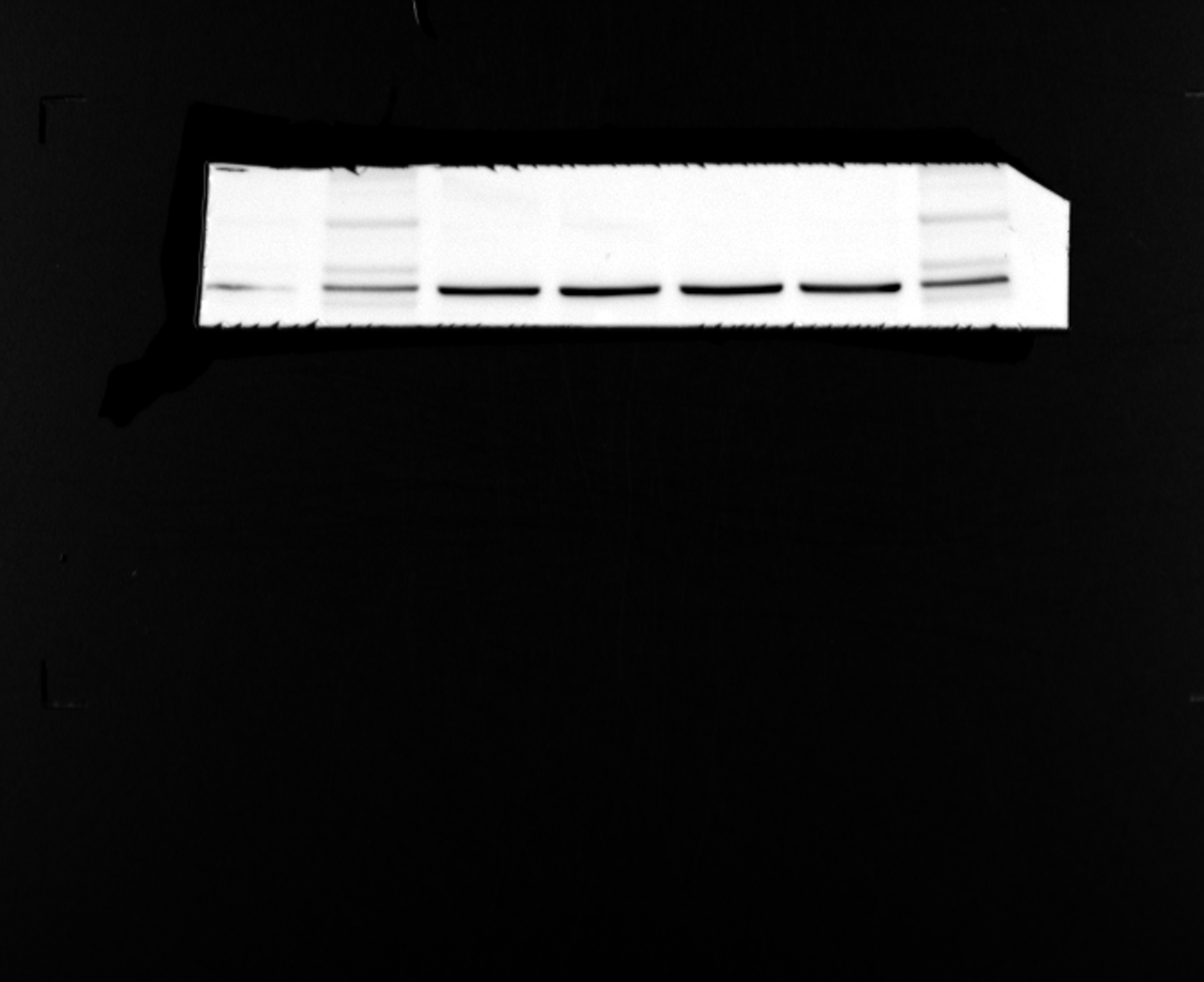

Supplement: Supplementary file 3 — Supplementary Information 3. [file 41598_2024_55043_MOESM3_ESM.zip › Supplementary material/Lo2-WB/GAPDH/GAPDH.Tif]

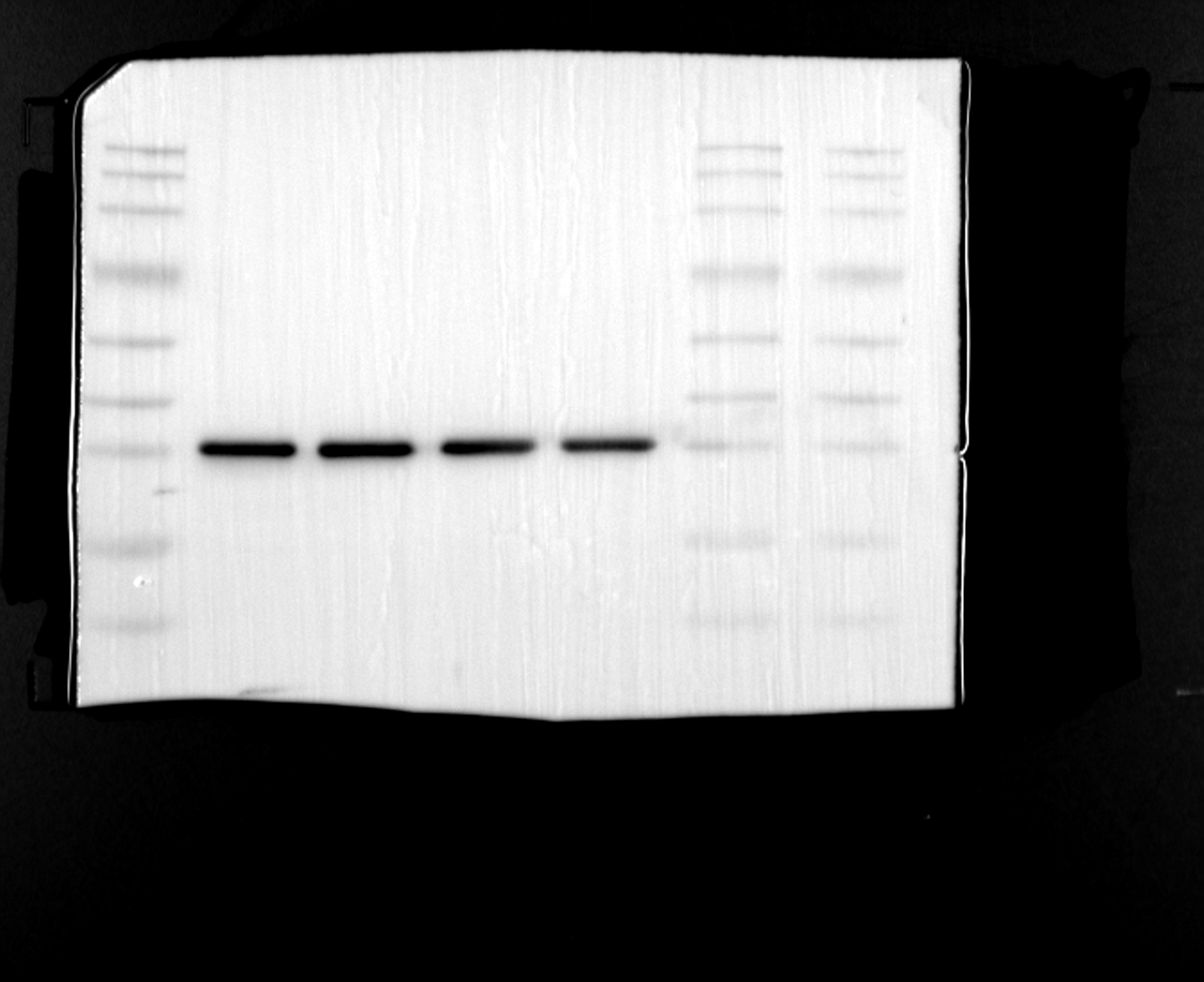

Supplement: Supplementary file 3 — Supplementary Information 3. [file 41598_2024_55043_MOESM3_ESM.zip › Supplementary material/Lo2-WB/GAPDH/GAPDH-2.Tif]

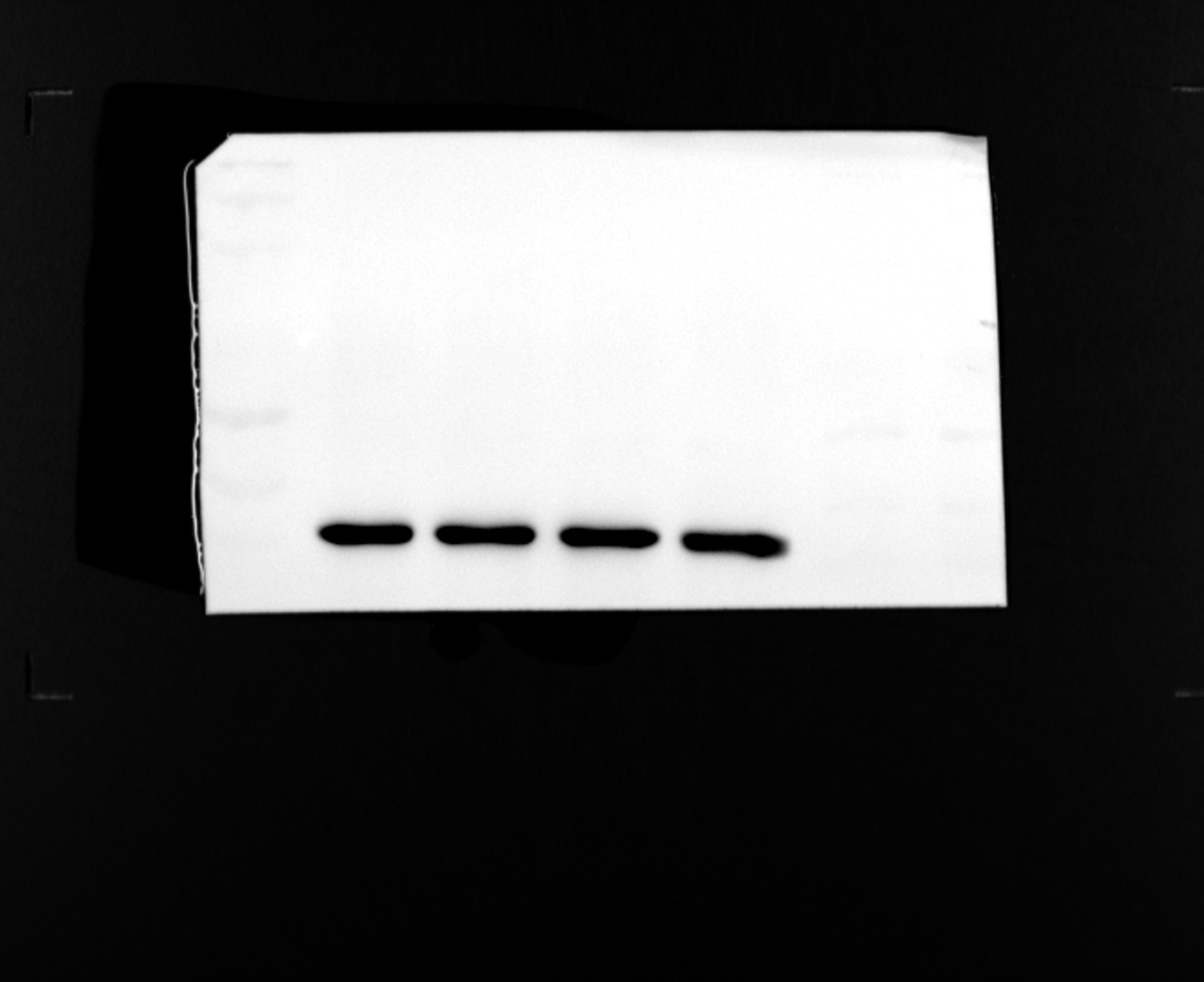

Supplement: Supplementary file 3 — Supplementary Information 3. [file 41598_2024_55043_MOESM3_ESM.zip › Supplementary material/Lo2-WB/GAPDH/GAPDH-.Tif]

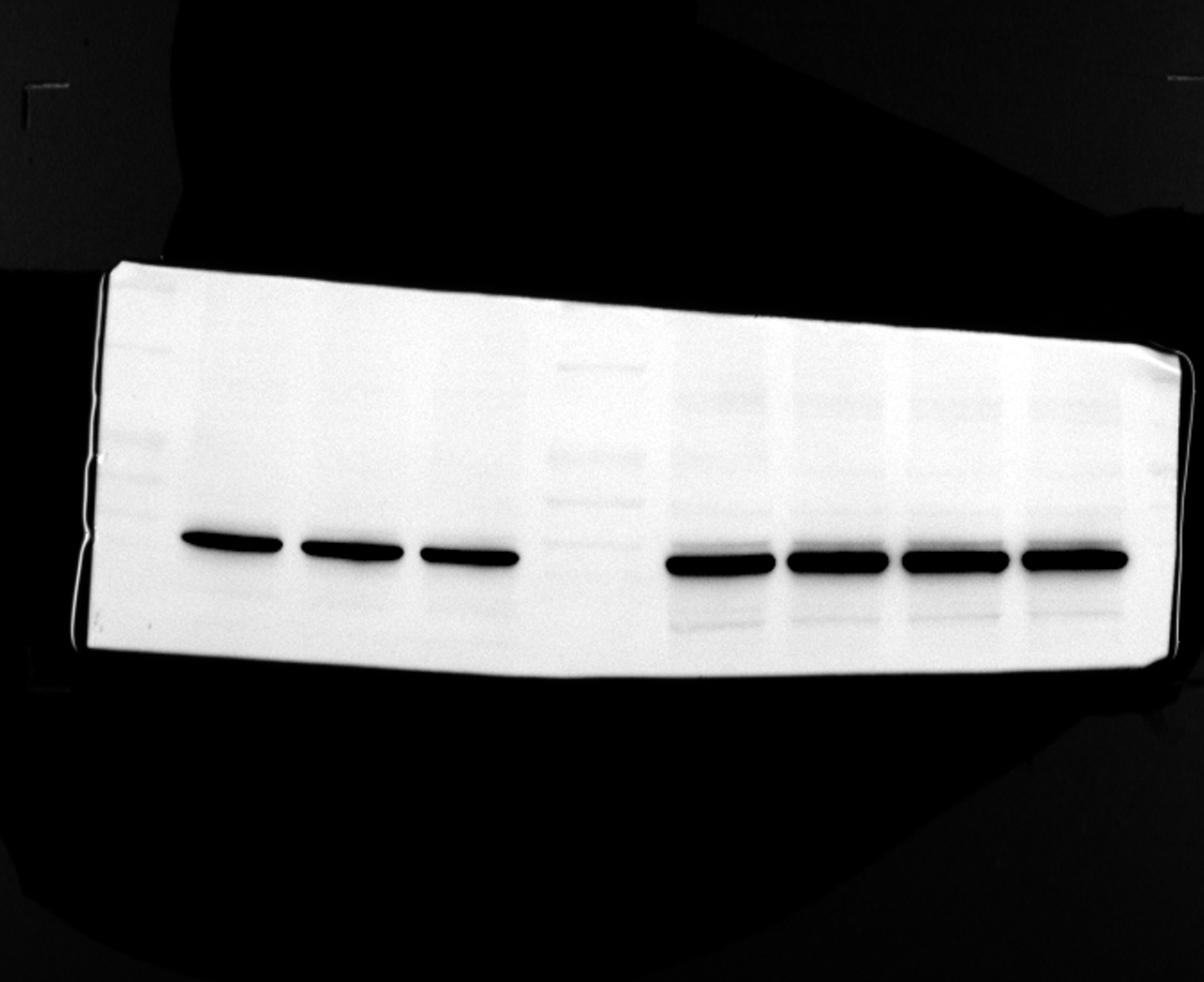

Supplement: Supplementary file 3 — Supplementary Information 3. [file 41598_2024_55043_MOESM3_ESM.zip › Supplementary material/Lo2-WB/GAPDH/GAPDH-(3+4).Tif]

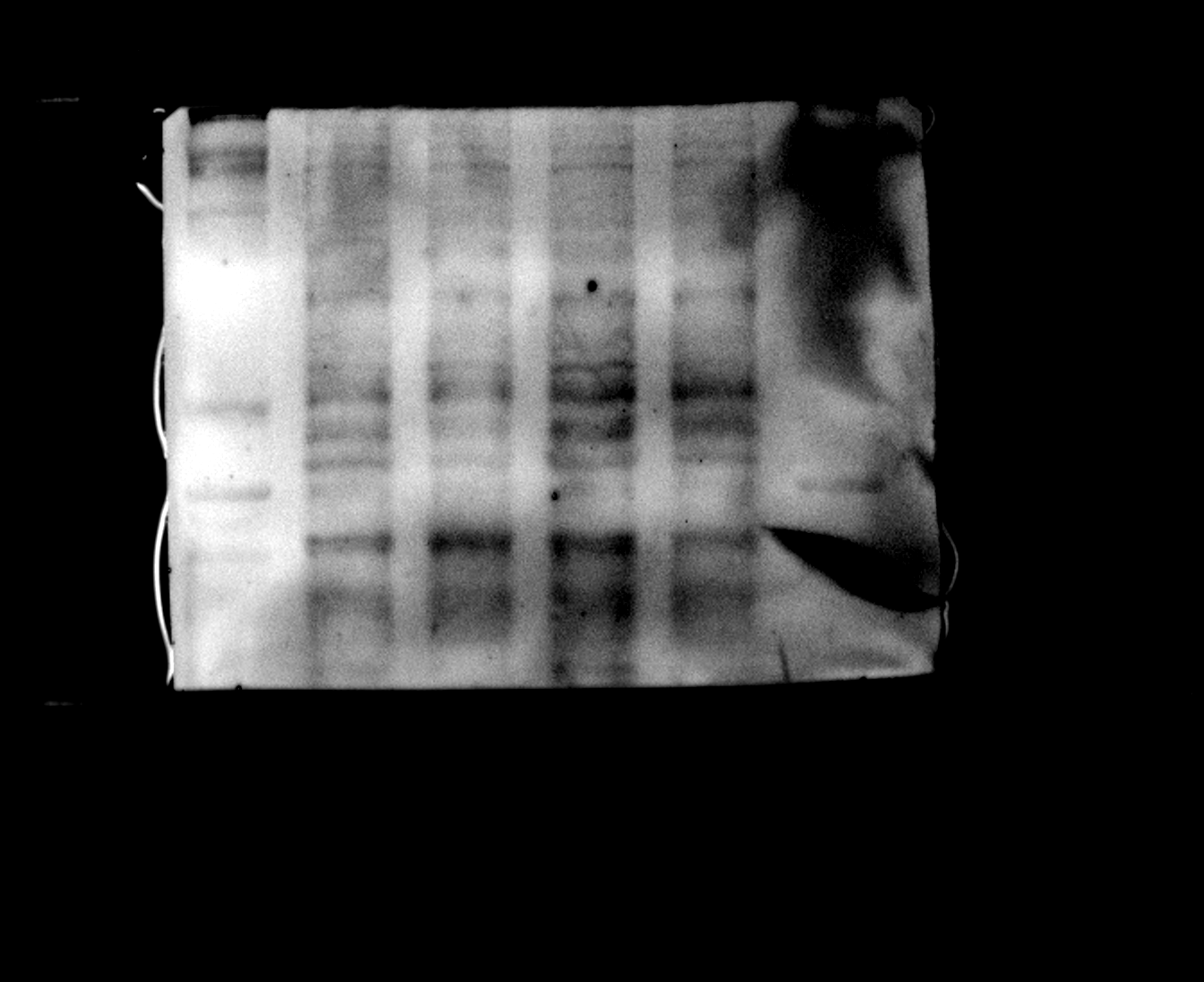

Supplement: Supplementary file 3 — Supplementary Information 3. [file 41598_2024_55043_MOESM3_ESM.zip › Supplementary material/Lo2-WB/IL-6/2πÇüIL-6.Tif]
